# Identification of the molecular subtypes and construction of risk models in neuroblastoma

**DOI:** 10.1038/s41598-023-35401-3

**Published:** 2023-07-21

**Authors:** Enyang He, Bowen Shi, Ziyu Liu, Kaili Chang, Hailan Zhao, Wei Zhao, Hualei Cui

**Affiliations:** 1 Tianjin Medical University, Tianjin, China; 2 Tianjin Children’s Hospital, Tianjin, China; 3 Graduate School of Tianjin Medical University, Tianjin, China; 4 Basic Medical Sciences School of Tianjin Medical University, Tianjin, China

**Keywords:** Cancer, Computational biology and bioinformatics, Biomarkers, Molecular medicine

## Abstract

The heterogeneity of neuroblastoma directly affects the prognosis of patients. Individualization of patient treatment to improve prognosis is a clinical challenge at this stage and the aim of this study is to characterize different patient populations. To achieve this, immune-related cell cycle genes, identified in the GSE45547 dataset using WGCNA, were used to classify cases from multiple datasets (GSE45547, GSE49710, GSE73517, GES120559, E-MTAB-8248, and TARGET) into subgroups by consensus clustering. ESTIMATES, CIBERSORT and ssGSEA were used to assess the immune status of the patients. And a 7-gene risk model was constructed based on differentially expressed genes between subtypes using randomForestSRC and LASSO. Enrichment analysis was used to demonstrate the biological characteristics between different groups. Key genes were screened using randomForest to construct neural network and validated. Finally, drug sensitivity was assessed in the GSCA and CellMiner databases. We classified the 1811 patients into two subtypes based on immune-related cell cycle genes. The two subtypes (Cluster1 and Cluster2) exhibited distinct clinical features, immune levels, chromosomal instability and prognosis. The same significant differences were demonstrated between the high-risk and low-risk groups. Through our analysis, we identified neuroblastoma subtypes with unique characteristics and established risk models which will improve our understanding of neuroblastoma heterogeneity.

## Introduction

Neuroblastoma, a tumor of sympathetic origin, is the most common extracranial solid tumor in early childhood. Neuroblastoma account for 7–8% of childhood malignancies with a heterogeneous clinical course from local or spontaneous regression to extensive metastatic disease^1^. The etiology of the disease is complex and diverse, with multiple signaling pathways involved. The mammalian target of rapamycin (mTOR) pathway promotes neuroblastoma cell survival and chemoresistance^2^. The WNT signaling pathway, on the other hand, increases MYC levels in patients without MYCN amplification^3^. Additionally, the ALK signaling pathway is the primary oncogene target pathway in sporadic and familial neuroblastoma cases^4^.

As we all know, unrestricted proliferation is a common feature of malignant tumors and is closely related to cell cycle dysregulation^5^. The cell cycle is a complex process that contains four phases: Gap 1 (G1), DNA-synthesis (S), Gap 2 (G2) and mitosis (M). Cell cycle proteins and cell cycle protein-dependent kinases (CDK) regulate the progression of cell cycle phases^6^. At the same time, whether each cell cycle event is completed, correctly or not, is subject to a cellular checkpoint monitoring mechanism^7^. The DNA damage response and the Mitotic Spindle Checkpoint play a key role in maintaining the health of the organism. As known, the p53 tumor suppressor is involved in multiple cell cycle checkpoints^8^. And abnormalities in p53 can lead to cancer development and progression through multiple pathways^9^.

Abnormalities in cell cycle-related mechanisms likewise play an important role in the onset and development of neuroblastoma. Increased MYCN copy number was detected in 25% of patients with neuroblastoma^10^, which was strongly associated with an unfavorable clinical prognosis^11^. Meanwhile, MYCN can accelerate cell proliferation^12^, which may be related to cell cycle protein-dependent kinase 4 (CDK4)^13^. For patients with neuroblastoma without MYCN amplification, it is more likely to exhibit chromosomal alterations and again leads to poor prognostic outcomes^14^. This may be related to the absence of a common region that codes a series of proteins that play a role in the DNA damage response (DDR)^15^. As the research becomes more in-depth, chromosome instability plays an important role in the development and progression of the disease^16^. Study found that unbalanced loss of heterozygosity (LOH) in chromosome 11q and LOH in chromosome 1p36 are independent risk factors for poor prognosis in patients with neuroblastoma^17^. 17q gain was also associated with poorer overall survival (OS)^14^. Chromosomal instability has also been observed during early human embryogenesis^18^. However, the underlying mechanism ensures that the cell cycle proceeds correctly. Therefore, understanding the mechanisms involved in the cell cycle is crucial to our understanding of neuroblastoma.

Various etiologies lead to the variability among individual patients. The heterogeneity of patients poses a great challenge for individualized treatment. In order to evaluate patients for stratification to guide treatment, classification methods based on multiple biological indicators have been proposed and applied. Stage, age, histologic category, grade of tumor differentiation, the status of the MYCN oncogene, chromosome 11q status, and DNA ploidy were used as the classification basis for the International Neuroblastoma Risk Group Staging System^19^. Segmental chromosomal aberrations (SCA) have also been studied as an additional genomic biomarker in combination with INSS staging to guide treatment^20^. Based on the concept of stratified treatment, the prognosis of neuroblastoma patients is gradually improving. Over the past few decades, the 5-year survival rate for patients with metastatic neuroblastoma has increased from less than 20% to over 50% through a combination of therapies including immunotherapy, stem cell therapy, etc^21^. Although these staging plays a role in assessing patients and guiding treatment, clinical use is somewhat limited. With the development of gene chip technology, how to stratify patients at the genetic level to guide targeted therapy is an urgent issue.

Given the role of cell cycle abnormalities in the pathogenesis of neuroblastoma, it is essential to understand the causes of Chromosomal instability (CIN) in neuroblastoma and to study the chromosome and centrosome segregation, spindle machinery and DNA repair^1^. This facilitates the exploration of individualized treatment of neuroblastoma patients with drugs that target the cell cycle. The aim of our study is to explore molecular subtyping in tumor patients by analyzing cell cycle gene expression levels to further refine individualized patient stratification management. Molecular subtyping and risk scores will be used to guide individualized patient treatment and thus improve patient prognosis.

## Results

### Identification of a set of 924 immune-related cell cycle genes

**Figure 1:**
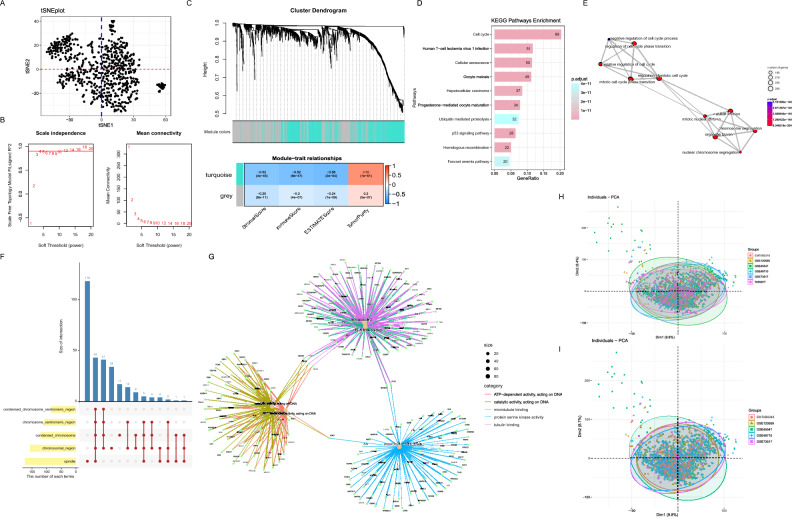
Identification and functional analysis of IRCCGs. (**A**) The results of the t-SNE algorithm show heterogeneity in the patients. (**B**) Analysis of network topology for various soft-thresholding powers. The left panel shows the scale-free fit index as a function of the soft-thresholding power. The right panel displays the mean connectivity as a function of the soft-thresholding power. Based on the scale-free fit index greater than 0.9, we chose 4 as the soft thresholding power. (**C**) At the top is Clustering dendrogram of genes with assigned module colors. At the bottom is Module-trait associations. Each cell contains the corresponding correlation and P value. The darker the color of the cell, the higher the correlation. (**D**) Results of KEGG enrichment. The numbers in the graph indicated the counts of the pathway. (**E**) Results of Biological Process enrichment. The line between dots indicated the presence of identical genes between pathways. (**F**) The top 5 pathways of Cellular Component enrichment was demonstrated. The length of the yellow bar indicated the number of pathway genes. The height of the blue bar indicated the number of intersecting genes. (**G**) The first 5 enriched to Molecular Function terms and the genes in the terms. (**H**,**I**) Principal component analysis (PCA) of the gene expression in datasets. The visualization of patients by scatter plots were based on the top two Dims of gene expression profiles with the removal of batch effect.

First, the t-SNE algorithm classified the 643 patients in GSE45547 into different regions based on gene expression levels, indicating heterogeneity among patients. This result suggested that the disease can be further subdivided into molecular subtypes (Fig. 1A). Consideration of the close correlation of disease with the cell cycle and immunity, to assess the level of infiltration of immune and stromal cells involved in the tumor microenvironment (TME) of GSE45547, the algorithm ESTIMATE was applied based on transcriptomic data from 643 samples. The results were also incorporated into the WGCNA algorithm as clinical information in the search for immune-related cell cycle genes (Supplementary Fig. S1A). Subsequently, the scale-free co-expression network was obtained by WGCNA of 1740 cell cycle gene expressions from 643 samples with immunization results (Fig. 1B). Two gene modules were generated with a power of 4 as the optimal soft threshold (Fig. 1C). Among these modules, the turquoise module exhibited the highest correlation with the result of ESTIMATE and was considered as “Immune-related cell cycle genes (IRCCGs) module”. And there were 924 genes in the turquoise module (a detailed list of genes could be available in the Supplementary Material). We further explored the function of IRCCGs by enrichment analysis. KEGG enrichment results for IRCCGs showed links to both immune and cell cycle-related pathways (Fig. 1D). The results enriched in Biological Process showed that cell cycle regulation and nuclear division were involved (Fig. 1E). The gene products of IRCCGs play a role in the spindle and chromosomal region (Fig. 1F). For Molecular Function enrichment results showed that pathways such as tubulin binding and microtubule binding were involved (Fig. 1G).

To make the results of the study more objective and generalizable, GSE45547, GSE49710, GSE73517, GSE120559, E-MTAB-8248 and GDC TARGET-NBL data were integrated for analysis. In total, 16,978 genes from 1811 patients were jointly detected. Before the removal of batch effect, the result of principal component analysis (PCA) showed that the samples were clustered by batches (Supplementary Fig. S1B). On the contrast, the results after data processing show that cross-platform normalization has been successful in eliminating batch effects (Fig. 1H). In the normalized data, the intersection of 16,978 genes with Immune-related cell cycle genes was 913 genes. In total, 11 genes from 924 IRCCGs were not included in the follow-up analysis. This was due to the fact that different microarrays have different probes and the common genes were selected for the combined analysis. Considering that the microarray data were all from the same platform, the five microarray datasets were normalized and included in the subsequent study as a whole (Supplementary Fig. S1C, Fig. 1I).

### Two distinct cell cycle subtypes were identified with IRCCGs

**Figure 2:**
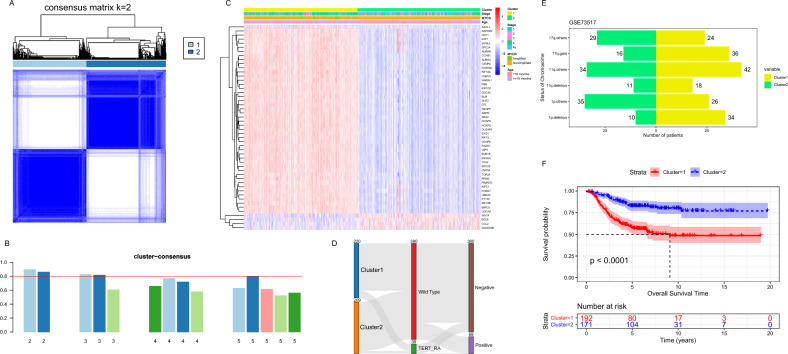
Identification of subtypes and clinical correlations of subtypes. (**A**) Consensus matrix heatmap with cluster count of 2. (**B**) The bar-plots represent the consensus scores for subgroups and we chose the results with consensus scores greater than 0.8. (**C**) Heatmap of Top 50 Immune-related cell cycle genes levels and distribution of age, MYCN status, and INSS stage in the two clusters. (**D**) The Sankey diagram showing whether TERT was rearrangement and whether APB existed. (**E**) The bar chart showed the distribution of chromosomal abnormalities in the two clusters. (**F**) The Kaplan–Meier curves showed the OS time of the two clusters of patients inside the E-MTAB-8248 and the TARGET datasets.

Based on the expression matrix after removing batch effect of 913 IRCCGs, the all 6 datasets (n = 1811) were divided into two distinct cell cycle clusters by consensus clustering (Fig. 2A), an unsupervised clustering method with the k value of 2 (Supplementary Fig. S2A–C). There were 871 patients in Cluster I and 940 patients in Cluster II. Moreover, the cluster consensus score for each subgroup was higher than 0.8 only in two-subgroup classification (Fig. 2B), which suggested that the classification with two subgroups was more robust than others.

To understand the differences between the clusters, the clinical information in the dataset was used to explore the characteristics of each of the two clusters. The heat map shows the clustering in relation to age, International Neuroblastoma Staging System (INSS) stages and MYCN status, along with the expression of the genes used for clustering in 1811 patients (Fig. 2C). The genes shown in the heat map were the top 50 genes with the largest Median absolute deviation of gene expression. Further statistical analysis of the clinical information of the two clusters revealed that the age of Cluster 2 was smaller than that of Cluster 1 (*P* < 0.05). The detailed statistical results were shown in Table 1 below. Meanwhile, the status of MYCN of patients in Cluster 1 was mainly amplified, while the status of MYCN of patients in Cluster 2 was mainly non amplified (*P* < 0.05). INSS stage, which is closely related to prognosis, was also significantly different in Cluster 1 and Cluster 2 (*P* < 0.05). Alternative lengthening of telomeres (ALT) is regulated by break-induced replication. A Sankey diagram depicts the flow from the two cell cycle clusters to different status of telomerase reverse transcriptase (TERT) and ALT-associated promyelocytic leukemia bodies (APBs) in E-MTAB-8248 and GSE120559 datasets, in which the width of the flow rate is proportional to the number of patients (Fig. 2D). For status of telomerase reverse transcriptase, TERT rearrangements were more predominant in Cluster 1 (*P* < 0.05), while whether ALT-associated promyelocytic leukemia bodies were detected or not did not differ in the two clusters directly (*P* > 0.05). The bar chart showed the three chromosomal abnormalities closely associated with prognosis in the GSE73517 dataset, they were 1p deletion, 11q deletion, and 17q gain (Fig. 2E). As shown inside the statistical Table1, the respective proportion of 1p deletion and 17q gain to the total number of clusters differed in the two clusters (*P* < 0.05). However, the quantities of 11q deletion did not differ between the two clusters (*P* > 0.05). Using survival data from E-MTAB-8248 and GDC TARGET-NBL, the differences in prognosis between the two clusters were compared. The results showed that the prognostic status of Cluster 2 was better than that of Cluster 1, which was consistent with the distribution of clinical prognostic indicators between the two groups (Fig. 2F).

**Table 1:** Comparison of clinical characteristics between the two clusters.

Data source	Clinical information	Cluster1	Cluster 2	*P* value
ALL6 datasets	Age			*P* < 0.001
	< 18 months	381	619	
	≥ 18 months	490	321	
ALL6 datasets	MYCN status			*P* < 0.001
	Amplified	317	29	
	Non amplified	554	911	
ALL6 datasets	INSS stage			*P* < 0.001
	1	75	264	
	2	70	203	
	3	120	111	
	4	526	215	
	4s	80	147	
E-MTAB-8248 + GSE120559	TERT status			0.007
	Wild type	192	198	
	TERT rearrangement	28	11	
E-MTAB-8248 + GSE120559	APBs status			0.492
	Negative	182	178	
	Positive	38	31	
GSE73517	Chromosomes 1			*P* < 0.001
	1p deletion	34	10	
	Others	26	35	
GSE73517	Chromosomes 11			0.529
	11q deletion	18	11	
	Others	42	34	
GSE73517	Chromosomes 17			0.013
	17q gain	36	16	
	Others	24	29	

### Characterization of immunity in two clusters

**Figure 3:**
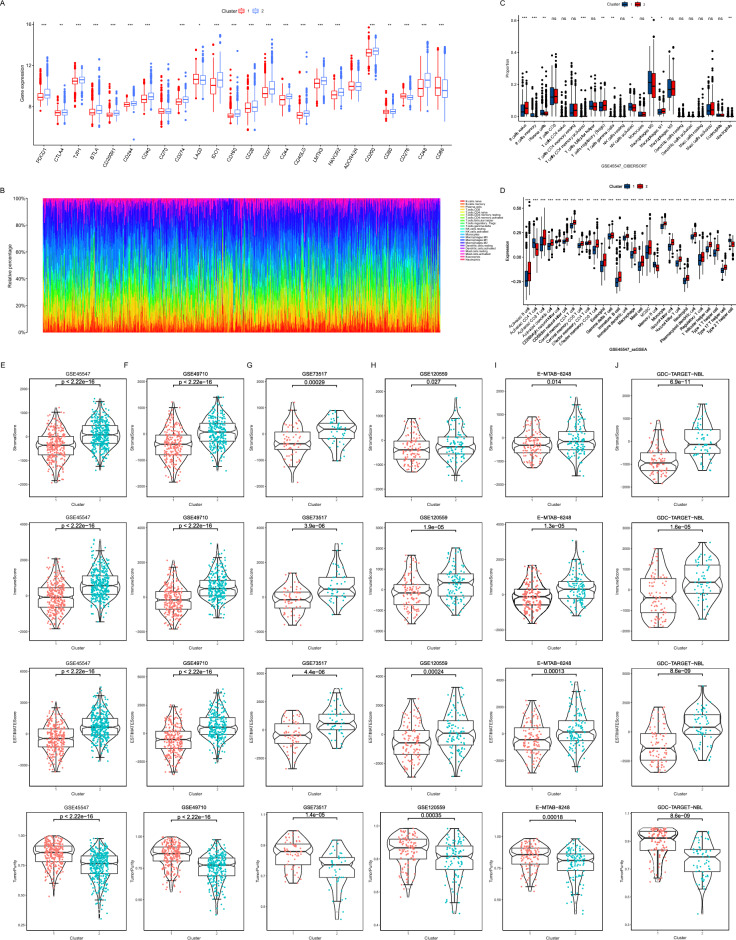
Comparison of immunization of two clustered subtypes. (**A**) Box plots showed the mRNA expression of immune checkpoints in two clusters (*P < 0.05; **P < 0.01; ***P < 0.001). (**B**) Stacked bar chart showed the percentage of immune cells in 1811 patients. (**C**) Box plots were used to display the distribution of the cell proportions calculated by the CIBERSORT algorithm of immune cells between the two clusters (*P < 0.05; **P < 0.01; ***P < 0.001). (**D**) Box plot of the distribution of immune cell expression between the two clusters as calculated by the ssGSEA algorithm (*P < 0.05; **P < 0.01; ***P < 0.001). (**E**–**J**) Box plots were created to visualize the distribution of the Stromal Score, Immune Score, ESTIMATES Score, and  Tumor Purity, which were calculated by the ESTIMATE algorithm between the two clusters in the GSE45547 (**E**), GSE49710 (**F**), GSE73517 (**G**), GSE120559 (**H**), E-MTAB-828 (**I**) and TARGET (**J**) datasets.

The immune microenvironment is closely related to tumors and the expression of immune checkpoints is a reflection of the immune response. Among the five microarray datasets integrated, 24 immune checkpoints were selected for comparison between clusters. As the results demonstrate, except for LAG3, CD276and CD86, the levels of immune checkpoints were higher in Cluster 2 than in Cluster 1 (Fig. 3A). Based on which CIBERSORT were used to estimate the immune infiltration and a bar chart was used to show the percentage of immune cells in each patient (Fig. 3B). To compare the variability of immunization between clusters in GSE45547, an analysis was conducted to compare the differences between the two clusters of immune cells according to the clustering grouping. The results indicate a significant variability in the immune cells of the two clusters (Fig. 3C). Further quantification of immune cells using ssGSEA shows that Cluster 2 has more immune cells overall than Cluster 1 (Fig. 3D). In the other five datasets, again using the CIBERSORT results and the ssGSEA results compared between the two clusters, Cluster 2 all showed more immune infiltration (Supplementary Fig. S3A–E).

The immune status of the patients was further assessed inside the six datasets using the ESTIMATE algorithm. The analysis results surface higher Tumor Purity in Cluster 1 than in Cluster 2 in the GSE45547 dataset (*P* < 0.05). Relatively, Stromal Score, Immune Score, and ESTIMATE Score in Cluster 1 were lower than in Cluster 2 (*P* < 0.05) (Fig. 3E). The same analysis was validated for the other five datasets (Fig. 3F–J). Combining the results of the previous analysis, we believe that Cluster 2 belongs to the class of rich immune status and Cluster 1 is the class of poor immune status.

### Identification of subgroup DEGs and functional annotation

**Figure 4:**
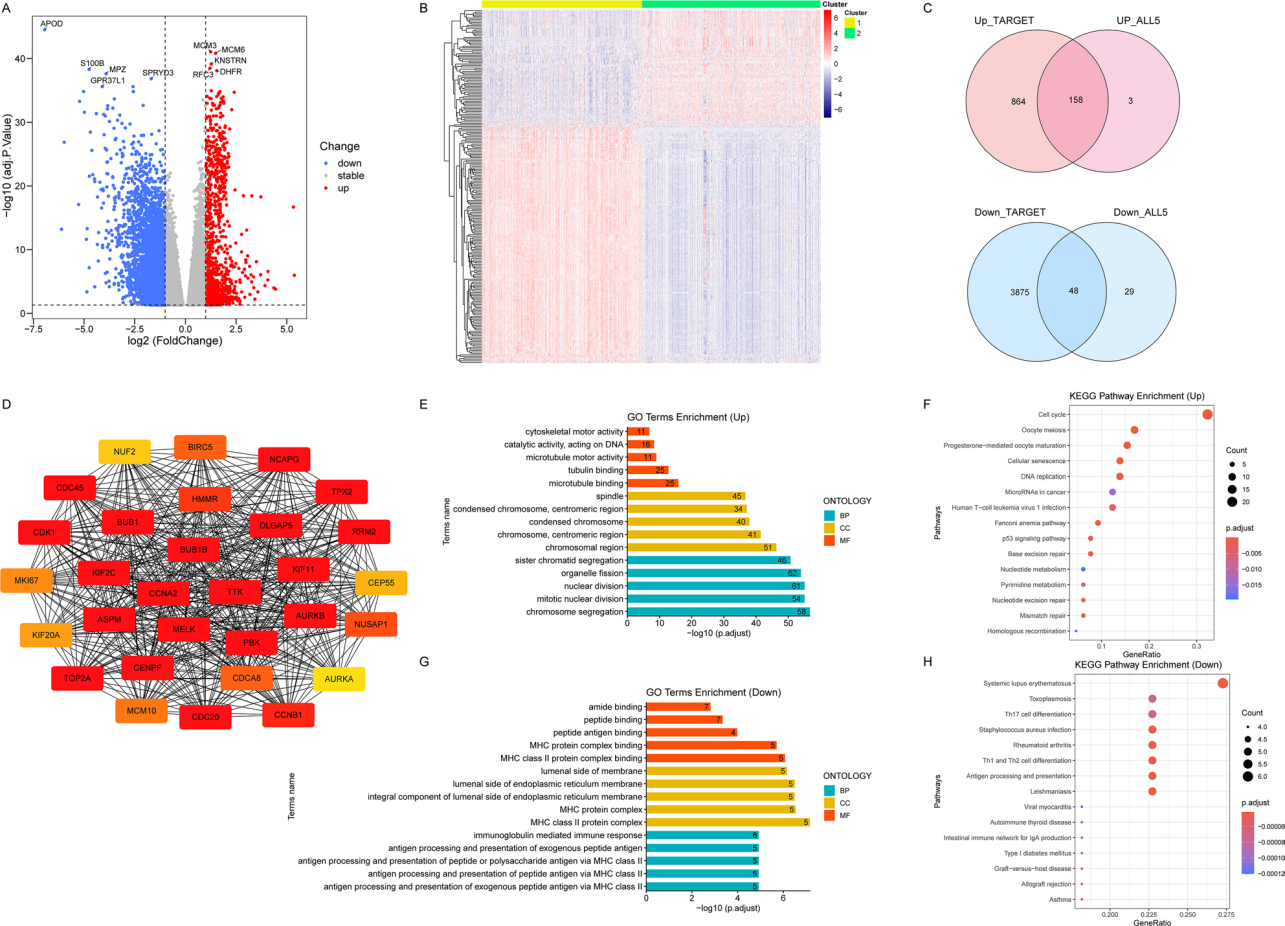
Identification DEGs and functional annotation of DEGs. (**A**) Volcano plot depicted the distribution of DEGs in TARGET dataset (Cluster1 VS Cluster2) and labeled the top 5 genes with the smallest ranking according to adjusted *P* value. (Genes with adjusted *P* value > 0.05 were not shown in the plot). (**B**) Heatmap of the DEGs derived from the 5 microarray datasets. (**C**) The Ven diagram showed the number of intersecting genes in the results of the difference analysis. (**D**) The TOP 30 genes based on the MCC algorithm, with the darker colors, indicating the higher MCC scores. (**E**,**F**) Bar graph (**E**) showed the results of GO enrichment and Bubble plots (**F**) showed KEGG enrichment results for Cluster 1 relative to Cluster 2 highly expressed genes. The numbers in the Bar graph represented the counts in the pathway. (**G**,**H**) Bar graph (**G**) showed the results of GO enrichment and Bubble plots (**H**) showed KEGG enrichment results for Cluster 1 relative to Cluster 2 low expressed genes. The numbers in the Bar graph represented the counts in the pathway. In the GO enrichment results (**E**,**G**), BP refers to Biological Process, CC denotes Cellular Component, and MF represents Molecular Function.

In order to investigate the key genes causing the differences between clusters in depth, a total of 4945 differential genes were obtained using “DESeq” package in the TARGET data between the two clusters, of which 1022 were highly expressed genes in Cluster 1 relative to Cluster 2 and 3923 were lowly expressed genes (Fig. 4A) (Supplementary Fig. S4A). The variance analysis of the five normalized datasets using the “limma” package yielded 238 variance genes. There were 161 up-regulated genes and 77 down-regulated genes (Cluster1 VS Cluster2) in the result (Fig. 4B). A total of 206 intersecting genes from the two difference analyses were designated as intergroup difference genes for Clusters 1 and 2 (Fig. 4C). We then constructed a Protein–Protein Interaction (PPI) network using the STRING database (Supplementary Fig. S4B). The TOP 30 genes based on the MCC algorithm were further demonstrated using the the cytoHubba plug-in in Cytoscape (Fig. 4D).

To gain insight into the function of the differential genes, enrichment analysis was performed. For the highly expressed genes (Cluster1 VS Cluster2) between the two clusters, GO enrichment results showed that these genes were closely associated with the cell cycle progression (Fig. 4E). The TOP 5 terms in Biological Process were chromosome segregation, mitotic nuclear division, nuclear division, organelle fission and sister chromatid segregation. The results of Cellular Component were mainly involved in chromosomal region; chromosome, centromeric region; condensed chromosome; condensed chromosome, centromeric region and spindle. Microtubule binding; tubulin binding; microtubule motor activity; catalytic activity (acting on DNA) and cytoskeletal motor activity were the TOP 5 terms in Molecular Function. KEGG analysis suggested that highly expressed genes (Cluster1 VS Cluster2) were mainly associated with Cell cycle, DNA replication, Oocyte meiosis and other pathways closely related to the cell cycle (Fig. 4F).

The enrichment results for low expressed genes showed an association with immunity. GO enrichment results mainly include antigen processing and presentation of exogenous peptide antigen via MHC class II and MHC class II protein complex binding (Fig. 4G). Similarly, the results of KEGG enrichment were closely related to immunity (Fig. 4H). Generally, the enrichment analyses showed that DEGs not only play an important role in the division of chromosome but are also associated with repair of DNA and immunity. At the same time, the enrichment results of each of the two group DEGs corresponded to the results of the previous clinical and immunological analyses.

### Identification of prognostic key genes and establishment risk score model

**Figure 5:**
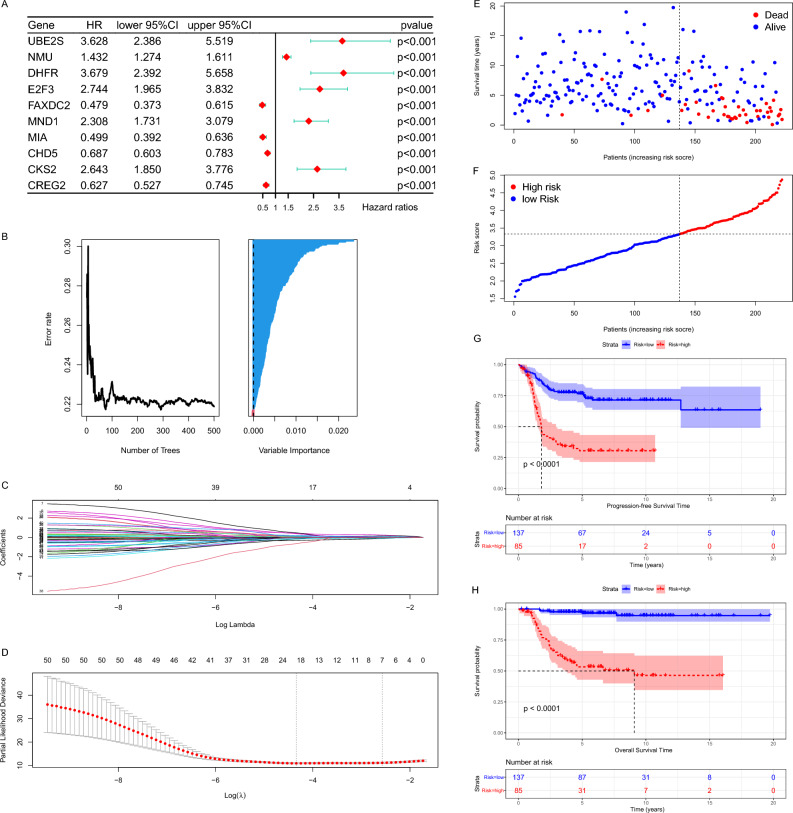
Construction of the risk model. (**A**) The forest plot showed the HR and 95% confidence interval of the most significant TOP 10 genes in the univariate regression results, sorted by *P* value. (**B**) The left graph showed the variation of Error rate with the number of trees. The right graph showed the ranking of genes according to the importance of the VIMP algorithm, where blue represents favorable to the correct judgment of the endings and red represents unfavorable. (**C**) Each line in the above graph represented a gene, the vertical coordinate was the value of the coefficient, the lower horizontal coordinate was log(λ), and the upper horizontal coordinate was the number of non-zero coefficients in the model at this time. (**D**) Based on cross-validation, for each value of λ, around the mean value of the target covariate shown in red, we can obtain a confidence interval for the target covariate. The two dashed lines indicate each of the two particular λ values. We chose lambda.1se as the final model parameter. (**E**) Each point in the scatter plot represented the survival status and survival time of a patient. The horizontal coordinates were the patients ranked from lowest to highest according to their risk scores. (**F**) Based on the risk score of each point in the scatter plot representing one patient, we divided them into high-risk and low-risk groups. (**G**,**H**) The Kaplan–Meier curves showed the progression-free survival time (**G**) and OS time (**H**) of the two risk groups of patients inside the E-MTAB-8248 dataset.

Based on the enrichment results, which imply that DEGs were strongly associated with chromosomal instability and disease heterogeneity in patients, we decided to search for key genes from within DEGs to construct a risk model. Firstly, we preliminarily screened out 177 OS-related genes with a filtering threshold of *P* value less than 0.01 by univariate Cox regression analysis in E-MTAB-8248 dataset (Supplementary Table 1) and displayed their top 10 significant genes by forest map (Fig. 5A). In the next step, “randomForestSRC” package were used to filter the key variables. As shown in the Fig. 5B, the oob error rate tends to stabilize when tree > 200, while the importance of the variables was judged using Variable Importance (VIMP) algorithm and the longer blue bars indicate the more important variables (Fig. 5B). We selected the TOP 50 most important genes based on the VIMP for inclusion in the LASSO Cox regression model (Supplementary Fig. S5A). With an optimal λ value (Fig. 5C,D), 7 genes (NMU, E2F3, UBE2S, DHFR, MIA, CHD5, and FAXDC2) retained their individual Cox coefficients after LASSO regularization (Supplementary Table 2). Using the established formula, the risk score was calculated for each sample (Fig. 5E). With a best cut-off value (Supplementary Fig. S5B), the dataset was divided into low-risk and high-risk groups (Fig. 5F). Kaplan–Meier analysis demonstrated that patients with higher risk score exhibited worse progression-free survival (PFS) and OS in the E-MTAB-8248 dataset (Fig. 5G,H).

### Validation of the risk score model

**Figure 6:**
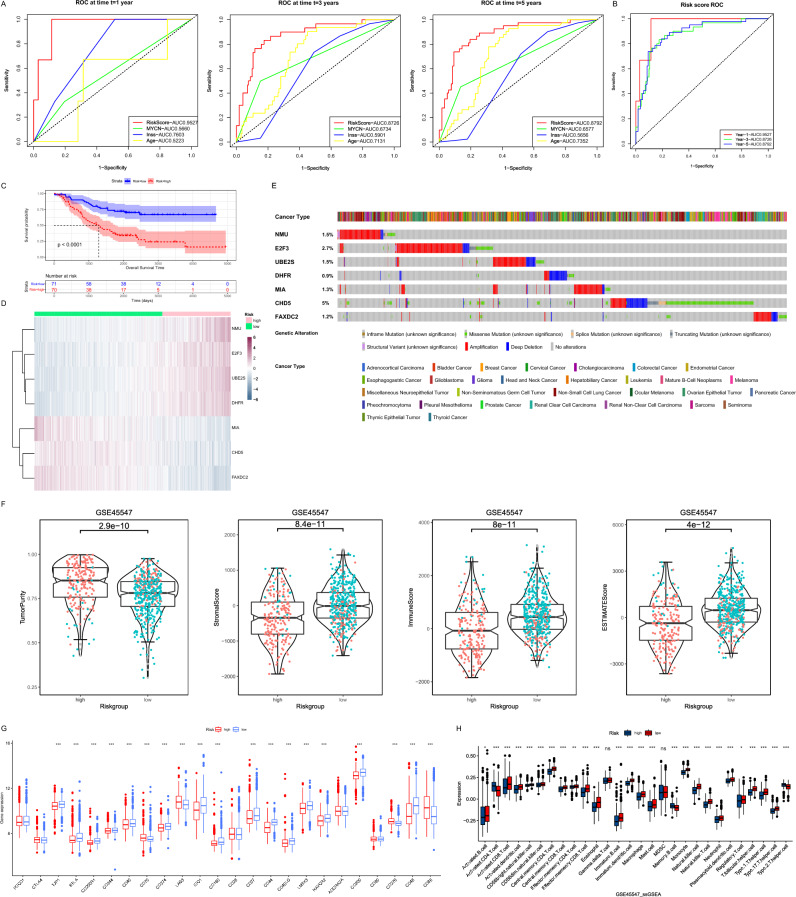
Validation and investigation of risk models. (**A**) The ability of clinical indicators and risk scores to determine prognosis at year 1, year 3, and year 5 were compared using ROC curves. (**B**) The ROC curve demonstrates the ability of the risk score to determine prognosis at year 1, year 3, and year 5. (**C**) The Kaplan–Meier curves showed the OS time of the two risk groups of patients inside the TARGET dataset. (**D**) The heat map demonstrated the expression levels of seven risk model genes in patients. (**E**) Mutations of 7 risk model genes in multiple tumors. (**F**) Comparison of differences in ESTIMATES results between high and low risk groups. Red dots indicated that patients belong to Cluster 1 and green dots indicated that patients belong to Cluster 2. (**G**) Box plots showed the mRNA expression of immune checkpoints in two risk groups (*P < 0.05; **P < 0.01; ***P < 0.001). (**H**) Box plot of the distribution of immune cell expression between the two risk groups as calculated by the ssGSEA algorithm (*P < 0.05; **P < 0.01; ***P < 0.001).

First, the receiver operating characteristic (ROC) curves of clinical indicators related to prognosis were compared inside the E-MTAB-8248 dataset, and the risk scores were all better than these indicators (Fig. 6A). In addition, ROC curve analysis indicated that the area under the curve (AUC) values of OS signature in 1-, 3-, and 5-year were 0.9527, 0.87266, and 0.8792, indicating that our prognosis signatures have favorable discrimination (Fig. 6B). Meanwhile, in the GDC TARGT-NBL dataset, risk scores were also strongly correlated with OS (Fig. 6C). In addition, we analyzed and mapped the expression profiles of seven genes in different risk subgroups of 1670 patients, and significant expression differences could be seen (Fig. 6D). We further compared the distribution of the seven gene expressions in a variety of tumors. UBE2S was found to be highly expressed in most tumor tissues, while MIA was mainly concentrated in tumor tissues of melanoma (SKCM) (Supplementary Fig. S6A). The mutations of the seven genes were further explored in a variety of tumors, and it could be found that the highest mutation rate was CHD5, followed by E2F3. And the types of mutations were mainly concentrated in Amplification, Deep Deletion and Missense Mutation (Fig. 6E).

The results of the previous ESTIMATE algorithm were used to compare groups based on high and low risk. The analysis results surface higher Tumor Purity in high-risk group than in low-risk group in the GSE45547 dataset (*P* < 0.05). Relatively, Stromal Score, Immune Score and ESTIMATE Score in high-risk group were lower than in low-risk group (*P* < 0.05) (Fig. 6F). In GSE49710, GSE73517, GSE120559 and E-MTAB-8248, Tumor Purity, Immune Score and ESTIMATE Score had the same variation in the high-risk versus low-risk groups (Supplementary Fig. S6B–E). We further compared the expression of immune checkpoints between high and low risk groups (Fig. 6G). Combined with the results of ssGSEA it can be concluded that the low-risk group had a better immune status (Fig. 6H).

### Compare differences between high and low risk groups

**Figure 7:**
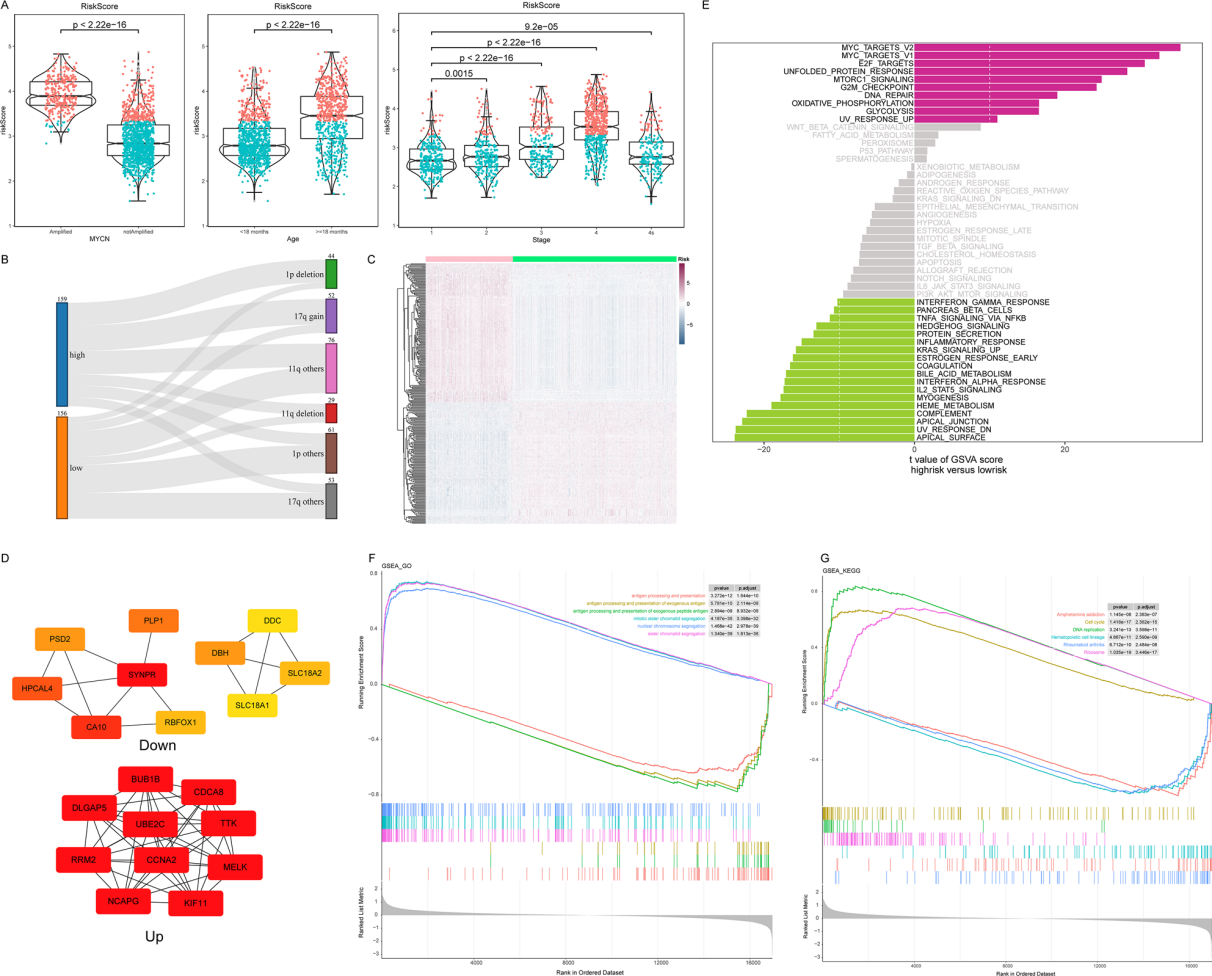
Comparison between high and low risk groups. (**A**) Comparison of risk scores in MYCN status, age groups and INSS stages. (**B**) The Sankey diagram showed the distribution of chromosomal abnormalities in the two risk groups. (**C**) The heat map showed the levels of differential genes between the high and low risk groups. (**D**) TOP 10 hub genes identified by MCC algorithm. (**E**) The bar graph showed the results of GSVA enrichment. Purple represented the major pathways enriched to in the high-risk group and green represented the major pathways in the low-risk group. (**F**) The TOP 3 most significant GO enriched terms in the high-risk and low-risk groups. (**G**) The TOP 3 most significant KEGG enriched pathways in the high-risk and low-risk groups.

The distribution of risk scores inside MYCN status, age and INSS stages was further explored in 1670 patients from all 5 microarray datasets. The results revealed that patients with MYCN amplification status, age ≥18 months and progressive worsening of INSS staging all had higher risk scores (Fig. 7A). Patients in E-MTAB-8248, GSE73517 and GSE120559 were divided into two groups, high-risk and low-risk, based on the optimal cut-off values. As shown in the Table 2, TERT rearrangements were more common in the high-risk group (*P* < 0.05). However, the positive of ALT-associated promyelocytic leukemia bodies was not statistically different between the two groups (*P* > 0.05). After that, the relationship between risk grouping and chromosomal instability was further explored. The results of the analysis confirmed that 1p deletion and 17q gain differed in the high- and low-risk subgroups and that the high-risk group was more likely to have these aberrations (*P* < 0.05). In contrast, 11q deletion was not statistically different between the two groups (*P* < 0.05) (Fig. 7B).

**Table 2:** Comparison of clinical characteristics between the two risk groups.

Data source	Clinical Information	High-risk	Low-risk	*P* value
E-MTAB-8248 + GSE120559	TERT status			*P* < 0.001
	Wild type	130	260	
	TERT rearrangement	30	9	
E-MTAB-8248 + GSE120559	APBs status			0.310
	Negative	138	222	
	Positive	22	47	
GSE73517	Chromosomes 1			*P* < 0.001
	1p deletion	35	9	
	Others	18	43	
GSE73517	Chromosomes 11			0.302
	11q deletion	17	12	
	Others	36	40	
GSE73517	Chromosomes 17			*P* < 0.001
	17q gain	39	13	
	Others	14	39	

We further delved into the closely related mechanisms of clinical and risk grouping through analysis of variance. Patients with 1670 microarray data were further divided into high and low risk groups based on the risk model for difference analysis. A total of 314 differential genes were obtained, including 146 down-regulated genes and 168 up-regulated genes (high-risk VS low-risk). As shown in the heatmap, the difference genes were able to distinguish well between high and low risk groups (Fig. 7C). The volcano plot showed the top five genes in the differential genes ranked according to their adjusted *P* value (Supplementary Fig. S7A). We then constructed a PPI network using the STRING database (Supplementary Fig. S7B,C). The TOP 10 genes based on the MCC algorithm were further demonstrated in down-regulated genes and up-regulated genes (Fig. 7D).

Enrichment analysis of differential genes in the Hallmark database using the GSVA algorithm revealed significant differences between the two groups in numerous pathways (Fig. 7E). In the high-risk group, the significant pathways were MYC Targets_V2, MYC Targets_V1, E2F Targets, Unfolded protein response, Mtorc1 signaling, G2M chickpoint and DNA repair. In the low-risk group, Apical surface, UV response_DN, Apical junction, Complement, HEME metabolism and Myogenesis were the significant pathways. Further analysis using GSEA enrichment method, we could find that the pathways of GO in the high-risk group were mitotic sister chromatid segregation, nuclear chromosome segregation and sister chromatid segregation. The TOP 3 terms of low-risk group were antigen processing and presentation, antigen processing and presentation of exogenous antigen and antigen processing and presentation of exogenous peptide antigen (Fig. 7F). The KEGG results, on the other hand, showed that the high-risk group was mainly closely associated with the three pathways of the Cell cycle, DNA replication and Ribosome (Fig. 7G). The pathways in the low-risk group were focused on immune-related pathways such as Amphetamine addiction, Hematopoietic cell lineage and Rheumatoid arthritis. Based on the enrichment results, the worse prognosis in the high-risk group may be related to this.

### Construction of neural network and integrated prognostic models to guide treatment

**Figure 8:**
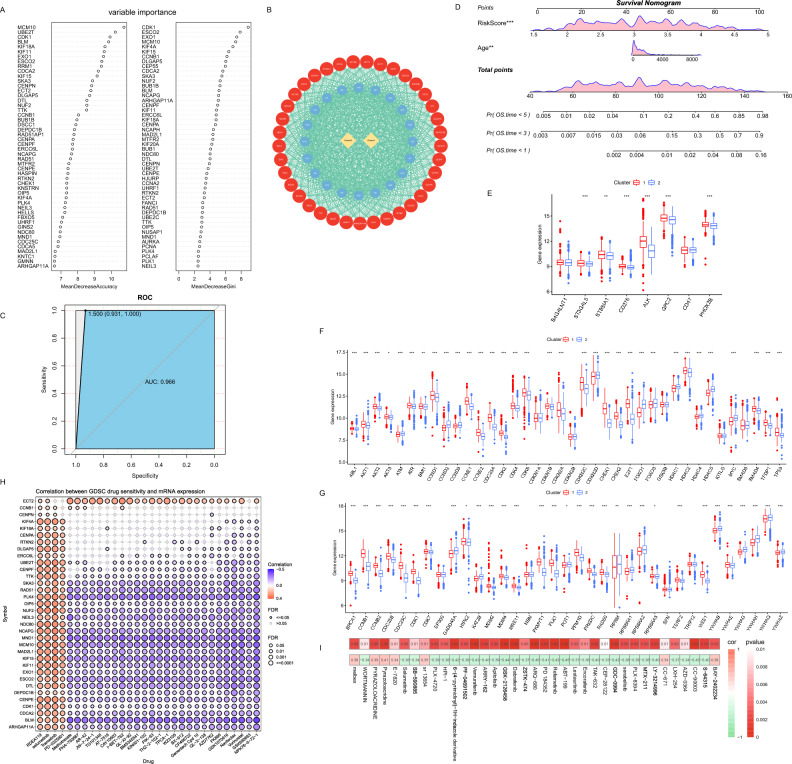
Neural Networks and Treatment Analysis. (**A**) Importance ranking chart of variables based on MDA and MDG. (**B**) Neural network structure schematic. The outer red layer represents the input layer, the middle blue represents the 20 hidden neurons, and the output layer is yellow. (**C**) ROC curves demonstrate the classification performance of the neural network in the GSE49710 dataset. (**D**) Assessment of patient survival probability using nomograms. (**E**) Immunotherapy target gene expression levels. (**F**) G1/S cell cycle checkpoint gene expression levels. (**G**) G2/M cell cycle checkpoint gene expression levels. (**H**) GDSC database drug sensitivity analysis results. (**I**) Heat map of correlation between risk score and drug sensitivity.

The random forest algorithm was employed to select the neural network genes. We used both the Mean Decrease Accuracy (MDA) and the Mean Decrease Gini (MDG) to obtain the top 50 most important genes, and took the intersection of the two as the final key genes. Through the graph of rate, error versus number of trees, we chose mtry = 6, ntree = 1200 as the final parameter of the model (Supplementary Fig. 8A,B). In our final fitted model, the out-of-bag (OOB) value was 2.95%. As shown in Fig. 8A, 37 genes were finally identified for the construction of neural network models for neuroblastoma patients. By experiment, the number of hidden layers was 1, with a total of 20 hidden neurons, and learningrate = 0.1 as the final setting of the model (Fig. 8B). Meanwhile, the Activation Function we chose was “tanh”. We completed the training using 643 patients from the GSE45547 dataset and performed external validation in 493 patients from GSE49710 with good results (AUC = 0.966) (Fig. 8C).

We further explored whether these two indicators are related to survival. The clustering grouping and risk score were included in the univariate Cox regression analysis, which revealed that both the Cluster 1, and the higher risk score were risk factors affecting prognosis (Table 3). Clinical indicators such as age, whether MYCN was amplified, and INSS staging were further included for multifactorial regression analysis and the results revealed that only the risk score and age were an independent risk factor for prognosis (Supplementary Fig. 8C). To facilitate the assessment of prognosis, nomograms were constructed by age and risk score. The probability of survival at 1, 3, and 5 years were predicted by calculating the number of points (Fig. 8D).

**Table 3:** Result of the univariate Cox regression analysis.

Variables	HR	z	*P* value	95% confidence interval	
Cluster	0.153	− 4.532	5.85e−06	0.068	0.344
Risk score	6.876	7.555	4.20e−14	4.170	11.339

We further evaluated the significance of clustering and risk score to guide treatment. Immunotherapy as a treatment modality with great potential, we compared the distribution of potentially used targets in immunotherapy between the two clustered subgroups. We could find different expression levels of immunotherapy targets in Cluster 1 and Cluster 2, suggesting that different clusters using different immunotherapy may be more prognostic (Fig. 8E). Cell cycle targeted therapy is also an important modality of treatment. It was interesting to note that the cell cycle checkpoints were significantly different between the two groups (Fig. 8F,G). The expressions of CDK2, CDK4 and CDK6 were higher in Cluster 1 than in Cluster 2, while the expressions of ATM were higher in Cluster 2 than in Cluster 1. We also analyzed the correlation with the IC50 of oncology drug in the GDSC database by GSCA using the genes that used for neural network (Fig. 8H). Based on the results of the analysis, we believe that Cluster 2 may be more appropriate for the four drugs RDEA119, Selumetinib, Trametinib and PD-0325901. Data from the CellMiner database of NCI-60 cell lines were downloaded and sensitivity analyses were performed between risk scores and drugs that had undergone Clinical trials and FDA approved. We set *P* < 0.01 as our filtering index and showed the results with a heatmap (Fig. 8I). Based on the results of the analysis, it could be found that most of the drugs were negatively correlated with risk scores.

## Discussion

As a highly heterogeneous solid tumor, individualized treatment of neuroblastoma to improve its prognosis is a problem at this stage. Currently, neuroblastoma is mainly based on the INRG risk stratification system to guide the treatment of different patients^22^. Despite this, the 5-year EFS for children with metastatic neuroblastoma and aged 18 months or older is only close to 50%^23^. As cell cycle-targeted inhibitors are being studied and cell cycle-related mechanisms are gaining ground in neuroblastoma patients, the use of cell cycle-related genes is important for identifying molecular subtypes and finding therapeutic targets or prognostic biomarkers in neuroblastoma patients.

We first demonstrated the feasibility of typing patients according to their genes by downscaling 643 samples using the tSNE algorithm based on gene expression levels. Considering the promising application of immunotherapy, in our study, we initially identified 924 immune-related cell cycle genes using the WGCNA algorithm. These genes were negatively correlated with ESTIMATE Score and positively correlated with Tumor Purity. Based on the above genes, we classified the 1811 patients into two clusters with distinct differences.

In terms of clinical information, the two clusters have their own significant characteristics. Overall, the clinical indicators in Cluster 1 were all more inclined toward an unfavorable prognosis relative to Cluster 2. The percentage of patients in Cluster 2 with an age < 18 months was much higher than in Cluster 1 (*P* < 0.05). For the distribution of MYCN status in the two groups, Cluster 1 can be considered as MYCN amplified group and Cluster 2 as MYCN non-amplified group. Although 29 out of 940 patients in Cluster 2 were MYCN amplified status, which indirectly illustrates the limitation of using a single biological indicator classification in clinical situations. For the commonly used INSS staging, stage 4 accounted for 60% of the total in Cluster 1, while in Cluster 2 this proportion was only about 22%. The study showed that the older the child was diagnosed (18 months as cut-off value), the amplified MYCN status and INSS stage was stage 4, all three of which were markers of unfavorable prognosis^22,24^. Cluster 1 also showed more chromosomal instability, as demonstrated by the fact that patients with 1p-deletion and 11q-deletion were more concentrated in Cluster 1 (*P* < 0.05). The TERT rearrangements phenomenon was likewise more common in Cluster 1 (*P* < 0.05). However, there were no difference in the distribution of 17q-gain and ALT-associated promyelocytic leukemia bodies in the two clusters (*P* > 0.05). The Kaplan–Meier curves plotted by the survival analysis also corroborated that the OS time of Cluster 2 was better than that of Cluster 1. We further explored the immune infiltration between the two clusters. We performed immune evaluation inside each of the six datasets using the three algorithms CIBERSORT, ssGSEA and ESTIMATE. Combining the results, we can assume that the Cluster 2 have a better immune status. This may also partially explain why Cluster 2 has a better prognosis.

Immune checkpoints as a basis for immunotherapy, we evaluated the expression of 24 immune checkpoints between two clusters. We found that LAG3, CD276 and CD86 were highly expressed in Cluster 1, while most of the immune checkpoints were highly expressed in Cluster 2. This was inseparable from the characteristics of the disease. MYCN amplification correlated to a higher number of LAG3 + type 1 regulatory (Tr1) cells in peripheral blood^25^. CD276(B7-H3) is highly expressed in tumors and restricted expression in normal tissues which is a potential therapeutic target^26^. In the experiment, Chimeric antigen receptor T cells against CD276 were able to overcome the heterogeneity of neuroblastoma^27^. The high expression of CD86 may be associated with higher tumor purity in Cluster 1. Research shows that CD86 induced a T-cell immune response in neuroblastoma in vitro and served as an effective tumor vaccine in the tumor prevention model^28^.

The results of the enrichment analysis of differential genes between the two clusters further revealed the differences in pathway mechanisms between the two clusters. The highly expressed genes in Cluster 1 were concentrated in cell cycle-related pathways involving chromosome segregation, microtubule binding and chromosomal region. The analysis of the previous clinical information also showed that Cluster 1 exhibited more chromosomal instability. Similarly, Cluster 2 has a better immune status as evidenced in the enrichment results. Evidence suggests that tumor-specific MHC-II is associated with a good prognosis for cancer patients, including those treated with immunotherapy^29^.

In order to better assess the individual situation of each patient, we tried to construct a risk model using DEGs between the two clusters. Further analysis showed that it had better predictive power than traditional biomarkers. We first obtained 177 genes from DEGs that were closely associated with survival using Cox model screening (*P* < 0.01). Random Survival Forest (RSF), a machine learning survival algorithm, has many applications in biomedicine^30,31^. In this study, VIMP values of each gene that calculated by RSF was used to further screen for genes closely related to survival. The 50 genes with the largest VIMP values were included in the Lasso Cox regression model finally 7 genes (NMU, E2F3, UBE2S, DHFR, MIA, CHD5, FAXDC2) were obtained for the construction of the model. Among these, NMU, E2F3, UBE2S and DHFR belong to Cluster 1 relative to Cluster 2 of highly expressed genes. While MIA, CHD5 and FAXDC2 were low expression genes.

Neuromedin U (NMU) derives its name from its powerful contraction effect on the muscles of the rat uterus^32^. Although neurons regulate type 2 congenital lymphocytes via neuromedin U^33^, high NMU expression is associated with poor prognosis of cancer^34,35^. E2F Transcription Factor 3 (E2F3) interacts with retinoblastoma protein directly to regulate the expression of genes participating in the cell cycle. Harold I Saavedra et al. found that E2F3 overexpression causes centrosome amplification and uncontrolled mitosis in several studies, which can promote chromosomal instability leading to tumors^36,37^. Ubiquitin Conjugating Enzyme E2 S (UBE2S) has been shown to promote ovarian cancer development by promoting the PI3K/AKT/mTOR signaling pathway to regulate cell cycle^38^. Meanwhile, UBE2S can work with TRIM28 in the nucleus to accelerate the cell cycle through ubiquitination of p27 to develop hepatocellular carcinoma^39^. In recent years, Dihydrofolate Reductase (DHFR), a key enzyme in one-carbon metabolism, has been well recognized as a target for cancer therapy^40,41^. A positive coefficient for the four genes mentioned above in the risk model means that the higher the level of gene expression, the more at risk the patient is.

MIA may promote the separation of cells from the extracellular matrix^42^. Chromodomain Helicase DNA Binding Protein 5 (CHD5) has demonstrated its unique role as a novel tumor suppressor in a variety of cancers^43–45^. Fatty acid hydroxylase domain containing 2 (FAXDC2), a member of the fatty acid hydroxylase superfamily, is a neo gene that enhances megakaryocyte maturation, suggesting that it may have a potential value as a therapy for differentiation^46^. Taken together, the seven risk model genes include both those that have been intensively studied and those that lack research, suggesting the potential broad research value of risk model genes in neuroblastoma. At the same time, ROC curve analysis indicated that the AUC values of OS signature in 1-, 3-, and 5-year were 0.9527, 0.87266, and 0.8792, indicating that our prognosis signatures have favorable discrimination. Moreover, the risk model showed better predictiveness compared to other single clinical biological indicators.

A between-group analysis of the two groups grouped based on risk scores revealed distinct differences in immune levels and clinical information between the two groups. We could find that LAG3, CD276 and CD86 were highly expressed in the high-risk group. Combining multiple immunization algorithms, we could assume that the low-risk group has a better immune status. Results with clinical analysis showed that patients with MYCN amplification status, age ≥ 18 months and progressive worsening of INSS staging all had higher risk scores which demonstrated the consistency of the risk model with the clinical. The GSEA enrichment results in the high-risk group showed a strong correlation with the MYC pathway. MYC genes are a class of nucleoprotein oncogenes, and as a broadly acting transcription factor, MYC regulates cell differentiation and proliferation through a variety of mechanisms, including the transcriptional amplification of target genes^47,48^. In addition, the high-risk group is closely associated with signaling pathways such as cell cycle and chromosome segregation. Abnormalities in these pathways may drive patients toward a poor prognosis. In contrast the low-risk group showed a strong correlation with immunity, which together with the results of the immune analysis corroborated the better immune status of the low-risk group.

Two molecular subtypes of neuroblastoma successfully classified patients, and a risk model based on the analysis of differences between subtypes better quantitatively assessed the survival status of patients. At this stage, neural network models have become a powerful tool for machine learning. To better apply the results of the study in the clinic, we used the results of inter-cluster variance analysis to construct a neural network classifier applicable to neuroblastoma patients. A neural network based on 37 genes built in 643 patients was well validated in the classification of 493 patients (AUC = 0.966).

The goal of molecular subtypes and risk models is to help patients develop individualized treatment plans and improve prognosis. This study provides the results of sensitivity analyses for multiple drug data. Cell cycle-targeted therapy serves as a promising therapeutic tool^49^ . With the clinical success of CDK4/6 inhibitors, targeting individual cell cycle components may become an effective anti-cancer strategy^50^. The distribution of cell cycle checkpoints between the two clusters had their own significant characteristics. For Cluster 1, with higher expression levels of CDK4, CDK6 and PLK1, we can take the treatment by applying cell cycle brakes. Drugs in this segment include palbociclib, ribociclib and abemaciclib, which target CD4/6^51^, and BI 2536^52^ and GSK461364^53^ which target PLK1. In contrast, ATM was highly expressed in Cluster 2. Patients may be treated through M3541 and AZD0156 by accelerating the cell cycle^15^.

Among these, immunotherapy has great potential to fight against cancer, and immunotherapy for neuroblastoma is gradually being studied in depth. GD2 is the most common target antigen for neuroblastoma immunotherapy^54,55^. Although B4GALNT1, the enzyme that catalyzes the final step of GD2 synthesis, did not differ between the two clusters, ST3GAL5 and ST8SIA1, genes more upstream in the synthesis pathway, were more highly expressed in Cluster 1 than in Cluster 2. It has been shown that downregulation of ST8SIA1 promotes the loss of GD2, leading to a bottleneck in the synthesis and expression of GD2, which results in the failure of anti-GD2 antibodies^56^. The results of studies on B7-H3 (CD276), ALK, GPC2, and PHOX2B as novel immunotherapeutic targets show great promise for the treatment of neuroblastoma^57,58^. In this study, a comparison of the expression of these targets revealed higher expression in Cluster 1.

The heterogeneity of neuroblastoma is manifested in several ways, and we hope to be able to classify different patient categories and assess the risk profile of patients at the genetic level. Based on the results of the study, a rational individualized treatment plan is further assigned to the patient. Individualized treatment is beneficial to the patient's prognosis, while making the best use of medical resources and reducing the financial burden on the patient. Although our molecular subtype and risk models performed well in the assessment of clinical performance, immune status and survival prognosis, certain limitations should be noted in this study. All of our results were obtained by analyzing patient information and gene expression profiles in public databases, which may be influenced by the data leading to biased results. However, we compensated for this shortcoming by collecting as many patients as possible.

## Conclusions

We have developed a neural network model to classify neuroblastoma patients and a risk model to assess the prognostic status of patients. The intergroup mechanistic differences revealed in the study are more beneficial to our understanding of neuroblastoma. At the same time, the molecular subtypes and risk model will be used to help clinicians choose the best treatment strategy. The 37 subtype classification genes and 7 risk model genes obtained in this study provide new ideas for further experiments.

## Materials and methods

### Data acquisition and preprocessing

The set of genes of cell cycle-related signaling pathways in GO and KEGG and pathways were downloaded through the Molecular Signatures Database^59^ (https://www.gsea-msigdb.org/gsea/msigdb/index.jsp) and collated to obtain 1865 cell cycle-related genes for further studies. Common immune checkpoint and cell cycle checkpoint names were collected through literature reading and translated to match the gene names in the expression matrix. Data on the expression levels of target genes in multiple cancers were obtained from the Gene Expression Profiling Interactive Analysis platform^60^ (GEPIA, http://gepia.cancer-pku.cn/). Exploring and visualizing mutations in target genes from multidimensional cancer by The cBioPortal for Cancer Genomics^61^ (http://www.cbioportal.org).

A systematic search of publicly available transcriptomic data with clinical annotation for neuroblastoma was performed. In total, five microarray datasets with clinical information and one RNA-sequencing (RNA-seq) datasets named TARGET-NBL which was downloaded from Genomic Data Commons (https://gdc.cancer.gov/) was included in our study. Microarray gene expression data that contained GSE45547^62^, GSE49710, GSE73517^63^ and GSE120559^64^ were downloaded from Gene Expression Omnibus (GEO, https://www.ncbi.nlm.nih.gov/geo/) and E-MTAB-8248^65^ was download from ArrayExpress (https://www.ebi.ac.uk/biostudies/arrayexpress). For the downloaded microarray data were normalized. For TARGET dataset, the FPKM value of gene expression and the counts value were both downloaded. In all datasets, patients without MYCN status were removed. We used the t-distributed stochastic neighbor embedding (t-SNE) algorithm to downscale the multidimensional expression data of patients for the observation of tumor heterogeneity. For subsequent integration of the dataset, we adopted the ComBat method in R language with “sva” package^66^ (version 3.44.0) to remove the batch effect between the datasets. The principal component analysis was used to evaluate whether the batch effect was removed.

### Identification of immune-related cell cycle genes (IRCCGs)

Included in the analysis were the cell cycle-related genes obtained from the previous collation. Weighted gene co-expression network analysis (WGCNA)^67^ was performed using the “WGCNA” package (version 1.71) to construct a scale-free co-expression network and identify a gene module that was mostly associated with ESTIMATE results. The genes in that module were identified as Immune-related cell cycle genes (IRCCGs).

### Consensus clustering

We used the “ConsensusClusterPlus” package^68^ (version 1.60.0) in R and the clustering was selected on the basis of the identified Immune-related cell cycle genes. The maximum cluster number was set to be 5. The final cluster number was determined by the consensus matrix and the cluster consensus score (> 0.8). The higher cluster consensus scores indicate more robust clustering.

### Immune infiltration analysis

The tumor purity of samples, StromalScore, ImmuneScore, and ESTIMATEScore were estimated using R package “estimate”^69^ (version 1.0.13). CIBERSORT^70^ was used to quantify the relative abundance of 22 immune cell species in the sample. The Single-sample gene set enrichment analysis (ssGSEA) algorithm was employed to quantify the abundance of 28 immune cell types in different samples.

### Differentially expressed gene analysis between clusters or risk groups

Patients were divided into different groups according to the result of cluster analysis or the result of risk score. DEGs of microarray datasets were explored between two groups using the “limma” package^71^ (version 3.52.2). DEGs of sequencing datasets were explored between two groups using the “DESeq2” R package (version 1.36.0). The DEG cut-off was set as |log2 (Fold Change) |> 1 and adjusted *P* value < 0.05. The visualization of the variance analysis results was in the form of volcano plots and heatmaps.

### Enrichment analysis and protein–protein interaction network of the differentially expressed genes

DEG functional enrichment analysis, including Gene Ontology (GO)^72^ and Kyoto Encyclopedia of Genes and Genomes (KEGG)^73^ analysis, was carried out using the “clusterProfiler” R package^74^ (version 4.4.4). Adjusted *P* value < 0.05 was considered statistically significant. The R package “GSVA” (version 1.44.2) was used to perform enrichment analysis in the Hallmark database, and the cutoff value was set to 10. For the GSEA enrichment results, we set the screening metrics as |Normalized Enrichment Score (NES)|> 1, NOM *P* value < 0.05 and FDR (adjusted *P* value) < 0.05.

The PPI network was performed automatically by Search Tool for the Retrieval of Interacting Genes/Proteins (version 11.5; https://string-db.org/). Cytoscape software (version 3.9.1) was used for visualization. Moreover, CytoHubba plug-in was used to identify significant genes in this network as hub genes. We used Maximal Clique Centrality (MCC) algorithms to calculate the top 30 hub genes.

### Establishment of the prognostic risk score

Firstly, we performed single factor analysis by proportional hazards model in the E-MTAB-8248 dataset using the results of inter-cluster analysis of variance. Analysis was achieved through “survival” R packages (version 3.3.1) and genes with *P* < 0.01 were screened as prognosis-related genes. Next, we used the random survival forest (RSF) model from the “randomForestSRC” R package (version 3.1.1) to further filter candidate genes that were closely related to survival. The algorithm ranked each gene according to importance, and we selected the 50 most important genes to be included in the subsequent analysis. By “glmnet” R package (version 4.1-4), these 50 genes were used as the input of the least absolute shrinkage and selection operator (LASSO) Cox regression model and ultimately to screen out the significant genes. We finally obtained 7 genes for the construction of the risk score model in our analysis. Based on the expression values of the corresponding genes of the patients and the Cox coefficients, we can calculate the risk score for each patient according to the algorithm of the inner product of matrices. The calculation was publicly announced as follows:

$$risk\, score={\sum }_{n=1}^{7}Coefficient({gene}_{n})\times Expression({gene}_{n})$$

### Construction of neural network for clusters

Random forest algorithm from the “randomForest” R package (version 4.7-1.1) was applied to screen for the most important candidate genes correlated with different clusters in GSE45547 dataset. Based on the results of ranking the importance of genes, we selected the intersecting genes in the top 50 genes of both the Mean Decrease Accuracy (MDA) and the Mean Decrease Gini (MDG) as the input genes for constructing the neural network. The R package “neuralnet” (version: 1.44.2) has been used to develop a deep learning model of the candidate genes after the expression values of genes were standardized to the maximum and lowest values. We set a hidden layers and 20 hidden neurons in the GSE45547 dataset to train the model. For our constructed neural network model in GSE49710 for external validation.

### Drug sensitivity analysis

The analysis of the correlation between gene sets and drugs was obtained from an analysis with the online site GSCA^75^ (http://bioinfo.life.hust.edu.cn/GSCA/#/). This analysis platform integrates data on drug sensitivity and gene expression from the GDSC database. CellMiner (https://discover.nci.nih.gov/cellminer/home.do) is a database designed for the cancer research community to facilitate integration and study of molecular and pharmacological data for the NCI-60 cancerous cell lines. We downloaded data about the NCI-60 drug trials and screened it for inclusion in our study for drugs that had undergone Clinical trials and FDA approved. After collation of the data, we used correlation analysis to assess the relationship between risk scores and drug sensitivity.

### Survival analyses and nomogram construction

The Kaplan–Meier method was used to draw survival curves by “survminer” package (version 0.4.9). Single factor analysis by proportional hazards model was used to identify prognostic factors. Multi factor analysis by proportional hazards model was used to identify independent prognostic factors. A prognostic nomogram including all independent prognostic factors was constructed to predict the OS of neuroblastoma patients by “rms” package (version 6.3-0).

### Statistical analysis

All data processing and analysis were performed in R software (version 4.2.1) by RStudio. In order to compare two groups of continuous variables, we used independent Student's t-tests to calculate the statistical significance, and differences between non-normally distributed variables were calculated using the Wilcoxon rank sum test. We used the chi-square test or Fisher's exact test to analyse the statistical significance between the two sets of categorical variables. All statistical *P* values were two-sided, and *P* < 0.05 was considered statistically significant.

### Ethics approval and inform consent

The study was based on open-source data from multiple databases. Ethical approval has been provided for the patients involved in these databases. Therefore, there are no ethical issues with this article.

## Supplementary Information

Supplementary Information 1.

Supplementary Information 2.

Supplementary Information 3.

## Data Availability

The genetic and clinical data used in this study are available in the GEO (GSE45547: https://www.ncbi.nlm.nih.gov/geo/query/acc.cgi?acc=GSE45547; GSE49710: https://www.ncbi.nlm.nih.gov/geo/query/acc.cgi?acc=GSE49710; GSE73517: https://www.ncbi.nlm.nih.gov/geo/query/acc.cgi?acc=GSE73517; GSE120559: https://www.ncbi.nlm.nih.gov/geo/query/acc.cgi?acc=GSE120559), GDC (https://xenabrowser.net/datapages/?cohort=GDC%20TARGET-NBL&removeHub=https%3A%2F%2Fxena.treehouse.gi.ucsc.edu%3A443) and ArrayExpress (https://www.ebi.ac.uk/biostudies/arrayexpress/studies/E-MTAB-8248?query=E-MTAB-8248) databases. Cell cycle-related genes were obtained from the MSigDB (KEGG: https://www.gsea-msigdb.org/gsea/msigdb/human/geneset/KEGG_CELL_CYCLE.html; GOBP: https://www.gsea-msigdb.org/gsea/msigdb/human/geneset/GOBP_CELL_CYCLE.html). Data on the expression levels of 7 risk model genes in multiple cancers were obtained from the GEPIA (http://gepia.cancer-pku.cn/detail.php?clicktag=matrix) platform. Mutations in 7 risk model genes from multidimensional cancer by the cBioPortal for Cancer Genomics (https://www.cbioportal.org/results/oncoprint?cancer_study_list=laml_tcga_pan_can_atlas_2018%2Cacc_tcga_pan_can_atlas_2018%2Cblca_tcga_pan_can_atlas_2018%2Clgg_tcga_pan_can_atlas_2018%2Cbrca_tcga_pan_can_atlas_2018%2Ccesc_tcga_pan_can_atlas_2018%2Cchol_tcga_pan_can_atlas_2018%2Ccoadread_tcga_pan_can_atlas_2018%2Cdlbc_tcga_pan_can_atlas_2018%2Cesca_tcga_pan_can_atlas_2018%2Cgbm_tcga_pan_can_atlas_2018%2Chnsc_tcga_pan_can_atlas_2018%2Ckich_tcga_pan_can_atlas_2018%2Ckirc_tcga_pan_can_atlas_2018%2Ckirp_tcga_pan_can_atlas_2018%2Clihc_tcga_pan_can_atlas_2018%2Cluad_tcga_pan_can_atlas_2018%2Clusc_tcga_pan_can_atlas_2018%2Cmeso_tcga_pan_can_atlas_2018%2Cov_tcga_pan_can_atlas_2018%2Cpaad_tcga_pan_can_atlas_2018%2Cpcpg_tcga_pan_can_atlas_2018%2Cprad_tcga_pan_can_atlas_2018%2Csarc_tcga_pan_can_atlas_2018%2Cskcm_tcga_pan_can_atlas_2018%2Cstad_tcga_pan_can_atlas_2018%2Ctgct_tcga_pan_can_atlas_2018%2Cthym_tcga_pan_can_atlas_2018%2Cthca_tcga_pan_can_atlas_2018%2Cucs_tcga_pan_can_atlas_2018%2Cucec_tcga_pan_can_atlas_2018%2Cuvm_tcga_pan_can_atlas_2018&Z_SCORE_THRESHOLD=2.0&RPPA_SCORE_THRESHOLD=2.0&profileFilter=mutations%2Cstructural_variants%2Cgistic&case_set_id=w_mut&gene_list=NMU%252C%2520E2F3%252C%2520UBE2S%252C%2520DHFR%252C%2520MIA%252C%2520CHD5%252C%2520FAXDC2&geneset_list=%20&tab_index=tab_visualize&Action=Submit). The data used for drug analysis were obtained from the GSCA (http://bioinfo.life.hust.edu.cn/GSCA/#/drug) and CellMiner (https://discover.nci.nih.gov/cellminer/loadDownload.do) databases. All other data that support the conclusions of this study are provided in the article and its supplementary files.
