# Supplementary material for: Identification of the molecular subtypes and construction of risk models in neuroblastoma
Source: Sci Rep. 2023 Jul 21;13:11790. doi: 10.1038/s41598-023-35401-3 (PMC10362029; doi:10.1038/s41598-023-35401-3)
Supplement: Supplementary file 1 — Supplementary Information 1. [file 41598_2023_35401_MOESM1_ESM.pdf]

Result of Neuralnetwork gene in GDSC

Symbol	Drug	Cor	Fdr	Entrez
ARHGAP11A	(5Z)-7-Oxozeaenol	0.093418	0.015583	9824
ARHGAP11A	17-AAG	0.082695	0.02693	9824
ARHGAP11A	5-Fluorouracil	-0.08021	0.028157	9824
ARHGAP11A	681640	-0.04514	0.458381	9824
ARHGAP11A	A-443654	-0.0307	0.88938	9824
ARHGAP11A	A-770041	-0.0631	0.44017	9824
ARHGAP11A	AC220	-0.06634	0.137105	9824
ARHGAP11A	AG-014699	0.057145	0.17744	9824
ARHGAP11A	AICAR	-0.15583	3.58E-05	9824
ARHGAP11A	AKT inhibitor VIII	0.101246	0.015882	9824
ARHGAP11A	AMG-706	0.03555	0.578023	9824
ARHGAP11A	AP-24534	-0.11567	0.002752	9824
ARHGAP11A	AR-42	-0.14692	2.77E-05	9824
ARHGAP11A	AS601245	0.031469	0.589628	9824
ARHGAP11A	AS605240	-0.02107	0.665924	9824
ARHGAP11A	AT-7519	-0.17038	8.68E-07	9824
ARHGAP11A	ATRA	-0.0721	0.115502	9824
ARHGAP11A	AUY922	0.000183	0.998203	9824
ARHGAP11A	AZ628	0.122535	0.070352	9824
ARHGAP11A	AZD6482	0.016227	0.778989	9824
ARHGAP11A	AZD7762	-0.20305	4.21E-08	9824
ARHGAP11A	AZD8055	-0.11148	0.003667	9824
ARHGAP11A	Afatinib	0.115697	0.001234	9824
ARHGAP11A	Axitinib	-0.09038	0.050238	9824
ARHGAP11A	BAY 61-3606	-0.08686	0.028865	9824
ARHGAP11A	BEZ235	-0.00591	0.925496	9824
ARHGAP11A	BHG712	-0.1242	0.000449	9824
ARHGAP11A	BI-2536	-0.14488	0.096431	9824
ARHGAP11A	BIRB 0796	0.039737	0.454644	9824
ARHGAP11A	BIX02189	-0.14161	6.14E-05	9824
ARHGAP11A	BMS-509744	0.020561	0.851224	9824
ARHGAP11A	BMS-536924	0.004785	0.961099	9824
ARHGAP11A	BMS-708163	0.117321	0.002286	9824
ARHGAP11A	BMS-754807	0.075292	0.13254	9824
ARHGAP11A	BMS345541	-0.19118	3.07E-08	9824
ARHGAP11A	BX-795	-0.15615	7.5E-05	9824
ARHGAP11A	BX-912	-0.19912	5.77E-09	9824
ARHGAP11A	Belinostat	-0.12602	0.000499	9824
ARHGAP11A	Bexarotene	0.001363	0.989919	9824
ARHGAP11A	Bicalutamide	0.010602	0.845463	9824
ARHGAP11A	Bleomycin	0.040629	0.47618	9824
ARHGAP11A	Bleomycin (50 uM)	0.052519	0.157581	9824
ARHGAP11A	Bortezomib	0.010121	0.925277	9824
ARHGAP11A	Bosutinib	-0.12629	0.003265	9824
ARHGAP11A	Bryostatin 1	0.06591	0.179507	9824
ARHGAP11A	CAL-101	-0.09131	0.013451	9824
ARHGAP11A	CAY10603	-0.15832	5.52E-06	9824
ARHGAP11A	CCT007093	0.105935	0.008857	9824

ARHGAP11A	CCT018159	-0.04341	0.404289	9824
ARHGAP11A	CEP-701	-0.14584	0.000122	9824
ARHGAP11A	CGP-082996	-0.03726	0.731975	9824
ARHGAP11A	CGP-60474	-0.00515	0.96579	9824
ARHGAP11A	CH5424802	-0.08242	0.093902	9824
ARHGAP11A	CHIR-99021	0.05167	0.18521	9824
ARHGAP11A	CI-1040	0.072947	0.084734	9824
ARHGAP11A	CMK	-0.02429	0.863898	9824
ARHGAP11A	CP466722	-0.17943	2.04E-07	9824
ARHGAP11A	CP724714	0.079992	0.088055	9824
ARHGAP11A	CUDC-101	-0.15351	1.55E-05	9824
ARHGAP11A	CX-5461	-0.13067	0.000245	9824
ARHGAP11A	Camptothecin	-0.12203	0.002107	9824
ARHGAP11A	Cetuximab	0.092891	0.018281	9824
ARHGAP11A	Cisplatin	0.002922	0.965286	9824
ARHGAP11A	Crizotinib	-0.1416	0.121024	9824
ARHGAP11A	Cyclopamine	-0.04965	0.663175	9824
ARHGAP11A	Cytarabine	-0.0756	0.105074	9824
ARHGAP11A	DMOG	-0.11713	0.003586	9824
ARHGAP11A	Dabrafenib	0.121241	0.001871	9824
ARHGAP11A	Dasatinib	0.001261	0.98705	9824
ARHGAP11A	Docetaxel	0.023376	0.560482	9824
ARHGAP11A	Doxorubicin	0.011558	0.889575	9824
ARHGAP11A	EHT 1864	0.051339	0.411207	9824
ARHGAP11A	EKB-569	-0.09081	0.018713	9824
ARHGAP11A	EX-527	0.001048	0.994028	9824
ARHGAP11A	Elesclomol	-0.03672	0.433695	9824
ARHGAP11A	Embelin	0.005967	0.944775	9824
ARHGAP11A	Epothilone B	-0.01216	0.847191	9824
ARHGAP11A	Erlotinib	0.100186	0.160685	9824
ARHGAP11A	Etoposide	-0.08148	0.061897	9824
ARHGAP11A	FH535	0.091028	0.035263	9824
ARHGAP11A	FK866	-0.19131	3.89E-08	9824
ARHGAP11A	FMK	-0.06744	0.268619	9824
ARHGAP11A	FR-180204	0.004448	0.954617	9824
ARHGAP11A	FTI-277	0.118708	0.002577	9824
ARHGAP11A	Foretinib	-0.15615	2.12E-05	9824
ARHGAP11A	GDC0449	0.00552	0.971221	9824
ARHGAP11A	GDC0941	-0.05268	0.325564	9824
ARHGAP11A	GNF-2	-0.06438	0.776567	9824
ARHGAP11A	GSK-650394	-0.04003	0.589008	9824
ARHGAP11A	GSK1070916	-0.1982	1.36E-08	9824
ARHGAP11A	GSK1904529A	0.064198	0.150429	9824
ARHGAP11A	GSK2126458	-0.10159	0.005573	9824
ARHGAP11A	GSK269962A	-0.04175	0.430048	9824
ARHGAP11A	GSK429286A	-0.12365	0.00106	9824
ARHGAP11A	GSK690693	-0.13358	0.000177	9824
ARHGAP11A	GW 441756	-0.02916	0.896458	9824
ARHGAP11A	GW-2580	0.032296	0.938845	9824
ARHGAP11A	GW843682X	-0.16685	0.02286	9824

ARHGAP11A	Gefitinib	0.085952	0.033231	9824
ARHGAP11A	Gemcitabine	-0.09433	0.033276	9824
ARHGAP11A	Genentech Cpd 10	-0.18448	1.17E-07	9824
ARHGAP11A	HG-5-113-01	-0.13969	0.021947	9824
ARHGAP11A	HG-5-88-01	0.020184	0.917421	9824
ARHGAP11A	HG-6-64-1	-0.02923	0.578708	9824
ARHGAP11A	I-BET-762	-0.16009	3.07E-06	9824
ARHGAP11A	IOX2	0.025887	0.688232	9824
ARHGAP11A	IPA-3	-0.08585	0.026568	9824
ARHGAP11A	Imatinib	-0.08175	0.516345	9824
ARHGAP11A	Ispinesib Mesylate	-0.17107	1E-06	9824
ARHGAP11A	JNJ-26854165	0.007716	0.91014	9824
ARHGAP11A	JNK Inhibitor VIII	0.067525	0.108752	9824
ARHGAP11A	JNK-9L	-0.0068	0.932252	9824
ARHGAP11A	JQ1	0.006875	0.900952	9824
ARHGAP11A	JQ12	0.005576	0.93728	9824
ARHGAP11A	JW-7-24-1	-0.11639	0.00097	9824
ARHGAP11A	JW-7-52-1	-0.02096	0.869312	9824
ARHGAP11A	KIN001-055	-0.00481	0.959288	9824
ARHGAP11A	KIN001-102	-0.14309	3.89E-05	9824
ARHGAP11A	KIN001-135	0.087333	0.355628	9824
ARHGAP11A	KIN001-236	-0.10145	0.005254	9824
ARHGAP11A	KIN001-244	-0.11775	0.001032	9824
ARHGAP11A	KIN001-260	-0.1044	0.003553	9824
ARHGAP11A	KIN001-266	-0.04962	0.297283	9824
ARHGAP11A	KIN001-270	-0.13816	0.000187	9824
ARHGAP11A	KU-55933	-0.09815	0.047995	9824
ARHGAP11A	LAQ824	-0.07517	0.055512	9824
ARHGAP11A	LFM-A13	0.112687	0.012324	9824
ARHGAP11A	LY317615	-0.0983	0.015026	9824
ARHGAP11A	Lapatinib	0.146824	0.014828	9824
ARHGAP11A	Lenalidomide	-0.06464	0.314171	9824
ARHGAP11A	Linifanib	-0.06143	0.201206	9824
ARHGAP11A	Lisitinib	0.055248	0.359286	9824
ARHGAP11A	MG-132	0.030952	0.770972	9824
ARHGAP11A	MK-2206	-0.07858	0.102022	9824
ARHGAP11A	MLN4924	0.026959	0.66719	9824
ARHGAP11A	MP470	-0.04281	0.33305	9824
ARHGAP11A	MPS-1-IN-1	-0.09449	0.010571	9824
ARHGAP11A	MS-275	-0.12785	0.076006	9824
ARHGAP11A	Masitinib	-0.09538	0.009609	9824
ARHGAP11A	Methotrexate	-0.17194	1.67E-06	9824
ARHGAP11A	Midostaurin	0.006621	0.904833	9824
ARHGAP11A	Mitomycin C	-0.0619	0.203193	9824
ARHGAP11A	NG-25	-0.17122	7.9E-07	9824
ARHGAP11A	NPK76-II-72-1	-0.25153	6.76E-14	9824
ARHGAP11A	NSC-207895	-0.11396	0.004412	9824
ARHGAP11A	NSC-87877	0.125817	0.01191	9824
ARHGAP11A	NU-7441	-0.03604	0.636493	9824
ARHGAP11A	Navitoclax	-0.1953	4.76E-08	9824

ARHGAP11A	Nilotinib	-0.0972	0.030346	9824
ARHGAP11A	Nutlin-3a (-)	0.10426	0.014266	9824
ARHGAP11A	OSI-027	-0.11383	0.001394	9824
ARHGAP11A	OSI-930	-0.06199	0.111186	9824
ARHGAP11A	OSU-03012	-0.0224	0.691645	9824
ARHGAP11A	Obatoclax Mesylate	-0.02898	0.572917	9824
ARHGAP11A	Olaparib	0.015976	0.750116	9824
ARHGAP11A	PAC-1	-0.0263	0.573977	9824
ARHGAP11A	PD-0325901	0.152187	5.27E-05	9824
ARHGAP11A	PD-0332991	-0.05185	0.338565	9824
ARHGAP11A	PD-173074	0.000853	0.996072	9824
ARHGAP11A	PF-4708671	0.077285	0.358522	9824
ARHGAP11A	PF-562271	0.004421	0.952536	9824
ARHGAP11A	PFI-1	0.01711	0.762399	9824
ARHGAP11A	PHA-665752	0.005785	0.977314	9824
ARHGAP11A	PHA-793887	-0.16393	1.86E-06	9824
ARHGAP11A	PI-103	-0.1359	0.000119	9824
ARHGAP11A	PIK-93	-0.18086	1.35E-07	9824
ARHGAP11A	PLX4720	0.122652	0.00109	9824
ARHGAP11A	Paclitaxel	-0.05803	0.608264	9824
ARHGAP11A	Parthenolide	-0.06418	0.530152	9824
ARHGAP11A	Pazopanib	0.045224	0.414301	9824
ARHGAP11A	Phenformin	-0.10842	0.002897	9824
ARHGAP11A	Pyrimethamine	-0.04961	0.678431	9824
ARHGAP11A	QL-VIII-58	-0.0175	0.858985	9824
ARHGAP11A	QL-X-138	-0.13013	0.000264	9824
ARHGAP11A	QL-XI-92	-0.13904	7.93E-05	9824
ARHGAP11A	QL-XII-47	-0.09153	0.017302	9824
ARHGAP11A	QL-XII-61	-0.10026	0.100046	9824
ARHGAP11A	QS11	-0.02337	0.746167	9824
ARHGAP11A	RDEA119	0.174063	4.76E-07	9824
ARHGAP11A	RO-3306	-0.00728	0.882612	9824
ARHGAP11A	Rapamycin	-0.08112	0.453151	9824
ARHGAP11A	Roscovitine	0.030727	0.882245	9824
ARHGAP11A	Ruxolitinib	-0.05545	0.255876	9824
ARHGAP11A	S-Trityl-L-cysteine	-0.12314	0.093488	9824
ARHGAP11A	SB 216763	0.006555	0.91105	9824
ARHGAP11A	SB 505124	0.040994	0.586202	9824
ARHGAP11A	SB52334	0.053835	0.243577	9824
ARHGAP11A	SB590885	0.15857	9.12E-05	9824
ARHGAP11A	SGC0946	0.080284	0.083447	9824
ARHGAP11A	SL 0101-1	0.071959	0.324786	9824
ARHGAP11A	SN-38	-0.04373	0.335089	9824
ARHGAP11A	SNX-2112	-0.175	5.31E-07	9824
ARHGAP11A	STF-62247	-0.08446	0.031139	9824
ARHGAP11A	Salubrinal	-0.07346	0.379438	9824
ARHGAP11A	Saracatinib	-0.0838	0.300616	9824
ARHGAP11A	Shikonin	-0.0397	0.47001	9824
ARHGAP11A	Sorafenib	-0.08554	0.373454	9824
ARHGAP11A	Sunitinib	-0.14803	0.022232	9824

ARHGAP11A	T0901317	-0.08239	0.036873	9824
ARHGAP11A	TAE684	-0.07601	0.465305	9824
ARHGAP11A	TAK-715	-0.13882	8.35E-05	9824
ARHGAP11A	TG101348	-0.14725	2.4E-05	9824
ARHGAP11A	TGX221	0.096533	0.12922	9824
ARHGAP11A	THZ-2-102-1	-0.15692	8E-06	9824
ARHGAP11A	THZ-2-49	-0.1297	0.000303	9824
ARHGAP11A	TL-1-85	-0.16263	3.05E-06	9824
ARHGAP11A	TL-2-105	-0.13333	0.000204	9824
ARHGAP11A	TPCA-1	-0.17336	4.61E-07	9824
ARHGAP11A	TW 37	-0.08816	0.03355	9824
ARHGAP11A	Talazoparib	0.014619	0.781984	9824
ARHGAP11A	Tamoxifen	0.029387	0.81836	9824
ARHGAP11A	Temozolomide	-0.021	0.803411	9824
ARHGAP11A	Temsirolimus	-0.09506	0.0284	9824
ARHGAP11A	Thapsigargin	-0.03016	0.675294	9824
ARHGAP11A	Tipifarnib	0.012206	0.889332	9824
ARHGAP11A	Tivozanib	-0.04065	0.491313	9824
ARHGAP11A	Trametinib	0.196053	2.26E-08	9824
ARHGAP11A	Tubastatin A	-0.13677	9.41E-05	9824
ARHGAP11A	UNC0638	-0.09416	0.007293	9824
ARHGAP11A	UNC1215	0.089948	0.047297	9824
ARHGAP11A	VNLG/124	-0.05286	0.204246	9824
ARHGAP11A	VX-11e	0.066473	0.139476	9824
ARHGAP11A	VX-680	-0.13367	0.092521	9824
ARHGAP11A	VX-702	-0.02131	0.934547	9824
ARHGAP11A	Veliparib	-0.02195	0.778334	9824
ARHGAP11A	Vinblastine	-0.15177	0.000112	9824
ARHGAP11A	Vinorelbine	-0.06203	0.229799	9824
ARHGAP11A	Vorinostat	-0.19684	3.34E-08	9824
ARHGAP11A	WH-4-023	0.012407	0.897452	9824
ARHGAP11A	WZ-1-84	-0.00857	0.930177	9824
ARHGAP11A	WZ3105	-0.15052	1.48E-05	9824
ARHGAP11A	XAV939	0.052215	0.188084	9824
ARHGAP11A	XL-184	-0.10473	0.010997	9824
ARHGAP11A	XMD11-85h	0.040778	0.827965	9824
ARHGAP11A	XMD13-2	-0.14249	4.75E-05	9824
ARHGAP11A	XMD14-99	-0.04911	0.209457	9824
ARHGAP11A	XMD15-27	-0.08055	0.064296	9824
ARHGAP11A	XMD8-85	-0.03392	0.718202	9824
ARHGAP11A	XMD8-92	-0.03829	0.744379	9824
ARHGAP11A	Y-39983	-0.17207	1.13E-06	9824
ARHGAP11A	YK 4-279	-0.06	0.240853	9824
ARHGAP11A	YM155	-0.10329	0.019539	9824
ARHGAP11A	YM201636	-0.1096	0.002298	9824
ARHGAP11A	Z-LLNle-CHO	0.006732	0.944087	9824
ARHGAP11A	ZG-10	-0.11792	0.047372	9824
ARHGAP11A	ZM-447439	-0.15407	0.000133	9824
ARHGAP11A	ZSTK474	-0.11734	0.000997	9824
ARHGAP11A	Zibotentan	0.009701	0.986223	9824

ARHGAP11A	piperlongumine	-0.00981	0.85721	9824
ARHGAP11A	rTRAIL	0.020102	0.817668	9824
ARHGAP11A	selumetinib	0.199744	6.21E-09	9824
BLM	(5Z)-7-Oxozeaenol	0.093278	0.015747	641
BLM	17-AAG	0.28726	0	641
BLM	5-Fluorouracil	-0.23696	6.33E-12	641
BLM	681640	0.034644	0.583849	641
BLM	A-443654	0.032094	0.880956	641
BLM	A-770041	0.060029	0.468188	641
BLM	AC220	-0.12907	0.001294	641
BLM	AG-014699	0.107963	0.007353	641
BLM	AICAR	-0.23373	1.79E-10	641
BLM	AKT inhibitor VIII	0.171085	2.31E-05	641
BLM	AMG-706	0.115066	0.017227	641
BLM	AP-24534	-0.138	0.000283	641
BLM	AR-42	-0.30396	1.44E-19	641
BLM	AS601245	0.107201	0.018425	641
BLM	AS605240	-0.11372	0.00371	641
BLM	AT-7519	-0.30797	2.84E-20	641
BLM	ATRA	-0.16853	2.52E-05	641
BLM	AUY922	0.031115	0.587193	641
BLM	AZ628	0.086575	0.229396	641
BLM	AZD6482	0.102832	0.010392	641
BLM	AZD7762	-0.21894	2.78E-09	641
BLM	AZD8055	-0.19374	1.83E-07	641
BLM	Afatinib	0.147767	2.66E-05	641
BLM	Axitinib	-0.10749	0.016765	641
BLM	BAY 61-3606	-0.11078	0.00445	641
BLM	BEZ235	0.080525	0.072626	641
BLM	BHG712	-0.25274	1.14E-13	641
BLM	BI-2536	-0.11557	0.19977	641
BLM	BIRB 0796	0.136682	0.002619	641
BLM	BIX02189	-0.237	4.97E-12	641
BLM	BMS-509744	0.090229	0.300756	641
BLM	BMS-536924	-0.06641	0.247873	641
BLM	BMS-708163	0.148416	9.9E-05	641
BLM	BMS-754807	0.006595	0.927125	641
BLM	BMS345541	-0.30613	5.59E-20	641
BLM	BX-795	-0.11557	0.004599	641
BLM	BX-912	-0.30852	1.63E-20	641
BLM	Belinostat	-0.24227	3.9E-12	641
BLM	Bexarotene	0.035613	0.64884	641
BLM	Bicalutamide	0.11194	0.005963	641
BLM	Bleomycin	0.054153	0.313074	641
BLM	Bleomycin (50 uM)	0.208625	1.81E-09	641
BLM	Bortezomib	0.109923	0.120164	641
BLM	Bosutinib	-0.0535	0.298513	641
BLM	Bryostatin 1	0.117649	0.009251	641
BLM	CAL-101	-0.17468	8.1E-07	641
BLM	CAY10603	-0.26655	3.86E-15	641

BLM	CCT007093	0.133467	0.000779	641
BLM	CCT018159	0.023227	0.688515	641
BLM	CEP-701	-0.1898	3.37E-07	641
BLM	CGP-082996	0.037387	0.731182	641
BLM	CGP-60474	0.087183	0.273213	641
BLM	CH5424802	-0.11555	0.009502	641
BLM	CHIR-99021	0.164944	3.3E-06	641
BLM	CI-1040	0.070651	0.096709	641
BLM	CMK	0.040414	0.754487	641
BLM	CP466722	-0.2917	3.25E-18	641
BLM	CP724714	0.062482	0.21188	641
BLM	CUDC-101	-0.25648	1.03E-13	641
BLM	CX-5461	-0.23614	7.3E-12	641
BLM	Camptothecin	-0.13434	0.000635	641
BLM	Cetuximab	0.174144	3.18E-06	641
BLM	Cisplatin	0.037411	0.467927	641
BLM	Crizotinib	-0.03903	0.809381	641
BLM	Cyclopamine	-0.00153	0.990894	641
BLM	Cytarabine	-0.02677	0.636019	641
BLM	DMOG	-0.10819	0.007795	641
BLM	Dabrafenib	0.062205	0.142968	641
BLM	Dasatinib	0.179412	0.003035	641
BLM	Docetaxel	0.185865	3.17E-07	641
BLM	Doxorubicin	-0.00678	0.936764	641
BLM	EHT 1864	0.052317	0.40035	641
BLM	EKB-569	-0.16138	1.15E-05	641
BLM	EX-527	-0.01399	0.916455	641
BLM	Elesclomol	0.08833	0.036013	641
BLM	Embelin	0.040369	0.505003	641
BLM	Epothilone B	0.031355	0.567125	641
BLM	Erlotinib	0.220813	0.000486	641
BLM	Etoposide	-0.12593	0.002229	641
BLM	FH535	0.119271	0.004294	641
BLM	FK866	-0.29095	1.06E-17	641
BLM	FMK	-0.04892	0.462813	641
BLM	FR-180204	-0.0916	0.043262	641
BLM	FTI-277	0.227259	2.59E-09	641
BLM	Foretinib	-0.1723	2.33E-06	641
BLM	GDC0449	-0.02108	0.858755	641
BLM	GDC0941	-0.02881	0.644744	641
BLM	GNF-2	-0.01385	0.968565	641
BLM	GSK-650394	-0.03806	0.611263	641
BLM	GSK1070916	-0.33247	4.62E-23	641
BLM	GSK1904529A	0.14367	0.000487	641
BLM	GSK2126458	-0.21868	3.52E-10	641
BLM	GSK269962A	0.056104	0.252542	641
BLM	GSK429286A	-0.1569	2.26E-05	641
BLM	GSK690693	-0.24424	1.15E-12	641
BLM	GW 441756	0.037351	0.851374	641
BLM	GW-2580	0.02648	0.957241	641

BLM	GW843682X	-0.15973	0.030008	641
BLM	Gefitinib	0.129332	0.000892	641
BLM	Gemcitabine	-0.09363	0.034812	641
BLM	Genentech Cpd 10	-0.26874	3.23E-15	641
BLM	HG-5-113-01	-0.14826	0.014475	641
BLM	HG-5-88-01	-0.06883	0.585817	641
BLM	HG-6-64-1	0.031767	0.541256	641
BLM	I-BET-762	-0.32703	3.25E-23	641
BLM	IOX2	0.075672	0.149591	641
BLM	IPA-3	-0.16096	1.37E-05	641
BLM	Imatinib	-0.07932	0.537252	641
BLM	Ispinesib Mesylate	-0.23467	8.49E-12	641
BLM	JNJ-26854165	0.073487	0.114067	641
BLM	JNK Inhibitor VIII	0.186037	1.56E-06	641
BLM	JNK-9L	-0.01444	0.839491	641
BLM	JQ1	-0.05576	0.189098	641
BLM	JQ12	-0.08125	0.100028	641
BLM	JW-7-24-1	-0.26612	3.35E-15	641
BLM	JW-7-52-1	0.040055	0.719739	641
BLM	KIN001-055	0.002011	0.982967	641
BLM	KIN001-102	-0.29179	2.82E-18	641
BLM	KIN001-135	0.144276	0.090375	641
BLM	KIN001-236	-0.23787	5.48E-12	641
BLM	KIN001-244	-0.22276	1.31E-10	641
BLM	KIN001-260	-0.23754	4.18E-12	641
BLM	KIN001-266	-0.12121	0.004288	641
BLM	KIN001-270	-0.1385	0.00018	641
BLM	KU-55933	-0.09159	0.068968	641
BLM	LAQ824	-0.21059	7.71E-09	641
BLM	LFM-A13	0.150901	0.00062	641
BLM	LY317615	-0.13208	0.000747	641
BLM	Lapatinib	0.255724	6.35E-06	641
BLM	Lenalidomide	-0.05451	0.430169	641
BLM	Linifanib	-0.08723	0.049445	641
BLM	Lisitinib	-0.02235	0.766536	641
BLM	MG-132	0.10651	0.168886	641
BLM	MK-2206	-0.07427	0.125111	641
BLM	MLN4924	0.069325	0.19763	641
BLM	MP470	-0.11121	0.007431	641
BLM	MPS-1-IN-1	-0.24796	1.39E-12	641
BLM	MS-275	-0.22183	0.001119	641
BLM	Masitinib	-0.20904	2.39E-09	641
BLM	Methotrexate	-0.3486	2.35E-24	641
BLM	Midostaurin	0.117424	0.003865	641
BLM	Mitomycin C	-0.12221	0.004862	641
BLM	NG-25	-0.27263	6.46E-16	641
BLM	NPK76-II-72-1	-0.40888	4.16E-37	641
BLM	NSC-207895	-0.15612	6.85E-05	641
BLM	NSC-87877	0.136941	0.006399	641
BLM	NU-7441	-0.00841	0.939532	641

BLM	Navitoclax	-0.35438	3.96E-25	641
BLM	Nilotinib	-0.13346	0.001744	641
BLM	Nutlin-3a (-)	0.031697	0.54127	641
BLM	OSI-027	-0.25293	1.39E-13	641
BLM	OSI-930	-0.16708	3.86E-06	641
BLM	OSU-03012	-0.0781	0.086492	641
BLM	Obatoclax Mesylate	-0.0507	0.284509	641
BLM	Olaparib	0.063479	0.139283	641
BLM	PAC-1	-0.17904	2.26E-06	641
BLM	PD-0325901	0.184368	7.08E-07	641
BLM	PD-0332991	-0.00645	0.934014	641
BLM	PD-173074	0.001431	0.995206	641
BLM	PF-4708671	0.062409	0.480577	641
BLM	PF-562271	0.059911	0.262237	641
BLM	PFI-1	-0.02512	0.640396	641
BLM	PHA-665752	0.126921	0.256685	641
BLM	PHA-793887	-0.33119	8.25E-24	641
BLM	PI-103	-0.23886	3.31E-12	641
BLM	PIK-93	-0.32614	5.23E-23	641
BLM	PLX4720	0.073937	0.06558	641
BLM	Paclitaxel	0.009754	0.954465	641
BLM	Parthenolide	-0.05937	0.568091	641
BLM	Pazopanib	0.101901	0.030121	641
BLM	Phenformin	-0.19622	2.82E-08	641
BLM	Pyrimethamine	-0.10344	0.32129	641
BLM	QL-VIII-58	0.043842	0.591948	641
BLM	QL-X-138	-0.24597	8.8E-13	641
BLM	QL-XI-92	-0.26991	1.68E-15	641
BLM	QL-XII-47	-0.16706	5.44E-06	641
BLM	QL-XII-61	-0.2221	4.07E-05	641
BLM	QS11	-0.09873	0.063496	641
BLM	RDEA119	0.178038	2.49E-07	641
BLM	RO-3306	0.190285	1.1E-06	641
BLM	Rapamycin	-0.00332	0.985107	641
BLM	Roscovitine	0.07529	0.628141	641
BLM	Ruxolitinib	-0.08964	0.042808	641
BLM	S-Trityl-L-cysteine	-0.11394	0.12291	641
BLM	SB 216763	0.13303	0.003305	641
BLM	SB 505124	0.05601	0.419375	641
BLM	SB52334	-0.03199	0.534128	641
BLM	SB590885	0.097195	0.029865	641
BLM	SGC0946	0.116149	0.008195	641
BLM	SL 0101-1	0.080461	0.271668	641
BLM	SN-38	-0.03791	0.413174	641
BLM	SNX-2112	-0.25625	6.19E-14	641
BLM	STF-62247	-0.13107	0.000566	641
BLM	Salubrinal	-0.0804	0.326859	641
BLM	Saracatinib	0.067615	0.416361	641
BLM	Shikonin	-0.0461	0.388693	641
BLM	Sorafenib	-0.03	0.817103	641

BLM	Sunitinib	-0.11943	0.076353	641
BLM	T0901317	-0.15028	5.72E-05	641
BLM	TAE684	0.035499	0.790368	641
BLM	TAK-715	-0.23306	1.3E-11	641
BLM	TG101348	-0.2819	4.68E-17	641
BLM	TGX221	0.255273	7.33E-06	641
BLM	THZ-2-102-1	-0.3146	9.23E-21	641
BLM	THZ-2-49	-0.21283	1.16E-09	641
BLM	TL-1-85	-0.27047	1.25E-15	641
BLM	TL-2-105	-0.22666	6.98E-11	641
BLM	TPCA-1	-0.33581	2.16E-24	641
BLM	TW 37	-0.02824	0.580789	641
BLM	Talazoparib	-0.00808	0.88478	641
BLM	Tamoxifen	0.019577	0.893631	641
BLM	Temozolomide	-0.02191	0.793349	641
BLM	Temsirolimus	-0.00842	0.891765	641
BLM	Thapsigargin	0.002306	0.981564	641
BLM	Tipifarnib	-0.014	0.871107	641
BLM	Tivozanib	-0.0758	0.125413	641
BLM	Trametinib	0.233455	1.8E-11	641
BLM	Tubastatin A	-0.29586	1.05E-18	641
BLM	UNC0638	-0.24751	9.09E-14	641
BLM	UNC1215	0.125265	0.004678	641
BLM	VNLG/124	-0.14925	6.16E-05	641
BLM	VX-11e	-0.02706	0.610011	641
BLM	VX-680	-0.15012	0.052731	641
BLM	VX-702	-0.05257	0.76319	641
BLM	Veliparib	0.042609	0.537101	641
BLM	Vinblastine	-0.1232	0.002113	641
BLM	Vinorelbine	-0.08483	0.080575	641
BLM	Vorinostat	-0.36752	3.49E-27	641
BLM	WH-4-023	0.148682	0.027144	641
BLM	WZ-1-84	0.139607	0.042963	641
BLM	WZ3105	-0.3108	9.93E-21	641
BLM	XAV939	0.136025	0.000249	641
BLM	XL-184	-0.09517	0.022829	641
BLM	XMD11-85h	-0.00745	0.976453	641
BLM	XMD13-2	-0.26555	4.16E-15	641
BLM	XMD14-99	-0.1909	7.25E-08	641
BLM	XMD15-27	-0.12656	0.001808	641
BLM	XMD8-85	0.019039	0.852918	641
BLM	XMD8-92	-0.05769	0.59218	641
BLM	Y-39983	-0.22535	9.23E-11	641
BLM	YK 4-279	-0.00181	0.98255	641
BLM	YM155	-0.09756	0.028756	641
BLM	YM201636	-0.22507	7.42E-11	641
BLM	Z-LLNle-CHO	0.038455	0.641148	641
BLM	ZG-10	-0.15617	0.006293	641
BLM	ZM-447439	-0.19284	1.27E-06	641
BLM	ZSTK474	-0.23781	4.17E-12	641

BLM	Zibotentan	0.050232	0.810004	641
BLM	piperlongumine	0.095541	0.017192	641
BLM	rTRAIL	0.037993	0.614922	641
BLM	selumetinib	0.183066	1.18E-07	641
CCNB1	(5Z)-7-Oxozeaenol	0.068809	0.086596	891
CCNB1	17-AAG	-0.0128	0.759727	891
CCNB1	5-Fluorouracil	0.121978	0.000639	891
CCNB1	681640	-0.05627	0.338983	891
CCNB1	A-443654	-0.03421	0.869564	891
CCNB1	A-770041	-0.05496	0.516176	891
CCNB1	AC220	0.050937	0.276553	891
CCNB1	AG-014699	0.002685	0.960821	891
CCNB1	AICAR	0.029887	0.484577	891
CCNB1	AKT inhibitor VIII	0.052388	0.248152	891
CCNB1	AMG-706	-0.02645	0.705054	891
CCNB1	AP-24534	0.049279	0.243188	891
CCNB1	AR-42	0.076765	0.033589	891
CCNB1	AS601245	5.09E-05	0.999289	891
CCNB1	AS605240	0.048908	0.260163	891
CCNB1	AT-7519	0.052506	0.15385	891
CCNB1	ATRA	0.035125	0.499392	891
CCNB1	AUY922	0.032278	0.569917	891
CCNB1	AZ628	0.074856	0.313301	891
CCNB1	AZD6482	0.011452	0.851196	891
CCNB1	AZD7762	-0.03565	0.406526	891
CCNB1	AZD8055	0.009747	0.831058	891
CCNB1	Afatinib	0.020531	0.617035	891
CCNB1	Axitinib	-0.01331	0.842765	891
CCNB1	BAY 61-3606	0.093735	0.017561	891
CCNB1	BEZ235	-0.04505	0.364062	891
CCNB1	BHG712	0.095642	0.007759	891
CCNB1	BI-2536	-0.07349	0.45925	891
CCNB1	BIRB 0796	-0.02226	0.69985	891
CCNB1	BIX02189	0.055022	0.142253	891
CCNB1	BMS-509744	-0.05719	0.546124	891
CCNB1	BMS-536924	0.012592	0.893479	891
CCNB1	BMS-708163	0.001201	0.981798	891
CCNB1	BMS-754807	0.036608	0.519562	891
CCNB1	BMS345541	0.04759	0.201408	891
CCNB1	BX-795	-0.02701	0.596483	891
CCNB1	BX-912	0.045496	0.217455	891
CCNB1	Belinostat	0.06952	0.063622	891
CCNB1	Bexarotene	-0.01846	0.844375	891
CCNB1	Bicalutamide	-0.05043	0.258092	891
CCNB1	Bleomycin	0.052217	0.334435	891
CCNB1	Bleomycin (50 uM)	-0.02268	0.563378	891
CCNB1	Bortezomib	-0.01426	0.891559	891
CCNB1	Bosutinib	-0.0246	0.688792	891
CCNB1	Bryostatin 1	0.010741	0.871327	891
CCNB1	CAL-101	0.053018	0.169837	891

CCNB1	CAY10603	0.062127	0.088619	891
CCNB1	CCT007093	0.013529	0.794061	891
CCNB1	CCT018159	-0.03548	0.511012	891
CCNB1	CEP-701	0.018049	0.695273	891
CCNB1	CGP-082996	-0.07522	0.405351	891
CCNB1	CGP-60474	-0.05176	0.567751	891
CCNB1	CH5424802	-0.00155	0.985643	891
CCNB1	CHIR-99021	-0.07544	0.045494	891
CCNB1	CI-1040	0.05424	0.218641	891
CCNB1	CMK	0.017004	0.910123	891
CCNB1	CP466722	0.024254	0.533305	891
CCNB1	CP724714	0.038651	0.48944	891
CCNB1	CUDC-101	0.069597	0.060768	891
CCNB1	CX-5461	0.063924	0.085623	891
CCNB1	Camptothecin	-0.017	0.743879	891
CCNB1	Cetuximab	-0.01551	0.736683	891
CCNB1	Cisplatin	-0.03212	0.541142	891
CCNB1	Crizotinib	-0.04548	0.771471	891
CCNB1	Cyclopamine	0.029708	0.815587	891
CCNB1	Cytarabine	-0.00775	0.904879	891
CCNB1	DMOG	0.002954	0.960183	891
CCNB1	Dabrafenib	0.065556	0.12021	891
CCNB1	Dasatinib	-0.04063	0.573675	891
CCNB1	Docetaxel	-0.08754	0.019384	891
CCNB1	Doxorubicin	0.013896	0.862727	891
CCNB1	EHT 1864	0.022695	0.773617	891
CCNB1	EKB-569	0.027126	0.53685	891
CCNB1	EX-527	-0.02446	0.842704	891
CCNB1	Elesclomol	-0.1271	0.00176	891
CCNB1	Embelin	-0.00579	0.946552	891
CCNB1	Epothilone B	-0.01576	0.795654	891
CCNB1	Erlotinib	-0.00468	0.959556	891
CCNB1	Etoposide	-0.03437	0.500917	891
CCNB1	FH535	0.032205	0.536354	891
CCNB1	FK866	0.058925	0.113047	891
CCNB1	FMK	0.031699	0.665096	891
CCNB1	FR-180204	0.040872	0.442976	891
CCNB1	FTI-277	0.073899	0.070498	891
CCNB1	Foretinib	0.035997	0.413839	891
CCNB1	GDC0449	0.035581	0.728598	891
CCNB1	GDC0941	-0.03056	0.620092	891
CCNB1	GNF-2	-0.03329	0.901926	891
CCNB1	GSK-650394	0.00325	0.974298	891
CCNB1	GSK1070916	0.032074	0.413637	891
CCNB1	GSK1904529A	-0.02692	0.591591	891
CCNB1	GSK2126458	0.048951	0.205289	891
CCNB1	GSK269962A	0.037293	0.490163	891
CCNB1	GSK429286A	0.012023	0.805711	891
CCNB1	GSK690693	0.0063	0.883193	891
CCNB1	GW 441756	-0.02109	0.93525	891

CCNB1	GW-2580	0.039787	0.91528	891
CCNB1	GW843682X	-0.08776	0.290148	891
CCNB1	Gefitinib	0.031091	0.476496	891
CCNB1	Gemcitabine	-0.03773	0.465035	891
CCNB1	Genentech Cpd 10	0.019021	0.643566	891
CCNB1	HG-5-113-01	-0.0227	0.790685	891
CCNB1	HG-5-88-01	0.051318	0.731338	891
CCNB1	HG-6-64-1	0.053776	0.254965	891
CCNB1	I-BET-762	0.100156	0.004176	891
CCNB1	IOX2	-0.06147	0.263168	891
CCNB1	IPA-3	0.023772	0.582329	891
CCNB1	Imatinib	0.003183	0.989531	891
CCNB1	Ispinesib Mesylate	0.085812	0.018608	891
CCNB1	JNJ-26854165	-0.05384	0.277875	891
CCNB1	JNK Inhibitor VIII	-0.09479	0.020359	891
CCNB1	JNK-9L	0.061044	0.246898	891
CCNB1	JQ1	0.08854	0.02763	891
CCNB1	JQ12	0.07954	0.108526	891
CCNB1	JW-7-24-1	0.059192	0.105767	891
CCNB1	JW-7-52-1	-0.05558	0.592252	891
CCNB1	KIN001-055	0.016759	0.840057	891
CCNB1	KIN001-102	0.063122	0.081634	891
CCNB1	KIN001-135	0.071566	0.470889	891
CCNB1	KIN001-236	0.121628	0.000721	891
CCNB1	KIN001-244	0.075102	0.041767	891
CCNB1	KIN001-260	0.087097	0.015965	891
CCNB1	KIN001-266	0.049611	0.297344	891
CCNB1	KIN001-270	-0.03463	0.40636	891
CCNB1	KU-55933	-0.03423	0.602721	891
CCNB1	LAQ824	0.072775	0.064566	891
CCNB1	LFM-A13	0.006223	0.92481	891
CCNB1	LY317615	0.014323	0.780566	891
CCNB1	Lapatinib	0.008559	0.90736	891
CCNB1	Lenalidomide	-0.02387	0.794019	891
CCNB1	Linifanib	0.065167	0.170199	891
CCNB1	Lisitinib	0.03763	0.576414	891
CCNB1	MG-132	-0.02071	0.858851	891
CCNB1	MK-2206	-0.04919	0.339364	891
CCNB1	MLN4924	-0.00599	0.936349	891
CCNB1	MP470	-0.01891	0.693437	891
CCNB1	MPS-1-IN-1	0.05385	0.162207	891
CCNB1	MS-275	-0.03004	0.755098	891
CCNB1	Masitinib	0.078538	0.035365	891
CCNB1	Methotrexate	0.092672	0.01225	891
CCNB1	Midostaurin	-0.02112	0.674646	891
CCNB1	Mitomycin C	0.04892	0.339568	891
CCNB1	NG-25	0.088048	0.014236	891
CCNB1	NPK76-II-72-1	0.032936	0.37748	891
CCNB1	NSC-207895	-0.06662	0.120941	891
CCNB1	NSC-87877	0.058413	0.275929	891

CCNB1	NU-7441	-0.02502	0.76763	891
CCNB1	Navitoclax	0.018198	0.661345	891
CCNB1	Nilotinib	0.01166	0.855181	891
CCNB1	Nutlin-3a (-)	0.120314	0.003978	891
CCNB1	OSI-027	0.054219	0.144385	891
CCNB1	OSI-930	0.0733	0.056499	891
CCNB1	OSU-03012	0.037885	0.46608	891
CCNB1	Obatoclax Mesylate	0.050884	0.28272	891
CCNB1	Olaparib	0.008492	0.872826	891
CCNB1	PAC-1	0.060481	0.150047	891
CCNB1	PD-0325901	0.06712	0.094937	891
CCNB1	PD-0332991	0.036963	0.527174	891
CCNB1	PD-173074	-0.00741	0.979441	891
CCNB1	PF-4708671	0.061272	0.489113	891
CCNB1	PF-562271	-0.01289	0.851432	891
CCNB1	PFI-1	0.097347	0.02356	891
CCNB1	PHA-665752	0.020084	0.907554	891
CCNB1	PHA-793887	0.084733	0.016618	891
CCNB1	PI-103	0.052653	0.157032	891
CCNB1	PIK-93	0.058894	0.104528	891
CCNB1	PLX4720	0.068292	0.09183	891
CCNB1	Paclitaxel	-0.04321	0.721052	891
CCNB1	Parthenolide	0.026198	0.832206	891
CCNB1	Pazopanib	0.000546	0.995322	891
CCNB1	Phenformin	0.088566	0.016104	891
CCNB1	Pyrimethamine	0.045044	0.71041	891
CCNB1	QL-VIII-58	-0.0157	0.876424	891
CCNB1	QL-X-138	0.01999	0.620402	891
CCNB1	QL-XI-92	0.040683	0.284838	891
CCNB1	QL-XII-47	0.024061	0.585097	891
CCNB1	QL-XII-61	0.004011	0.96356	891
CCNB1	QS11	0.004377	0.960656	891
CCNB1	RDEA119	0.104558	0.003478	891
CCNB1	RO-3306	-0.04856	0.255936	891
CCNB1	Rapamycin	-0.03587	0.798678	891
CCNB1	Roscovitine	0.049642	0.780379	891
CCNB1	Ruxolitinib	0.002986	0.965076	891
CCNB1	S-Trityl-L-cysteine	0.001231	0.99194	891
CCNB1	SB 216763	-0.01951	0.723957	891
CCNB1	SB 505124	0.045281	0.535354	891
CCNB1	SB52334	0.037465	0.451765	891
CCNB1	SB590885	0.091688	0.042812	891
CCNB1	SGC0946	-0.00732	0.918951	891
CCNB1	SL 0101-1	0.044197	0.564616	891
CCNB1	SN-38	0.036548	0.433749	891
CCNB1	SNX-2112	0.098412	0.006303	891
CCNB1	STF-62247	0.022371	0.619749	891
CCNB1	Salubrinal	0.054304	0.537266	891
CCNB1	Saracatinib	-0.07687	0.347251	891
CCNB1	Shikonin	-0.00539	0.938042	891

CCNB1	Sorafenib	0.070388	0.496596	891
CCNB1	Sunitinib	0.014049	0.885209	891
CCNB1	T0901317	0.048848	0.242106	891
CCNB1	TAE684	-0.0625	0.572844	891
CCNB1	TAK-715	0.059434	0.108275	891
CCNB1	TG101348	0.066756	0.066378	891
CCNB1	TGX221	0.026005	0.717674	891
CCNB1	THZ-2-102-1	0.057803	0.118406	891
CCNB1	THZ-2-49	0.053012	0.160724	891
CCNB1	TL-1-85	0.075239	0.03905	891
CCNB1	TL-2-105	0.042137	0.278121	891
CCNB1	TPCA-1	0.05594	0.124637	891
CCNB1	TW 37	-0.09083	0.027695	891
CCNB1	Talazoparib	0.027714	0.573024	891
CCNB1	Tamoxifen	0.008431	0.960748	891
CCNB1	Temozolomide	0.041865	0.557393	891
CCNB1	Temsirolimus	-0.07266	0.111594	891
CCNB1	Thapsigargin	-0.01158	0.897145	891
CCNB1	Tipifarnib	0.037459	0.586391	891
CCNB1	Tivozanib	-0.00955	0.902498	891
CCNB1	Trametinib	0.096098	0.008879	891
CCNB1	Tubastatin A	0.104694	0.003148	891
CCNB1	UNC0638	0.086104	0.014488	891
CCNB1	UNC1215	0.047935	0.342783	891
CCNB1	VNLG/124	0.056096	0.174512	891
CCNB1	VX-11e	0.097238	0.021652	891
CCNB1	VX-680	-0.0291	0.795079	891
CCNB1	VX-702	0.026833	0.913331	891
CCNB1	Veliparib	-0.03193	0.659169	891
CCNB1	Vinblastine	-0.02765	0.570155	891
CCNB1	Vinorelbine	-0.00709	0.922851	891
CCNB1	Vorinostat	0.015381	0.702052	891
CCNB1	WH-4-023	0.005166	0.959715	891
CCNB1	WZ-1-84	-0.01765	0.851019	891
CCNB1	WZ3105	0.061958	0.0885	891
CCNB1	XAV939	-0.06505	0.095349	891
CCNB1	XL-184	0.052742	0.252232	891
CCNB1	XMD11-85h	0.107295	0.377694	891
CCNB1	XMD13-2	0.097125	0.006528	891
CCNB1	XMD14-99	0.103909	0.004786	891
CCNB1	XMD15-27	0.029974	0.55885	891
CCNB1	XMD8-85	0.064592	0.439317	891
CCNB1	XMD8-92	0.046684	0.675385	891
CCNB1	Y-39983	0.02955	0.465982	891
CCNB1	YK 4-279	0.007914	0.914306	891
CCNB1	YM155	-0.02058	0.736219	891
CCNB1	YM201636	0.035964	0.354971	891
CCNB1	Z-LLNle-CHO	-0.00569	0.952586	891
CCNB1	ZG-10	-0.00154	0.98628	891
CCNB1	ZM-447439	0.014759	0.790278	891

CCNB1	ZSTK474	0.020579	0.60643	891
CCNB1	Zibotentan	-0.01497	0.969121	891
CCNB1	piperlongumine	-0.03857	0.396574	891
CCNB1	rTRAIL	-0.00853	0.931104	891
CCNB1	selumetinib	0.109832	0.002176	891
CDCA2	(5Z)-7-Oxozeaenol	0.090891	0.018983	157313
CDCA2	17-AAG	0.14857	4.3E-05	157313
CDCA2	5-Fluorouracil	-0.03311	0.392127	157313
CDCA2	681640	0.022927	0.730986	157313
CDCA2	A-443654	-0.03086	0.888908	157313
CDCA2	A-770041	-0.04548	0.60414	157313
CDCA2	AC220	-0.02382	0.661888	157313
CDCA2	AG-014699	0.067733	0.104777	157313
CDCA2	AICAR	-0.04813	0.241854	157313
CDCA2	AKT inhibitor VIII	0.101983	0.015062	157313
CDCA2	AMG-706	0.036429	0.567678	157313
CDCA2	AP-24534	-0.1002	0.010405	157313
CDCA2	AR-42	-0.15334	1.17E-05	157313
CDCA2	AS601245	0.084998	0.074849	157313
CDCA2	AS605240	0.011021	0.832458	157313
CDCA2	AT-7519	-0.12617	0.00033	157313
CDCA2	ATRA	-0.01714	0.76766	157313
CDCA2	AUY922	0.034571	0.537334	157313
CDCA2	AZ628	0.110373	0.110175	157313
CDCA2	AZD6482	-0.02139	0.697348	157313
CDCA2	AZD7762	-0.18764	4.71E-07	157313
CDCA2	AZD8055	-0.12668	0.000875	157313
CDCA2	Afatinib	0.13917	8.19E-05	157313
CDCA2	Axitinib	-0.05407	0.289623	157313
CDCA2	BAY 61-3606	-0.07339	0.068793	157313
CDCA2	BEZ235	-0.04508	0.363624	157313
CDCA2	BHG712	-0.11473	0.001256	157313
CDCA2	BI-2536	-0.07677	0.43329	157313
CDCA2	BIRB 0796	0.035566	0.511729	157313
CDCA2	BIX02189	-0.134	0.000157	157313
CDCA2	BMS-509744	0.081319	0.357612	157313
CDCA2	BMS-536924	0.048561	0.455789	157313
CDCA2	BMS-708163	0.094011	0.015978	157313
CDCA2	BMS-754807	0.095567	0.04518	157313
CDCA2	BMS345541	-0.13364	0.000143	157313
CDCA2	BX-795	-0.08374	0.049985	157313
CDCA2	BX-912	-0.17437	4.11E-07	157313
CDCA2	Belinostat	-0.12965	0.000335	157313
CDCA2	Bexarotene	0.022523	0.79797	157313
CDCA2	Bicalutamide	0.026742	0.583845	157313
CDCA2	Bleomycin	0.07748	0.119015	157313
CDCA2	Bleomycin (50 uM)	0.081441	0.024628	157313
CDCA2	Bortezomib	0.011948	0.910363	157313
CDCA2	Bosutinib	-0.0561	0.269678	157313
CDCA2	Bryostatin 1	0.064667	0.189112	157313

CDCA2	CAL-101	-0.12603	0.000492	157313
CDCA2	CAY10603	-0.16333	2.66E-06	157313
CDCA2	CCT007093	0.108481	0.007178	157313
CDCA2	CCT018159	0.018225	0.764486	157313
CDCA2	CEP-701	-0.13619	0.000358	157313
CDCA2	CGP-082996	0.017079	0.892291	157313
CDCA2	CGP-60474	-0.02704	0.790006	157313
CDCA2	CH5424802	-0.03548	0.570392	157313
CDCA2	CHIR-99021	0.007025	0.878293	157313
CDCA2	CI-1040	0.126046	0.001473	157313
CDCA2	CMK	0.000165	0.999035	157313
CDCA2	CP466722	-0.15134	1.4E-05	157313
CDCA2	CP724714	0.072326	0.133455	157313
CDCA2	CUDC-101	-0.12752	0.000381	157313
CDCA2	CX-5461	-0.10998	0.00223	157313
CDCA2	Camptothecin	-0.09474	0.02064	157313
CDCA2	Cetuximab	0.081929	0.039396	157313
CDCA2	Cisplatin	-0.04447	0.377923	157313
CDCA2	Crizotinib	-0.11308	0.271785	157313
CDCA2	Cyclopamine	-0.00114	0.993163	157313
CDCA2	Cytarabine	-0.0406	0.436681	157313
CDCA2	DMOG	-0.10426	0.010627	157313
CDCA2	Dabrafenib	0.099863	0.012397	157313
CDCA2	Dasatinib	0.01607	0.837893	157313
CDCA2	Docetaxel	0.102409	0.005871	157313
CDCA2	Doxorubicin	0.005319	0.94991	157313
CDCA2	EHT 1864	0.061068	0.311348	157313
CDCA2	EKB-569	-0.0474	0.249987	157313
CDCA2	EX-527	-0.00791	0.95587	157313
CDCA2	Elesclomol	-0.05489	0.217317	157313
CDCA2	Embelin	-0.00151	0.987657	157313
CDCA2	Epothilone B	0.057954	0.243649	157313
CDCA2	Erlotinib	0.13439	0.049034	157313
CDCA2	Etoposide	-0.08475	0.050736	157313
CDCA2	FH535	0.092148	0.032665	157313
CDCA2	FK866	-0.15504	1.05E-05	157313
CDCA2	FMK	-0.02618	0.73161	157313
CDCA2	FR-180204	0.009923	0.885524	157313
CDCA2	FTI-277	0.121486	0.00201	157313
CDCA2	Foretinib	-0.10993	0.003793	157313
CDCA2	GDC0449	0.052462	0.562297	157313
CDCA2	GDC0941	-0.08502	0.072429	157313
CDCA2	GNF-2	-0.03368	0.899601	157313
CDCA2	GSK-650394	-0.00868	0.932412	157313
CDCA2	GSK1070916	-0.15747	8.55E-06	157313
CDCA2	GSK1904529A	0.047998	0.297933	157313
CDCA2	GSK2126458	-0.1202	0.000912	157313
CDCA2	GSK269962A	-0.0096	0.885664	157313
CDCA2	GSK429286A	-0.12999	0.000541	157313
CDCA2	GSK690693	-0.1867	8.82E-08	157313

CDCA2	GW 441756	-0.035	0.863925	157313
CDCA2	GW-2580	-0.03214	0.941018	157313
CDCA2	GW843682X	-0.08528	0.307503	157313
CDCA2	Gefitinib	0.130222	0.000818	157313
CDCA2	Gemcitabine	-0.05845	0.221736	157313
CDCA2	Genentech Cpd 10	-0.1622	3.78E-06	157313
CDCA2	HG-5-113-01	-0.05724	0.426955	157313
CDCA2	HG-5-88-01	0.011321	0.959238	157313
CDCA2	HG-6-64-1	-0.01696	0.768582	157313
CDCA2	I-BET-762	-0.17921	1.55E-07	157313
CDCA2	IOX2	-0.05043	0.373041	157313
CDCA2	IPA-3	-0.10842	0.004377	157313
CDCA2	Imatinib	-0.09029	0.442539	157313
CDCA2	Ispinesib Mesylate	-0.08	0.028839	157313
CDCA2	JNJ-26854165	0.020996	0.722032	157313
CDCA2	JNK Inhibitor VIII	0.050246	0.244961	157313
CDCA2	JNK-9L	0.008689	0.908188	157313
CDCA2	JQ1	0.046637	0.281814	157313
CDCA2	JQ12	0.009153	0.894897	157313
CDCA2	JW-7-24-1	-0.16264	2.79E-06	157313
CDCA2	JW-7-52-1	0.009765	0.942538	157313
CDCA2	KIN001-055	0.043204	0.489814	157313
CDCA2	KIN001-102	-0.12117	0.000544	157313
CDCA2	KIN001-135	0.08788	0.352689	157313
CDCA2	KIN001-236	-0.0965	0.008085	157313
CDCA2	KIN001-244	-0.14709	3.34E-05	157313
CDCA2	KIN001-260	-0.11202	0.001694	157313
CDCA2	KIN001-266	-0.04492	0.351782	157313
CDCA2	KIN001-270	-0.15969	1.32E-05	157313
CDCA2	KU-55933	-0.05291	0.360959	157313
CDCA2	LAQ824	-0.04183	0.31156	157313
CDCA2	LFM-A13	0.090532	0.050184	157313
CDCA2	LY317615	-0.02133	0.664488	157313
CDCA2	Lapatinib	0.185139	0.001633	157313
CDCA2	Lenalidomide	-0.02959	0.729375	157313
CDCA2	Linifanib	-0.00243	0.973119	157313
CDCA2	Lisitinib	0.085563	0.102964	157313
CDCA2	MG-132	-0.00881	0.943324	157313
CDCA2	MK-2206	-0.1387	0.001752	157313
CDCA2	MLN4924	0.06594	0.224499	157313
CDCA2	MP470	-0.05566	0.19647	157313
CDCA2	MPS-1-IN-1	-0.0556	0.148046	157313
CDCA2	MS-275	-0.11381	0.121639	157313
CDCA2	Masitinib	-0.09266	0.012016	157313
CDCA2	Methotrexate	-0.11369	0.001897	157313
CDCA2	Midostaurin	0.031183	0.516509	157313
CDCA2	Mitomycin C	-0.00586	0.932814	157313
CDCA2	NG-25	-0.15237	1.25E-05	157313
CDCA2	NPK76-II-72-1	-0.20293	2.3E-09	157313
CDCA2	NSC-207895	-0.17854	4.81E-06	157313

CDCA2	NSC-87877	0.111855	0.026046	157313
CDCA2	NU-7441	-0.05471	0.416049	157313
CDCA2	Navitoclax	-0.18057	4.93E-07	157313
CDCA2	Nilotinib	-0.07229	0.128095	157313
CDCA2	Nutlin-3a (-)	0.072225	0.108422	157313
CDCA2	OSI-027	-0.15879	5.91E-06	157313
CDCA2	OSI-930	-0.06529	0.092236	157313
CDCA2	OSU-03012	0.008708	0.892511	157313
CDCA2	Obatoclax Mesylate	0.00705	0.906679	157313
CDCA2	Olaparib	0.016771	0.737631	157313
CDCA2	PAC-1	-0.03445	0.445212	157313
CDCA2	PD-0325901	0.177821	1.79E-06	157313
CDCA2	PD-0332991	0.026943	0.667157	157313
CDCA2	PD-173074	-0.02994	0.85747	157313
CDCA2	PF-4708671	0.068905	0.427385	157313
CDCA2	PF-562271	0.047308	0.394529	157313
CDCA2	PFI-1	0.031754	0.54148	157313
CDCA2	PHA-665752	0.017333	0.922026	157313
CDCA2	PHA-793887	-0.12867	0.000213	157313
CDCA2	PI-103	-0.17074	1.02E-06	157313
CDCA2	PIK-93	-0.19228	1.9E-08	157313
CDCA2	PLX4720	0.116315	0.002092	157313
CDCA2	Paclitaxel	0.019452	0.897498	157313
CDCA2	Parthenolide	-0.07024	0.481517	157313
CDCA2	Pazopanib	-0.00369	0.961579	157313
CDCA2	Phenformin	-0.03464	0.378835	157313
CDCA2	Pyrimethamine	-0.02862	0.827896	157313
CDCA2	QL-VIII-58	-0.0452	0.580591	157313
CDCA2	QL-X-138	-0.17653	4.76E-07	157313
CDCA2	QL-XI-92	-0.17559	4.54E-07	157313
CDCA2	QL-XII-47	-0.09395	0.014404	157313
CDCA2	QL-XII-61	-0.14172	0.014105	157313
CDCA2	QS11	-0.0363	0.576978	157313
CDCA2	RDEA119	0.194441	1.48E-08	157313
CDCA2	RO-3306	0.040679	0.349147	157313
CDCA2	Rapamycin	-0.09523	0.358681	157313
CDCA2	Roscovitine	0.026043	0.904201	157313
CDCA2	Ruxolitinib	-0.05438	0.26704	157313
CDCA2	S-Trityl-L-cysteine	-0.04594	0.603524	157313
CDCA2	SB 216763	0.064091	0.188268	157313
CDCA2	SB 505124	0.023497	0.790364	157313
CDCA2	SB52334	0.005588	0.932346	157313
CDCA2	SB590885	0.146577	0.000375	157313
CDCA2	SGC0946	0.032266	0.572407	157313
CDCA2	SL 0101-1	0.103198	0.167083	157313
CDCA2	SN-38	-0.03519	0.454549	157313
CDCA2	SNX-2112	-0.12395	0.000493	157313
CDCA2	STF-62247	-0.09228	0.017912	157313
CDCA2	Salubrinal	-0.01374	0.899943	157313
CDCA2	Saracatinib	0.000538	0.996767	157313

CDCA2	Shikonin	-0.03405	0.547907	157313
CDCA2	Sorafenib	0.007847	0.95713	157313
CDCA2	Sunitinib	-0.04392	0.591305	157313
CDCA2	T0901317	-0.11176	0.003591	157313
CDCA2	TAE684	-0.00844	0.960986	157313
CDCA2	TAK-715	-0.10749	0.002635	157313
CDCA2	TG101348	-0.13652	9.54E-05	157313
CDCA2	TGX221	0.077277	0.235025	157313
CDCA2	THZ-2-102-1	-0.13405	0.000153	157313
CDCA2	THZ-2-49	-0.09808	0.00707	157313
CDCA2	TL-1-85	-0.14548	3.34E-05	157313
CDCA2	TL-2-105	-0.12676	0.000431	157313
CDCA2	TPCA-1	-0.16342	2.16E-06	157313
CDCA2	TW 37	-0.09521	0.019916	157313
CDCA2	Talazoparib	-0.01706	0.743426	157313
CDCA2	Tamoxifen	0.020447	0.887011	157313
CDCA2	Temozolomide	0.006528	0.94989	157313
CDCA2	Temsirolimus	-0.13391	0.001137	157313
CDCA2	Thapsigargin	0.014051	0.872214	157313
CDCA2	Tipifarnib	0.037428	0.586869	157313
CDCA2	Tivozanib	-0.06078	0.2493	157313
CDCA2	Trametinib	0.217921	4.05E-10	157313
CDCA2	Tubastatin A	-0.11979	0.000675	157313
CDCA2	UNC0638	-0.10354	0.002999	157313
CDCA2	UNC1215	0.095366	0.034307	157313
CDCA2	VNLG/124	-0.03852	0.375278	157313
CDCA2	VX-11e	0.078253	0.073863	157313
CDCA2	VX-680	-0.04774	0.635282	157313
CDCA2	VX-702	0.009242	0.978789	157313
CDCA2	Veliparib	0.030646	0.674069	157313
CDCA2	Vinblastine	-0.05689	0.195075	157313
CDCA2	Vinorelbine	-0.02501	0.68669	157313
CDCA2	Vorinostat	-0.18083	4.31E-07	157313
CDCA2	WH-4-023	0.032786	0.710206	157313
CDCA2	WZ-1-84	0.070225	0.368426	157313
CDCA2	WZ3105	-0.10682	0.002502	157313
CDCA2	XAV939	0.046316	0.246913	157313
CDCA2	XL-184	-0.04691	0.319015	157313
CDCA2	XMD11-85h	0.027903	0.889602	157313
CDCA2	XMD13-2	-0.11546	0.001111	157313
CDCA2	XMD14-99	-0.0428	0.280342	157313
CDCA2	XMD15-27	-0.08407	0.051699	157313
CDCA2	XMD8-85	-0.01294	0.903739	157313
CDCA2	XMD8-92	-0.013	0.933282	157313
CDCA2	Y-39983	-0.16846	1.94E-06	157313
CDCA2	YK 4-279	0.034266	0.551397	157313
CDCA2	YM155	-0.08198	0.074229	157313
CDCA2	YM201636	-0.13876	9.19E-05	157313
CDCA2	Z-LLNle-CHO	0.003146	0.97421	157313
CDCA2	ZG-10	-0.09259	0.136618	157313

CDCA2	ZM-447439	-0.05703	0.214054	157313
CDCA2	ZSTK474	-0.15106	1.75E-05	157313
CDCA2	Zibotentan	-0.03222	0.912262	157313
CDCA2	piperlongumine	-0.00146	0.981545	157313
CDCA2	rTRAIL	0.002384	0.980991	157313
CDCA2	selumetinib	0.202378	3.81E-09	157313
CDK1	(5Z)-7-Oxozeaenol	0.172772	2.02E-06	983
CDK1	17-AAG	0.158261	1.24E-05	983
CDK1	5-Fluorouracil	-0.00799	0.847245	983
CDK1	681640	-0.01302	0.858135	983
CDK1	A-443654	-0.04182	0.829446	983
CDK1	A-770041	-0.05273	0.537015	983
CDK1	AC220	0.002804	0.969529	983
CDK1	AG-014699	0.053846	0.206906	983
CDK1	AICAR	-0.13437	0.000409	983
CDK1	AKT inhibitor VIII	0.069197	0.113972	983
CDK1	AMG-706	0.066391	0.221527	983
CDK1	AP-24534	-0.01311	0.788653	983
CDK1	AR-42	-0.10077	0.004714	983
CDK1	AS601245	0.064425	0.20496	983
CDK1	AS605240	0.011326	0.827666	983
CDK1	AT-7519	-0.12071	0.000607	983
CDK1	ATRA	-0.01906	0.739821	983
CDK1	AUY922	0.046092	0.378785	983
CDK1	AZ628	0.172251	0.006652	983
CDK1	AZD6482	0.066374	0.12567	983
CDK1	AZD7762	-0.1035	0.007762	983
CDK1	AZD8055	-0.05557	0.168001	983
CDK1	Afatinib	0.040033	0.301972	983
CDK1	Axitinib	-0.03205	0.573863	983
CDK1	BAY 61-3606	0.007878	0.87289	983
CDK1	BEZ235	0.038468	0.449998	983
CDK1	BHG712	-0.018	0.650549	983
CDK1	BI-2536	-0.09069	0.337273	983
CDK1	BIRB 0796	0.066519	0.175431	983
CDK1	BIX02189	-0.03561	0.356424	983
CDK1	BMS-509744	0.041537	0.673367	983
CDK1	BMS-536924	0.012558	0.89367	983
CDK1	BMS-708163	0.07244	0.067244	983
CDK1	BMS-754807	0.046164	0.397845	983
CDK1	BMS345541	-0.12703	0.00031	983
CDK1	BX-795	-0.0194	0.71773	983
CDK1	BX-912	-0.11117	0.001588	983
CDK1	Belinostat	-0.07952	0.032383	983
CDK1	Bexarotene	0.034476	0.663467	983
CDK1	Bicalutamide	0.025305	0.606194	983
CDK1	Bleomycin	0.083449	0.088606	983
CDK1	Bleomycin (50 uM)	0.11313	0.001513	983
CDK1	Bortezomib	0.052263	0.534312	983
CDK1	Bosutinib	-0.00909	0.89366	983

CDK1	Bryostatin 1	0.066747	0.172717	983
CDK1	CAL-101	-0.05845	0.127186	983
CDK1	CAY10603	-0.08996	0.011964	983
CDK1	CCT007093	0.059566	0.165962	983
CDK1	CCT018159	0.016849	0.784875	983
CDK1	CEP-701	-0.02791	0.529351	983
CDK1	CGP-082996	-0.03651	0.738624	983
CDK1	CGP-60474	0.025678	0.802919	983
CDK1	CH5424802	-0.00274	0.974402	983
CDK1	CHIR-99021	0.081604	0.029478	983
CDK1	CI-1040	0.115051	0.004028	983
CDK1	CMK	-0.00638	0.967322	983
CDK1	CP466722	-0.09638	0.006875	983
CDK1	CP724714	-0.00713	0.9231	983
CDK1	CUDC-101	-0.10677	0.003201	983
CDK1	CX-5461	-0.09539	0.008515	983
CDK1	Camptothecin	-0.04903	0.274415	983
CDK1	Cetuximab	0.053364	0.196898	983
CDK1	Cisplatin	0.019362	0.737092	983
CDK1	Crizotinib	-0.01237	0.955692	983
CDK1	Cyclopamine	0.00557	0.968941	983
CDK1	Cytarabine	-0.02564	0.651639	983
CDK1	DMOG	-0.01656	0.759859	983
CDK1	Dabrafenib	0.155776	4.09E-05	983
CDK1	Dasatinib	0.02059	0.790974	983
CDK1	Docetaxel	0.060351	0.113679	983
CDK1	Doxorubicin	0.020895	0.781579	983
CDK1	EHT 1864	0.043589	0.505634	983
CDK1	EKB-569	-0.06162	0.123845	983
CDK1	EX-527	0.001283	0.99329	983
CDK1	Elesclomol	-0.02744	0.572222	983
CDK1	Embelin	0.016481	0.82521	983
CDK1	Epothilone B	0.020427	0.726623	983
CDK1	Erlotinib	0.085625	0.238772	983
CDK1	Etoposide	-0.06593	0.14314	983
CDK1	FH535	0.080396	0.068753	983
CDK1	FK866	-0.11744	0.00099	983
CDK1	FMK	0.044104	0.519834	983
CDK1	FR-180204	0.041593	0.433944	983
CDK1	FTI-277	0.160255	3.36E-05	983
CDK1	Foretinib	-0.03842	0.378662	983
CDK1	GDC0449	0.037304	0.711235	983
CDK1	GDC0941	-0.03512	0.556146	983
CDK1	GNF-2	-0.0239	0.934089	983
CDK1	GSK-650394	-0.00299	0.976576	983
CDK1	GSK1070916	-0.1247	0.000509	983
CDK1	GSK1904529A	0.050554	0.27117	983
CDK1	GSK2126458	-0.08019	0.031149	983
CDK1	GSK269962A	0.065162	0.165704	983
CDK1	GSK429286A	-0.06388	0.117764	983

CDK1	GSK690693	-0.14489	4.35E-05	983
CDK1	GW 441756	-0.00412	0.994189	983
CDK1	GW-2580	0.039146	0.91569	983
CDK1	GW843682X	-0.11331	0.145083	983
CDK1	Gefitinib	0.056799	0.173065	983
CDK1	Gemcitabine	-0.04441	0.376003	983
CDK1	Genentech Cpd 10	-0.10181	0.004882	983
CDK1	HG-5-113-01	-0.01832	0.834416	983
CDK1	HG-5-88-01	0.04499	0.773421	983
CDK1	HG-6-64-1	0.074673	0.092341	983
CDK1	I-BET-762	-0.08828	0.012039	983
CDK1	IOX2	0.008745	0.909945	983
CDK1	IPA-3	-0.07076	0.071922	983
CDK1	Imatinib	-0.04411	0.79589	983
CDK1	Ispinesib Mesylate	-0.05142	0.173639	983
CDK1	JNJ-26854165	0.047717	0.346963	983
CDK1	JNK Inhibitor VIII	0.043837	0.317784	983
CDK1	JNK-9L	0.034675	0.565764	983
CDK1	JQ1	0.01982	0.681389	983
CDK1	JQ12	0.020536	0.742929	983
CDK1	JW-7-24-1	-0.0856	0.016866	983
CDK1	JW-7-52-1	-0.0132	0.921887	983
CDK1	KIN001-055	0.031128	0.654803	983
CDK1	KIN001-102	-0.12871	0.000231	983
CDK1	KIN001-135	0.08779	0.352689	983
CDK1	KIN001-236	-0.03866	0.32014	983
CDK1	KIN001-244	-0.05939	0.113313	983
CDK1	KIN001-260	-0.02827	0.470487	983
CDK1	KIN001-266	-0.03029	0.555114	983
CDK1	KIN001-270	-0.0994	0.008578	983
CDK1	KU-55933	-0.04987	0.396553	983
CDK1	LAQ824	-0.08642	0.025827	983
CDK1	LFM-A13	0.085013	0.068776	983
CDK1	LY317615	-0.04482	0.31549	983
CDK1	Lapatinib	0.087453	0.166652	983
CDK1	Lenalidomide	0.00119	0.991669	983
CDK1	Linifanib	0.028619	0.616649	983
CDK1	Lisitinib	0.032957	0.633911	983
CDK1	MG-132	0.063233	0.478385	983
CDK1	MK-2206	-0.08886	0.059568	983
CDK1	MLN4924	0.084041	0.108304	983
CDK1	MP470	-0.06071	0.156669	983
CDK1	MPS-1-IN-1	-0.0474	0.223297	983
CDK1	MS-275	-0.11436	0.119222	983
CDK1	Masitinib	-0.02973	0.462715	983
CDK1	Methotrexate	-0.08449	0.022997	983
CDK1	Midostaurin	0.07559	0.076639	983
CDK1	Mitomycin C	-0.01151	0.854461	983
CDK1	NG-25	-0.07382	0.042002	983
CDK1	NPK76-II-72-1	-0.15108	1.16E-05	983

CDK1	NSC-207895	-0.12756	0.001291	983
CDK1	NSC-87877	0.081996	0.111565	983
CDK1	NU-7441	-0.00181	0.986645	983
CDK1	Navitoclax	-0.16309	6.23E-06	983
CDK1	Nilotinib	-0.04473	0.391039	983
CDK1	Nutlin-3a (-)	0.144797	0.000399	983
CDK1	OSI-027	-0.08458	0.019247	983
CDK1	OSI-930	-0.03143	0.44913	983
CDK1	OSU-03012	-0.0292	0.589908	983
CDK1	Obatoclax Mesylate	0.024797	0.639695	983
CDK1	Olaparib	0.031719	0.493313	983
CDK1	PAC-1	-0.05096	0.235056	983
CDK1	PD-0325901	0.176233	2.24E-06	983
CDK1	PD-0332991	0.050224	0.35836	983
CDK1	PD-173074	-0.00319	0.991024	983
CDK1	PF-4708671	0.07321	0.387433	983
CDK1	PF-562271	0.04215	0.456784	983
CDK1	PFI-1	0.049092	0.305921	983
CDK1	PHA-665752	0.081721	0.550842	983
CDK1	PHA-793887	-0.11037	0.001594	983
CDK1	PI-103	-0.09976	0.005379	983
CDK1	PIK-93	-0.10545	0.00271	983
CDK1	PLX4720	0.148691	5.06E-05	983
CDK1	Paclitaxel	-0.01453	0.926965	983
CDK1	Parthenolide	-0.01655	0.899876	983
CDK1	Pazopanib	0.068264	0.181468	983
CDK1	Phenformin	-0.05338	0.160554	983
CDK1	Pyrimethamine	-0.02107	0.882179	983
CDK1	QL-VIII-58	0.046307	0.569	983
CDK1	QL-X-138	-0.12113	0.000721	983
CDK1	QL-XI-92	-0.08809	0.014542	983
CDK1	QL-XII-47	-0.08129	0.03632	983
CDK1	QL-XII-61	-0.09044	0.145016	983
CDK1	QS11	-0.05248	0.38162	983
CDK1	RDEA119	0.2087	1.03E-09	983
CDK1	RO-3306	0.069381	0.096335	983
CDK1	Rapamycin	-0.05976	0.615582	983
CDK1	Roscovitine	0.090767	0.546252	983
CDK1	Ruxolitinib	-0.01153	0.861297	983
CDK1	S-Trityl-L-cysteine	-0.03258	0.730867	983
CDK1	SB 216763	0.069555	0.149211	983
CDK1	SB 505124	0.036968	0.633335	983
CDK1	SB52334	0.031645	0.539096	983
CDK1	SB590885	0.165666	3.76E-05	983
CDK1	SGC0946	0.041713	0.441302	983
CDK1	SL 0101-1	0.085487	0.243324	983
CDK1	SN-38	0.021923	0.661797	983
CDK1	SNX-2112	-0.06826	0.064265	983
CDK1	STF-62247	-0.00792	0.874075	983
CDK1	Salubrinal	0.010094	0.927962	983

CDK1	Saracatinib	-0.03137	0.737717	983
CDK1	Shikonin	-0.04014	0.463475	983
CDK1	Sorafenib	0.043842	0.715299	983
CDK1	Sunitinib	-0.027	0.759165	983
CDK1	T0901317	-0.07953	0.044574	983
CDK1	TAE684	0.028336	0.83596	983
CDK1	TAK-715	-0.08556	0.018053	983
CDK1	TG101348	-0.07263	0.044829	983
CDK1	TGX221	0.149812	0.013451	983
CDK1	THZ-2-102-1	-0.11515	0.001235	983
CDK1	THZ-2-49	-0.05011	0.187138	983
CDK1	TL-1-85	-0.06568	0.074159	983
CDK1	TL-2-105	-0.0838	0.023203	983
CDK1	TPCA-1	-0.09153	0.00984	983
CDK1	TW 37	-0.05927	0.184542	983
CDK1	Talazoparib	0.031071	0.519518	983
CDK1	Tamoxifen	0.021414	0.879546	983
CDK1	Temozolomide	0.052773	0.429114	983
CDK1	Temsirolimus	-0.03051	0.57195	983
CDK1	Thapsigargin	0.019414	0.809418	983
CDK1	Tipifarnib	0.034471	0.624002	983
CDK1	Tivozanib	-0.01422	0.845878	983
CDK1	Trametinib	0.238988	5.56E-12	983
CDK1	Tubastatin A	-0.05661	0.121874	983
CDK1	UNC0638	-0.02928	0.43719	983
CDK1	UNC1215	0.108034	0.01538	983
CDK1	VNLG/124	-0.00495	0.923793	983
CDK1	VX-11e	0.100375	0.017199	983
CDK1	VX-680	-0.08265	0.353554	983
CDK1	VX-702	0.016316	0.950801	983
CDK1	Veliparib	0.025055	0.738955	983
CDK1	Vinblastine	-0.01488	0.776547	983
CDK1	Vinorelbine	-0.0255	0.679171	983
CDK1	Vorinostat	-0.17573	9.3E-07	983
CDK1	WH-4-023	0.04581	0.583777	983
CDK1	WZ-1-84	0.035136	0.686136	983
CDK1	WZ3105	-0.11304	0.001329	983
CDK1	XAV939	0.046498	0.245144	983
CDK1	XL-184	0.001955	0.973989	983
CDK1	XMD11-85h	0.068858	0.656339	983
CDK1	XMD13-2	-0.06095	0.096227	983
CDK1	XMD14-99	0.020464	0.629016	983
CDK1	XMD15-27	-0.00367	0.952979	983
CDK1	XMD8-85	0.064247	0.442249	983
CDK1	XMD8-92	0.012524	0.935321	983
CDK1	Y-39983	-0.08543	0.020957	983
CDK1	YK 4-279	0.066418	0.186093	983
CDK1	YM155	-0.06594	0.169247	983
CDK1	YM201636	-0.09146	0.011838	983
CDK1	Z-LLNle-CHO	0.023174	0.792014	983

CDK1	ZG-10	-0.03042	0.688341	983
CDK1	ZM-447439	-0.06389	0.156544	983
CDK1	ZSTK474	-0.10704	0.0028	983
CDK1	Zibotentan	0.008119	0.987731	983
CDK1	piperlongumine	0.05851	0.171487	983
CDK1	rTRAIL	0.053734	0.442152	983
CDK1	selumetinib	0.219792	1.26E-10	983
CENPA	(5Z)-7-Oxozeaenol	0.140186	0.000155	1058
CENPA	17-AAG	0.115227	0.001728	1058
CENPA	5-Fluorouracil	0.061247	0.098826	1058
CENPA	681640	-0.0181	0.793773	1058
CENPA	A-443654	-0.02495	0.913117	1058
CENPA	A-770041	-0.00623	0.954775	1058
CENPA	AC220	0.002501	0.972824	1058
CENPA	AG-014699	0.047315	0.274656	1058
CENPA	AICAR	-0.08567	0.028883	1058
CENPA	AKT inhibitor VIII	0.080748	0.060541	1058
CENPA	AMG-706	0.017108	0.814632	1058
CENPA	AP-24534	-0.00534	0.918065	1058
CENPA	AR-42	-0.02838	0.458321	1058
CENPA	AS601245	0.057846	0.264692	1058
CENPA	AS605240	0.028886	0.53719	1058
CENPA	AT-7519	-0.03144	0.408251	1058
CENPA	ATRA	-0.05104	0.296174	1058
CENPA	AUY922	0.051212	0.316271	1058
CENPA	AZ628	0.13697	0.038921	1058
CENPA	AZD6482	0.033193	0.507513	1058
CENPA	AZD7762	-0.10196	0.008797	1058
CENPA	AZD8055	-0.04368	0.287786	1058
CENPA	Afatinib	0.059062	0.116061	1058
CENPA	Axitinib	-0.02514	0.676628	1058
CENPA	BAY 61-3606	0.04789	0.253529	1058
CENPA	BEZ235	0.025606	0.638697	1058
CENPA	BHG712	0.010795	0.791025	1058
CENPA	BI-2536	-0.05434	0.616231	1058
CENPA	BIRB 0796	0.023502	0.682023	1058
CENPA	BIX02189	-0.0275	0.483657	1058
CENPA	BMS-509744	0.022098	0.839988	1058
CENPA	BMS-536924	0.007371	0.940502	1058
CENPA	BMS-708163	0.055231	0.172942	1058
CENPA	BMS-754807	0.060684	0.244297	1058
CENPA	BMS345541	-0.05843	0.112028	1058
CENPA	BX-795	-0.02585	0.616507	1058
CENPA	BX-912	-0.04348	0.239897	1058
CENPA	Belinostat	-0.01074	0.798728	1058
CENPA	Bexarotene	-0.00383	0.974622	1058
CENPA	Bicalutamide	0.014107	0.788651	1058
CENPA	Bleomycin	0.070852	0.161694	1058
CENPA	Bleomycin (50 uM)	0.091348	0.01122	1058
CENPA	Bortezomib	0.041722	0.636714	1058

CENPA	Bosutinib	-0.03654	0.518302	1058
CENPA	Bryostatin 1	0.054551	0.282833	1058
CENPA	CAL-101	-0.00508	0.91038	1058
CENPA	CAY10603	-0.01866	0.634585	1058
CENPA	CCT007093	0.045185	0.311284	1058
CENPA	CCT018159	0.017029	0.781968	1058
CENPA	CEP-701	-0.03758	0.382587	1058
CENPA	CGP-082996	-0.03658	0.738333	1058
CENPA	CGP-60474	0.036847	0.703677	1058
CENPA	CH5424802	-0.00414	0.960907	1058
CENPA	CHIR-99021	0.046032	0.242905	1058
CENPA	CI-1040	0.114804	0.004118	1058
CENPA	CMK	0.085272	0.418731	1058
CENPA	CP466722	-0.05302	0.150724	1058
CENPA	CP724714	0.068499	0.161393	1058
CENPA	CUDC-101	-0.02176	0.589616	1058
CENPA	CX-5461	-0.04208	0.273002	1058
CENPA	Camptothecin	-0.03843	0.408577	1058
CENPA	Cetuximab	0.043375	0.302892	1058
CENPA	Cisplatin	-0.01302	0.8311	1058
CENPA	Crizotinib	0.00532	0.983743	1058
CENPA	Cyclopamine	0.087887	0.361637	1058
CENPA	Cytarabine	-0.02639	0.640675	1058
CENPA	DMOG	-0.01439	0.793903	1058
CENPA	Dabrafenib	0.086627	0.033274	1058
CENPA	Dasatinib	0.041976	0.559544	1058
CENPA	Docetaxel	0.038997	0.31856	1058
CENPA	Doxorubicin	0.02247	0.762512	1058
CENPA	EHT 1864	0.034892	0.62018	1058
CENPA	EKB-569	-0.01673	0.71973	1058
CENPA	EX-527	-0.04147	0.695894	1058
CENPA	Elesclomol	-0.05616	0.205541	1058
CENPA	Embelin	0.050241	0.380301	1058
CENPA	Epothilone B	0.024457	0.666036	1058
CENPA	Erlotinib	0.057873	0.439286	1058
CENPA	Etoposide	-0.0526	0.261243	1058
CENPA	FH535	0.08317	0.058131	1058
CENPA	FK866	-0.06101	0.100096	1058
CENPA	FMK	0.004662	0.959615	1058
CENPA	FR-180204	0.027568	0.635637	1058
CENPA	FTI-277	0.12593	0.001326	1058
CENPA	Foretinib	0.007298	0.884637	1058
CENPA	GDC0449	0.031032	0.772887	1058
CENPA	GDC0941	-0.02575	0.688083	1058
CENPA	GNF-2	-0.04102	0.876686	1058
CENPA	GSK-650394	0.006639	0.949196	1058
CENPA	GSK1070916	-0.06874	0.065175	1058
CENPA	GSK1904529A	0.026651	0.595963	1058
CENPA	GSK2126458	-0.01352	0.751816	1058
CENPA	GSK269962A	0.079116	0.077904	1058

CENPA	GSK429286A	-0.02538	0.580392	1058
CENPA	GSK690693	-0.09037	0.013334	1058
CENPA	GW 441756	-0.06103	0.678263	1058
CENPA	GW-2580	0.022375	0.967411	1058
CENPA	GW843682X	-0.06362	0.477124	1058
CENPA	Gefitinib	0.063459	0.124813	1058
CENPA	Gemcitabine	-0.05028	0.305976	1058
CENPA	Genentech Cpd 10	-0.06516	0.080969	1058
CENPA	HG-5-113-01	-0.01217	0.895432	1058
CENPA	HG-5-88-01	0.062328	0.640517	1058
CENPA	HG-6-64-1	0.052177	0.271918	1058
CENPA	I-BET-762	-0.02562	0.494922	1058
CENPA	IOX2	-0.01318	0.857877	1058
CENPA	IPA-3	-0.01958	0.657918	1058
CENPA	Imatinib	-0.01945	0.918989	1058
CENPA	Ispinesib Mesylate	-0.00436	0.917987	1058
CENPA	JNJ-26854165	0.003384	0.963452	1058
CENPA	JNK Inhibitor VIII	0.015188	0.752315	1058
CENPA	JNK-9L	0.064688	0.213763	1058
CENPA	JQ1	0.045799	0.292106	1058
CENPA	JQ12	0.048839	0.366616	1058
CENPA	JW-7-24-1	-0.03845	0.307269	1058
CENPA	JW-7-52-1	0.023753	0.84771	1058
CENPA	KIN001-055	0.0194	0.807385	1058
CENPA	KIN001-102	-0.05068	0.168268	1058
CENPA	KIN001-135	0.082836	0.386588	1058
CENPA	KIN001-236	0.00318	0.943815	1058
CENPA	KIN001-244	-0.00852	0.841979	1058
CENPA	KIN001-260	-0.01949	0.628234	1058
CENPA	KIN001-266	0.019593	0.71592	1058
CENPA	KIN001-270	-0.03768	0.361894	1058
CENPA	KU-55933	-0.05083	0.385496	1058
CENPA	LAQ824	-0.02665	0.534871	1058
CENPA	LFM-A13	0.040023	0.447099	1058
CENPA	LY317615	-0.00452	0.936496	1058
CENPA	Lapatinib	0.077376	0.226059	1058
CENPA	Lenalidomide	-0.04134	0.583835	1058
CENPA	Linifanib	0.025544	0.664397	1058
CENPA	Lisitinib	0.033336	0.628956	1058
CENPA	MG-132	0.030885	0.771443	1058
CENPA	MK-2206	-0.0638	0.19773	1058
CENPA	MLN4924	0.035241	0.559105	1058
CENPA	MP470	-0.00466	0.930618	1058
CENPA	MPS-1-IN-1	0.014719	0.73105	1058
CENPA	MS-275	-0.08759	0.259917	1058
CENPA	Masitinib	-0.00325	0.943371	1058
CENPA	Methotrexate	-0.05305	0.165411	1058
CENPA	Midostaurin	0.069311	0.108486	1058
CENPA	Mitomycin C	-0.0099	0.877011	1058
CENPA	NG-25	-0.02535	0.516518	1058

CENPA	NPK76-II-72-1	-0.10085	0.004069	1058
CENPA	NSC-207895	-0.09011	0.02851	1058
CENPA	NSC-87877	0.035875	0.539601	1058
CENPA	NU-7441	-0.01119	0.914306	1058
CENPA	Navitoclax	-0.14263	8.75E-05	1058
CENPA	Nilotinib	-0.03417	0.533791	1058
CENPA	Nutlin-3a (-)	0.123451	0.003045	1058
CENPA	OSI-027	-0.02247	0.569101	1058
CENPA	OSI-930	0.019713	0.650364	1058
CENPA	OSU-03012	0.024493	0.659374	1058
CENPA	Obatoclax Mesylate	0.046236	0.335438	1058
CENPA	Olaparib	0.031329	0.498896	1058
CENPA	PAC-1	-0.02679	0.565977	1058
CENPA	PD-0325901	0.168974	6.1E-06	1058
CENPA	PD-0332991	0.047455	0.390446	1058
CENPA	PD-173074	0.004404	0.989461	1058
CENPA	PF-4708671	0.053401	0.555034	1058
CENPA	PF-562271	0.059031	0.270822	1058
CENPA	PFI-1	0.085806	0.050324	1058
CENPA	PHA-665752	0.054146	0.714723	1058
CENPA	PHA-793887	-0.01592	0.68153	1058
CENPA	PI-103	-0.03545	0.354143	1058
CENPA	PIK-93	-0.05831	0.108142	1058
CENPA	PLX4720	0.081646	0.03936	1058
CENPA	Paclitaxel	0.02449	0.862917	1058
CENPA	Parthenolide	0.047961	0.658302	1058
CENPA	Pazopanib	0.057087	0.280638	1058
CENPA	Phenformin	-0.04285	0.268101	1058
CENPA	Pyrimethamine	-0.02729	0.836144	1058
CENPA	QL-VIII-58	0.015895	0.874672	1058
CENPA	QL-X-138	-0.05179	0.170603	1058
CENPA	QL-XI-92	-0.02231	0.575114	1058
CENPA	QL-XII-47	-7.1E-06	0.999885	1058
CENPA	QL-XII-61	-0.05109	0.462902	1058
CENPA	QS11	-0.0145	0.854417	1058
CENPA	RDEA119	0.207187	1.38E-09	1058
CENPA	RO-3306	0.035629	0.416123	1058
CENPA	Rapamycin	-0.05028	0.688205	1058
CENPA	Roscovitine	0.145171	0.242524	1058
CENPA	Ruxolitinib	-0.03957	0.451517	1058
CENPA	S-Trityl-L-cysteine	0.009282	0.933947	1058
CENPA	SB 216763	0.072373	0.131596	1058
CENPA	SB 505124	0.057433	0.40608	1058
CENPA	SB52334	0.00102	0.989081	1058
CENPA	SB590885	0.112111	0.010052	1058
CENPA	SGC0946	0.042956	0.424625	1058
CENPA	SL 0101-1	0.043604	0.571645	1058
CENPA	SN-38	0.021912	0.661936	1058
CENPA	SNX-2112	0.009229	0.826804	1058
CENPA	STF-62247	-0.01566	0.738043	1058

CENPA	Salubrinal	0.045046	0.621127	1058
CENPA	Saracatinib	-0.02319	0.810634	1058
CENPA	Shikonin	0.005377	0.938151	1058
CENPA	Sorafenib	0.064537	0.544081	1058
CENPA	Sunitinib	0.001081	0.99268	1058
CENPA	T0901317	0.010632	0.827102	1058
CENPA	TAE684	-0.00868	0.96026	1058
CENPA	TAK-715	-0.03673	0.338737	1058
CENPA	TG101348	-0.03488	0.358568	1058
CENPA	TGX221	0.123119	0.046849	1058
CENPA	THZ-2-102-1	-0.06435	0.080122	1058
CENPA	THZ-2-49	-0.02382	0.552276	1058
CENPA	TL-1-85	-0.02871	0.461782	1058
CENPA	TL-2-105	-0.04448	0.250178	1058
CENPA	TPCA-1	-0.05985	0.098834	1058
CENPA	TW 37	-0.05677	0.207429	1058
CENPA	Talazoparib	0.030625	0.526908	1058
CENPA	Tamoxifen	0.02991	0.815232	1058
CENPA	Temozolomide	0.016401	0.854098	1058
CENPA	Temsirolimus	-0.03693	0.478215	1058
CENPA	Thapsigargin	0.040806	0.537036	1058
CENPA	Tipifarnib	0.055114	0.368237	1058
CENPA	Tivozanib	-0.02553	0.695062	1058
CENPA	Trametinib	0.230108	3.58E-11	1058
CENPA	Tubastatin A	0.005945	0.885556	1058
CENPA	UNC0638	-0.01688	0.665632	1058
CENPA	UNC1215	0.08852	0.051712	1058
CENPA	VNLG/124	-0.00595	0.908046	1058
CENPA	VX-11e	0.119907	0.003494	1058
CENPA	VX-680	-0.01644	0.894027	1058
CENPA	VX-702	-0.00418	0.991734	1058
CENPA	Veliparib	0.003789	0.968997	1058
CENPA	Vinblastine	-0.02748	0.572421	1058
CENPA	Vinorelbine	0.001523	0.984367	1058
CENPA	Vorinostat	-0.11505	0.001568	1058
CENPA	WH-4-023	0.069751	0.369441	1058
CENPA	WZ-1-84	0.013065	0.8898	1058
CENPA	WZ3105	-0.05069	0.168707	1058
CENPA	XAV939	0.045337	0.258213	1058
CENPA	XL-184	0.030661	0.539179	1058
CENPA	XMD11-85h	0.063732	0.692408	1058
CENPA	XMD13-2	-0.00149	0.972957	1058
CENPA	XMD14-99	0.049565	0.205124	1058
CENPA	XMD15-27	0.012066	0.833801	1058
CENPA	XMD8-85	0.051907	0.547269	1058
CENPA	XMD8-92	0.050013	0.653138	1058
CENPA	Y-39983	-0.02074	0.618963	1058
CENPA	YK 4-279	0.059456	0.245767	1058
CENPA	YM155	-0.0448	0.389204	1058
CENPA	YM201636	-0.04599	0.228448	1058

CENPA	Z-LLNle-CHO	0.050568	0.52341	1058
CENPA	ZG-10	-0.02872	0.708491	1058
CENPA	ZM-447439	-0.02513	0.630029	1058
CENPA	ZSTK474	-0.03624	0.344782	1058
CENPA	Zibotentan	0.011013	0.983594	1058
CENPA	piperlongumine	0.048835	0.266344	1058
CENPA	rTRAIL	0.044061	0.547632	1058
CENPA	selumetinib	0.194228	1.68E-08	1058
CENPE	(5Z)-7-Oxozeaenol	0.146534	7.21E-05	1062
CENPE	17-AAG	0.150109	3.55E-05	1062
CENPE	5-Fluorouracil	-0.06222	0.093428	1062
CENPE	681640	0.00123	0.987848	1062
CENPE	A-443654	-0.04966	0.783548	1062
CENPE	A-770041	0.001622	0.989113	1062
CENPE	AC220	-0.04786	0.312485	1062
CENPE	AG-014699	0.017115	0.724015	1062
CENPE	AICAR	-0.13592	0.000347	1062
CENPE	AKT inhibitor VIII	0.057953	0.195173	1062
CENPE	AMG-706	0.02083	0.772192	1062
CENPE	AP-24534	-0.06969	0.086712	1062
CENPE	AR-42	-0.13365	0.000147	1062
CENPE	AS601245	0.06383	0.21063	1062
CENPE	AS605240	0.0087	0.870056	1062
CENPE	AT-7519	-0.17003	9.17E-07	1062
CENPE	ATRA	-0.04951	0.31364	1062
CENPE	AUY922	0.024712	0.678043	1062
CENPE	AZ628	0.163566	0.0108	1062
CENPE	AZD6482	0.01444	0.80641	1062
CENPE	AZD7762	-0.16996	5.78E-06	1062
CENPE	AZD8055	-0.1062	0.005808	1062
CENPE	Afatinib	0.130072	0.000248	1062
CENPE	Axitinib	-0.07688	0.106375	1062
CENPE	BAY 61-3606	-0.03571	0.410029	1062
CENPE	BEZ235	-0.00829	0.894126	1062
CENPE	BHG712	-0.06519	0.076236	1062
CENPE	BI-2536	-0.13916	0.111729	1062
CENPE	BIRB 0796	0.039565	0.457073	1062
CENPE	BIX02189	-0.11868	0.00088	1062
CENPE	BMS-509744	-0.00104	0.993524	1062
CENPE	BMS-536924	-0.01051	0.914458	1062
CENPE	BMS-708163	0.066831	0.093625	1062
CENPE	BMS-754807	0.047043	0.387876	1062
CENPE	BMS345541	-0.16381	2.48E-06	1062
CENPE	BX-795	-0.13094	0.001146	1062
CENPE	BX-912	-0.2045	2.11E-09	1062
CENPE	Belinostat	-0.10405	0.004477	1062
CENPE	Bexarotene	-0.0243	0.778438	1062
CENPE	Bicalutamide	-0.0079	0.888615	1062
CENPE	Bleomycin	0.068891	0.175731	1062
CENPE	Bleomycin (50 uM)	0.076119	0.036311	1062

CENPE	Bortezomib	0.013443	0.899067	1062
CENPE	Bosutinib	-0.03077	0.598907	1062
CENPE	Bryostatin 1	0.073273	0.127289	1062
CENPE	CAL-101	-0.10464	0.004261	1062
CENPE	CAY10603	-0.14277	4.59E-05	1062
CENPE	CCT007093	0.077921	0.061136	1062
CENPE	CCT018159	-0.03813	0.473595	1062
CENPE	CEP-701	-0.08757	0.027118	1062
CENPE	CGP-082996	0.005467	0.967659	1062
CENPE	CGP-60474	-0.04315	0.646054	1062
CENPE	CH5424802	-0.05496	0.314558	1062
CENPE	CHIR-99021	0.029785	0.467653	1062
CENPE	CI-1040	0.139312	0.000384	1062
CENPE	CMK	-0.01319	0.930506	1062
CENPE	CP466722	-0.1825	1.23E-07	1062
CENPE	CP724714	0.063797	0.200672	1062
CENPE	CUDC-101	-0.12097	0.000776	1062
CENPE	CX-5461	-0.14186	6.32E-05	1062
CENPE	Camptothecin	-0.1059	0.008635	1062
CENPE	Cetuximab	0.089941	0.022666	1062
CENPE	Cisplatin	-0.01269	0.835752	1062
CENPE	Crizotinib	-0.07841	0.528386	1062
CENPE	Cyclopamine	-0.04138	0.729336	1062
CENPE	Cytarabine	-0.04956	0.325848	1062
CENPE	DMOG	-0.08411	0.045591	1062
CENPE	Dabrafenib	0.140272	0.00026	1062
CENPE	Dasatinib	0.055495	0.425905	1062
CENPE	Docetaxel	0.057968	0.129605	1062
CENPE	Doxorubicin	0.001071	0.990515	1062
CENPE	EHT 1864	0.031685	0.661076	1062
CENPE	EKB-569	-0.04163	0.319936	1062
CENPE	EX-527	0.033141	0.769628	1062
CENPE	Elesclomol	-0.06254	0.153126	1062
CENPE	Embelin	0.012517	0.8741	1062
CENPE	Epothilone B	0.023409	0.682051	1062
CENPE	Erlotinib	0.151503	0.023812	1062
CENPE	Etoposide	-0.11069	0.008154	1062
CENPE	FH535	0.103535	0.014978	1062
CENPE	FK866	-0.17831	3.32E-07	1062
CENPE	FMK	-0.0043	0.962722	1062
CENPE	FR-180204	0.009805	0.887294	1062
CENPE	FTI-277	0.106338	0.007328	1062
CENPE	Foretinib	-0.11908	0.001555	1062
CENPE	GDC0449	-0.0009	0.995569	1062
CENPE	GDC0941	-0.06703	0.184332	1062
CENPE	GNF-2	-0.03077	0.913374	1062
CENPE	GSK-650394	-0.01122	0.913039	1062
CENPE	GSK1070916	-0.18657	9.85E-08	1062
CENPE	GSK1904529A	0.044023	0.346063	1062
CENPE	GSK2126458	-0.09941	0.006734	1062

CENPE	GSK269962A	-0.00824	0.902493	1062
CENPE	GSK429286A	-0.12117	0.001371	1062
CENPE	GSK690693	-0.15346	1.39E-05	1062
CENPE	GW 441756	-0.00493	0.992585	1062
CENPE	GW-2580	0.015347	0.984347	1062
CENPE	GW843682X	-0.15954	0.030156	1062
CENPE	Gefitinib	0.121892	0.001827	1062
CENPE	Gemcitabine	-0.06678	0.154314	1062
CENPE	Genentech Cpd 10	-0.16538	2.37E-06	1062
CENPE	HG-5-113-01	-0.08802	0.181451	1062
CENPE	HG-5-88-01	-0.01431	0.945218	1062
CENPE	HG-6-64-1	0.019363	0.730489	1062
CENPE	I-BET-762	-0.16893	8.05E-07	1062
CENPE	IOX2	-0.0123	0.868582	1062
CENPE	IPA-3	-0.10128	0.008058	1062
CENPE	Imatinib	-0.05357	0.73953	1062
CENPE	Ispinesib Mesylate	-0.13822	9.4E-05	1062
CENPE	JNJ-26854165	-0.01024	0.876502	1062
CENPE	JNK Inhibitor VIII	0.048646	0.262143	1062
CENPE	JNK-9L	0.048961	0.377098	1062
CENPE	JQ1	-0.00416	0.943407	1062
CENPE	JQ12	-0.00773	0.91071	1062
CENPE	JW-7-24-1	-0.15868	4.98E-06	1062
CENPE	JW-7-52-1	-0.01009	0.940319	1062
CENPE	KIN001-055	0.01019	0.911045	1062
CENPE	KIN001-102	-0.16455	1.95E-06	1062
CENPE	KIN001-135	0.082593	0.38789	1062
CENPE	KIN001-236	-0.09093	0.012905	1062
CENPE	KIN001-244	-0.11912	0.000894	1062
CENPE	KIN001-260	-0.11306	0.001526	1062
CENPE	KIN001-266	-0.03289	0.515905	1062
CENPE	KIN001-270	-0.16804	4.19E-06	1062
CENPE	KU-55933	-0.0761	0.147262	1062
CENPE	LAQ824	-0.06366	0.110206	1062
CENPE	LFM-A13	0.015287	0.801981	1062
CENPE	LY317615	-0.06128	0.153131	1062
CENPE	Lapatinib	0.173762	0.003351	1062
CENPE	Lenalidomide	-0.04404	0.550657	1062
CENPE	Linifanib	-0.01516	0.814155	1062
CENPE	Lisitinib	0.042871	0.509132	1062
CENPE	MG-132	0.021005	0.856472	1062
CENPE	MK-2206	-0.10244	0.026924	1062
CENPE	MLN4924	0.037146	0.534706	1062
CENPE	MP470	-0.07421	0.078418	1062
CENPE	MPS-1-IN-1	-0.07759	0.038491	1062
CENPE	MS-275	-0.14314	0.042763	1062
CENPE	Masitinib	-0.09662	0.008664	1062
CENPE	Methotrexate	-0.1549	1.76E-05	1062
CENPE	Midostaurin	-0.00139	0.979498	1062
CENPE	Mitomycin C	-0.02646	0.645431	1062

CENPE	NG-25	-0.13032	0.00021	1062
CENPE	NPK76-II-72-1	-0.22073	6.62E-11	1062
CENPE	NSC-207895	-0.11536	0.003919	1062
CENPE	NSC-87877	0.094074	0.0644	1062
CENPE	NU-7441	-0.02888	0.721804	1062
CENPE	Navitoclax	-0.18235	3.77E-07	1062
CENPE	Nilotinib	-0.09037	0.046915	1062
CENPE	Nutlin-3a (-)	0.079227	0.073641	1062
CENPE	OSI-027	-0.13823	9.01E-05	1062
CENPE	OSI-930	-0.06257	0.107618	1062
CENPE	OSU-03012	-0.01535	0.795285	1062
CENPE	Obatoclax Mesylate	0.031512	0.534111	1062
CENPE	Olaparib	-0.0045	0.934709	1062
CENPE	PAC-1	-0.08057	0.047895	1062
CENPE	PD-0325901	0.199683	6.83E-08	1062
CENPE	PD-0332991	0.015408	0.82358	1062
CENPE	PD-173074	-0.032	0.843473	1062
CENPE	PF-4708671	0.038895	0.705557	1062
CENPE	PF-562271	0.013168	0.848085	1062
CENPE	PFI-1	0.004945	0.937761	1062
CENPE	PHA-665752	0.041687	0.791756	1062
CENPE	PHA-793887	-0.15831	4.28E-06	1062
CENPE	PI-103	-0.14849	2.42E-05	1062
CENPE	PIK-93	-0.17842	2.02E-07	1062
CENPE	PLX4720	0.168407	3.11E-06	1062
CENPE	Paclitaxel	-0.04583	0.699591	1062
CENPE	Parthenolide	-0.05877	0.57397	1062
CENPE	Pazopanib	0.019052	0.76615	1062
CENPE	Phenformin	-0.0713	0.055861	1062
CENPE	Pyrimethamine	-0.04517	0.709125	1062
CENPE	QL-VIII-58	-0.02867	0.750536	1062
CENPE	QL-X-138	-0.16713	2.02E-06	1062
CENPE	QL-XI-92	-0.15751	6.81E-06	1062
CENPE	QL-XII-47	-0.11521	0.002272	1062
CENPE	QL-XII-61	-0.10093	0.09755	1062
CENPE	QS11	-0.06528	0.252289	1062
CENPE	RDEA119	0.241711	1.01E-12	1062
CENPE	RO-3306	0.031339	0.480524	1062
CENPE	Rapamycin	-0.04755	0.70686	1062
CENPE	Roscovitine	0.036203	0.856106	1062
CENPE	Ruxolitinib	-0.0739	0.107838	1062
CENPE	S-Trityl-L-cysteine	-0.09497	0.21361	1062
CENPE	SB 216763	0.043987	0.385282	1062
CENPE	SB 505124	0.039922	0.598579	1062
CENPE	SB52334	0.026071	0.626065	1062
CENPE	SB590885	0.189311	1.36E-06	1062
CENPE	SGC0946	0.017513	0.78512	1062
CENPE	SL 0101-1	0.042981	0.577088	1062
CENPE	SN-38	-0.03887	0.400769	1062
CENPE	SNX-2112	-0.13625	0.000118	1062

CENPE	STF-62247	-0.0812	0.038732	1062
CENPE	Salubrinal	-0.07626	0.356909	1062
CENPE	Saracatinib	-0.01858	0.851215	1062
CENPE	Shikonin	-0.04608	0.388943	1062
CENPE	Sorafenib	0.001538	0.992965	1062
CENPE	Sunitinib	-0.06985	0.351701	1062
CENPE	T0901317	-0.09807	0.011604	1062
CENPE	TAE684	-0.03013	0.824646	1062
CENPE	TAK-715	-0.12638	0.000361	1062
CENPE	TG101348	-0.17256	6.03E-07	1062
CENPE	TGX221	0.120956	0.051268	1062
CENPE	THZ-2-102-1	-0.15223	1.51E-05	1062
CENPE	THZ-2-49	-0.07351	0.047055	1062
CENPE	TL-1-85	-0.12332	0.00049	1062
CENPE	TL-2-105	-0.12858	0.00035	1062
CENPE	TPCA-1	-0.17102	6.68E-07	1062
CENPE	TW 37	-0.06297	0.153217	1062
CENPE	Talazoparib	-0.03953	0.397448	1062
CENPE	Tamoxifen	-0.02107	0.882152	1062
CENPE	Temozolomide	-0.02772	0.724117	1062
CENPE	Temsirolimus	-0.08826	0.04473	1062
CENPE	Thapsigargin	0.006222	0.946892	1062
CENPE	Tipifarnib	0.021323	0.786107	1062
CENPE	Tivozanib	-0.05882	0.270544	1062
CENPE	Trametinib	0.270673	0	1062
CENPE	Tubastatin A	-0.12429	0.000411	1062
CENPE	UNC0638	-0.09208	0.008753	1062
CENPE	UNC1215	0.052707	0.287928	1062
CENPE	VNLG/124	-0.06937	0.085404	1062
CENPE	VX-11e	0.111437	0.007181	1062
CENPE	VX-680	-0.10801	0.19583	1062
CENPE	VX-702	-0.03352	0.890442	1062
CENPE	Veliparib	0.013187	0.87665	1062
CENPE	Vinblastine	-0.10025	0.014567	1062
CENPE	Vinorelbine	-0.0725	0.14662	1062
CENPE	Vorinostat	-0.18569	2.03E-07	1062
CENPE	WH-4-023	0.036635	0.671712	1062
CENPE	WZ-1-84	0.061515	0.440274	1062
CENPE	WZ3105	-0.13722	8.44E-05	1062
CENPE	XAV939	0.019516	0.650832	1062
CENPE	XL-184	-0.06659	0.134655	1062
CENPE	XMD11-85h	0.044622	0.8045	1062
CENPE	XMD13-2	-0.13246	0.000165	1062
CENPE	XMD14-99	-0.06383	0.094726	1062
CENPE	XMD15-27	-0.07276	0.09905	1062
CENPE	XMD8-85	-0.00435	0.968163	1062
CENPE	XMD8-92	-0.00779	0.961679	1062
CENPE	Y-39983	-0.16913	1.76E-06	1062
CENPE	YK 4-279	0.016831	0.798576	1062
CENPE	YM155	-0.07844	0.090616	1062

CENPE	YM201636	-0.13881	9.14E-05	1062
CENPE	Z-LLNle-CHO	0.005357	0.95478	1062
CENPE	ZG-10	-0.13074	0.025296	1062
CENPE	ZM-447439	-0.09573	0.024251	1062
CENPE	ZSTK474	-0.14013	7.22E-05	1062
CENPE	Zibotentan	-0.00673	0.989444	1062
CENPE	piperlongumine	0.006268	0.910961	1062
CENPE	rTRAIL	0.034161	0.658458	1062
CENPE	selumetinib	0.270364	0	1062
CENPF	(5Z)-7-Oxozeaenol	0.132361	0.000382	1063
CENPF	17-AAG	0.177926	7.81E-07	1063
CENPF	5-Fluorouracil	0.007012	0.865844	1063
CENPF	681640	0.029559	0.64725	1063
CENPF	A-443654	-0.0363	0.860921	1063
CENPF	A-770041	0.01783	0.862662	1063
CENPF	AC220	-0.02916	0.578286	1063
CENPF	AG-014699	-0.00796	0.875865	1063
CENPF	AICAR	-0.03135	0.462918	1063
CENPF	AKT inhibitor VIII	0.053158	0.240144	1063
CENPF	AMG-706	0.014963	0.84104	1063
CENPF	AP-24534	-0.00923	0.855112	1063
CENPF	AR-42	-0.09844	0.005806	1063
CENPF	AS601245	0.09609	0.038167	1063
CENPF	AS605240	0.020026	0.683332	1063
CENPF	AT-7519	-0.06723	0.063818	1063
CENPF	ATRA	-0.00715	0.910038	1063
CENPF	AUY922	0.092642	0.041595	1063
CENPF	AZ628	0.135994	0.040617	1063
CENPF	AZD6482	0.042147	0.375882	1063
CENPF	AZD7762	-0.07476	0.061557	1063
CENPF	AZD8055	-0.00872	0.849953	1063
CENPF	Afatinib	0.092598	0.010913	1063
CENPF	Axitinib	-0.05121	0.321185	1063
CENPF	BAY 61-3606	0.006897	0.890478	1063
CENPF	BEZ235	0.080734	0.07168	1063
CENPF	BHG712	-0.01279	0.752229	1063
CENPF	BI-2536	-0.04958	0.655295	1063
CENPF	BIRB 0796	0.029431	0.595693	1063
CENPF	BIX02189	-0.05989	0.108387	1063
CENPF	BMS-509744	-0.01288	0.911052	1063
CENPF	BMS-536924	-0.01698	0.844701	1063
CENPF	BMS-708163	0.068147	0.086816	1063
CENPF	BMS-754807	0.028412	0.633002	1063
CENPF	BMS345541	-0.08679	0.015715	1063
CENPF	BX-795	-0.06348	0.15245	1063
CENPF	BX-912	-0.1455	2.82E-05	1063
CENPF	Belinostat	-0.06864	0.06735	1063
CENPF	Bexarotene	0.022936	0.793061	1063
CENPF	Bicalutamide	-0.01581	0.762443	1063
CENPF	Bleomycin	0.09297	0.051867	1063

CENPF	Bleomycin (50 uM)	0.100018	0.005291	1063
CENPF	Bortezomib	0.043878	0.614589	1063
CENPF	Bosutinib	0.014209	0.831049	1063
CENPF	Bryostatin 1	0.076704	0.108527	1063
CENPF	CAL-101	-0.05335	0.166989	1063
CENPF	CAY10603	-0.09352	0.00885	1063
CENPF	CCT007093	0.06928	0.100906	1063
CENPF	CCT018159	0.039752	0.451096	1063
CENPF	CEP-701	-0.02614	0.55789	1063
CENPF	CGP-082996	0.010502	0.935932	1063
CENPF	CGP-60474	0.024306	0.815661	1063
CENPF	CH5424802	-0.03814	0.531845	1063
CENPF	CHIR-99021	-0.01607	0.711208	1063
CENPF	CI-1040	0.150924	0.000107	1063
CENPF	CMK	0.042973	0.735279	1063
CENPF	CP466722	-0.11347	0.001325	1063
CENPF	CP724714	0.037393	0.505614	1063
CENPF	CUDC-101	-0.08131	0.026875	1063
CENPF	CX-5461	-0.10933	0.002378	1063
CENPF	Camptothecin	-0.05272	0.234298	1063
CENPF	Cetuximab	0.03133	0.470551	1063
CENPF	Cisplatin	-0.03419	0.511741	1063
CENPF	Crizotinib	-0.02873	0.869641	1063
CENPF	Cyclopamine	-0.00306	0.983342	1063
CENPF	Cytarabine	-0.0227	0.693842	1063
CENPF	DMOG	-0.03561	0.459509	1063
CENPF	Dabrafenib	0.126463	0.001127	1063
CENPF	Dasatinib	0.060916	0.37756	1063
CENPF	Docetaxel	0.092678	0.01307	1063
CENPF	Doxorubicin	0.036523	0.584888	1063
CENPF	EHT 1864	0.018438	0.824216	1063
CENPF	EKB-569	-0.00797	0.868746	1063
CENPF	EX-527	0.01845	0.883471	1063
CENPF	Elesclomol	-0.0478	0.292135	1063
CENPF	Embelin	0.039591	0.515235	1063
CENPF	Epothilone B	0.072919	0.129473	1063
CENPF	Erlotinib	0.084429	0.245947	1063
CENPF	Etoposide	-0.07021	0.114947	1063
CENPF	FH535	0.099121	0.020321	1063
CENPF	FK866	-0.12331	0.000527	1063
CENPF	FMK	0.009244	0.917655	1063
CENPF	FR-180204	-0.00899	0.897056	1063
CENPF	FTI-277	0.126296	0.001279	1063
CENPF	Foretinib	-0.07594	0.056092	1063
CENPF	GDC0449	0.02629	0.820792	1063
CENPF	GDC0941	-0.02771	0.660991	1063
CENPF	GNF-2	-0.0018	0.996256	1063
CENPF	GSK-650394	0.030504	0.703137	1063
CENPF	GSK1070916	-0.16266	4.1E-06	1063
CENPF	GSK1904529A	0.011019	0.842731	1063

CENPF	GSK2126458	-0.05879	0.12307	1063
CENPF	GSK269962A	0.029727	0.602499	1063
CENPF	GSK429286A	-0.11628	0.002228	1063
CENPF	GSK690693	-0.11997	0.000817	1063
CENPF	GW 441756	-0.05321	0.73804	1063
CENPF	GW-2580	0.008005	0.994893	1063
CENPF	GW843682X	-0.05399	0.560667	1063
CENPF	Gefitinib	0.094002	0.018845	1063
CENPF	Gemcitabine	-0.01259	0.832272	1063
CENPF	Genentech Cpd 10	-0.14875	2.46E-05	1063
CENPF	HG-5-113-01	-0.09772	0.132288	1063
CENPF	HG-5-88-01	-0.00419	0.987441	1063
CENPF	HG-6-64-1	0.059382	0.200644	1063
CENPF	I-BET-762	-0.08593	0.014629	1063
CENPF	IOX2	-0.01793	0.798944	1063
CENPF	IPA-3	-0.03678	0.376754	1063
CENPF	Imatinib	-0.02009	0.916249	1063
CENPF	Ispinesib Mesylate	-0.04068	0.289934	1063
CENPF	JNJ-26854165	0.034323	0.525778	1063
CENPF	JNK Inhibitor VIII	0.016476	0.730683	1063
CENPF	JNK-9L	0.067214	0.193363	1063
CENPF	JQ1	0.041171	0.34899	1063
CENPF	JQ12	0.009093	0.895379	1063
CENPF	JW-7-24-1	-0.10001	0.004872	1063
CENPF	JW-7-52-1	0.052749	0.615501	1063
CENPF	KIN001-055	0.011526	0.897951	1063
CENPF	KIN001-102	-0.10141	0.004078	1063
CENPF	KIN001-135	-0.01301	0.926698	1063
CENPF	KIN001-236	-0.0367	0.347408	1063
CENPF	KIN001-244	-0.07004	0.05866	1063
CENPF	KIN001-260	-0.04041	0.288741	1063
CENPF	KIN001-266	-0.02349	0.656524	1063
CENPF	KIN001-270	-0.11609	0.001896	1063
CENPF	KU-55933	-0.01085	0.895778	1063
CENPF	LAQ824	-0.04422	0.282546	1063
CENPF	LFM-A13	0.007065	0.914292	1063
CENPF	LY317615	-0.02806	0.554476	1063
CENPF	Lapatinib	0.135084	0.026356	1063
CENPF	Lenalidomide	0.000979	0.993173	1063
CENPF	Linifanib	0.00764	0.912587	1063
CENPF	Lisitinib	0.019109	0.80551	1063
CENPF	MG-132	0.043856	0.651814	1063
CENPF	MK-2206	-0.04879	0.34326	1063
CENPF	MLN4924	0.073558	0.168035	1063
CENPF	MP470	-0.07241	0.086486	1063
CENPF	MPS-1-IN-1	-0.05715	0.136408	1063
CENPF	MS-275	-0.10938	0.13977	1063
CENPF	Masitinib	-0.02648	0.518099	1063
CENPF	Methotrexate	-0.05323	0.164008	1063
CENPF	Midostaurin	0.016657	0.745886	1063

CENPF	Mitomycin C	0.003113	0.965361	1063
CENPF	NG-25	-0.06184	0.092215	1063
CENPF	NPK76-II-72-1	-0.1577	4.51E-06	1063
CENPF	NSC-207895	-0.13967	0.000395	1063
CENPF	NSC-87877	0.087434	0.087543	1063
CENPF	NU-7441	0.019468	0.831806	1063
CENPF	Navitoclax	-0.20227	1.47E-08	1063
CENPF	Nilotinib	-0.02268	0.701146	1063
CENPF	Nutlin-3a (-)	0.057067	0.222796	1063
CENPF	OSI-027	-0.07729	0.033267	1063
CENPF	OSI-930	0.010598	0.815523	1063
CENPF	OSU-03012	0.058624	0.219919	1063
CENPF	Obatoclax Mesylate	0.076789	0.083018	1063
CENPF	Olaparib	-0.02739	0.561847	1063
CENPF	PAC-1	-0.03705	0.406613	1063
CENPF	PD-0325901	0.186238	5.39E-07	1063
CENPF	PD-0332991	0.046881	0.397619	1063
CENPF	PD-173074	-0.00214	0.994718	1063
CENPF	PF-4708671	0.04651	0.618729	1063
CENPF	PF-562271	0.011551	0.867308	1063
CENPF	PFI-1	0.072288	0.107991	1063
CENPF	PHA-665752	0.033904	0.833639	1063
CENPF	PHA-793887	-0.06404	0.07411	1063
CENPF	PI-103	-0.08237	0.022969	1063
CENPF	PIK-93	-0.11132	0.001507	1063
CENPF	PLX4720	0.124625	0.000885	1063
CENPF	Paclitaxel	0.036866	0.767636	1063
CENPF	Parthenolide	-0.02101	0.86883	1063
CENPF	Pazopanib	0.004755	0.949838	1063
CENPF	Phenformin	0.000672	0.988825	1063
CENPF	Pyrimethamine	0.015769	0.912183	1063
CENPF	QL-VIII-58	0.027994	0.75747	1063
CENPF	QL-X-138	-0.11477	0.001407	1063
CENPF	QL-XI-92	-0.10725	0.002649	1063
CENPF	QL-XII-47	-0.06625	0.093395	1063
CENPF	QL-XII-61	-0.08262	0.190501	1063
CENPF	QS11	-0.03562	0.585056	1063
CENPF	RDEA119	0.224075	4.77E-11	1063
CENPF	RO-3306	0.034954	0.42612	1063
CENPF	Rapamycin	0.007465	0.969094	1063
CENPF	Roscovitine	0.063696	0.692948	1063
CENPF	Ruxolitinib	-0.0424	0.410694	1063
CENPF	S-Trityl-L-cysteine	0.000343	0.997858	1063
CENPF	SB 216763	0.03454	0.50856	1063
CENPF	SB 505124	0.05864	0.39312	1063
CENPF	SB52334	-0.02814	0.593278	1063
CENPF	SB590885	0.16902	2.44E-05	1063
CENPF	SGC0946	-0.0098	0.887459	1063
CENPF	SL 0101-1	0.035764	0.651423	1063
CENPF	SN-38	0.010285	0.851921	1063

CENPF	SNX-2112	-0.0247	0.533139	1063
CENPF	STF-62247	-0.03763	0.373901	1063
CENPF	Salubrinal	0.035025	0.710716	1063
CENPF	Saracatinib	-0.02266	0.815448	1063
CENPF	Shikonin	-0.01587	0.804869	1063
CENPF	Sorafenib	0.030454	0.814475	1063
CENPF	Sunitinib	0.001698	0.988346	1063
CENPF	T0901317	-0.05784	0.15774	1063
CENPF	TAE684	-0.0342	0.798162	1063
CENPF	TAK-715	-0.05417	0.145581	1063
CENPF	TG101348	-0.10179	0.004172	1063
CENPF	TGX221	0.127478	0.03904	1063
CENPF	THZ-2-102-1	-0.07955	0.028619	1063
CENPF	THZ-2-49	-0.00966	0.820123	1063
CENPF	TL-1-85	-0.04857	0.196304	1063
CENPF	TL-2-105	-0.08451	0.022006	1063
CENPF	TPCA-1	-0.10965	0.0018	1063
CENPF	TW 37	-0.05752	0.200472	1063
CENPF	Talazoparib	-0.05249	0.243014	1063
CENPF	Tamoxifen	0.040337	0.721944	1063
CENPF	Temozolomide	0.009385	0.923036	1063
CENPF	Temsirolimus	-0.00372	0.954697	1063
CENPF	Thapsigargin	0.036862	0.584928	1063
CENPF	Tipifarnib	0.060805	0.306993	1063
CENPF	Tivozanib	-0.0171	0.808284	1063
CENPF	Trametinib	0.251881	3.26E-13	1063
CENPF	Tubastatin A	-0.08116	0.023657	1063
CENPF	UNC0638	-0.05948	0.097961	1063
CENPF	UNC1215	0.017685	0.767468	1063
CENPF	VNLG/124	-0.02835	0.529522	1063
CENPF	VX-11e	0.127502	0.001711	1063
CENPF	VX-680	-0.07531	0.407777	1063
CENPF	VX-702	0.008927	0.979483	1063
CENPF	Veliparib	0.017925	0.825137	1063
CENPF	Vinblastine	0.005009	0.929895	1063
CENPF	Vinorelbine	-0.01155	0.869427	1063
CENPF	Vorinostat	-0.13635	0.000164	1063
CENPF	WH-4-023	0.054428	0.501861	1063
CENPF	WZ-1-84	0.048413	0.5561	1063
CENPF	WZ3105	-0.07463	0.038097	1063
CENPF	XAV939	0.018615	0.667205	1063
CENPF	XL-184	0.000444	0.994375	1063
CENPF	XMD11-85h	0.022616	0.913018	1063
CENPF	XMD13-2	-0.06656	0.067832	1063
CENPF	XMD14-99	-0.02942	0.473149	1063
CENPF	XMD15-27	-0.06296	0.162708	1063
CENPF	XMD8-85	0.069848	0.393413	1063
CENPF	XMD8-92	0.014299	0.925071	1063
CENPF	Y-39983	-0.12291	0.000657	1063
CENPF	YK 4-279	0.082449	0.088022	1063

CENPF	YM155	-0.02697	0.645417	1063
CENPF	YM201636	-0.1114	0.001923	1063
CENPF	Z-LLNle-CHO	0.092775	0.189792	1063
CENPF	ZG-10	-0.10894	0.071492	1063
CENPF	ZM-447439	-0.06278	0.165053	1063
CENPF	ZSTK474	-0.08964	0.013102	1063
CENPF	Zibotentan	0.00819	0.987731	1063
CENPF	piperlongumine	0.03524	0.444599	1063
CENPF	rTRAIL	0.047341	0.51486	1063
CENPF	selumetinib	0.241574	1.15E-12	1063
CENPN	(5Z)-7-Oxozeaenol	0.079304	0.04421	55839
CENPN	17-AAG	-0.03646	0.352256	55839
CENPN	5-Fluorouracil	0.095691	0.008202	55839
CENPN	681640	-0.0197	0.773224	55839
CENPN	A-443654	-0.04345	0.818869	55839
CENPN	A-770041	-0.10133	0.168636	55839
CENPN	AC220	0.063788	0.155528	55839
CENPN	AG-014699	0.031548	0.488741	55839
CENPN	AICAR	0.022961	0.599944	55839
CENPN	AKT inhibitor VIII	-0.03303	0.495292	55839
CENPN	AMG-706	0.003872	0.963335	55839
CENPN	AP-24534	0.021932	0.635013	55839
CENPN	AR-42	0.044675	0.230662	55839
CENPN	AS601245	-0.00833	0.908366	55839
CENPN	AS605240	0.028768	0.539106	55839
CENPN	AT-7519	-0.00808	0.842775	55839
CENPN	ATRA	0.055224	0.251828	55839
CENPN	AUY922	-0.01775	0.77941	55839
CENPN	AZ628	0.063131	0.410017	55839
CENPN	AZD6482	-0.0554	0.218197	55839
CENPN	AZD7762	-0.10005	0.010254	55839
CENPN	AZD8055	-0.03632	0.384426	55839
CENPN	Afatinib	0.025609	0.523741	55839
CENPN	Axitinib	-0.00179	0.981964	55839
CENPN	BAY 61-3606	0.031996	0.464622	55839
CENPN	BEZ235	-0.04548	0.358536	55839
CENPN	BHG712	0.020669	0.600289	55839
CENPN	BI-2536	-0.11561	0.199424	55839
CENPN	BIRB 0796	-0.02836	0.61189	55839
CENPN	BIX02189	0.00457	0.916125	55839
CENPN	BMS-509744	-0.01207	0.916827	55839
CENPN	BMS-536924	0.081592	0.130265	55839
CENPN	BMS-708163	0.011876	0.797616	55839
CENPN	BMS-754807	0.107133	0.021967	55839
CENPN	BMS345541	-0.00855	0.834468	55839
CENPN	BX-795	0.02666	0.602811	55839
CENPN	BX-912	0.033484	0.37323	55839
CENPN	Belinostat	0.037149	0.341655	55839
CENPN	Bexarotene	-0.01209	0.908639	55839
CENPN	Bicalutamide	-0.05542	0.208543	55839

CENPN	Bleomycin	-0.00435	0.954487	55839
CENPN	Bleomycin (50 uM)	0.024673	0.52778	55839
CENPN	Bortezomib	-0.06468	0.4197	55839
CENPN	Bosutinib	-0.08129	0.081489	55839
CENPN	Bryostatin 1	-0.01121	0.864513	55839
CENPN	CAL-101	-0.02708	0.509318	55839
CENPN	CAY10603	0.054496	0.138381	55839
CENPN	CCT007093	-0.02827	0.551674	55839
CENPN	CCT018159	-0.04087	0.435573	55839
CENPN	CEP-701	-0.03938	0.357261	55839
CENPN	CGP-082996	-0.13099	0.088085	55839
CENPN	CGP-60474	-0.0909	0.246818	55839
CENPN	CH5424802	-0.01183	0.879241	55839
CENPN	CHIR-99021	0.013826	0.753677	55839
CENPN	CI-1040	0.024076	0.623306	55839
CENPN	CMK	-0.09044	0.381698	55839
CENPN	CP466722	0.010554	0.795553	55839
CENPN	CP724714	0.03304	0.566192	55839
CENPN	CUDC-101	0.026054	0.512454	55839
CENPN	CX-5461	0.061131	0.101168	55839
CENPN	Camptothecin	-0.01507	0.776203	55839
CENPN	Cetuximab	0.002539	0.957049	55839
CENPN	Cisplatin	-0.01499	0.802386	55839
CENPN	Crizotinib	-0.02705	0.877395	55839
CENPN	Cyclopamine	-0.05024	0.657922	55839
CENPN	Cytarabine	-0.03756	0.47876	55839
CENPN	DMOG	-0.06426	0.142943	55839
CENPN	Dabrafenib	0.03168	0.494664	55839
CENPN	Dasatinib	-0.03434	0.642013	55839
CENPN	Docetaxel	-0.07587	0.044275	55839
CENPN	Doxorubicin	-0.01487	0.85094	55839
CENPN	EHT 1864	-0.03252	0.650339	55839
CENPN	EKB-569	-0.04074	0.331793	55839
CENPN	EX-527	0.016707	0.897134	55839
CENPN	Elesclomol	-0.13639	0.000713	55839
CENPN	Embelin	-0.04769	0.409356	55839
CENPN	Epothilone B	-0.03744	0.481737	55839
CENPN	Erlotinib	0.039008	0.618435	55839
CENPN	Etoposide	-0.02989	0.569818	55839
CENPN	FH535	-0.02749	0.609382	55839
CENPN	FK866	0.049961	0.183723	55839
CENPN	FMK	-0.00125	0.989377	55839
CENPN	FR-180204	0.028588	0.621952	55839
CENPN	FTI-277	0.030099	0.496726	55839
CENPN	Foretinib	0.044499	0.296485	55839
CENPN	GDC0449	0.00351	0.984049	55839
CENPN	GDC0941	-0.01024	0.886829	55839
CENPN	GNF-2	0.031308	0.911624	55839
CENPN	GSK-650394	-0.00268	0.978737	55839
CENPN	GSK1070916	0.005267	0.903732	55839

CENPN	GSK1904529A	-0.01966	0.70753	55839
CENPN	GSK2126458	0.015963	0.704487	55839
CENPN	GSK269962A	0.04602	0.370503	55839
CENPN	GSK429286A	0.024371	0.596707	55839
CENPN	GSK690693	-0.04338	0.260895	55839
CENPN	GW 441756	-0.06548	0.650281	55839
CENPN	GW-2580	0.080671	0.420979	55839
CENPN	GW843682X	-0.11549	0.135503	55839
CENPN	Gefitinib	0.01743	0.701199	55839
CENPN	Gemcitabine	-0.05832	0.222977	55839
CENPN	Genentech Cpd 10	0.003313	0.940514	55839
CENPN	HG-5-113-01	0.074904	0.271648	55839
CENPN	HG-5-88-01	0.12546	0.161434	55839
CENPN	HG-6-64-1	-0.00662	0.916132	55839
CENPN	I-BET-762	0.004471	0.912013	55839
CENPN	IOX2	-0.05842	0.290865	55839
CENPN	IPA-3	-0.01652	0.712237	55839
CENPN	Imatinib	0.035343	0.842171	55839
CENPN	Ispinesib Mesylate	0.004927	0.906531	55839
CENPN	JNJ-26854165	-0.05163	0.301215	55839
CENPN	JNK Inhibitor VIII	-0.06629	0.11597	55839
CENPN	JNK-9L	-0.00496	0.951454	55839
CENPN	JQ1	-0.01338	0.79419	55839
CENPN	JQ12	0.004794	0.94684	55839
CENPN	JW-7-24-1	0.005873	0.887901	55839
CENPN	JW-7-52-1	-0.07237	0.451762	55839
CENPN	KIN001-055	-0.00453	0.962381	55839
CENPN	KIN001-102	-0.02354	0.543503	55839
CENPN	KIN001-135	0.085281	0.370617	55839
CENPN	KIN001-236	0.0243	0.54685	55839
CENPN	KIN001-244	0.017845	0.663592	55839
CENPN	KIN001-260	0.021902	0.583225	55839
CENPN	KIN001-266	0.024613	0.639891	55839
CENPN	KIN001-270	-0.02111	0.632121	55839
CENPN	KU-55933	-0.02069	0.777725	55839
CENPN	LAQ824	-0.00815	0.861561	55839
CENPN	LFM-A13	-0.02091	0.720103	55839
CENPN	LY317615	-0.03915	0.389146	55839
CENPN	Lapatinib	0.080064	0.2086	55839
CENPN	Lenalidomide	-0.05012	0.478327	55839
CENPN	Linifanib	0.088565	0.045335	55839
CENPN	Lisitinib	0.099713	0.046898	55839
CENPN	MG-132	-0.0842	0.310488	55839
CENPN	MK-2206	-0.09449	0.043235	55839
CENPN	MLN4924	0.027492	0.660471	55839
CENPN	MP470	0.007493	0.887006	55839
CENPN	MPS-1-IN-1	0.055576	0.148175	55839
CENPN	MS-275	-0.10317	0.167362	55839
CENPN	Masitinib	0.058349	0.126552	55839
CENPN	Methotrexate	0.020233	0.618086	55839

CENPN	Midostaurin	0.014283	0.784155	55839
CENPN	Mitomycin C	-0.02285	0.694903	55839
CENPN	NG-25	0.046468	0.215199	55839
CENPN	NPK76-II-72-1	-0.00169	0.967524	55839
CENPN	NSC-207895	-0.02068	0.688216	55839
CENPN	NSC-87877	0.0508	0.354834	55839
CENPN	NU-7441	-0.04308	0.552539	55839
CENPN	Navitoclax	0.04866	0.208682	55839
CENPN	Nilotinib	0.031766	0.568608	55839
CENPN	Nutlin-3a (-)	0.09502	0.027524	55839
CENPN	OSI-027	0.035033	0.359234	55839
CENPN	OSI-930	0.054853	0.163329	55839
CENPN	OSU-03012	-0.02166	0.702334	55839
CENPN	Obatoclax Mesylate	-0.02593	0.621082	55839
CENPN	Olaparib	0.072394	0.086967	55839
CENPN	PAC-1	-0.01995	0.681332	55839
CENPN	PD-0325901	0.038161	0.366853	55839
CENPN	PD-0332991	0.025296	0.69025	55839
CENPN	PD-173074	-0.00523	0.986979	55839
CENPN	PF-4708671	-0.01189	0.932611	55839
CENPN	PF-562271	0.012059	0.861835	55839
CENPN	PFI-1	-0.00181	0.979095	55839
CENPN	PHA-665752	0.046205	0.760498	55839
CENPN	PHA-793887	0.017587	0.648675	55839
CENPN	PI-103	0.021987	0.578217	55839
CENPN	PIK-93	-0.01645	0.67548	55839
CENPN	PLX4720	0.0524	0.208554	55839
CENPN	Paclitaxel	-0.05718	0.615667	55839
CENPN	Parthenolide	-0.06299	0.54054	55839
CENPN	Pazopanib	0.026532	0.663119	55839
CENPN	Phenformin	0.024995	0.534956	55839
CENPN	Pyrimethamine	-0.05837	0.617772	55839
CENPN	QL-VIII-58	0.01851	0.850149	55839
CENPN	QL-X-138	0.015005	0.714024	55839
CENPN	QL-XI-92	0.0085	0.839613	55839
CENPN	QL-XII-47	0.01956	0.663526	55839
CENPN	QL-XII-61	0.030598	0.688583	55839
CENPN	QS11	-0.00969	0.907892	55839
CENPN	RDEA119	0.078329	0.032438	55839
CENPN	RO-3306	-0.06681	0.110356	55839
CENPN	Rapamycin	-0.08419	0.429413	55839
CENPN	Roscovitine	0.048535	0.785714	55839
CENPN	Ruxolitinib	-0.00324	0.962418	55839
CENPN	S-Trityl-L-cysteine	-0.05927	0.480839	55839
CENPN	SB 216763	0.057142	0.247139	55839
CENPN	SB 505124	0.032899	0.681248	55839
CENPN	SB52334	0.081674	0.054306	55839
CENPN	SB590885	0.050836	0.305269	55839
CENPN	SGC0946	-0.00443	0.952718	55839
CENPN	SL 0101-1	0.064516	0.376123	55839

CENPN	SN-38	0.045108	0.317195	55839
CENPN	SNX-2112	0.017686	0.663324	55839
CENPN	STF-62247	-0.01196	0.802565	55839
CENPN	Salubrinal	-0.00865	0.938494	55839
CENPN	Saracatinib	-0.04307	0.630856	55839
CENPN	Shikonin	-0.01085	0.868284	55839
CENPN	Sorafenib	0.093245	0.31543	55839
CENPN	Sunitinib	-0.00804	0.938878	55839
CENPN	T0901317	-0.03005	0.494707	55839
CENPN	TAE684	-0.00504	0.975947	55839
CENPN	TAK-715	-0.01377	0.73492	55839
CENPN	TG101348	0.026467	0.494026	55839
CENPN	TGX221	0.03375	0.633236	55839
CENPN	THZ-2-102-1	0.011724	0.774285	55839
CENPN	THZ-2-49	-0.02171	0.590542	55839
CENPN	TL-1-85	0.033884	0.379935	55839
CENPN	TL-2-105	0.050826	0.184284	55839
CENPN	TPCA-1	0.017168	0.661275	55839
CENPN	TW 37	-0.09103	0.027324	55839
CENPN	Talazoparib	0.053077	0.236741	55839
CENPN	Tamoxifen	-0.01115	0.945806	55839
CENPN	Temozolomide	0.011704	0.902246	55839
CENPN	Temsirolimus	-0.1088	0.010355	55839
CENPN	Thapsigargin	-0.02572	0.731225	55839
CENPN	Tipifarnib	-0.02507	0.738609	55839
CENPN	Tivozanib	0.01146	0.880432	55839
CENPN	Trametinib	0.076325	0.041057	55839
CENPN	Tubastatin A	0.076298	0.034003	55839
CENPN	UNC0638	0.024609	0.517281	55839
CENPN	UNC1215	0.076331	0.100484	55839
CENPN	VNLG/124	0.062618	0.124878	55839
CENPN	VX-11e	0.041384	0.39797	55839
CENPN	VX-680	-0.00394	0.978822	55839
CENPN	VX-702	0.022045	0.932405	55839
CENPN	Veliparib	-0.01662	0.840051	55839
CENPN	Vinblastine	-0.09682	0.018809	55839
CENPN	Vinorelbine	-0.0653	0.201619	55839
CENPN	Vorinostat	-0.03892	0.310214	55839
CENPN	WH-4-023	-0.00564	0.956704	55839
CENPN	WZ-1-84	0.00169	0.987072	55839
CENPN	WZ3105	0.013527	0.732616	55839
CENPN	XAV939	-0.05361	0.175938	55839
CENPN	XL-184	0.082763	0.053078	55839
CENPN	XMD11-85h	0.059802	0.716164	55839
CENPN	XMD13-2	0.00623	0.881566	55839
CENPN	XMD14-99	0.060513	0.114737	55839
CENPN	XMD15-27	-0.01519	0.786078	55839
CENPN	XMD8-85	-0.03098	0.74664	55839
CENPN	XMD8-92	0.009244	0.953892	55839
CENPN	Y-39983	-0.01991	0.633869	55839

CENPN	YK 4-279	-0.01208	0.861387	55839
CENPN	YM155	-0.01247	0.850582	55839
CENPN	YM201636	-0.00828	0.847268	55839
CENPN	Z-LLNle-CHO	-0.07906	0.277395	55839
CENPN	ZG-10	0.057873	0.3915	55839
CENPN	ZM-447439	0.010284	0.856921	55839
CENPN	ZSTK474	-0.02281	0.565391	55839
CENPN	Zibotentan	-0.04043	0.861754	55839
CENPN	piperlongumine	-0.0116	0.827607	55839
CENPN	rTRAIL	0.0025	0.980419	55839
CENPN	selumetinib	0.07991	0.029821	55839
DEPDC1B	(5Z)-7-Oxozeaenol	0.074763	0.060005	55789
DEPDC1B	17-AAG	0.196233	4.39E-08	55789
DEPDC1B	5-Fluorouracil	-0.07883	0.031189	55789
DEPDC1B	681640	0.007796	0.916493	55789
DEPDC1B	A-443654	-0.06008	0.71583	55789
DEPDC1B	A-770041	0.003888	0.973265	55789
DEPDC1B	AC220	-0.0145	0.80778	55789
DEPDC1B	AG-014699	0.084935	0.038652	55789
DEPDC1B	AICAR	-0.10529	0.006319	55789
DEPDC1B	AKT inhibitor VIII	0.105906	0.011216	55789
DEPDC1B	AMG-706	0.088919	0.079681	55789
DEPDC1B	AP-24534	-0.05958	0.149906	55789
DEPDC1B	AR-42	-0.13725	9.45E-05	55789
DEPDC1B	AS601245	0.020935	0.739515	55789
DEPDC1B	AS605240	-0.07181	0.081766	55789
DEPDC1B	AT-7519	-0.14173	5.01E-05	55789
DEPDC1B	ATRA	-0.0243	0.661937	55789
DEPDC1B	AUY922	0.020631	0.736724	55789
DEPDC1B	AZ628	0.090921	0.20346	55789
DEPDC1B	AZD6482	0.0552	0.219932	55789
DEPDC1B	AZD7762	-0.12233	0.001427	55789
DEPDC1B	AZD8055	-0.04906	0.228284	55789
DEPDC1B	Afatinib	0.011433	0.787687	55789
DEPDC1B	Axitinib	-0.02732	0.644137	55789
DEPDC1B	BAY 61-3606	-0.00907	0.851778	55789
DEPDC1B	BEZ235	0.056144	0.23771	55789
DEPDC1B	BHG712	-0.12517	0.000403	55789
DEPDC1B	BI-2536	-0.12161	0.171631	55789
DEPDC1B	BIRB 0796	0.07913	0.099771	55789
DEPDC1B	BIX02189	-0.11575	0.001198	55789
DEPDC1B	BMS-509744	0.048377	0.616823	55789
DEPDC1B	BMS-536924	0.01006	0.91892	55789
DEPDC1B	BMS-708163	0.133205	0.000482	55789
DEPDC1B	BMS-754807	0.064917	0.207463	55789
DEPDC1B	BMS345541	-0.14068	5.94E-05	55789
DEPDC1B	BX-795	-0.03658	0.451113	55789
DEPDC1B	BX-912	-0.13351	0.00013	55789
DEPDC1B	Belinostat	-0.11505	0.001566	55789
DEPDC1B	Bexarotene	0.023625	0.78574	55789

DEPDC1B	Bicalutamide	0.064123	0.138685	55789
DEPDC1B	Bleomycin	0.046965	0.396526	55789
DEPDC1B	Bleomycin (50 uM)	0.099978	0.005309	55789
DEPDC1B	Bortezomib	0.048259	0.572453	55789
DEPDC1B	Bosutinib	-0.08352	0.072388	55789
DEPDC1B	Bryostatin 1	0.084444	0.072835	55789
DEPDC1B	CAL-101	-0.11547	0.00151	55789
DEPDC1B	CAY10603	-0.12202	0.000545	55789
DEPDC1B	CCT007093	0.147597	0.000194	55789
DEPDC1B	CCT018159	0.027552	0.625156	55789
DEPDC1B	CEP-701	-0.05769	0.161275	55789
DEPDC1B	CGP-082996	-0.00969	0.941885	55789
DEPDC1B	CGP-60474	0.013878	0.902374	55789
DEPDC1B	CH5424802	-0.05131	0.357892	55789
DEPDC1B	CHIR-99021	0.064894	0.089637	55789
DEPDC1B	CI-1040	0.051295	0.247933	55789
DEPDC1B	CMK	0.052283	0.66773	55789
DEPDC1B	CP466722	-0.1442	3.67E-05	55789
DEPDC1B	CP724714	0.086666	0.059223	55789
DEPDC1B	CUDC-101	-0.14957	2.61E-05	55789
DEPDC1B	CX-5461	-0.13698	0.000116	55789
DEPDC1B	Camptothecin	-0.05407	0.22066	55789
DEPDC1B	Cetuximab	-0.04565	0.276125	55789
DEPDC1B	Cisplatin	0.049598	0.316511	55789
DEPDC1B	Crizotinib	-0.05332	0.722811	55789
DEPDC1B	Cyclopamine	0.028481	0.82503	55789
DEPDC1B	Cytarabine	-0.01079	0.863509	55789
DEPDC1B	DMOG	-0.12027	0.002697	55789
DEPDC1B	Dabrafenib	0.054939	0.203193	55789
DEPDC1B	Dasatinib	0.048212	0.496183	55789
DEPDC1B	Docetaxel	0.140167	0.000136	55789
DEPDC1B	Doxorubicin	-0.01213	0.8829	55789
DEPDC1B	EHT 1864	0.089629	0.098292	55789
DEPDC1B	EKB-569	-0.10719	0.004753	55789
DEPDC1B	EX-527	0.006255	0.965674	55789
DEPDC1B	Elesclomol	0.041594	0.3675	55789
DEPDC1B	Embelin	-0.00236	0.978928	55789
DEPDC1B	Epothilone B	-0.00445	0.945327	55789
DEPDC1B	Erlotinib	0.150564	0.024856	55789
DEPDC1B	Etoposide	-0.10366	0.014134	55789
DEPDC1B	FH535	0.091618	0.033814	55789
DEPDC1B	FK866	-0.11473	0.00131	55789
DEPDC1B	FMK	-0.06057	0.334622	55789
DEPDC1B	FR-180204	-0.06051	0.216823	55789
DEPDC1B	FTI-277	0.157274	4.78E-05	55789
DEPDC1B	Foretinib	-0.06722	0.095913	55789
DEPDC1B	GDC0449	0.018256	0.884822	55789
DEPDC1B	GDC0941	-0.0265	0.67658	55789
DEPDC1B	GNF-2	-0.05977	0.792872	55789
DEPDC1B	GSK-650394	-0.01784	0.845765	55789

DEPDC1B	GSK1070916	-0.16696	2.18E-06	55789
DEPDC1B	GSK1904529A	0.036555	0.44535	55789
DEPDC1B	GSK2126458	-0.1048	0.004166	55789
DEPDC1B	GSK269962A	0.059936	0.212491	55789
DEPDC1B	GSK429286A	-0.06623	0.103167	55789
DEPDC1B	GSK690693	-0.14308	5.48E-05	55789
DEPDC1B	GW 441756	-0.01267	0.973676	55789
DEPDC1B	GW-2580	-0.0319	0.941501	55789
DEPDC1B	GW843682X	-0.15131	0.040082	55789
DEPDC1B	Gefitinib	0.011798	0.801212	55789
DEPDC1B	Gemcitabine	-0.10031	0.022298	55789
DEPDC1B	Genentech Cpd 10	-0.11391	0.001506	55789
DEPDC1B	HG-5-113-01	-0.06609	0.34456	55789
DEPDC1B	HG-5-88-01	-0.00199	0.994588	55789
DEPDC1B	HG-6-64-1	0.015663	0.788713	55789
DEPDC1B	I-BET-762	-0.14229	3.65E-05	55789
DEPDC1B	IOX2	0.017578	0.803602	55789
DEPDC1B	IPA-3	-0.07291	0.06302	55789
DEPDC1B	Imatinib	-0.06976	0.618736	55789
DEPDC1B	Ispinesib Mesylate	-0.09009	0.01322	55789
DEPDC1B	JNJ-26854165	0.002024	0.97925	55789
DEPDC1B	JNK Inhibitor VIII	0.052895	0.219329	55789
DEPDC1B	JNK-9L	-0.00429	0.958015	55789
DEPDC1B	JQ1	-0.01377	0.788039	55789
DEPDC1B	JQ12	-0.01964	0.755549	55789
DEPDC1B	JW-7-24-1	-0.11358	0.001303	55789
DEPDC1B	JW-7-52-1	0.009939	0.941531	55789
DEPDC1B	KIN001-055	-0.04843	0.421144	55789
DEPDC1B	KIN001-102	-0.12019	0.000607	55789
DEPDC1B	KIN001-135	0.158627	0.061518	55789
DEPDC1B	KIN001-236	-0.08101	0.027857	55789
DEPDC1B	KIN001-244	-0.10902	0.00247	55789
DEPDC1B	KIN001-260	-0.09217	0.01053	55789
DEPDC1B	KIN001-266	-0.07979	0.07312	55789
DEPDC1B	KIN001-270	-0.09421	0.013202	55789
DEPDC1B	KU-55933	-0.07074	0.187109	55789
DEPDC1B	LAQ824	-0.11547	0.002323	55789
DEPDC1B	LFM-A13	0.084348	0.071056	55789
DEPDC1B	LY317615	-0.07101	0.091514	55789
DEPDC1B	Lapatinib	0.200856	0.000556	55789
DEPDC1B	Lenalidomide	-0.03094	0.71235	55789
DEPDC1B	Linifanib	-0.01004	0.880341	55789
DEPDC1B	Lisitinib	0.038649	0.562754	55789
DEPDC1B	MG-132	0.056613	0.537757	55789
DEPDC1B	MK-2206	-0.06129	0.2174	55789
DEPDC1B	MLN4924	0.069887	0.193735	55789
DEPDC1B	MP470	-0.03588	0.428624	55789
DEPDC1B	MPS-1-IN-1	-0.09171	0.013248	55789
DEPDC1B	MS-275	-0.16847	0.015007	55789
DEPDC1B	Masitinib	-0.08993	0.014955	55789

DEPDC1B	Methotrexate	-0.13364	0.000234	55789
DEPDC1B	Midostaurin	0.047519	0.295291	55789
DEPDC1B	Mitomycin C	-0.06673	0.164874	55789
DEPDC1B	NG-25	-0.12715	0.000304	55789
DEPDC1B	NPK76-II-72-1	-0.2059	1.3E-09	55789
DEPDC1B	NSC-207895	-0.17937	4.28E-06	55789
DEPDC1B	NSC-87877	0.078396	0.129298	55789
DEPDC1B	NU-7441	-0.0151	0.876433	55789
DEPDC1B	Navitoclax	-0.23838	1.59E-11	55789
DEPDC1B	Nilotinib	-0.08974	0.048632	55789
DEPDC1B	Nutlin-3a (-)	0.056546	0.227754	55789
DEPDC1B	OSI-027	-0.12202	0.000589	55789
DEPDC1B	OSI-930	-0.07109	0.064972	55789
DEPDC1B	OSU-03012	-0.02455	0.658456	55789
DEPDC1B	Obatoclax Mesylate	-0.03507	0.480936	55789
DEPDC1B	Olaparib	0.050314	0.253043	55789
DEPDC1B	PAC-1	-0.11238	0.00434	55789
DEPDC1B	PD-0325901	0.112038	0.003613	55789
DEPDC1B	PD-0332991	0.024972	0.694854	55789
DEPDC1B	PD-173074	0.069919	0.534852	55789
DEPDC1B	PF-4708671	0.096432	0.23058	55789
DEPDC1B	PF-562271	0.021176	0.740659	55789
DEPDC1B	PFI-1	-0.00747	0.903816	55789
DEPDC1B	PHA-665752	0.136633	0.19743	55789
DEPDC1B	PHA-793887	-0.14793	1.85E-05	55789
DEPDC1B	PI-103	-0.13551	0.000124	55789
DEPDC1B	PIK-93	-0.1361	9.05E-05	55789
DEPDC1B	PLX4720	0.083691	0.034167	55789
DEPDC1B	Paclitaxel	-0.03017	0.819375	55789
DEPDC1B	Parthenolide	-0.04248	0.705472	55789
DEPDC1B	Pazopanib	0.065619	0.201981	55789
DEPDC1B	Phenformin	-0.06486	0.083896	55789
DEPDC1B	Pyrimethamine	-0.01327	0.930354	55789
DEPDC1B	QL-VIII-58	0.060138	0.430802	55789
DEPDC1B	QL-X-138	-0.12258	0.000617	55789
DEPDC1B	QL-XI-92	-0.12706	0.000329	55789
DEPDC1B	QL-XII-47	-0.08149	0.035827	55789
DEPDC1B	QL-XII-61	-0.0856	0.172594	55789
DEPDC1B	QS11	-0.02771	0.68866	55789
DEPDC1B	RDEA119	0.131001	0.000197	55789
DEPDC1B	RO-3306	0.090716	0.026411	55789
DEPDC1B	Rapamycin	-0.04114	0.759006	55789
DEPDC1B	Roscovitine	0.073181	0.637078	55789
DEPDC1B	Ruxolitinib	-0.05605	0.249934	55789
DEPDC1B	S-Trityl-L-cysteine	-0.09763	0.198048	55789
DEPDC1B	SB 216763	0.088394	0.06078	55789
DEPDC1B	SB 505124	0.08477	0.169408	55789
DEPDC1B	SB52334	0.018502	0.7444	55789
DEPDC1B	SB590885	0.101572	0.022067	55789
DEPDC1B	SGC0946	0.024747	0.682745	55789

DEPDC1B	SL 0101-1	0.06266	0.393454	55789
DEPDC1B	SN-38	0.005291	0.926194	55789
DEPDC1B	SNX-2112	-0.06548	0.076803	55789
DEPDC1B	STF-62247	-0.116	0.002471	55789
DEPDC1B	Salubrinal	-0.01346	0.901848	55789
DEPDC1B	Saracatinib	-0.00683	0.950654	55789
DEPDC1B	Shikonin	-0.06224	0.215236	55789
DEPDC1B	Sorafenib	0.018214	0.893858	55789
DEPDC1B	Sunitinib	-0.04911	0.541169	55789
DEPDC1B	T0901317	-0.0585	0.152551	55789
DEPDC1B	TAE684	-0.01906	0.900007	55789
DEPDC1B	TAK-715	-0.14157	5.92E-05	55789
DEPDC1B	TG101348	-0.13256	0.000155	55789
DEPDC1B	TGX221	0.190992	0.001203	55789
DEPDC1B	THZ-2-102-1	-0.11805	0.000913	55789
DEPDC1B	THZ-2-49	-0.04649	0.222831	55789
DEPDC1B	TL-1-85	-0.12301	0.000507	55789
DEPDC1B	TL-2-105	-0.12346	0.000618	55789
DEPDC1B	TPCA-1	-0.18518	6.6E-08	55789
DEPDC1B	TW 37	-0.02639	0.607752	55789
DEPDC1B	Talazoparib	0.029505	0.544405	55789
DEPDC1B	Tamoxifen	0.052022	0.623686	55789
DEPDC1B	Temozolomide	0.003265	0.975783	55789
DEPDC1B	Temsirolimus	-0.00267	0.968282	55789
DEPDC1B	Thapsigargin	-0.00744	0.935754	55789
DEPDC1B	Tipifarnib	-0.00824	0.927749	55789
DEPDC1B	Tivozanib	-0.05102	0.360466	55789
DEPDC1B	Trametinib	0.128096	0.000372	55789
DEPDC1B	Tubastatin A	-0.13384	0.000135	55789
DEPDC1B	UNC0638	-0.06752	0.058663	55789
DEPDC1B	UNC1215	0.096531	0.03188	55789
DEPDC1B	VNLG/124	-0.03514	0.42344	55789
DEPDC1B	VX-11e	0.020697	0.707037	55789
DEPDC1B	VX-680	-0.14079	0.072503	55789
DEPDC1B	VX-702	-0.00863	0.979793	55789
DEPDC1B	Veliparib	0.028752	0.693755	55789
DEPDC1B	Vinblastine	-0.01104	0.837713	55789
DEPDC1B	Vinorelbine	-0.04528	0.411868	55789
DEPDC1B	Vorinostat	-0.17409	1.18E-06	55789
DEPDC1B	WH-4-023	0.080333	0.289316	55789
DEPDC1B	WZ-1-84	0.121759	0.084749	55789
DEPDC1B	WZ3105	-0.10503	0.002972	55789
DEPDC1B	XAV939	0.020531	0.632803	55789
DEPDC1B	XL-184	-0.02044	0.702131	55789
DEPDC1B	XMD11-85h	-0.00501	0.983275	55789
DEPDC1B	XMD13-2	-0.08441	0.018989	55789
DEPDC1B	XMD14-99	-0.05766	0.134892	55789
DEPDC1B	XMD15-27	-0.03951	0.419395	55789
DEPDC1B	XMD8-85	0.057052	0.502809	55789
DEPDC1B	XMD8-92	-0.06193	0.557221	55789

DEPDC1B	Y-39983	-0.08806	0.01706	55789
DEPDC1B	YK 4-279	0.045481	0.402513	55789
DEPDC1B	YM155	-0.10595	0.015944	55789
DEPDC1B	YM201636	-0.10333	0.004173	55789
DEPDC1B	Z-LLNle-CHO	-0.02693	0.755893	55789
DEPDC1B	ZG-10	-0.05606	0.408794	55789
DEPDC1B	ZM-447439	-0.09434	0.026746	55789
DEPDC1B	ZSTK474	-0.13594	0.000121	55789
DEPDC1B	Zibotentan	0.012886	0.976549	55789
DEPDC1B	piperlongumine	0.051016	0.242478	55789
DEPDC1B	rTRAIL	0.093163	0.119637	55789
DEPDC1B	selumetinib	0.112304	0.001693	55789
DLGAP5	(5Z)-7-Oxozeaenol	0.149947	4.7E-05	9787
DLGAP5	17-AAG	0.094821	0.01062	9787
DLGAP5	5-Fluorouracil	0.045946	0.224524	9787
DLGAP5	681640	-0.03279	0.607084	9787
DLGAP5	A-443654	-0.07713	0.616739	9787
DLGAP5	A-770041	-0.03746	0.683897	9787
DLGAP5	AC220	0.01546	0.793436	9787
DLGAP5	AG-014699	0.032115	0.480542	9787
DLGAP5	AICAR	-0.05826	0.149585	9787
DLGAP5	AKT inhibitor VIII	0.058531	0.190183	9787
DLGAP5	AMG-706	0.013426	0.861017	9787
DLGAP5	AP-24534	0.002168	0.966814	9787
DLGAP5	AR-42	-0.00882	0.827118	9787
DLGAP5	AS601245	0.047546	0.377274	9787
DLGAP5	AS605240	0.061415	0.144378	9787
DLGAP5	AT-7519	-0.08675	0.015279	9787
DLGAP5	ATRA	0.006756	0.915121	9787
DLGAP5	AUY922	0.025799	0.661707	9787
DLGAP5	AZ628	0.186667	0.002956	9787
DLGAP5	AZD6482	0.03407	0.493446	9787
DLGAP5	AZD7762	-0.08184	0.039182	9787
DLGAP5	AZD8055	-0.02997	0.48135	9787
DLGAP5	Afatinib	0.047184	0.216684	9787
DLGAP5	Axitinib	-0.03591	0.519175	9787
DLGAP5	BAY 61-3606	0.019673	0.667346	9787
DLGAP5	BEZ235	0.003739	0.951857	9787
DLGAP5	BHG712	0.044562	0.237641	9787
DLGAP5	BI-2536	-0.11178	0.217495	9787
DLGAP5	BIRB 0796	0.001634	0.980666	9787
DLGAP5	BIX02189	-0.00481	0.911378	9787
DLGAP5	BMS-509744	0.007984	0.945207	9787
DLGAP5	BMS-536924	0.035692	0.62168	9787
DLGAP5	BMS-708163	0.059058	0.142325	9787
DLGAP5	BMS-754807	0.058236	0.266456	9787
DLGAP5	BMS345541	-0.06806	0.061504	9787
DLGAP5	BX-795	-0.05192	0.256563	9787
DLGAP5	BX-912	-0.07333	0.041306	9787
DLGAP5	Belinostat	-0.01072	0.79897	9787

DLGAP5	Bexarotene	-0.00394	0.974064	9787
DLGAP5	Bicalutamide	-0.01193	0.824787	9787
DLGAP5	Bleomycin	0.054548	0.308536	9787
DLGAP5	Bleomycin (50 uM)	0.019131	0.629462	9787
DLGAP5	Bortezomib	0.044216	0.611797	9787
DLGAP5	Bosutinib	-0.04649	0.384592	9787
DLGAP5	Bryostatin 1	0.065674	0.181285	9787
DLGAP5	CAL-101	-0.04182	0.288832	9787
DLGAP5	CAY10603	-0.01721	0.662757	9787
DLGAP5	CCT007093	0.06158	0.150625	9787
DLGAP5	CCT018159	-0.03865	0.466429	9787
DLGAP5	CEP-701	-0.0031	0.951045	9787
DLGAP5	CGP-082996	-0.03296	0.770367	9787
DLGAP5	CGP-60474	-0.05857	0.506127	9787
DLGAP5	CH5424802	-0.01866	0.797029	9787
DLGAP5	CHIR-99021	0.045574	0.247822	9787
DLGAP5	CI-1040	0.144294	0.000224	9787
DLGAP5	CMK	0.02337	0.870076	9787
DLGAP5	CP466722	-0.07419	0.040131	9787
DLGAP5	CP724714	0.037943	0.499229	9787
DLGAP5	CUDC-101	-0.03443	0.377029	9787
DLGAP5	CX-5461	-0.0408	0.289026	9787
DLGAP5	Camptothecin	-0.06077	0.16127	9787
DLGAP5	Cetuximab	0.030918	0.477079	9787
DLGAP5	Cisplatin	-0.04387	0.385013	9787
DLGAP5	Crizotinib	-0.02154	0.905903	9787
DLGAP5	Cyclopamine	0.015088	0.916722	9787
DLGAP5	Cytarabine	-0.01689	0.778946	9787
DLGAP5	DMOG	-0.0637	0.147047	9787
DLGAP5	Dabrafenib	0.163936	1.42E-05	9787
DLGAP5	Dasatinib	0.018238	0.815719	9787
DLGAP5	Docetaxel	-0.01255	0.760346	9787
DLGAP5	Doxorubicin	-0.00273	0.975314	9787
DLGAP5	EHT 1864	0.070468	0.223121	9787
DLGAP5	EKB-569	-0.03016	0.487139	9787
DLGAP5	EX-527	0.008933	0.949178	9787
DLGAP5	Elesclomol	-0.08067	0.057749	9787
DLGAP5	Embelin	0.008181	0.920365	9787
DLGAP5	Epothilone B	-0.0001	0.998333	9787
DLGAP5	Erlotinib	0.087095	0.229895	9787
DLGAP5	Etoposide	-0.08157	0.06163	9787
DLGAP5	FH535	0.08557	0.050183	9787
DLGAP5	FK866	-0.07607	0.037483	9787
DLGAP5	FMK	0.042109	0.541881	9787
DLGAP5	FR-180204	0.059632	0.224611	9787
DLGAP5	FTI-277	0.116277	0.003193	9787
DLGAP5	Foretinib	-0.01617	0.738517	9787
DLGAP5	GDC0449	0.028602	0.798914	9787
DLGAP5	GDC0941	-0.04899	0.37035	9787
DLGAP5	GNF-2	0.012803	0.970663	9787

DLGAP5	GSK-650394	-0.00147	0.988102	9787
DLGAP5	GSK1070916	-0.07385	0.046296	9787
DLGAP5	GSK1904529A	0.040133	0.396542	9787
DLGAP5	GSK2126458	-0.02919	0.468716	9787
DLGAP5	GSK269962A	0.016901	0.789982	9787
DLGAP5	GSK429286A	-0.04295	0.316643	9787
DLGAP5	GSK690693	-0.09694	0.007667	9787
DLGAP5	GW 441756	0.001014	0.99674	9787
DLGAP5	GW-2580	0.033946	0.937849	9787
DLGAP5	GW843682X	-0.12997	0.085767	9787
DLGAP5	Gefitinib	0.034224	0.430202	9787
DLGAP5	Gemcitabine	-0.05884	0.218014	9787
DLGAP5	Genentech Cpd 10	-0.0537	0.156356	9787
DLGAP5	HG-5-113-01	-0.02415	0.776057	9787
DLGAP5	HG-5-88-01	0.035975	0.830258	9787
DLGAP5	HG-6-64-1	0.047828	0.321444	9787
DLGAP5	I-BET-762	-0.0186	0.627314	9787
DLGAP5	IOX2	-0.02939	0.638484	9787
DLGAP5	IPA-3	-0.025	0.560887	9787
DLGAP5	Imatinib	-0.00313	0.989907	9787
DLGAP5	Ispinesib Mesylate	-0.01031	0.805051	9787
DLGAP5	JNJ-26854165	-0.00426	0.952875	9787
DLGAP5	JNK Inhibitor VIII	-0.01529	0.750405	9787
DLGAP5	JNK-9L	0.054138	0.316048	9787
DLGAP5	JQ1	0.053821	0.206167	9787
DLGAP5	JQ12	0.053743	0.31047	9787
DLGAP5	JW-7-24-1	-0.0315	0.410288	9787
DLGAP5	JW-7-52-1	0.005868	0.967179	9787
DLGAP5	KIN001-055	0.000437	0.996408	9787
DLGAP5	KIN001-102	-0.05714	0.11739	9787
DLGAP5	KIN001-135	0.09436	0.309468	9787
DLGAP5	KIN001-236	0.035071	0.370817	9787
DLGAP5	KIN001-244	-0.02775	0.484521	9787
DLGAP5	KIN001-260	-0.01467	0.720053	9787
DLGAP5	KIN001-266	0.031927	0.530153	9787
DLGAP5	KIN001-270	-0.08829	0.020855	9787
DLGAP5	KU-55933	-0.06611	0.224499	9787
DLGAP5	LAQ824	-0.00842	0.856763	9787
DLGAP5	LFM-A13	0.02685	0.63524	9787
DLGAP5	LY317615	-0.00818	0.880244	9787
DLGAP5	Lapatinib	0.087136	0.168274	9787
DLGAP5	Lenalidomide	-0.04461	0.543618	9787
DLGAP5	Linifanib	0.033194	0.548342	9787
DLGAP5	Lisitinib	0.048424	0.437796	9787
DLGAP5	MG-132	0.037315	0.711986	9787
DLGAP5	MK-2206	-0.09118	0.052214	9787
DLGAP5	MLN4924	0.054053	0.334173	9787
DLGAP5	MP470	-0.02544	0.586608	9787
DLGAP5	MPS-1-IN-1	0.013731	0.749854	9787
DLGAP5	MS-275	-0.09368	0.221336	9787

DLGAP5	Masitinib	0.018429	0.662233	9787
DLGAP5	Methotrexate	-0.01596	0.698476	9787
DLGAP5	Midostaurin	0.010508	0.842782	9787
DLGAP5	Mitomycin C	-0.00383	0.95717	9787
DLGAP5	NG-25	-0.01443	0.719771	9787
DLGAP5	NPK76-II-72-1	-0.08745	0.013347	9787
DLGAP5	NSC-207895	-0.12148	0.002268	9787
DLGAP5	NSC-87877	0.103204	0.040356	9787
DLGAP5	NU-7441	-0.04081	0.579682	9787
DLGAP5	Navitoclax	-0.10965	0.002912	9787
DLGAP5	Nilotinib	-0.02789	0.625107	9787
DLGAP5	Nutlin-3a (-)	0.141897	0.000547	9787
DLGAP5	OSI-027	-0.04077	0.281247	9787
DLGAP5	OSI-930	0.009768	0.830715	9787
DLGAP5	OSU-03012	0.007771	0.905321	9787
DLGAP5	Obatoclax Mesylate	0.048159	0.312288	9787
DLGAP5	Olaparib	-0.01853	0.70777	9787
DLGAP5	PAC-1	-0.00095	0.986071	9787
DLGAP5	PD-0325901	0.18156	1.06E-06	9787
DLGAP5	PD-0332991	0.058958	0.2625	9787
DLGAP5	PD-173074	-0.01267	0.952297	9787
DLGAP5	PF-4708671	0.078727	0.349857	9787
DLGAP5	PF-562271	0.013367	0.845052	9787
DLGAP5	PFI-1	0.055345	0.238508	9787
DLGAP5	PHA-665752	0.08102	0.553314	9787
DLGAP5	PHA-793887	-0.05252	0.147829	9787
DLGAP5	PI-103	-0.06339	0.084393	9787
DLGAP5	PIK-93	-0.0574	0.114099	9787
DLGAP5	PLX4720	0.156669	1.74E-05	9787
DLGAP5	Paclitaxel	-0.0509	0.66279	9787
DLGAP5	Parthenolide	0.00247	0.987129	9787
DLGAP5	Pazopanib	0.035299	0.54514	9787
DLGAP5	Phenformin	-0.01522	0.715266	9787
DLGAP5	Pyrimethamine	0.000208	0.999099	9787
DLGAP5	QL-VIII-58	-0.01416	0.891392	9787
DLGAP5	QL-X-138	-0.07262	0.049044	9787
DLGAP5	QL-XI-92	-0.03553	0.355578	9787
DLGAP5	QL-XII-47	-0.046	0.262844	9787
DLGAP5	QL-XII-61	-0.06699	0.309755	9787
DLGAP5	QS11	-0.03904	0.543063	9787
DLGAP5	RDEA119	0.220402	1.02E-10	9787
DLGAP5	RO-3306	0.005203	0.917945	9787
DLGAP5	Rapamycin	-0.09528	0.358297	9787
DLGAP5	Roscovitine	0.043365	0.817195	9787
DLGAP5	Ruxolitinib	-0.00165	0.980597	9787
DLGAP5	S-Trityl-L-cysteine	-0.04554	0.607206	9787
DLGAP5	SB 216763	0.050964	0.307136	9787
DLGAP5	SB 505124	0.038927	0.610744	9787
DLGAP5	SB52334	0.052687	0.256492	9787
DLGAP5	SB590885	0.185102	2.58E-06	9787

DLGAP5	SGC0946	0.03606	0.518122	9787
DLGAP5	SL 0101-1	0.0745	0.309023	9787
DLGAP5	SN-38	-0.01146	0.834049	9787
DLGAP5	SNX-2112	-0.01572	0.701064	9787
DLGAP5	STF-62247	0.018647	0.686783	9787
DLGAP5	Salubrinal	0.017107	0.872538	9787
DLGAP5	Saracatinib	-0.02723	0.773979	9787
DLGAP5	Shikonin	-0.01589	0.804616	9787
DLGAP5	Sorafenib	0.051174	0.657139	9787
DLGAP5	Sunitinib	-0.00591	0.956897	9787
DLGAP5	T0901317	-0.02035	0.657147	9787
DLGAP5	TAE684	-0.04005	0.751928	9787
DLGAP5	TAK-715	-0.04859	0.195442	9787
DLGAP5	TG101348	-0.03678	0.331073	9787
DLGAP5	TGX221	0.120239	0.052844	9787
DLGAP5	THZ-2-102-1	-0.03539	0.355548	9787
DLGAP5	THZ-2-49	0.012392	0.76673	9787
DLGAP5	TL-1-85	-0.00843	0.840953	9787
DLGAP5	TL-2-105	-0.02842	0.477513	9787
DLGAP5	TPCA-1	-0.04415	0.232438	9787
DLGAP5	TW 37	-0.07736	0.068467	9787
DLGAP5	Talazoparib	-0.00255	0.964788	9787
DLGAP5	Tamoxifen	0.008422	0.960839	9787
DLGAP5	Temozolomide	0.005602	0.956503	9787
DLGAP5	Temsirolimus	-0.06642	0.153434	9787
DLGAP5	Thapsigargin	0.012281	0.889901	9787
DLGAP5	Tipifarnib	0.026586	0.720596	9787
DLGAP5	Tivozanib	-0.02201	0.744089	9787
DLGAP5	Trametinib	0.228459	5.02E-11	9787
DLGAP5	Tubastatin A	0.002299	0.956782	9787
DLGAP5	UNC0638	0.01033	0.797346	9787
DLGAP5	UNC1215	0.070415	0.134422	9787
DLGAP5	VNLG/124	0.03417	0.437704	9787
DLGAP5	VX-11e	0.158087	7.24E-05	9787
DLGAP5	VX-680	-0.05023	0.611857	9787
DLGAP5	VX-702	0.003584	0.992323	9787
DLGAP5	Veliparib	-0.00869	0.923081	9787
DLGAP5	Vinblastine	-0.036	0.441809	9787
DLGAP5	Vinorelbine	-0.031	0.605364	9787
DLGAP5	Vorinostat	-0.09728	0.007952	9787
DLGAP5	WH-4-023	0.051994	0.523836	9787
DLGAP5	WZ-1-84	0.011711	0.902123	9787
DLGAP5	WZ3105	-0.04264	0.251255	9787
DLGAP5	XAV939	-0.01523	0.72867	9787
DLGAP5	XL-184	0.018106	0.738596	9787
DLGAP5	XMD11-85h	0.066062	0.678107	9787
DLGAP5	XMD13-2	-0.01389	0.731959	9787
DLGAP5	XMD14-99	0.056253	0.145804	9787
DLGAP5	XMD15-27	-0.00761	0.900191	9787
DLGAP5	XMD8-85	0.065711	0.429567	9787

DLGAP5	XMD8-92	0.008234	0.960252	9787
DLGAP5	Y-39983	-0.05431	0.156568	9787
DLGAP5	YK 4-279	0.038442	0.494793	9787
DLGAP5	YM155	-0.07993	0.083549	9787
DLGAP5	YM201636	-0.03268	0.403948	9787
DLGAP5	Z-LLNle-CHO	0.041556	0.610078	9787
DLGAP5	ZG-10	-0.04688	0.503967	9787
DLGAP5	ZM-447439	-0.02039	0.704434	9787
DLGAP5	ZSTK474	-0.05567	0.134705	9787
DLGAP5	Zibotentan	-0.00578	0.990687	9787
DLGAP5	piperlongumine	-0.00233	0.97034	9787
DLGAP5	rTRAIL	0.041357	0.577323	9787
DLGAP5	selumetinib	0.244062	6.5E-13	9787
DTL	(5Z)-7-Oxozeaenol	0.093762	0.015169	51514
DTL	17-AAG	0.197078	3.83E-08	51514
DTL	5-Fluorouracil	-0.14946	2.34E-05	51514
DTL	681640	0.060187	0.302685	51514
DTL	A-443654	-0.01628	0.946056	51514
DTL	A-770041	0.097435	0.190326	51514
DTL	AC220	-0.06595	0.140053	51514
DTL	AG-014699	0.046191	0.287101	51514
DTL	AICAR	-0.13041	0.000613	51514
DTL	AKT inhibitor VIII	0.10932	0.008648	51514
DTL	AMG-706	0.055946	0.324957	51514
DTL	AP-24534	-0.05542	0.183801	51514
DTL	AR-42	-0.19399	2.04E-08	51514
DTL	AS601245	0.115766	0.009762	51514
DTL	AS605240	-0.07316	0.075327	51514
DTL	AT-7519	-0.15274	1.15E-05	51514
DTL	ATRA	-0.09633	0.027265	51514
DTL	AUY922	0.064243	0.187692	51514
DTL	AZ628	0.043592	0.597532	51514
DTL	AZD6482	0.067833	0.115689	51514
DTL	AZD7762	-0.16352	1.35E-05	51514
DTL	AZD8055	-0.11886	0.001865	51514
DTL	Afatinib	0.157582	6.71E-06	51514
DTL	Axitinib	-0.11409	0.01046	51514
DTL	BAY 61-3606	-0.03814	0.376493	51514
DTL	BEZ235	0.076895	0.089187	51514
DTL	BHG712	-0.13263	0.00017	51514
DTL	BI-2536	-0.06778	0.502413	51514
DTL	BIRB 0796	0.078444	0.10288	51514
DTL	BIX02189	-0.14474	4.12E-05	51514
DTL	BMS-509744	0.033817	0.740011	51514
DTL	BMS-536924	-0.02799	0.719366	51514
DTL	BMS-708163	0.111828	0.003706	51514
DTL	BMS-754807	0.028475	0.632323	51514
DTL	BMS345541	-0.20792	1.46E-09	51514
DTL	BX-795	-0.10406	0.011845	51514
DTL	BX-912	-0.22467	3.75E-11	51514

DTL	Belinostat	-0.15885	8.86E-06	51514
DTL	Bexarotene	0.01618	0.869021	51514
DTL	Bicalutamide	0.074633	0.079889	51514
DTL	Bleomycin	0.07886	0.111798	51514
DTL	Bleomycin (50 uM)	0.160488	4.83E-06	51514
DTL	Bortezomib	0.067405	0.395853	51514
DTL	Bosutinib	0.0023	0.975441	51514
DTL	Bryostatin 1	0.108368	0.017611	51514
DTL	CAL-101	-0.10352	0.004722	51514
DTL	CAY10603	-0.1865	6.85E-08	51514
DTL	CCT007093	0.1211	0.002459	51514
DTL	CCT018159	0.020831	0.724506	51514
DTL	CEP-701	-0.13373	0.000469	51514
DTL	CGP-082996	0.051203	0.611109	51514
DTL	CGP-60474	0.063189	0.465834	51514
DTL	CH5424802	-0.07944	0.109507	51514
DTL	CHIR-99021	0.083922	0.02488	51514
DTL	CI-1040	0.106326	0.008446	51514
DTL	CMK	0.064923	0.568265	51514
DTL	CP466722	-0.20345	2.99E-09	51514
DTL	CP724714	0.102717	0.019504	51514
DTL	CUDC-101	-0.16479	3.22E-06	51514
DTL	CX-5461	-0.18011	2.63E-07	51514
DTL	Camptothecin	-0.10259	0.011315	51514
DTL	Cetuximab	0.12387	0.001232	51514
DTL	Cisplatin	-0.02223	0.692222	51514
DTL	Crizotinib	-0.06148	0.663199	51514
DTL	Cyclopamine	0.038104	0.757168	51514
DTL	Cytarabine	-0.05	0.321001	51514
DTL	DMOG	-0.06841	0.11522	51514
DTL	Dabrafenib	0.071327	0.087268	51514
DTL	Dasatinib	0.141711	0.023165	51514
DTL	Docetaxel	0.155968	2.03E-05	51514
DTL	Doxorubicin	0.026347	0.713747	51514
DTL	EHT 1864	0.010282	0.91177	51514
DTL	EKB-569	-0.06746	0.089085	51514
DTL	EX-527	-0.00482	0.973856	51514
DTL	Elesclomol	0.007881	0.884387	51514
DTL	Embelin	0.034838	0.578809	51514
DTL	Epothilone B	0.077744	0.102107	51514
DTL	Erlotinib	0.199466	0.001866	51514
DTL	Etoposide	-0.08648	0.045461	51514
DTL	FH535	0.110674	0.008609	51514
DTL	FK866	-0.21166	9.75E-10	51514
DTL	FMK	-0.03309	0.648618	51514
DTL	FR-180204	-0.04079	0.443998	51514
DTL	FTI-277	0.173516	6.33E-06	51514
DTL	Foretinib	-0.11894	0.001575	51514
DTL	GDC0449	0.036641	0.718442	51514
DTL	GDC0941	-0.02188	0.740598	51514

DTL	GNF-2	-0.05058	0.836716	51514
DTL	GSK-650394	0.018425	0.840465	51514
DTL	GSK1070916	-0.2455	1.08E-12	51514
DTL	GSK1904529A	0.079031	0.070435	51514
DTL	GSK2126458	-0.13073	0.000289	51514
DTL	GSK269962A	0.014195	0.826372	51514
DTL	GSK429286A	-0.16273	1.04E-05	51514
DTL	GSK690693	-0.15961	5.95E-06	51514
DTL	GW 441756	-0.02857	0.897484	51514
DTL	GW-2580	0.003119	0.9952	51514
DTL	GW843682X	-0.09653	0.233532	51514
DTL	Gefitinib	0.155272	5.01E-05	51514
DTL	Gemcitabine	-0.06179	0.192467	51514
DTL	Genentech Cpd 10	-0.19765	1.21E-08	51514
DTL	HG-5-113-01	-0.07548	0.267324	51514
DTL	HG-5-88-01	0.006589	0.978314	51514
DTL	HG-6-64-1	0.065966	0.146187	51514
DTL	I-BET-762	-0.23	9.7E-12	51514
DTL	IOX2	0.071026	0.181588	51514
DTL	IPA-3	-0.08015	0.039494	51514
DTL	Imatinib	-0.10572	0.32209	51514
DTL	Ispinesib Mesylate	-0.13835	9.25E-05	51514
DTL	JNJ-26854165	0.067158	0.157233	51514
DTL	JNK Inhibitor VIII	0.087429	0.033698	51514
DTL	JNK-9L	0.026621	0.673749	51514
DTL	JQ1	-0.03064	0.500805	51514
DTL	JQ12	-0.04227	0.444027	51514
DTL	JW-7-24-1	-0.18535	7.61E-08	51514
DTL	JW-7-52-1	0.046435	0.665953	51514
DTL	KIN001-055	0.051473	0.384459	51514
DTL	KIN001-102	-0.20212	3.58E-09	51514
DTL	KIN001-135	0.097386	0.291691	51514
DTL	KIN001-236	-0.09677	0.007897	51514
DTL	KIN001-244	-0.15503	1.16E-05	51514
DTL	KIN001-260	-0.13629	0.000115	51514
DTL	KIN001-266	-0.04716	0.324335	51514
DTL	KIN001-270	-0.1115	0.002935	51514
DTL	KU-55933	-0.04393	0.471624	51514
DTL	LAQ824	-0.12501	0.000918	51514
DTL	LFM-A13	0.081712	0.081776	51514
DTL	LY317615	-0.09324	0.021855	51514
DTL	Lapatinib	0.225346	8.61E-05	51514
DTL	Lenalidomide	-0.04979	0.482004	51514
DTL	Linifanib	-0.03676	0.498638	51514
DTL	Lisitinib	0.001819	0.984226	51514
DTL	MG-132	0.074183	0.38763	51514
DTL	MK-2206	-0.08343	0.079779	51514
DTL	MLN4924	0.045722	0.427271	51514
DTL	MP470	-0.07091	0.093657	51514
DTL	MPS-1-IN-1	-0.14949	3.18E-05	51514

DTL	MS-275	-0.20084	0.00345	51514
DTL	Masitinib	-0.15707	1.08E-05	51514
DTL	Methotrexate	-0.19194	7.55E-08	51514
DTL	Midostaurin	0.061037	0.163248	51514
DTL	Mitomycin C	-0.0717	0.13037	51514
DTL	NG-25	-0.15834	5.37E-06	51514
DTL	NPK76-II-72-1	-0.27238	3.57E-16	51514
DTL	NSC-207895	-0.13464	0.000657	51514
DTL	NSC-87877	0.10537	0.036244	51514
DTL	NU-7441	0.004867	0.966103	51514
DTL	Navitoclax	-0.2824	7.16E-16	51514
DTL	Nilotinib	-0.10415	0.018672	51514
DTL	Nutlin-3a (-)	0.01002	0.869039	51514
DTL	OSI-027	-0.15872	5.97E-06	51514
DTL	OSI-930	-0.0897	0.017673	51514
DTL	OSU-03012	0.013254	0.825452	51514
DTL	Obatoclax Mesylate	0.024987	0.636641	51514
DTL	Olaparib	0.028575	0.541873	51514
DTL	PAC-1	-0.10116	0.010987	51514
DTL	PD-0325901	0.177511	1.87E-06	51514
DTL	PD-0332991	-0.00057	0.994269	51514
DTL	PD-173074	-0.03714	0.813209	51514
DTL	PF-4708671	0.061687	0.487255	51514
DTL	PF-562271	0.038307	0.508735	51514
DTL	PFI-1	0.012109	0.838655	51514
DTL	PHA-665752	0.098201	0.44294	51514
DTL	PHA-793887	-0.18608	5.24E-08	51514
DTL	PI-103	-0.16799	1.55E-06	51514
DTL	PIK-93	-0.21702	1.71E-10	51514
DTL	PLX4720	0.068096	0.092723	51514
DTL	Paclitaxel	0.048813	0.676653	51514
DTL	Parthenolide	-0.02019	0.87487	51514
DTL	Pazopanib	0.043076	0.440288	51514
DTL	Phenformin	-0.09454	0.009943	51514
DTL	Pyrimethamine	-0.06866	0.546819	51514
DTL	QL-VIII-58	0.067718	0.358678	51514
DTL	QL-X-138	-0.17332	7.9E-07	51514
DTL	QL-XI-92	-0.18938	4.64E-08	51514
DTL	QL-XII-47	-0.12113	0.001278	51514
DTL	QL-XII-61	-0.14373	0.012691	51514
DTL	QS11	-0.03664	0.572962	51514
DTL	RDEA119	0.193809	1.65E-08	51514
DTL	RO-3306	0.109142	0.006933	51514
DTL	Rapamycin	0.021687	0.892295	51514
DTL	Roscovitine	0.075751	0.626279	51514
DTL	Ruxolitinib	-0.08314	0.063864	51514
DTL	S-Trityl-L-cysteine	-0.05741	0.498012	51514
DTL	SB 216763	0.099703	0.032215	51514
DTL	SB 505124	0.049037	0.490986	51514
DTL	SB52334	-0.04096	0.402428	51514

DTL	SB590885	0.097566	0.029078	51514
DTL	SGC0946	0.067389	0.163671	51514
DTL	SL 0101-1	0.070573	0.331565	51514
DTL	SN-38	-0.00848	0.879381	51514
DTL	SNX-2112	-0.15784	6.93E-06	51514
DTL	STF-62247	-0.071	0.073751	51514
DTL	Salubrinal	0.003723	0.973502	51514
DTL	Saracatinib	0.046452	0.600989	51514
DTL	Shikonin	-0.02622	0.657289	51514
DTL	Sorafenib	0.008699	0.952661	51514
DTL	Sunitinib	-0.05351	0.500827	51514
DTL	T0901317	-0.07672	0.053642	51514
DTL	TAE684	-0.00756	0.964524	51514
DTL	TAK-715	-0.13594	0.000118	51514
DTL	TG101348	-0.21874	1.47E-10	51514
DTL	TGX221	0.22076	0.000136	51514
DTL	THZ-2-102-1	-0.20404	3.93E-09	51514
DTL	THZ-2-49	-0.10666	0.003264	51514
DTL	TL-1-85	-0.1665	1.72E-06	51514
DTL	TL-2-105	-0.16606	2.75E-06	51514
DTL	TPCA-1	-0.21545	2.39E-10	51514
DTL	TW 37	-0.05717	0.203837	51514
DTL	Talazoparib	-0.02494	0.616798	51514
DTL	Tamoxifen	0.079596	0.373983	51514
DTL	Temozolomide	-0.02497	0.756783	51514
DTL	Temsirolimus	-0.00893	0.883814	51514
DTL	Thapsigargin	0.027755	0.706389	51514
DTL	Tipifarnib	0.042079	0.527988	51514
DTL	Tivozanib	-0.07622	0.122854	51514
DTL	Trametinib	0.248389	7.11E-13	51514
DTL	Tubastatin A	-0.16802	1.26E-06	51514
DTL	UNC0638	-0.16404	1.4E-06	51514
DTL	UNC1215	0.092738	0.040481	51514
DTL	VNLG/124	-0.08666	0.02772	51514
DTL	VX-11e	0.023785	0.661171	51514
DTL	VX-680	-0.08633	0.328221	51514
DTL	VX-702	-0.00289	0.9952	51514
DTL	Veliparib	0.039366	0.573815	51514
DTL	Vinblastine	-0.05392	0.223144	51514
DTL	Vinorelbine	-0.01429	0.831698	51514
DTL	Vorinostat	-0.26513	4.29E-14	51514
DTL	WH-4-023	0.137073	0.04475	51514
DTL	WZ-1-84	0.166228	0.012851	51514
DTL	WZ3105	-0.1843	8.58E-08	51514
DTL	XAV939	0.113927	0.002467	51514
DTL	XL-184	-0.03111	0.532842	51514
DTL	XMD11-85h	0.023537	0.908531	51514
DTL	XMD13-2	-0.17432	4.86E-07	51514
DTL	XMD14-99	-0.11718	0.001351	51514
DTL	XMD15-27	-0.09306	0.028796	51514

DTL	XMD8-85	0.040025	0.659052	51514
DTL	XMD8-92	0.007537	0.962116	51514
DTL	Y-39983	-0.17374	8.83E-07	51514
DTL	YK 4-279	0.032014	0.582853	51514
DTL	YM155	-0.06666	0.163841	51514
DTL	YM201636	-0.158	7.28E-06	51514
DTL	Z-LLNle-CHO	0.05846	0.445875	51514
DTL	ZG-10	-0.10557	0.082397	51514
DTL	ZM-447439	-0.14129	0.000501	51514
DTL	ZSTK474	-0.15607	8.84E-06	51514
DTL	Zibotentan	0.009162	0.987393	51514
DTL	piperlongumine	0.071671	0.085383	51514
DTL	rTRAIL	0.051728	0.464466	51514
DTL	selumetinib	0.203363	3.17E-09	51514
ECT2	(5Z)-7-Oxozeaenol	0.219755	6.43E-10	1894
ECT2	17-AAG	-0.09737	0.008615	1894
ECT2	5-Fluorouracil	0.22082	1.77E-10	1894
ECT2	681640	-0.00855	0.909212	1894
ECT2	A-443654	-0.1024	0.463042	1894
ECT2	A-770041	-0.02297	0.81439	1894
ECT2	AC220	0.153969	7.61E-05	1894
ECT2	AG-014699	0.01445	0.770041	1894
ECT2	AICAR	0.160355	2.05E-05	1894
ECT2	AKT inhibitor VIII	-0.0847	0.047524	1894
ECT2	AMG-706	0.014989	0.840712	1894
ECT2	AP-24534	0.209265	1.07E-08	1894
ECT2	AR-42	0.194465	1.88E-08	1894
ECT2	AS601245	-0.01184	0.86543	1894
ECT2	AS605240	0.164713	1.29E-05	1894
ECT2	AT-7519	0.148781	1.98E-05	1894
ECT2	ATRA	0.170785	1.89E-05	1894
ECT2	AUY922	0.086134	0.061751	1894
ECT2	AZ628	0.203191	0.001051	1894
ECT2	AZD6482	0.062324	0.155413	1894
ECT2	AZD7762	0.145447	0.000122	1894
ECT2	AZD8055	0.211498	9.99E-09	1894
ECT2	Afatinib	-0.14394	4.41E-05	1894
ECT2	Axitinib	0.115026	0.009797	1894
ECT2	BAY 61-3606	0.174607	3.51E-06	1894
ECT2	BEZ235	0.086483	0.051417	1894
ECT2	BHG712	0.268178	0	1894
ECT2	BI-2536	-0.11762	0.188984	1894
ECT2	BIRB 0796	-0.05842	0.243668	1894
ECT2	BIX02189	0.219769	1.82E-10	1894
ECT2	BMS-509744	-0.03841	0.699646	1894
ECT2	BMS-536924	0.092071	0.07674	1894
ECT2	BMS-708163	-0.04844	0.236708	1894
ECT2	BMS-754807	-0.00983	0.88541	1894
ECT2	BMS345541	0.144009	3.88E-05	1894
ECT2	BX-795	0.148218	0.000185	1894

ECT2	BX-912	0.198156	6.88E-09	1894
ECT2	Belinostat	0.173378	1.1E-06	1894
ECT2	Bexarotene	0.019544	0.833021	1894
ECT2	Bicalutamide	-0.09547	0.021107	1894
ECT2	Bleomycin	0.008919	0.90305	1894
ECT2	Bleomycin (50 uM)	-0.00486	0.907148	1894
ECT2	Bortezomib	0.010075	0.92567	1894
ECT2	Bosutinib	0.016185	0.805689	1894
ECT2	Bryostatin 1	0.001438	0.984796	1894
ECT2	CAL-101	0.156355	1.17E-05	1894
ECT2	CAY10603	0.217811	2.14E-10	1894
ECT2	CCT007093	-0.01095	0.835355	1894
ECT2	CCT018159	0.050011	0.3245	1894
ECT2	CEP-701	0.221749	1.62E-09	1894
ECT2	CGP-082996	-0.01416	0.91241	1894
ECT2	CGP-60474	0.006107	0.959002	1894
ECT2	CH5424802	0.08333	0.089574	1894
ECT2	CHIR-99021	0.008223	0.856842	1894
ECT2	CI-1040	0.124302	0.00174	1894
ECT2	CMK	0.031797	0.815658	1894
ECT2	CP466722	0.178943	2.21E-07	1894
ECT2	CP724714	-0.05838	0.251405	1894
ECT2	CUDC-101	0.148412	3.06E-05	1894
ECT2	CX-5461	0.159247	6.17E-06	1894
ECT2	Camptothecin	0.118565	0.002911	1894
ECT2	Cetuximab	-0.11987	0.001818	1894
ECT2	Cisplatin	0.012185	0.842433	1894
ECT2	Crizotinib	0.011865	0.957854	1894
ECT2	Cyclopamine	0.059161	0.584163	1894
ECT2	Cytarabine	0.043506	0.399148	1894
ECT2	DMOG	0.139412	0.000395	1894
ECT2	Dabrafenib	0.188182	4.1E-07	1894
ECT2	Dasatinib	-0.0218	0.776888	1894
ECT2	Docetaxel	-0.10974	0.003071	1894
ECT2	Doxorubicin	0.02112	0.779822	1894
ECT2	EHT 1864	0.041211	0.535713	1894
ECT2	EKB-569	0.063905	0.109305	1894
ECT2	EX-527	0.008344	0.954278	1894
ECT2	Elesclomol	-0.06491	0.136772	1894
ECT2	Embelin	-0.0177	0.808359	1894
ECT2	Epothilone B	-0.05607	0.261771	1894
ECT2	Erlotinib	-0.15056	0.024856	1894
ECT2	Etoposide	0.022372	0.683826	1894
ECT2	FH535	-0.04113	0.407429	1894
ECT2	FK866	0.144478	4.27E-05	1894
ECT2	FMK	0.083653	0.142202	1894
ECT2	FR-180204	0.092316	0.041263	1894
ECT2	FTI-277	0.010537	0.826404	1894
ECT2	Foretinib	0.133207	0.000344	1894
ECT2	GDC0449	0.096381	0.179347	1894

ECT2	GDC0941	0.020241	0.761408	1894
ECT2	GNF-2	0.009355	0.981259	1894
ECT2	GSK-650394	-0.00517	0.959368	1894
ECT2	GSK1070916	0.13984	8.7E-05	1894
ECT2	GSK1904529A	-0.0978	0.022164	1894
ECT2	GSK2126458	0.132963	0.000224	1894
ECT2	GSK269962A	0.082993	0.061624	1894
ECT2	GSK429286A	0.096739	0.012696	1894
ECT2	GSK690693	0.0653	0.080724	1894
ECT2	GW 441756	-0.01854	0.947563	1894
ECT2	GW-2580	-0.00246	0.99615	1894
ECT2	GW843682X	-0.06104	0.498631	1894
ECT2	Gefitinib	-0.09143	0.022661	1894
ECT2	Gemcitabine	0.032775	0.536799	1894
ECT2	Genentech Cpd 10	0.168574	1.46E-06	1894
ECT2	HG-5-113-01	0.079335	0.238513	1894
ECT2	HG-5-88-01	0.140666	0.094072	1894
ECT2	HG-6-64-1	0.164788	2.87E-05	1894
ECT2	I-BET-762	0.278267	0	1894
ECT2	IOX2	-0.08576	0.096183	1894
ECT2	IPA-3	0.139982	0.000181	1894
ECT2	Imatinib	0.076038	0.564146	1894
ECT2	Ispinesib Mesylate	0.158384	6.5E-06	1894
ECT2	JNJ-26854165	0.080554	0.078335	1894
ECT2	JNK Inhibitor VIII	-0.11331	0.004876	1894
ECT2	JNK-9L	0.097324	0.03982	1894
ECT2	JQ1	0.148824	0.000104	1894
ECT2	JQ12	0.124677	0.006894	1894
ECT2	JW-7-24-1	0.228071	2.2E-11	1894
ECT2	JW-7-52-1	0.012196	0.927705	1894
ECT2	KIN001-055	-0.00433	0.963928	1894
ECT2	KIN001-102	0.139621	6.09E-05	1894
ECT2	KIN001-135	0.006068	0.969935	1894
ECT2	KIN001-236	0.191152	4.66E-08	1894
ECT2	KIN001-244	0.179379	3.24E-07	1894
ECT2	KIN001-260	0.22713	3.88E-11	1894
ECT2	KIN001-266	0.068316	0.132599	1894
ECT2	KIN001-270	0.092694	0.014875	1894
ECT2	KU-55933	0.036897	0.567908	1894
ECT2	LAQ824	0.104824	0.006066	1894
ECT2	LFM-A13	-0.06038	0.214863	1894
ECT2	LY317615	0.052622	0.228256	1894
ECT2	Lapatinib	-0.16772	0.004827	1894
ECT2	Lenalidomide	0.059899	0.367232	1894
ECT2	Linifanib	0.17058	7.42E-06	1894
ECT2	Lisitinib	0.022948	0.75884	1894
ECT2	MG-132	0.020461	0.860563	1894
ECT2	MK-2206	-0.00052	0.993949	1894
ECT2	MLN4924	0.038281	0.519633	1894
ECT2	MP470	0.037643	0.402661	1894

ECT2	MPS-1-IN-1	0.16513	3.8E-06	1894
ECT2	MS-275	0.01357	0.899269	1894
ECT2	Masitinib	0.236449	1E-11	1894
ECT2	Methotrexate	0.259532	1.47E-13	1894
ECT2	Midostaurin	0.037131	0.428516	1894
ECT2	Mitomycin C	0.032214	0.560353	1894
ECT2	NG-25	0.249289	2.13E-13	1894
ECT2	NPK76-II-72-1	0.142116	3.88E-05	1894
ECT2	NSC-207895	-0.0157	0.769167	1894
ECT2	NSC-87877	-0.01094	0.8788	1894
ECT2	NU-7441	0.039525	0.594298	1894
ECT2	Navitoclax	0.111979	0.002339	1894
ECT2	Nilotinib	0.145754	0.000539	1894
ECT2	Nutlin-3a (-)	0.268497	1.26E-12	1894
ECT2	OSI-027	0.194971	1.92E-08	1894
ECT2	OSI-930	0.197459	3.15E-08	1894
ECT2	OSU-03012	0.02888	0.594643	1894
ECT2	Obatoclax Mesylate	0.11799	0.004458	1894
ECT2	Olaparib	0.025108	0.597601	1894
ECT2	PAC-1	0.084705	0.036559	1894
ECT2	PD-0325901	0.038459	0.362869	1894
ECT2	PD-0332991	0.136566	0.002084	1894
ECT2	PD-173074	0.013735	0.947772	1894
ECT2	PF-4708671	0.026635	0.820759	1894
ECT2	PF-562271	0.010241	0.885379	1894
ECT2	PFI-1	0.166369	3.07E-05	1894
ECT2	PHA-665752	-0.04207	0.789013	1894
ECT2	PHA-793887	0.206823	1.2E-09	1894
ECT2	PI-103	0.18058	2.18E-07	1894
ECT2	PIK-93	0.198666	6.02E-09	1894
ECT2	PLX4720	0.150502	4E-05	1894
ECT2	Paclitaxel	-0.07729	0.446745	1894
ECT2	Parthenolide	0.065222	0.522311	1894
ECT2	Pazopanib	0.05024	0.355072	1894
ECT2	Phenformin	0.15165	2.31E-05	1894
ECT2	Pyrimethamine	0.048852	0.682893	1894
ECT2	QL-VIII-58	0.107126	0.102625	1894
ECT2	QL-X-138	0.155375	1.08E-05	1894
ECT2	QL-XI-92	0.228723	2.46E-11	1894
ECT2	QL-XII-47	0.099202	0.009524	1894
ECT2	QL-XII-61	0.119892	0.043293	1894
ECT2	QS11	0.00865	0.919944	1894
ECT2	RDEA119	0.104413	0.003526	1894
ECT2	RO-3306	-0.06698	0.109462	1894
ECT2	Rapamycin	-0.02669	0.860409	1894
ECT2	Roscovitine	0.052544	0.761323	1894
ECT2	Ruxolitinib	0.125503	0.002435	1894
ECT2	S-Trityl-L-cysteine	0.069068	0.394309	1894
ECT2	SB 216763	-0.03441	0.510594	1894
ECT2	SB 505124	-0.01293	0.900594	1894

ECT2	SB52334	0.053567	0.246232	1894
ECT2	SB590885	0.13062	0.001934	1894
ECT2	SGC0946	-0.06699	0.167316	1894
ECT2	SL 0101-1	0.015997	0.861724	1894
ECT2	SN-38	0.106847	0.006733	1894
ECT2	SNX-2112	0.223814	7.71E-11	1894
ECT2	STF-62247	0.127631	0.000811	1894
ECT2	Salubrinal	0.115763	0.13497	1894
ECT2	Saracatinib	-0.07468	0.363589	1894
ECT2	Shikonin	0.108269	0.017413	1894
ECT2	Sorafenib	0.134619	0.09984	1894
ECT2	Sunitinib	0.178982	0.004416	1894
ECT2	T0901317	0.09622	0.013452	1894
ECT2	TAE684	-0.03037	0.823305	1894
ECT2	TAK-715	0.138949	8.21E-05	1894
ECT2	TG101348	0.251296	1.16E-13	1894
ECT2	TGX221	0.024124	0.738761	1894
ECT2	THZ-2-102-1	0.185185	1.07E-07	1894
ECT2	THZ-2-49	0.17675	5.75E-07	1894
ECT2	TL-1-85	0.271192	0	1894
ECT2	TL-2-105	0.14135	7.77E-05	1894
ECT2	TPCA-1	0.238154	1.91E-12	1894
ECT2	TW 37	0.005393	0.930187	1894
ECT2	Talazoparib	0.083706	0.046444	1894
ECT2	Tamoxifen	0.031851	0.799583	1894
ECT2	Temozolomide	0.087587	0.100606	1894
ECT2	Temsirolimus	0.085419	0.053608	1894
ECT2	Thapsigargin	0.006139	0.947463	1894
ECT2	Tipifarnib	0.050803	0.420147	1894
ECT2	Tivozanib	0.113938	0.01082	1894
ECT2	Trametinib	0.053629	0.162619	1894
ECT2	Tubastatin A	0.21598	2.74E-10	1894
ECT2	UNC0638	0.224817	1.68E-11	1894
ECT2	UNC1215	-0.01699	0.777499	1894
ECT2	VNLG/124	0.194551	7.41E-08	1894
ECT2	VX-11e	0.148879	0.000205	1894
ECT2	VX-680	0.033689	0.754921	1894
ECT2	VX-702	0.065429	0.676357	1894
ECT2	Veliparib	-0.0315	0.66381	1894
ECT2	Vinblastine	0.137589	0.000521	1894
ECT2	Vinorelbine	0.004912	0.948229	1894
ECT2	Vorinostat	0.190572	9.31E-08	1894
ECT2	WH-4-023	0.012254	0.898925	1894
ECT2	WZ-1-84	-0.0507	0.534005	1894
ECT2	WZ3105	0.138107	7.55E-05	1894
ECT2	XAV939	-0.15862	1.61E-05	1894
ECT2	XL-184	0.158108	4.31E-05	1894
ECT2	XMD11-85h	0.139508	0.165752	1894
ECT2	XMD13-2	0.196936	1.05E-08	1894
ECT2	XMD14-99	0.224809	1.38E-10	1894

ECT2	XMD15-27	0.105937	0.011007	1894
ECT2	XMD8-85	0.178653	0.007419	1894
ECT2	XMD8-92	0.103054	0.238756	1894
ECT2	Y-39983	0.133052	0.00021	1894
ECT2	YK 4-279	0.117811	0.009742	1894
ECT2	YM155	0.02435	0.683089	1894
ECT2	YM201636	0.132466	0.000196	1894
ECT2	Z-LLNle-CHO	0.103144	0.135939	1894
ECT2	ZG-10	0.091072	0.144351	1894
ECT2	ZM-447439	0.114253	0.005954	1894
ECT2	ZSTK474	0.144696	4.05E-05	1894
ECT2	Zibotentan	0.00069	0.998902	1894
ECT2	piperlongumine	0.064468	0.127814	1894
ECT2	rTRAIL	0.074347	0.241281	1894
ECT2	selumetinib	0.111759	0.00179	1894
ERCC6L	(5Z)-7-Oxozeaenol	0.070741	0.07714	54821
ERCC6L	17-AAG	0.06057	0.111153	54821
ERCC6L	5-Fluorouracil	0.037098	0.334177	54821
ERCC6L	681640	0.013466	0.852839	54821
ERCC6L	A-443654	-0.07468	0.63116	54821
ERCC6L	A-770041	-0.10434	0.154285	54821
ERCC6L	AC220	0.049827	0.289748	54821
ERCC6L	AG-014699	0.054693	0.199456	54821
ERCC6L	AICAR	-0.05031	0.219335	54821
ERCC6L	AKT inhibitor VIII	0.039413	0.404167	54821
ERCC6L	AMG-706	0.013177	0.864588	54821
ERCC6L	AP-24534	-0.0292	0.514445	54821
ERCC6L	AR-42	-0.05537	0.132301	54821
ERCC6L	AS601245	0.030845	0.598953	54821
ERCC6L	AS605240	0.024809	0.604982	54821
ERCC6L	AT-7519	-0.05937	0.104163	54821
ERCC6L	ATRA	0.011204	0.856018	54821
ERCC6L	AUY922	0.008463	0.904008	54821
ERCC6L	AZ628	0.125954	0.061309	54821
ERCC6L	AZD6482	-0.01888	0.738891	54821
ERCC6L	AZD7762	-0.14083	0.000206	54821
ERCC6L	AZD8055	-0.06211	0.120239	54821
ERCC6L	Afatinib	0.08064	0.028102	54821
ERCC6L	Axitinib	-0.07252	0.131942	54821
ERCC6L	BAY 61-3606	0.027398	0.536511	54821
ERCC6L	BEZ235	-0.04377	0.37951	54821
ERCC6L	BHG712	-0.03174	0.411336	54821
ERCC6L	BI-2536	-0.15604	0.070866	54821
ERCC6L	BIRB 0796	0.005805	0.926402	54821
ERCC6L	BIX02189	-0.03423	0.376374	54821
ERCC6L	BMS-509744	-0.04244	0.666837	54821
ERCC6L	BMS-536924	0.034708	0.635814	54821
ERCC6L	BMS-708163	0.073156	0.064514	54821
ERCC6L	BMS-754807	0.080074	0.106067	54821
ERCC6L	BMS345541	-0.0921	0.010099	54821

ERCC6L	BX-795	-0.04828	0.296251	54821
ERCC6L	BX-912	-0.05471	0.1339	54821
ERCC6L	Belinostat	-0.0449	0.244607	54821
ERCC6L	Bexarotene	0.013391	0.893731	54821
ERCC6L	Bicalutamide	0.007235	0.898962	54821
ERCC6L	Bleomycin	0.042318	0.454629	54821
ERCC6L	Bleomycin (50 uM)	0.018973	0.632294	54821
ERCC6L	Bortezomib	-0.05826	0.477036	54821
ERCC6L	Bosutinib	-0.07019	0.145788	54821
ERCC6L	Bryostatin 1	0.029141	0.608345	54821
ERCC6L	CAL-101	-0.04619	0.237544	54821
ERCC6L	CAY10603	-0.06909	0.056962	54821
ERCC6L	CCT007093	0.066594	0.116098	54821
ERCC6L	CCT018159	-0.02741	0.626807	54821
ERCC6L	CEP-701	-0.07695	0.054698	54821
ERCC6L	CGP-082996	-0.0524	0.601966	54821
ERCC6L	CGP-60474	-0.09342	0.23123	54821
ERCC6L	CH5424802	-0.01814	0.802776	54821
ERCC6L	CHIR-99021	0.002512	0.958086	54821
ERCC6L	CI-1040	0.101344	0.012545	54821
ERCC6L	CMK	-0.05178	0.671908	54821
ERCC6L	CP466722	-0.08073	0.024866	54821
ERCC6L	CP724714	0.060856	0.226777	54821
ERCC6L	CUDC-101	-0.05293	0.161121	54821
ERCC6L	CX-5461	-0.05405	0.151341	54821
ERCC6L	Camptothecin	-0.08288	0.046751	54821
ERCC6L	Cetuximab	0.076778	0.054997	54821
ERCC6L	Cisplatin	-0.03661	0.479035	54821
ERCC6L	Crizotinib	-0.06929	0.602373	54821
ERCC6L	Cyclopamine	-0.00669	0.964346	54821
ERCC6L	Cytarabine	-0.05741	0.241126	54821
ERCC6L	DMOG	-0.03539	0.462826	54821
ERCC6L	Dabrafenib	0.075473	0.068431	54821
ERCC6L	Dasatinib	-0.05592	0.4218	54821
ERCC6L	Docetaxel	-0.02733	0.493913	54821
ERCC6L	Doxorubicin	0.014503	0.856445	54821
ERCC6L	EHT 1864	0.082474	0.135047	54821
ERCC6L	EKB-569	-0.03952	0.348092	54821
ERCC6L	EX-527	-0.04449	0.670471	54821
ERCC6L	Elesclomol	-0.0688	0.112755	54821
ERCC6L	Embelin	-0.02274	0.745571	54821
ERCC6L	Epothilone B	-0.00849	0.893894	54821
ERCC6L	Erlotinib	0.072431	0.323007	54821
ERCC6L	Etoposide	-0.04776	0.316311	54821
ERCC6L	FH535	0.00795	0.896837	54821
ERCC6L	FK866	-0.08179	0.024706	54821
ERCC6L	FMK	0.009415	0.915941	54821
ERCC6L	FR-180204	0.07004	0.140749	54821
ERCC6L	FTI-277	0.091922	0.021946	54821
ERCC6L	Foretinib	-0.02978	0.509417	54821

ERCC6L	GDC0449	0.020049	0.868182	54821
ERCC6L	GDC0941	-0.05984	0.247163	54821
ERCC6L	GNF-2	-0.03522	0.896506	54821
ERCC6L	GSK-650394	-0.00163	0.986343	54821
ERCC6L	GSK1070916	-0.09639	0.008159	54821
ERCC6L	GSK1904529A	-0.02588	0.608363	54821
ERCC6L	GSK2126458	-0.04559	0.240933	54821
ERCC6L	GSK269962A	-0.01996	0.745193	54821
ERCC6L	GSK429286A	-0.10887	0.004478	54821
ERCC6L	GSK690693	-0.11463	0.001427	54821
ERCC6L	GW 441756	-0.03821	0.846614	54821
ERCC6L	GW-2580	0.007936	0.994893	54821
ERCC6L	GW843682X	-0.14982	0.042218	54821
ERCC6L	Gefitinib	0.109077	0.005754	54821
ERCC6L	Gemcitabine	-0.04763	0.336581	54821
ERCC6L	Genentech Cpd 10	-0.08447	0.021101	54821
ERCC6L	HG-5-113-01	-0.07238	0.292222	54821
ERCC6L	HG-5-88-01	0.037234	0.823369	54821
ERCC6L	HG-6-64-1	-0.00214	0.974592	54821
ERCC6L	I-BET-762	-0.07221	0.041888	54821
ERCC6L	IOX2	-0.00104	0.991631	54821
ERCC6L	IPA-3	-0.05642	0.158978	54821
ERCC6L	Imatinib	-0.01052	0.963404	54821
ERCC6L	Ispinesib Mesylate	-0.03673	0.342796	54821
ERCC6L	JNJ-26854165	0.031558	0.568695	54821
ERCC6L	JNK Inhibitor VIII	0.013292	0.785377	54821
ERCC6L	JNK-9L	0.021887	0.741503	54821
ERCC6L	JQ1	0.002231	0.969963	54821
ERCC6L	JQ12	0.028097	0.638008	54821
ERCC6L	JW-7-24-1	-0.0638	0.079924	54821
ERCC6L	JW-7-52-1	-0.07218	0.453372	54821
ERCC6L	KIN001-055	0.047881	0.428955	54821
ERCC6L	KIN001-102	-0.05362	0.143415	54821
ERCC6L	KIN001-135	0.050816	0.640643	54821
ERCC6L	KIN001-236	-0.01576	0.704626	54821
ERCC6L	KIN001-244	-0.04423	0.248386	54821
ERCC6L	KIN001-260	-0.03983	0.296394	54821
ERCC6L	KIN001-266	-0.00596	0.919701	54821
ERCC6L	KIN001-270	-0.11085	0.003119	54821
ERCC6L	KU-55933	-0.05253	0.365984	54821
ERCC6L	LAQ824	-0.03595	0.390633	54821
ERCC6L	LFM-A13	0.033016	0.543967	54821
ERCC6L	LY317615	-0.02745	0.563839	54821
ERCC6L	Lapatinib	0.106906	0.086429	54821
ERCC6L	Lenalidomide	-0.07655	0.206974	54821
ERCC6L	Linifanib	0.052898	0.287126	54821
ERCC6L	Lisitinib	0.083557	0.114664	54821
ERCC6L	MG-132	-0.08079	0.335633	54821
ERCC6L	MK-2206	-0.08359	0.079186	54821
ERCC6L	MLN4924	0.028352	0.648265	54821

ERCC6L	MP470	-0.0156	0.750953	54821
ERCC6L	MPS-1-IN-1	0.002866	0.950384	54821
ERCC6L	MS-275	-0.08878	0.25197	54821
ERCC6L	Masitinib	-0.0319	0.42802	54821
ERCC6L	Methotrexate	-0.06229	0.099984	54821
ERCC6L	Midostaurin	0.025416	0.605126	54821
ERCC6L	Mitomycin C	0.008035	0.903302	54821
ERCC6L	NG-25	-0.04455	0.236236	54821
ERCC6L	NPK76-II-72-1	-0.12006	0.000565	54821
ERCC6L	NSC-207895	-0.14551	0.000217	54821
ERCC6L	NSC-87877	0.086733	0.090581	54821
ERCC6L	NU-7441	-0.03499	0.648672	54821
ERCC6L	Navitoclax	-0.06581	0.082469	54821
ERCC6L	Nilotinib	-0.04021	0.451057	54821
ERCC6L	Nutlin-3a (-)	0.102438	0.016315	54821
ERCC6L	OSI-027	-0.05826	0.114828	54821
ERCC6L	OSI-930	-0.03296	0.425297	54821
ERCC6L	OSU-03012	-0.018	0.757267	54821
ERCC6L	Obatoclax Mesylate	-0.00516	0.932645	54821
ERCC6L	Olaparib	0.032902	0.475069	54821
ERCC6L	PAC-1	-0.04405	0.312809	54821
ERCC6L	PD-0325901	0.116327	0.002444	54821
ERCC6L	PD-0332991	0.020151	0.760531	54821
ERCC6L	PD-173074	-0.03382	0.829174	54821
ERCC6L	PF-4708671	0.055737	0.535089	54821
ERCC6L	PF-562271	-0.00025	0.997467	54821
ERCC6L	PFI-1	0.0093	0.879436	54821
ERCC6L	PHA-665752	0.085852	0.528136	54821
ERCC6L	PHA-793887	-0.04013	0.275879	54821
ERCC6L	PI-103	-0.06321	0.085302	54821
ERCC6L	PIK-93	-0.08606	0.015354	54821
ERCC6L	PLX4720	0.068555	0.090473	54821
ERCC6L	Paclitaxel	-0.10058	0.278389	54821
ERCC6L	Parthenolide	-0.03274	0.783154	54821
ERCC6L	Pazopanib	0.022333	0.72147	54821
ERCC6L	Phenformin	-0.00792	0.853939	54821
ERCC6L	Pyrimethamine	-0.01603	0.910668	54821
ERCC6L	QL-VIII-58	-0.02256	0.810831	54821
ERCC6L	QL-X-138	-0.07852	0.032596	54821
ERCC6L	QL-XI-92	-0.07034	0.054266	54821
ERCC6L	QL-XII-47	-0.03276	0.440903	54821
ERCC6L	QL-XII-61	-0.06281	0.347222	54821
ERCC6L	QS11	-0.00707	0.934908	54821
ERCC6L	RDEA119	0.153188	1.09E-05	54821
ERCC6L	RO-3306	0.01136	0.813503	54821
ERCC6L	Rapamycin	-0.09372	0.368279	54821
ERCC6L	Roscovitine	0.050554	0.774184	54821
ERCC6L	Ruxolitinib	-0.02831	0.619745	54821
ERCC6L	S-Trityl-L-cysteine	-0.0676	0.406858	54821
ERCC6L	SB 216763	0.041349	0.416596	54821

ERCC6L	SB 505124	0.025343	0.771332	54821
ERCC6L	SB52334	0.031255	0.54479	54821
ERCC6L	SB590885	0.106179	0.015796	54821
ERCC6L	SGC0946	-0.00031	0.996778	54821
ERCC6L	SL 0101-1	0.069113	0.343581	54821
ERCC6L	SN-38	-0.02429	0.623349	54821
ERCC6L	SNX-2112	-0.05017	0.183067	54821
ERCC6L	STF-62247	-0.07266	0.066701	54821
ERCC6L	Salubrinal	0.012623	0.907568	54821
ERCC6L	Saracatinib	-0.06297	0.454465	54821
ERCC6L	Shikonin	-0.02974	0.606524	54821
ERCC6L	Sorafenib	0.117227	0.172316	54821
ERCC6L	Sunitinib	0.016939	0.858756	54821
ERCC6L	T0901317	-0.04903	0.240256	54821
ERCC6L	TAE684	-0.05578	0.626344	54821
ERCC6L	TAK-715	-0.04191	0.270141	54821
ERCC6L	TG101348	-0.04262	0.255387	54821
ERCC6L	TGX221	0.041282	0.551656	54821
ERCC6L	THZ-2-102-1	-0.08475	0.019364	54821
ERCC6L	THZ-2-49	-0.04023	0.296492	54821
ERCC6L	TL-1-85	-0.04475	0.236367	54821
ERCC6L	TL-2-105	-0.03901	0.318627	54821
ERCC6L	TPCA-1	-0.08743	0.01383	54821
ERCC6L	TW 37	-0.08151	0.052934	54821
ERCC6L	Talazoparib	0.00599	0.915067	54821
ERCC6L	Tamoxifen	0.03008	0.814148	54821
ERCC6L	Temozolomide	-0.01528	0.866878	54821
ERCC6L	Temsirolimus	-0.09701	0.024724	54821
ERCC6L	Thapsigargin	-0.02526	0.737202	54821
ERCC6L	Tipifarnib	-0.00138	0.98816	54821
ERCC6L	Tivozanib	-0.04581	0.42411	54821
ERCC6L	Trametinib	0.157301	9.52E-06	54821
ERCC6L	Tubastatin A	-0.03776	0.314749	54821
ERCC6L	UNC0638	-0.04159	0.25735	54821
ERCC6L	UNC1215	0.051051	0.305608	54821
ERCC6L	VNLG/124	0.031955	0.471806	54821
ERCC6L	VX-11e	0.100306	0.017306	54821
ERCC6L	VX-680	-0.06378	0.497518	54821
ERCC6L	VX-702	0.012932	0.963653	54821
ERCC6L	Veliparib	0.018023	0.823935	54821
ERCC6L	Vinblastine	-0.09192	0.026608	54821
ERCC6L	Vinorelbine	-0.05457	0.301982	54821
ERCC6L	Vorinostat	-0.11895	0.001065	54821
ERCC6L	WH-4-023	-0.03744	0.663944	54821
ERCC6L	WZ-1-84	0.025837	0.775313	54821
ERCC6L	WZ3105	-0.06633	0.067164	54821
ERCC6L	XAV939	0.005318	0.909516	54821
ERCC6L	XL-184	0.035278	0.473513	54821
ERCC6L	XMD11-85h	0.025137	0.900288	54821
ERCC6L	XMD13-2	-0.03824	0.313733	54821

ERCC6L	XMD14-99	0.032655	0.422258	54821
ERCC6L	XMD15-27	-0.01827	0.737946	54821
ERCC6L	XMD8-85	0.035904	0.699446	54821
ERCC6L	XMD8-92	-0.05989	0.573198	54821
ERCC6L	Y-39983	-0.12728	0.000406	54821
ERCC6L	YK 4-279	-0.06398	0.205564	54821
ERCC6L	YM155	-0.09348	0.037219	54821
ERCC6L	YM201636	-0.05076	0.180078	54821
ERCC6L	Z-LLNle-CHO	-0.04404	0.58624	54821
ERCC6L	ZG-10	-0.09021	0.14888	54821
ERCC6L	ZM-447439	-0.06839	0.125304	54821
ERCC6L	ZSTK474	-0.06561	0.074727	54821
ERCC6L	Zibotentan	-0.02226	0.949888	54821
ERCC6L	piperlongumine	-0.04363	0.328411	54821
ERCC6L	rTRAIL	0.042195	0.566393	54821
ERCC6L	selumetinib	0.162343	3.15E-06	54821
ESCO2	(5Z)-7-Oxozeaenol	0.112071	0.003094	157570
ESCO2	17-AAG	0.215953	1.43E-09	157570
ESCO2	5-Fluorouracil	-0.11991	0.000799	157570
ESCO2	681640	0.027234	0.67706	157570
ESCO2	A-443654	-0.09317	0.514381	157570
ESCO2	A-770041	-0.07647	0.330197	157570
ESCO2	AC220	-0.07609	0.080285	157570
ESCO2	AG-014699	0.082648	0.044567	157570
ESCO2	AICAR	-0.15872	2.51E-05	157570
ESCO2	AKT inhibitor VIII	0.092052	0.029744	157570
ESCO2	AMG-706	0.080882	0.118164	157570
ESCO2	AP-24534	-0.16243	1.43E-05	157570
ESCO2	AR-42	-0.20363	3.57E-09	157570
ESCO2	AS601245	0.074199	0.132469	157570
ESCO2	AS605240	-0.04885	0.260838	157570
ESCO2	AT-7519	-0.20723	1.51E-09	157570
ESCO2	ATRA	-0.11431	0.006996	157570
ESCO2	AUY922	0.009704	0.888883	157570
ESCO2	AZ628	0.051572	0.521536	157570
ESCO2	AZD6482	-0.01515	0.795033	157570
ESCO2	AZD7762	-0.21541	5.17E-09	157570
ESCO2	AZD8055	-0.2	6.79E-08	157570
ESCO2	Afatinib	0.114912	0.001342	157570
ESCO2	Axitinib	-0.085	0.068418	157570
ESCO2	BAY 61-3606	-0.13009	0.00072	157570
ESCO2	BEZ235	-0.0362	0.480953	157570
ESCO2	BHG712	-0.20716	1.83E-09	157570
ESCO2	BI-2536	-0.1475	0.089113	157570
ESCO2	BIRB 0796	0.081633	0.087293	157570
ESCO2	BIX02189	-0.21852	2.35E-10	157570
ESCO2	BMS-509744	0.012199	0.915822	157570
ESCO2	BMS-536924	-0.00989	0.920004	157570
ESCO2	BMS-708163	0.120268	0.001741	157570
ESCO2	BMS-754807	0.046926	0.389161	157570

ESCO2	BMS345541	-0.22839	2.47E-11	157570
ESCO2	BX-795	-0.10201	0.013922	157570
ESCO2	BX-912	-0.24153	9.42E-13	157570
ESCO2	Belinostat	-0.17048	1.69E-06	157570
ESCO2	Bexarotene	-0.02318	0.791755	157570
ESCO2	Bicalutamide	0.051397	0.24784	157570
ESCO2	Bleomycin	0.050912	0.350044	157570
ESCO2	Bleomycin (50 uM)	0.183342	1.48E-07	157570
ESCO2	Bortezomib	-0.01786	0.861093	157570
ESCO2	Bosutinib	-0.09138	0.044824	157570
ESCO2	Bryostatin 1	0.069626	0.151889	157570
ESCO2	CAL-101	-0.20763	2.79E-09	157570
ESCO2	CAY10603	-0.19934	7.31E-09	157570
ESCO2	CCT007093	0.128917	0.001214	157570
ESCO2	CCT018159	0.032369	0.55696	157570
ESCO2	CEP-701	-0.16295	1.5E-05	157570
ESCO2	CGP-082996	-0.00671	0.96101	157570
ESCO2	CGP-60474	-0.02127	0.842267	157570
ESCO2	CH5424802	-0.01531	0.835979	157570
ESCO2	CHIR-99021	0.062441	0.103455	157570
ESCO2	CI-1040	0.081356	0.051146	157570
ESCO2	CMK	-0.02671	0.850474	157570
ESCO2	CP466722	-0.22438	4.83E-11	157570
ESCO2	CP724714	0.070906	0.143672	157570
ESCO2	CUDC-101	-0.19122	5.11E-08	157570
ESCO2	CX-5461	-0.16904	1.47E-06	157570
ESCO2	Camptothecin	-0.10494	0.009348	157570
ESCO2	Cetuximab	0.132946	0.000493	157570
ESCO2	Cisplatin	0.031367	0.552403	157570
ESCO2	Crizotinib	-0.08769	0.455238	157570
ESCO2	Cyclopamine	-0.04334	0.712864	157570
ESCO2	Cytarabine	-0.0197	0.738135	157570
ESCO2	DMOG	-0.13764	0.000482	157570
ESCO2	Dabrafenib	0.082224	0.04465	157570
ESCO2	Dasatinib	0.007166	0.929675	157570
ESCO2	Docetaxel	0.163573	7.5E-06	157570
ESCO2	Doxorubicin	-0.01452	0.856232	157570
ESCO2	EHT 1864	0.030986	0.669544	157570
ESCO2	EKB-569	-0.12886	0.000577	157570
ESCO2	EX-527	-0.01941	0.87659	157570
ESCO2	Elesclomol	0.020256	0.688311	157570
ESCO2	Embelin	0.023896	0.73032	157570
ESCO2	Epothilone B	0.031202	0.568604	157570
ESCO2	Erlotinib	0.112557	0.108401	157570
ESCO2	Etoposide	-0.09842	0.020695	157570
ESCO2	FH535	0.093207	0.030492	157570
ESCO2	FK866	-0.23441	1.01E-11	157570
ESCO2	FMK	-0.06493	0.292637	157570
ESCO2	FR-180204	-0.07935	0.088286	157570
ESCO2	FTI-277	0.191347	5.23E-07	157570

ESCO2	Foretinib	-0.16659	5.2E-06	157570
ESCO2	GDC0449	-0.01548	0.905858	157570
ESCO2	GDC0941	-0.09861	0.031277	157570
ESCO2	GNF-2	-0.05861	0.80029	157570
ESCO2	GSK-650394	-0.04641	0.510902	157570
ESCO2	GSK1070916	-0.25417	1.51E-13	157570
ESCO2	GSK1904529A	0.081773	0.060192	157570
ESCO2	GSK2126458	-0.19597	2.4E-08	157570
ESCO2	GSK269962A	-0.018	0.772832	157570
ESCO2	GSK429286A	-0.16307	9.92E-06	157570
ESCO2	GSK690693	-0.24247	1.72E-12	157570
ESCO2	GW 441756	-0.00666	0.990735	157570
ESCO2	GW-2580	-0.01402	0.986416	157570
ESCO2	GW843682X	-0.1513	0.040082	157570
ESCO2	Gefitinib	0.106515	0.007141	157570
ESCO2	Gemcitabine	-0.08639	0.054737	157570
ESCO2	Genentech Cpd 10	-0.18277	1.56E-07	157570
ESCO2	HG-5-113-01	-0.07721	0.254837	157570
ESCO2	HG-5-88-01	0.012093	0.95712	157570
ESCO2	HG-6-64-1	-0.05144	0.280451	157570
ESCO2	I-BET-762	-0.28482	1.29E-17	157570
ESCO2	IOX2	0.033817	0.579428	157570
ESCO2	IPA-3	-0.15055	5.21E-05	157570
ESCO2	Imatinib	-0.1004	0.359009	157570
ESCO2	Ispinesib Mesylate	-0.14856	2.49E-05	157570
ESCO2	JNJ-26854165	0.052594	0.290776	157570
ESCO2	JNK Inhibitor VIII	0.10287	0.011214	157570
ESCO2	JNK-9L	-0.04182	0.467663	157570
ESCO2	JQ1	-0.07394	0.072627	157570
ESCO2	JQ12	-0.02616	0.663632	157570
ESCO2	JW-7-24-1	-0.217	2.16E-10	157570
ESCO2	JW-7-52-1	-0.0196	0.878443	157570
ESCO2	KIN001-055	-0.00611	0.947124	157570
ESCO2	KIN001-102	-0.22566	3.52E-11	157570
ESCO2	KIN001-135	0.078556	0.417894	157570
ESCO2	KIN001-236	-0.17776	4.22E-07	157570
ESCO2	KIN001-244	-0.21803	3.3E-10	157570
ESCO2	KIN001-260	-0.21477	4.73E-10	157570
ESCO2	KIN001-266	-0.10103	0.019729	157570
ESCO2	KIN001-270	-0.13055	0.000437	157570
ESCO2	KU-55933	-0.0965	0.052845	157570
ESCO2	LAQ824	-0.14652	8.41E-05	157570
ESCO2	LFM-A13	0.052927	0.287317	157570
ESCO2	LY317615	-0.10783	0.007007	157570
ESCO2	Lapatinib	0.189862	0.001189	157570
ESCO2	Lenalidomide	-0.06895	0.274315	157570
ESCO2	Linifanib	-0.05582	0.255701	157570
ESCO2	Lisitinib	0.047831	0.445551	157570
ESCO2	MG-132	0.012426	0.921204	157570
ESCO2	MK-2206	-0.14574	0.000924	157570

ESCO2	MLN4924	0.083654	0.110055	157570
ESCO2	MP470	-0.05482	0.203663	157570
ESCO2	MPS-1-IN-1	-0.11604	0.001471	157570
ESCO2	MS-275	-0.1695	0.014489	157570
ESCO2	Masitinib	-0.16406	4.03E-06	157570
ESCO2	Methotrexate	-0.23423	3.36E-11	157570
ESCO2	Midostaurin	0.046994	0.301286	157570
ESCO2	Mitomycin C	-0.09255	0.040536	157570
ESCO2	NG-25	-0.2409	1.44E-12	157570
ESCO2	NPK76-II-72-1	-0.31694	7.75E-22	157570
ESCO2	NSC-207895	-0.15606	6.9E-05	157570
ESCO2	NSC-87877	0.130718	0.009192	157570
ESCO2	NU-7441	-0.06133	0.348837	157570
ESCO2	Navitoclax	-0.29962	7.62E-18	157570
ESCO2	Nilotinib	-0.11018	0.01211	157570
ESCO2	Nutlin-3a (-)	-0.00684	0.911105	157570
ESCO2	OSI-027	-0.20194	5.54E-09	157570
ESCO2	OSI-930	-0.1141	0.002109	157570
ESCO2	OSU-03012	-0.04759	0.337743	157570
ESCO2	Obatoclax Mesylate	-0.02283	0.671537	157570
ESCO2	Olaparib	0.064788	0.129992	157570
ESCO2	PAC-1	-0.15654	4.29E-05	157570
ESCO2	PD-0325901	0.167066	7.86E-06	157570
ESCO2	PD-0332991	-0.00952	0.898149	157570
ESCO2	PD-173074	-0.03485	0.823865	157570
ESCO2	PF-4708671	0.043078	0.658073	157570
ESCO2	PF-562271	0.029898	0.625211	157570
ESCO2	PFI-1	-0.03412	0.505828	157570
ESCO2	PHA-665752	0.026487	0.875673	157570
ESCO2	PHA-793887	-0.2116	4.73E-10	157570
ESCO2	PI-103	-0.22064	1.49E-10	157570
ESCO2	PIK-93	-0.29629	5.83E-19	157570
ESCO2	PLX4720	0.093947	0.015793	157570
ESCO2	Paclitaxel	-0.01909	0.899018	157570
ESCO2	Parthenolide	-0.07253	0.464234	157570
ESCO2	Pazopanib	0.025283	0.680099	157570
ESCO2	Phenformin	-0.12484	0.000549	157570
ESCO2	Pyrimethamine	-0.03821	0.764465	157570
ESCO2	QL-VIII-58	-0.00201	0.986614	157570
ESCO2	QL-X-138	-0.20784	2.18E-09	157570
ESCO2	QL-XI-92	-0.22333	7.63E-11	157570
ESCO2	QL-XII-47	-0.12049	0.00136	157570
ESCO2	QL-XII-61	-0.15218	0.007797	157570
ESCO2	QS11	-0.09649	0.070222	157570
ESCO2	RDEA119	0.178267	2.4E-07	157570
ESCO2	RO-3306	0.104569	0.009809	157570
ESCO2	Rapamycin	-0.08815	0.40127	157570
ESCO2	Roscovitine	0.084741	0.574217	157570
ESCO2	Ruxolitinib	-0.03382	0.535598	157570
ESCO2	S-Trityl-L-cysteine	-0.09756	0.19843	157570

ESCO2	SB 216763	0.058764	0.232287	157570
ESCO2	SB 505124	0.044249	0.546338	157570
ESCO2	SB52334	-0.02898	0.580334	157570
ESCO2	SB590885	0.123886	0.003647	157570
ESCO2	SGC0946	0.079274	0.088413	157570
ESCO2	SL 0101-1	0.043268	0.574678	157570
ESCO2	SN-38	-0.0015	0.979994	157570
ESCO2	SNX-2112	-0.19899	9.21E-09	157570
ESCO2	STF-62247	-0.14883	7.72E-05	157570
ESCO2	Salubrinal	-0.04877	0.587531	157570
ESCO2	Saracatinib	-0.06823	0.411433	157570
ESCO2	Shikonin	-0.06679	0.177655	157570
ESCO2	Sorafenib	-0.02013	0.882182	157570
ESCO2	Sunitinib	-0.11341	0.095334	157570
ESCO2	T0901317	-0.15241	4.37E-05	157570
ESCO2	TAE684	0.036109	0.786723	157570
ESCO2	TAK-715	-0.19232	3.16E-08	157570
ESCO2	TG101348	-0.21372	4.04E-10	157570
ESCO2	TGX221	0.133541	0.029598	157570
ESCO2	THZ-2-102-1	-0.23518	8.2E-12	157570
ESCO2	THZ-2-49	-0.14235	6.85E-05	157570
ESCO2	TL-1-85	-0.22724	3.01E-11	157570
ESCO2	TL-2-105	-0.16993	1.55E-06	157570
ESCO2	TPCA-1	-0.2753	2.21E-16	157570
ESCO2	TW 37	-0.04961	0.281295	157570
ESCO2	Talazoparib	0.024475	0.624256	157570
ESCO2	Tamoxifen	0.003127	0.986862	157570
ESCO2	Temozolomide	-0.00872	0.930296	157570
ESCO2	Temsirolimus	-0.11562	0.005966	157570
ESCO2	Thapsigargin	0.022163	0.776248	157570
ESCO2	Tipifarnib	0.005494	0.954484	157570
ESCO2	Tivozanib	-0.07476	0.132012	157570
ESCO2	Trametinib	0.234253	1.52E-11	157570
ESCO2	Tubastatin A	-0.18714	5.69E-08	157570
ESCO2	UNC0638	-0.17402	2.81E-07	157570
ESCO2	UNC1215	0.102889	0.021312	157570
ESCO2	VNLG/124	-0.12237	0.001297	157570
ESCO2	VX-11e	0.011937	0.842369	157570
ESCO2	VX-680	-0.13111	0.101798	157570
ESCO2	VX-702	-0.05429	0.754305	157570
ESCO2	Veliparib	-0.00584	0.951423	157570
ESCO2	Vinblastine	-0.07197	0.09127	157570
ESCO2	Vinorelbine	-0.08176	0.094414	157570
ESCO2	Vorinostat	-0.2615	9.72E-14	157570
ESCO2	WH-4-023	0.035115	0.687172	157570
ESCO2	WZ-1-84	0.012854	0.891967	157570
ESCO2	WZ3105	-0.19822	7.43E-09	157570
ESCO2	XAV939	0.065755	0.091684	157570
ESCO2	XL-184	-0.10451	0.01121	157570
ESCO2	XMD11-85h	0.001581	0.995823	157570

ESCO2	XMD13-2	-0.20419	2.77E-09	157570
ESCO2	XMD14-99	-0.09344	0.011694	157570
ESCO2	XMD15-27	-0.08442	0.05048	157570
ESCO2	XMD8-85	-0.05342	0.5344	157570
ESCO2	XMD8-92	-0.07006	0.487019	157570
ESCO2	Y-39983	-0.24247	2.58E-12	157570
ESCO2	YK 4-279	0.048055	0.370821	157570
ESCO2	YM155	-0.07663	0.10018	157570
ESCO2	YM201636	-0.21769	3.26E-10	157570
ESCO2	Z-LLNle-CHO	-0.05909	0.440015	157570
ESCO2	ZG-10	-0.13895	0.016507	157570
ESCO2	ZM-447439	-0.16311	4.97E-05	157570
ESCO2	ZSTK474	-0.22367	8.35E-11	157570
ESCO2	Zibotentan	-0.03369	0.902712	157570
ESCO2	piperlongumine	0.086524	0.033251	157570
ESCO2	rTRAIL	0.027592	0.734495	157570
ESCO2	selumetinib	0.192634	2.23E-08	157570
EXO1	(5Z)-7-Oxozeaenol	0.145931	7.8E-05	9156
EXO1	17-AAG	0.207567	6.4E-09	9156
EXO1	5-Fluorouracil	-0.07654	0.036706	9156
EXO1	681640	0.029579	0.647118	9156
EXO1	A-443654	-0.01405	0.957562	9156
EXO1	A-770041	0.073424	0.353124	9156
EXO1	AC220	-0.04606	0.335478	9156
EXO1	AG-014699	0.078753	0.056219	9156
EXO1	AICAR	-0.11814	0.002029	9156
EXO1	AKT inhibitor VIII	0.108575	0.009148	9156
EXO1	AMG-706	0.048629	0.409366	9156
EXO1	AP-24534	-0.03138	0.48041	9156
EXO1	AR-42	-0.16351	2.74E-06	9156
EXO1	AS601245	0.106226	0.019676	9156
EXO1	AS605240	-0.03327	0.469644	9156
EXO1	AT-7519	-0.1298	0.000216	9156
EXO1	ATRA	-0.04493	0.366508	9156
EXO1	AUY922	0.097111	0.031227	9156
EXO1	AZ628	0.115553	0.091781	9156
EXO1	AZD6482	0.066938	0.121473	9156
EXO1	AZD7762	-0.13248	0.000513	9156
EXO1	AZD8055	-0.08451	0.030607	9156
EXO1	Afatinib	0.129146	0.000276	9156
EXO1	Axitinib	-0.0703	0.146679	9156
EXO1	BAY 61-3606	-0.01575	0.734686	9156
EXO1	BEZ235	0.082252	0.065857	9156
EXO1	BHG712	-0.08799	0.014836	9156
EXO1	BI-2536	-0.04873	0.663175	9156
EXO1	BIRB 0796	0.067674	0.167859	9156
EXO1	BIX02189	-0.10688	0.002919	9156
EXO1	BMS-509744	0.026632	0.803116	9156
EXO1	BMS-536924	-0.01345	0.883851	9156
EXO1	BMS-708163	0.118779	0.002013	9156

EXO1	BMS-754807	0.047915	0.378101	9156
EXO1	BMS345541	-0.17257	6.55E-07	9156
EXO1	BX-795	-0.04279	0.364123	9156
EXO1	BX-912	-0.16368	2.14E-06	9156
EXO1	Belinostat	-0.13056	0.000303	9156
EXO1	Bexarotene	0.037223	0.632558	9156
EXO1	Bicalutamide	0.068807	0.108768	9156
EXO1	Bleomycin	0.115042	0.011966	9156
EXO1	Bleomycin (50 uM)	0.185365	1.06E-07	9156
EXO1	Bortezomib	0.095498	0.192104	9156
EXO1	Bosutinib	0.01948	0.761446	9156
EXO1	Bryostatin 1	0.090141	0.052954	9156
EXO1	CAL-101	-0.09066	0.014167	9156
EXO1	CAY10603	-0.14961	1.86E-05	9156
EXO1	CCT007093	0.083738	0.042202	9156
EXO1	CCT018159	0.060195	0.21858	9156
EXO1	CEP-701	-0.07638	0.056703	9156
EXO1	CGP-082996	0.017778	0.887581	9156
EXO1	CGP-60474	0.09857	0.200271	9156
EXO1	CH5424802	-0.06369	0.226259	9156
EXO1	CHIR-99021	0.090964	0.014486	9156
EXO1	CI-1040	0.125523	0.001548	9156
EXO1	CMK	0.040587	0.752835	9156
EXO1	CP466722	-0.17029	8.78E-07	9156
EXO1	CP724714	0.046404	0.385855	9156
EXO1	CUDC-101	-0.13218	0.000225	9156
EXO1	CX-5461	-0.13626	0.000127	9156
EXO1	Camptothecin	-0.05711	0.19228	9156
EXO1	Cetuximab	0.09414	0.016633	9156
EXO1	Cisplatin	-0.00231	0.972246	9156
EXO1	Crizotinib	-0.02566	0.884085	9156
EXO1	Cyclopamine	0.007628	0.959289	9156
EXO1	Cytarabine	-0.01773	0.765848	9156
EXO1	DMOG	-0.05	0.271538	9156
EXO1	Dabrafenib	0.094932	0.018135	9156
EXO1	Dasatinib	0.130499	0.038385	9156
EXO1	Docetaxel	0.160367	1.15E-05	9156
EXO1	Doxorubicin	0.049621	0.42259	9156
EXO1	EHT 1864	0.004564	0.96217	9156
EXO1	EKB-569	-0.05856	0.1455	9156
EXO1	EX-527	-0.02801	0.814851	9156
EXO1	Elesclomol	0.008509	0.87391	9156
EXO1	Embelin	0.056174	0.31282	9156
EXO1	Epothilone B	0.080427	0.089084	9156
EXO1	Erlotinib	0.176538	0.006947	9156
EXO1	Etoposide	-0.05495	0.237902	9156
EXO1	FH535	0.098398	0.021402	9156
EXO1	FK866	-0.17839	3.28E-07	9156
EXO1	FMK	0.003083	0.973787	9156
EXO1	FR-180204	-0.02457	0.676046	9156

EXO1	FTI-277	0.207706	4.71E-08	9156
EXO1	Foretinib	-0.06386	0.116258	9156
EXO1	GDC0449	0.030488	0.778682	9156
EXO1	GDC0941	-7.1E-05	0.999317	9156
EXO1	GNF-2	-0.0384	0.885254	9156
EXO1	GSK-650394	0.038833	0.602863	9156
EXO1	GSK1070916	-0.19441	2.63E-08	9156
EXO1	GSK1904529A	0.069271	0.117618	9156
EXO1	GSK2126458	-0.09311	0.011493	9156
EXO1	GSK269962A	0.050494	0.316445	9156
EXO1	GSK429286A	-0.10603	0.005752	9156
EXO1	GSK690693	-0.14398	4.89E-05	9156
EXO1	GW 441756	-0.02902	0.896458	9156
EXO1	GW-2580	0.022289	0.967411	9156
EXO1	GW843682X	-0.07563	0.377327	9156
EXO1	Gefitinib	0.130066	0.000829	9156
EXO1	Gemcitabine	-0.04039	0.428637	9156
EXO1	Genentech Cpd 10	-0.17069	1.06E-06	9156
EXO1	HG-5-113-01	-0.05143	0.485712	9156
EXO1	HG-5-88-01	-0.00151	0.995636	9156
EXO1	HG-6-64-1	0.08027	0.066759	9156
EXO1	I-BET-762	-0.17902	1.6E-07	9156
EXO1	IOX2	0.049379	0.382885	9156
EXO1	IPA-3	-0.06727	0.088491	9156
EXO1	Imatinib	-0.05941	0.698591	9156
EXO1	Ispinesib Mesylate	-0.10751	0.002805	9156
EXO1	JNJ-26854165	0.086147	0.057102	9156
EXO1	JNK Inhibitor VIII	0.068622	0.10275	9156
EXO1	JNK-9L	0.038324	0.51515	9156
EXO1	JQ1	0.010657	0.83922	9156
EXO1	JQ12	0.003939	0.95622	9156
EXO1	JW-7-24-1	-0.15386	9.85E-06	9156
EXO1	JW-7-52-1	0.038223	0.733786	9156
EXO1	KIN001-055	0.020279	0.794578	9156
EXO1	KIN001-102	-0.174	4.51E-07	9156
EXO1	KIN001-135	0.055405	0.603347	9156
EXO1	KIN001-236	-0.0833	0.023492	9156
EXO1	KIN001-244	-0.12107	0.000726	9156
EXO1	KIN001-260	-0.14173	5.87E-05	9156
EXO1	KIN001-266	-0.06199	0.17688	9156
EXO1	KIN001-270	-0.10691	0.004446	9156
EXO1	KU-55933	-0.0352	0.589817	9156
EXO1	LAQ824	-0.10177	0.00786	9156
EXO1	LFM-A13	0.098878	0.030133	9156
EXO1	LY317615	-0.06591	0.121479	9156
EXO1	Lapatinib	0.221989	0.000113	9156
EXO1	Lenalidomide	-0.03774	0.63016	9156
EXO1	Linifanib	0.002536	0.971884	9156
EXO1	Lisitinib	0.010948	0.89956	9156
EXO1	MG-132	0.077798	0.358661	9156

EXO1	MK-2206	-0.06786	0.166967	9156
EXO1	MLN4924	0.091493	0.076703	9156
EXO1	MP470	-0.09907	0.017384	9156
EXO1	MPS-1-IN-1	-0.09752	0.008189	9156
EXO1	MS-275	-0.15496	0.026566	9156
EXO1	Masitinib	-0.11332	0.001865	9156
EXO1	Methotrexate	-0.15573	1.58E-05	9156
EXO1	Midostaurin	0.104463	0.011211	9156
EXO1	Mitomycin C	-0.05687	0.250777	9156
EXO1	NG-25	-0.13068	0.000202	9156
EXO1	NPK76-II-72-1	-0.23935	1.16E-12	9156
EXO1	NSC-207895	-0.11514	0.003989	9156
EXO1	NSC-87877	0.104339	0.038038	9156
EXO1	NU-7441	0.008123	0.942099	9156
EXO1	Navitoclax	-0.2226	3.67E-10	9156
EXO1	Nilotinib	-0.07672	0.100926	9156
EXO1	Nutlin-3a (-)	0.063243	0.16866	9156
EXO1	OSI-027	-0.13572	0.000122	9156
EXO1	OSI-930	-0.05506	0.161704	9156
EXO1	OSU-03012	0.033945	0.519155	9156
EXO1	Obatoclax Mesylate	0.031763	0.530298	9156
EXO1	Olaparib	0.047491	0.28182	9156
EXO1	PAC-1	-0.08759	0.029928	9156
EXO1	PD-0325901	0.186023	5.56E-07	9156
EXO1	PD-0332991	0.042061	0.458376	9156
EXO1	PD-173074	-0.00402	0.990057	9156
EXO1	PF-4708671	0.03687	0.732407	9156
EXO1	PF-562271	0.076546	0.137187	9156
EXO1	PFI-1	0.030914	0.553264	9156
EXO1	PHA-665752	0.099052	0.437156	9156
EXO1	PHA-793887	-0.14593	2.42E-05	9156
EXO1	PI-103	-0.14005	7.18E-05	9156
EXO1	PIK-93	-0.17096	6.67E-07	9156
EXO1	PLX4720	0.077459	0.052465	9156
EXO1	Paclitaxel	0.055044	0.632577	9156
EXO1	Parthenolide	-0.01887	0.883671	9156
EXO1	Pazopanib	0.060294	0.248094	9156
EXO1	Phenformin	-0.08568	0.020093	9156
EXO1	Pyrimethamine	-0.03182	0.803733	9156
EXO1	QL-VIII-58	0.078444	0.268045	9156
EXO1	QL-X-138	-0.14202	6.35E-05	9156
EXO1	QL-XI-92	-0.13742	9.67E-05	9156
EXO1	QL-XII-47	-0.0789	0.042639	9156
EXO1	QL-XII-61	-0.1158	0.052437	9156
EXO1	QS11	-0.03679	0.571279	9156
EXO1	RDEA119	0.218508	1.51E-10	9156
EXO1	RO-3306	0.110124	0.006419	9156
EXO1	Rapamycin	0.024527	0.873416	9156
EXO1	Roscovitine	0.097663	0.499986	9156
EXO1	Ruxolitinib	-0.08622	0.052735	9156

EXO1	S-Trityl-L-cysteine	-0.02715	0.7807	9156
EXO1	SB 216763	0.082642	0.081499	9156
EXO1	SB 505124	0.061681	0.36184	9156
EXO1	SB52334	-0.07872	0.065474	9156
EXO1	SB590885	0.100091	0.02452	9156
EXO1	SGC0946	0.05504	0.280616	9156
EXO1	SL 0101-1	0.040492	0.601897	9156
EXO1	SN-38	0.035933	0.443731	9156
EXO1	SNX-2112	-0.08919	0.013886	9156
EXO1	STF-62247	-0.05392	0.18465	9156
EXO1	Salubrinal	-0.00724	0.950053	9156
EXO1	Saracatinib	0.028599	0.761962	9156
EXO1	Shikonin	-0.01679	0.792984	9156
EXO1	Sorafenib	0.028942	0.823316	9156
EXO1	Sunitinib	-0.02363	0.793927	9156
EXO1	T0901317	-0.06636	0.099632	9156
EXO1	TAE684	0.016208	0.918327	9156
EXO1	TAK-715	-0.12845	0.000285	9156
EXO1	TG101348	-0.14711	2.45E-05	9156
EXO1	TGX221	0.203932	0.000486	9156
EXO1	THZ-2-102-1	-0.16086	4.6E-06	9156
EXO1	THZ-2-49	-0.09924	0.006384	9156
EXO1	TL-1-85	-0.13389	0.000143	9156
EXO1	TL-2-105	-0.13051	0.00028	9156
EXO1	TPCA-1	-0.18877	3.56E-08	9156
EXO1	TW 37	-0.05391	0.235837	9156
EXO1	Talazoparib	-0.01671	0.749309	9156
EXO1	Tamoxifen	0.071956	0.439415	9156
EXO1	Temozolomide	0.024317	0.76604	9156
EXO1	Temsirolimus	0.010206	0.86776	9156
EXO1	Thapsigargin	0.03028	0.673224	9156
EXO1	Tipifarnib	0.052784	0.394784	9156
EXO1	Tivozanib	-0.02948	0.643025	9156
EXO1	Trametinib	0.26486	1.65E-14	9156
EXO1	Tubastatin A	-0.12302	0.000474	9156
EXO1	UNC0638	-0.13129	0.000136	9156
EXO1	UNC1215	0.0737	0.11441	9156
EXO1	VNLG/124	-0.05348	0.198273	9156
EXO1	VX-11e	0.075849	0.084884	9156
EXO1	VX-680	-0.05988	0.528757	9156
EXO1	VX-702	-0.0218	0.933016	9156
EXO1	Veliparib	0.019751	0.806517	9156
EXO1	Vinblastine	-0.01307	0.806137	9156
EXO1	Vinorelbine	0.00014	0.998867	9156
EXO1	Vorinostat	-0.22048	4.97E-10	9156
EXO1	WH-4-023	0.110859	0.118896	9156
EXO1	WZ-1-84	0.098805	0.17811	9156
EXO1	WZ3105	-0.16107	3.31E-06	9156
EXO1	XAV939	0.092967	0.014789	9156
EXO1	XL-184	0.01273	0.822218	9156

EXO1	XMD11-85h	0.032283	0.86628	9156
EXO1	XMD13-2	-0.12742	0.000296	9156
EXO1	XMD14-99	-0.0696	0.066203	9156
EXO1	XMD15-27	-0.0474	0.316493	9156
EXO1	XMD8-85	0.069419	0.396889	9156
EXO1	XMD8-92	0.022778	0.86601	9156
EXO1	Y-39983	-0.13075	0.000274	9156
EXO1	YK 4-279	0.06465	0.200146	9156
EXO1	YM155	-0.04404	0.398876	9156
EXO1	YM201636	-0.17635	4.72E-07	9156
EXO1	Z-LLNle-CHO	0.077103	0.291813	9156
EXO1	ZG-10	-0.07024	0.281155	9156
EXO1	ZM-447439	-0.09579	0.024134	9156
EXO1	ZSTK474	-0.12845	0.000294	9156
EXO1	Zibotentan	-0.02039	0.951546	9156
EXO1	piperlongumine	0.097919	0.014349	9156
EXO1	rTRAIL	0.026557	0.743958	9156
EXO1	selumetinib	0.218445	1.64E-10	9156
KIF11	(5Z)-7-Oxozeaenol	0.1212	0.00127	3832
KIF11	17-AAG	0.194839	5.51E-08	3832
KIF11	5-Fluorouracil	-0.07186	0.050752	3832
KIF11	681640	8.3E-05	0.999077	3832
KIF11	A-443654	-0.10501	0.454239	3832
KIF11	A-770041	-0.07631	0.33127	3832
KIF11	AC220	-0.03816	0.443804	3832
KIF11	AG-014699	0.040735	0.355235	3832
KIF11	AICAR	-0.15861	2.55E-05	3832
KIF11	AKT inhibitor VIII	0.089029	0.036042	3832
KIF11	AMG-706	0.063138	0.251119	3832
KIF11	AP-24534	-0.10008	0.010509	3832
KIF11	AR-42	-0.1633	2.83E-06	3832
KIF11	AS601245	0.06725	0.182231	3832
KIF11	AS605240	-0.04318	0.329973	3832
KIF11	AT-7519	-0.17451	4.58E-07	3832
KIF11	ATRA	-0.05918	0.213506	3832
KIF11	AUY922	0.020203	0.742816	3832
KIF11	AZ628	0.127697	0.05716	3832
KIF11	AZD6482	0.01606	0.78212	3832
KIF11	AZD7762	-0.19173	2.54E-07	3832
KIF11	AZD8055	-0.13752	0.000284	3832
KIF11	Afatinib	0.118387	0.000929	3832
KIF11	Axitinib	-0.075	0.116762	3832
KIF11	BAY 61-3606	-0.06932	0.087199	3832
KIF11	BEZ235	-0.01056	0.861065	3832
KIF11	BHG712	-0.11453	0.001282	3832
KIF11	BI-2536	-0.16424	0.054445	3832
KIF11	BIRB 0796	0.045407	0.383176	3832
KIF11	BIX02189	-0.1493	2.26E-05	3832
KIF11	BMS-509744	-0.02184	0.841996	3832
KIF11	BMS-536924	-0.0315	0.677069	3832

KIF11	BMS-708163	0.101082	0.00919	3832
KIF11	BMS-754807	0.018752	0.77131	3832
KIF11	BMS345541	-0.18639	6.89E-08	3832
KIF11	BX-795	-0.12555	0.001912	3832
KIF11	BX-912	-0.22658	2.49E-11	3832
KIF11	Belinostat	-0.1329	0.000232	3832
KIF11	Bexarotene	0.019052	0.838614	3832
KIF11	Bicalutamide	0.018946	0.709209	3832
KIF11	Bleomycin	0.058193	0.26957	3832
KIF11	Bleomycin (50 uM)	0.097645	0.006535	3832
KIF11	Bortezomib	0.009483	0.929625	3832
KIF11	Bosutinib	-0.05966	0.233649	3832
KIF11	Bryostatin 1	0.080308	0.090569	3832
KIF11	CAL-101	-0.13893	0.000111	3832
KIF11	CAY10603	-0.16262	2.95E-06	3832
KIF11	CCT007093	0.090255	0.027572	3832
KIF11	CCT018159	0.006989	0.919656	3832
KIF11	CEP-701	-0.12818	0.000832	3832
KIF11	CGP-082996	-0.0483	0.633861	3832
KIF11	CGP-60474	-0.05229	0.562536	3832
KIF11	CH5424802	-0.01954	0.785782	3832
KIF11	CHIR-99021	0.027462	0.507218	3832
KIF11	CI-1040	0.138291	0.000428	3832
KIF11	CMK	-0.0517	0.672193	3832
KIF11	CP466722	-0.20578	1.93E-09	3832
KIF11	CP724714	0.050859	0.331439	3832
KIF11	CUDC-101	-0.1516	2.01E-05	3832
KIF11	CX-5461	-0.16619	2.25E-06	3832
KIF11	Camptothecin	-0.1234	0.001854	3832
KIF11	Cetuximab	0.096534	0.013774	3832
KIF11	Cisplatin	-0.02661	0.624402	3832
KIF11	Crizotinib	-0.05773	0.693397	3832
KIF11	Cyclopamine	-0.03896	0.750471	3832
KIF11	Cytarabine	-0.07091	0.133483	3832
KIF11	DMOG	-0.0947	0.021995	3832
KIF11	Dabrafenib	0.143169	0.000189	3832
KIF11	Dasatinib	0.01834	0.814425	3832
KIF11	Docetaxel	0.083546	0.025936	3832
KIF11	Doxorubicin	-0.01476	0.852318	3832
KIF11	EHT 1864	0.045364	0.482634	3832
KIF11	EKB-569	-0.07697	0.049497	3832
KIF11	EX-527	-0.01415	0.916277	3832
KIF11	Elesclomol	-0.06365	0.14516	3832
KIF11	Embelin	0.019161	0.791756	3832
KIF11	Epothilone B	0.015249	0.802694	3832
KIF11	Erlotinib	0.132236	0.053261	3832
KIF11	Etoposide	-0.11648	0.005118	3832
KIF11	FH535	0.117635	0.004877	3832
KIF11	FK866	-0.19869	1.07E-08	3832
KIF11	FMK	-0.02941	0.69309	3832

KIF11	FR-180204	-0.01238	0.85295	3832
KIF11	FTI-277	0.169079	1.12E-05	3832
KIF11	Foretinib	-0.14935	5.04E-05	3832
KIF11	GDC0449	0.008591	0.95433	3832
KIF11	GDC0941	-0.08483	0.073073	3832
KIF11	GNF-2	-0.02371	0.934879	3832
KIF11	GSK-650394	-0.02839	0.728373	3832
KIF11	GSK1070916	-0.22344	1.14E-10	3832
KIF11	GSK1904529A	0.065254	0.142991	3832
KIF11	GSK2126458	-0.14552	4.86E-05	3832
KIF11	GSK269962A	-0.01239	0.850104	3832
KIF11	GSK429286A	-0.14711	7.55E-05	3832
KIF11	GSK690693	-0.1865	9.12E-08	3832
KIF11	GW 441756	-0.0427	0.813754	3832
KIF11	GW-2580	-0.00721	0.994893	3832
KIF11	GW843682X	-0.184	0.011526	3832
KIF11	Gefitinib	0.112212	0.004423	3832
KIF11	Gemcitabine	-0.08653	0.054267	3832
KIF11	Genentech Cpd 10	-0.17642	4.34E-07	3832
KIF11	HG-5-113-01	-0.12013	0.055758	3832
KIF11	HG-5-88-01	-0.02471	0.894445	3832
KIF11	HG-6-64-1	-0.00836	0.890703	3832
KIF11	I-BET-762	-0.20074	3.54E-09	3832
KIF11	IOX2	0.002906	0.973893	3832
KIF11	IPA-3	-0.12893	0.000605	3832
KIF11	Imatinib	-0.05539	0.72413	3832
KIF11	Ispinesib Mesylate	-0.12426	0.00049	3832
KIF11	JNJ-26854165	0.012411	0.847133	3832
KIF11	JNK Inhibitor VIII	0.067372	0.109662	3832
KIF11	JNK-9L	0.013105	0.856248	3832
KIF11	JQ1	-0.0297	0.515735	3832
KIF11	JQ12	0.007011	0.919821	3832
KIF11	JW-7-24-1	-0.16693	1.47E-06	3832
KIF11	JW-7-52-1	-0.03665	0.745369	3832
KIF11	KIN001-055	0.028125	0.694522	3832
KIF11	KIN001-102	-0.17653	3E-07	3832
KIF11	KIN001-135	0.078957	0.415169	3832
KIF11	KIN001-236	-0.1132	0.001723	3832
KIF11	KIN001-244	-0.14692	3.42E-05	3832
KIF11	KIN001-260	-0.12597	0.000386	3832
KIF11	KIN001-266	-0.05102	0.281696	3832
KIF11	KIN001-270	-0.137	0.000213	3832
KIF11	KU-55933	-0.08965	0.076232	3832
KIF11	LAQ824	-0.10207	0.007669	3832
KIF11	LFM-A13	0.058927	0.228607	3832
KIF11	LY317615	-0.07112	0.091009	3832
KIF11	Lapatinib	0.17385	0.003335	3832
KIF11	Lenalidomide	-0.08254	0.161554	3832
KIF11	Linifanib	-0.02135	0.725158	3832
KIF11	Lisitinib	0.007625	0.93396	3832

KIF11	MG-132	0.036967	0.7149	3832
KIF11	MK-2206	-0.12777	0.004392	3832
KIF11	MLN4924	0.06482	0.234013	3832
KIF11	MP470	-0.07604	0.070958	3832
KIF11	MPS-1-IN-1	-0.1137	0.001852	3832
KIF11	MS-275	-0.16643	0.016438	3832
KIF11	Masitinib	-0.09748	0.008054	3832
KIF11	Methotrexate	-0.17572	9.55E-07	3832
KIF11	Midostaurin	0.01087	0.838874	3832
KIF11	Mitomycin C	-0.0595	0.224716	3832
KIF11	NG-25	-0.1592	4.74E-06	3832
KIF11	NPK76-II-72-1	-0.26457	2.74E-15	3832
KIF11	NSC-207895	-0.17044	1.29E-05	3832
KIF11	NSC-87877	0.133587	0.007691	3832
KIF11	NU-7441	-0.02831	0.728308	3832
KIF11	Navitoclax	-0.25002	1.35E-12	3832
KIF11	Nilotinib	-0.05789	0.241742	3832
KIF11	Nutlin-3a (-)	0.032218	0.533579	3832
KIF11	OSI-027	-0.16406	2.77E-06	3832
KIF11	OSI-930	-0.07542	0.049262	3832
KIF11	OSU-03012	-0.05118	0.297062	3832
KIF11	Obatoclax Mesylate	0.004327	0.944967	3832
KIF11	Olaparib	0.02074	0.671067	3832
KIF11	PAC-1	-0.0973	0.01485	3832
KIF11	PD-0325901	0.210592	1.14E-08	3832
KIF11	PD-0332991	-0.02296	0.722642	3832
KIF11	PD-173074	-0.03813	0.803092	3832
KIF11	PF-4708671	0.073525	0.387433	3832
KIF11	PF-562271	0.0123	0.858693	3832
KIF11	PFI-1	9.43E-06	0.999827	3832
KIF11	PHA-665752	0.065402	0.646797	3832
KIF11	PHA-793887	-0.18057	1.34E-07	3832
KIF11	PI-103	-0.16632	1.98E-06	3832
KIF11	PIK-93	-0.22134	7.08E-11	3832
KIF11	PLX4720	0.151805	3.37E-05	3832
KIF11	Paclitaxel	-0.05003	0.668448	3832
KIF11	Parthenolide	-0.06351	0.535393	3832
KIF11	Pazopanib	0.014235	0.829906	3832
KIF11	Phenformin	-0.09144	0.012787	3832
KIF11	Pyrimethamine	-0.00776	0.961528	3832
KIF11	QL-VIII-58	0.011881	0.911336	3832
KIF11	QL-X-138	-0.18775	7.69E-08	3832
KIF11	QL-XI-92	-0.18131	1.8E-07	3832
KIF11	QL-XII-47	-0.11724	0.001878	3832
KIF11	QL-XII-61	-0.17538	0.001741	3832
KIF11	QS11	-0.06831	0.225902	3832
KIF11	RDEA119	0.230607	1.19E-11	3832
KIF11	RO-3306	0.047737	0.265299	3832
KIF11	Rapamycin	-0.07976	0.464856	3832
KIF11	Roscovitine	0.065492	0.682476	3832

KIF11	Ruxolitinib	-0.05118	0.302382	3832
KIF11	S-Trityl-L-cysteine	-0.09835	0.193812	3832
KIF11	SB 216763	0.039746	0.438419	3832
KIF11	SB 505124	0.045706	0.529484	3832
KIF11	SB52334	0.008269	0.895592	3832
KIF11	SB590885	0.160359	7.33E-05	3832
KIF11	SGC0946	0.051638	0.317236	3832
KIF11	SL 0101-1	0.081037	0.268103	3832
KIF11	SN-38	-0.03543	0.450997	3832
KIF11	SNX-2112	-0.16503	2.45E-06	3832
KIF11	STF-62247	-0.09276	0.017311	3832
KIF11	Salubrinal	-0.06486	0.447009	3832
KIF11	Saracatinib	-0.05355	0.535949	3832
KIF11	Shikonin	-0.07768	0.107547	3832
KIF11	Sorafenib	-0.01006	0.944078	3832
KIF11	Sunitinib	-0.10044	0.147755	3832
KIF11	T0901317	-0.13436	0.000375	3832
KIF11	TAE684	-0.03008	0.824879	3832
KIF11	TAK-715	-0.1221	0.000583	3832
KIF11	TG101348	-0.19233	2.21E-08	3832
KIF11	TGX221	0.123575	0.045955	3832
KIF11	THZ-2-102-1	-0.17607	4.67E-07	3832
KIF11	THZ-2-49	-0.06543	0.079136	3832
KIF11	TL-1-85	-0.14424	3.93E-05	3832
KIF11	TL-2-105	-0.12667	0.000435	3832
KIF11	TPCA-1	-0.20558	1.65E-09	3832
KIF11	TW 37	-0.087	0.036407	3832
KIF11	Talazoparib	-0.0217	0.668762	3832
KIF11	Tamoxifen	-0.01635	0.912377	3832
KIF11	Temozolomide	0.016893	0.849269	3832
KIF11	Temsirolimus	-0.10127	0.018164	3832
KIF11	Thapsigargin	0.015248	0.857027	3832
KIF11	Tipifarnib	0.019586	0.806928	3832
KIF11	Tivozanib	-0.05638	0.297009	3832
KIF11	Trametinib	0.273436	0	3832
KIF11	Tubastatin A	-0.15126	1.42E-05	3832
KIF11	UNC0638	-0.11238	0.001211	3832
KIF11	UNC1215	0.077895	0.092808	3832
KIF11	VNLG/124	-0.05946	0.147399	3832
KIF11	VX-11e	0.054333	0.243034	3832
KIF11	VX-680	-0.14399	0.064655	3832
KIF11	VX-702	-0.02456	0.921524	3832
KIF11	Veliparib	0.003577	0.970558	3832
KIF11	Vinblastine	-0.08044	0.055867	3832
KIF11	Vinorelbine	-0.05286	0.320784	3832
KIF11	Vorinostat	-0.24015	1.01E-11	3832
KIF11	WH-4-023	0.027675	0.759817	3832
KIF11	WZ-1-84	0.031321	0.721403	3832
KIF11	WZ3105	-0.15731	5.72E-06	3832
KIF11	XAV939	0.045744	0.253525	3832

KIF11	XL-184	-0.07155	0.102566	3832
KIF11	XMD11-85h	-0.01146	0.964062	3832
KIF11	XMD13-2	-0.15799	5.73E-06	3832
KIF11	XMD14-99	-0.0651	0.087756	3832
KIF11	XMD15-27	-0.09153	0.031613	3832
KIF11	XMD8-85	-0.02498	0.802484	3832
KIF11	XMD8-92	-0.06628	0.51877	3832
KIF11	Y-39983	-0.18602	1.25E-07	3832
KIF11	YK 4-279	0.022852	0.713653	3832
KIF11	YM155	-0.08466	0.063362	3832
KIF11	YM201636	-0.14852	2.65E-05	3832
KIF11	Z-LLNle-CHO	-0.00505	0.956894	3832
KIF11	ZG-10	-0.13958	0.015967	3832
KIF11	ZM-447439	-0.14074	0.000529	3832
KIF11	ZSTK474	-0.16887	1.39E-06	3832
KIF11	Zibotentan	-0.00728	0.988489	3832
KIF11	piperlongumine	0.022595	0.646484	3832
KIF11	rTRAIL	0.043841	0.548462	3832
KIF11	selumetinib	0.242821	8.69E-13	3832
KIF15	(5Z)-7-Oxozeaenol	0.101235	0.008149	56992
KIF15	17-AAG	0.218268	9.36E-10	56992
KIF15	5-Fluorouracil	-0.1767	4.55E-07	56992
KIF15	681640	0.026795	0.682	56992
KIF15	A-443654	-0.05727	0.733808	56992
KIF15	A-770041	0.02994	0.7528	56992
KIF15	AC220	-0.07924	0.066771	56992
KIF15	AG-014699	0.032746	0.470468	56992
KIF15	AICAR	-0.15682	3.16E-05	56992
KIF15	AKT inhibitor VIII	0.123149	0.002812	56992
KIF15	AMG-706	0.088276	0.082127	56992
KIF15	AP-24534	-0.13863	0.000265	56992
KIF15	AR-42	-0.22685	3.82E-11	56992
KIF15	AS601245	0.095578	0.039155	56992
KIF15	AS605240	-0.06198	0.140184	56992
KIF15	AT-7519	-0.23685	3.52E-12	56992
KIF15	ATRA	-0.11422	0.00705	56992
KIF15	AUY922	0.01366	0.836377	56992
KIF15	AZ628	0.102044	0.145717	56992
KIF15	AZD6482	0.045651	0.327696	56992
KIF15	AZD7762	-0.22194	1.65E-09	56992
KIF15	AZD8055	-0.1535	4.57E-05	56992
KIF15	Afatinib	0.211527	6.79E-10	56992
KIF15	Axitinib	-0.11805	0.007798	56992
KIF15	BAY 61-3606	-0.09915	0.011635	56992
KIF15	BEZ235	0.016531	0.773965	56992
KIF15	BHG712	-0.1873	6.36E-08	56992
KIF15	BI-2536	-0.1682	0.049014	56992
KIF15	BIRB 0796	0.103237	0.026117	56992
KIF15	BIX02189	-0.21229	8.05E-10	56992
KIF15	BMS-509744	0.011558	0.920399	56992

KIF15	BMS-536924	-0.01112	0.90892	56992
KIF15	BMS-708163	0.122044	0.001483	56992
KIF15	BMS-754807	0.019426	0.761622	56992
KIF15	BMS345541	-0.25247	1.18E-13	56992
KIF15	BX-795	-0.16145	3.98E-05	56992
KIF15	BX-912	-0.3005	1.85E-19	56992
KIF15	Belinostat	-0.1972	2.42E-08	56992
KIF15	Bexarotene	0.009966	0.92788	56992
KIF15	Bicalutamide	0.097307	0.018425	56992
KIF15	Bleomycin	0.032494	0.58861	56992
KIF15	Bleomycin (50 uM)	0.123022	0.000536	56992
KIF15	Bortezomib	0.016565	0.873037	56992
KIF15	Bosutinib	-0.05043	0.335371	56992
KIF15	Bryostatin 1	0.109461	0.016459	56992
KIF15	CAL-101	-0.18276	2.23E-07	56992
KIF15	CAY10603	-0.23254	1.02E-11	56992
KIF15	CCT007093	0.148121	0.000183	56992
KIF15	CCT018159	-0.01239	0.84947	56992
KIF15	CEP-701	-0.17397	3.42E-06	56992
KIF15	CGP-082996	0.019328	0.875855	56992
KIF15	CGP-60474	-0.00746	0.950247	56992
KIF15	CH5424802	-0.07512	0.135237	56992
KIF15	CHIR-99021	0.079525	0.034308	56992
KIF15	CI-1040	0.121513	0.00226	56992
KIF15	CMK	-0.00611	0.968588	56992
KIF15	CP466722	-0.2639	5.79E-15	56992
KIF15	CP724714	0.090913	0.045114	56992
KIF15	CUDC-101	-0.19923	1.26E-08	56992
KIF15	CX-5461	-0.26105	2.62E-14	56992
KIF15	Camptothecin	-0.1734	7.05E-06	56992
KIF15	Cetuximab	0.179406	1.5E-06	56992
KIF15	Cisplatin	-0.03499	0.501289	56992
KIF15	Crizotinib	-0.07872	0.527778	56992
KIF15	Cyclopamine	0.004198	0.976891	56992
KIF15	Cytarabine	-0.09946	0.025057	56992
KIF15	DMOG	-0.10582	0.009391	56992
KIF15	Dabrafenib	0.125738	0.001213	56992
KIF15	Dasatinib	0.104685	0.105206	56992
KIF15	Docetaxel	0.110752	0.002803	56992
KIF15	Doxorubicin	-0.0133	0.869691	56992
KIF15	EHT 1864	0.063162	0.290838	56992
KIF15	EKB-569	-0.10831	0.004319	56992
KIF15	EX-527	0.001064	0.99399	56992
KIF15	Elesclomol	-0.01441	0.781698	56992
KIF15	Embelin	0.011954	0.879649	56992
KIF15	Epothilone B	0.034089	0.528123	56992
KIF15	Erlotinib	0.20391	0.001414	56992
KIF15	Etoposide	-0.14868	0.000253	56992
KIF15	FH535	0.109011	0.009848	56992
KIF15	FK866	-0.27501	7.88E-16	56992

KIF15	FMK	-0.04257	0.536073	56992
KIF15	FR-180204	-0.06159	0.20693	56992
KIF15	FTI-277	0.189088	7.21E-07	56992
KIF15	Foretinib	-0.16711	4.84E-06	56992
KIF15	GDC0449	-0.02384	0.839154	56992
KIF15	GDC0941	-0.0552	0.296833	56992
KIF15	GNF-2	-0.02543	0.930464	56992
KIF15	GSK-650394	-0.03568	0.640796	56992
KIF15	GSK1070916	-0.31527	1.07E-20	56992
KIF15	GSK1904529A	0.126009	0.002442	56992
KIF15	GSK2126458	-0.18805	9.35E-08	56992
KIF15	GSK269962A	-0.02649	0.649848	56992
KIF15	GSK429286A	-0.19943	4.07E-08	56992
KIF15	GSK690693	-0.2333	1.28E-11	56992
KIF15	GW 441756	-0.02231	0.929412	56992
KIF15	GW-2580	0.006827	0.994893	56992
KIF15	GW843682X	-0.19476	0.007252	56992
KIF15	Gefitinib	0.195264	1.89E-07	56992
KIF15	Gemcitabine	-0.10086	0.021472	56992
KIF15	Genentech Cpd 10	-0.25096	2.27E-13	56992
KIF15	HG-5-113-01	-0.15861	0.008165	56992
KIF15	HG-5-88-01	-0.0337	0.846066	56992
KIF15	HG-6-64-1	-0.01952	0.728135	56992
KIF15	I-BET-762	-0.26903	9.27E-16	56992
KIF15	IOX2	0.052725	0.346861	56992
KIF15	IPA-3	-0.14362	0.00012	56992
KIF15	Imatinib	-0.08449	0.488468	56992
KIF15	Ispinesib Mesylate	-0.18345	1.4E-07	56992
KIF15	JNJ-26854165	0.059327	0.223887	56992
KIF15	JNK Inhibitor VIII	0.135037	0.000651	56992
KIF15	JNK-9L	-0.01279	0.859963	56992
KIF15	JQ1	-0.0357	0.426526	56992
KIF15	JQ12	-0.00848	0.902805	56992
KIF15	JW-7-24-1	-0.21412	3.85E-10	56992
KIF15	JW-7-52-1	-0.02138	0.866158	56992
KIF15	KIN001-055	0.049922	0.403179	56992
KIF15	KIN001-102	-0.24926	1.86E-13	56992
KIF15	KIN001-135	0.14007	0.102464	56992
KIF15	KIN001-236	-0.18889	6.83E-08	56992
KIF15	KIN001-244	-0.2129	8.87E-10	56992
KIF15	KIN001-260	-0.17942	2.62E-07	56992
KIF15	KIN001-266	-0.08628	0.050374	56992
KIF15	KIN001-270	-0.16164	1.01E-05	56992
KIF15	KU-55933	-0.09838	0.047271	56992
KIF15	LAQ824	-0.13863	0.000211	56992
KIF15	LFM-A13	0.116001	0.00978	56992
KIF15	LY317615	-0.09303	0.022162	56992
KIF15	Lapatinib	0.233957	4.27E-05	56992
KIF15	Lenalidomide	-0.03047	0.719319	56992
KIF15	Linifanib	-0.04507	0.383779	56992

KIF15	Lisitinib	0.020265	0.791814	56992
KIF15	MG-132	0.044917	0.642838	56992
KIF15	MK-2206	-0.0917	0.050711	56992
KIF15	MLN4924	0.040072	0.497762	56992
KIF15	MP470	-0.07883	0.060882	56992
KIF15	MPS-1-IN-1	-0.18532	1.76E-07	56992
KIF15	MS-275	-0.17383	0.012058	56992
KIF15	Masitinib	-0.16527	3.4E-06	56992
KIF15	Methotrexate	-0.24978	1.28E-12	56992
KIF15	Midostaurin	0.048678	0.281641	56992
KIF15	Mitomycin C	-0.07279	0.123206	56992
KIF15	NG-25	-0.2144	3.81E-10	56992
KIF15	NPK76-II-72-1	-0.36019	2.25E-28	56992
KIF15	NSC-207895	-0.15581	7.07E-05	56992
KIF15	NSC-87877	0.113385	0.024213	56992
KIF15	NU-7441	-0.03819	0.611799	56992
KIF15	Navitoclax	-0.3154	8.83E-20	56992
KIF15	Nilotinib	-0.10934	0.012849	56992
KIF15	Nutlin-3a (-)	0.042446	0.390617	56992
KIF15	OSI-027	-0.21349	6.31E-10	56992
KIF15	OSI-930	-0.13219	0.000318	56992
KIF15	OSU-03012	-0.05697	0.2353	56992
KIF15	Obatoclax Mesylate	-0.03568	0.472578	56992
KIF15	Olaparib	-0.01786	0.719583	56992
KIF15	PAC-1	-0.14583	0.000152	56992
KIF15	PD-0325901	0.222417	1.49E-09	56992
KIF15	PD-0332991	-0.04809	0.382963	56992
KIF15	PD-173074	-0.05196	0.700153	56992
KIF15	PF-4708671	0.071488	0.400451	56992
KIF15	PF-562271	0.015348	0.817749	56992
KIF15	PFI-1	0.008001	0.897049	56992
KIF15	PHA-665752	0.070181	0.616371	56992
KIF15	PHA-793887	-0.24668	2.43E-13	56992
KIF15	PI-103	-0.22137	1.29E-10	56992
KIF15	PIK-93	-0.29811	3.42E-19	56992
KIF15	PLX4720	0.130884	0.000443	56992
KIF15	Paclitaxel	-0.05583	0.6259	56992
KIF15	Parthenolide	-0.0704	0.480527	56992
KIF15	Pazopanib	0.045095	0.41606	56992
KIF15	Phenformin	-0.15373	1.76E-05	56992
KIF15	Pyrimethamine	-0.07392	0.508657	56992
KIF15	QL-VIII-58	-0.03483	0.68251	56992
KIF15	QL-X-138	-0.23901	4.09E-12	56992
KIF15	QL-XI-92	-0.23259	1.07E-11	56992
KIF15	QL-XII-47	-0.14842	6.18E-05	56992
KIF15	QL-XII-61	-0.18923	0.000642	56992
KIF15	QS11	-0.08255	0.131845	56992
KIF15	RDEA119	0.239484	1.69E-12	56992
KIF15	RO-3306	0.096488	0.017688	56992
KIF15	Rapamycin	-0.03946	0.770783	56992

KIF15	Roscovitine	0.073699	0.634357	56992
KIF15	Ruxolitinib	-0.07698	0.090993	56992
KIF15	S-Trityl-L-cysteine	-0.10511	0.159806	56992
KIF15	SB 216763	0.101404	0.029141	56992
KIF15	SB 505124	0.087018	0.151862	56992
KIF15	SB52334	-0.01055	0.863076	56992
KIF15	SB590885	0.1388	0.000868	56992
KIF15	SGC0946	0.094526	0.036534	56992
KIF15	SL 0101-1	0.058806	0.427287	56992
KIF15	SN-38	-0.09209	0.022103	56992
KIF15	SNX-2112	-0.23781	4.04E-12	56992
KIF15	STF-62247	-0.11804	0.002033	56992
KIF15	Salubrinal	-0.04211	0.647419	56992
KIF15	Saracatinib	0.044487	0.618561	56992
KIF15	Shikonin	-0.07517	0.121371	56992
KIF15	Sorafenib	-0.01434	0.918215	56992
KIF15	Sunitinib	-0.09191	0.194149	56992
KIF15	T0901317	-0.13936	0.000212	56992
KIF15	TAE684	-0.01015	0.950555	56992
KIF15	TAK-715	-0.18189	1.78E-07	56992
KIF15	TG101348	-0.25234	9.06E-14	56992
KIF15	TGX221	0.187166	0.001522	56992
KIF15	THZ-2-102-1	-0.26444	1.04E-14	56992
KIF15	THZ-2-49	-0.18348	2E-07	56992
KIF15	TL-1-85	-0.20836	1.33E-09	56992
KIF15	TL-2-105	-0.20329	6.18E-09	56992
KIF15	TPCA-1	-0.27913	7.91E-17	56992
KIF15	TW 37	-0.0561	0.213866	56992
KIF15	Talazoparib	-0.06324	0.149054	56992
KIF15	Tamoxifen	0.061386	0.547227	56992
KIF15	Temozolomide	0.006576	0.949344	56992
KIF15	Temsirolimus	-0.07313	0.108871	56992
KIF15	Thapsigargin	-0.01687	0.838782	56992
KIF15	Tipifarnib	-0.01285	0.88229	56992
KIF15	Tivozanib	-0.0849	0.078711	56992
KIF15	Trametinib	0.293503	0	56992
KIF15	Tubastatin A	-0.22912	1.86E-11	56992
KIF15	UNC0638	-0.18915	2.07E-08	56992
KIF15	UNC1215	0.136424	0.002077	56992
KIF15	VNLG/124	-0.12012	0.001614	56992
KIF15	VX-11e	0.020157	0.715151	56992
KIF15	VX-680	-0.17518	0.020261	56992
KIF15	VX-702	1.7E-05	0.999887	56992
KIF15	Veliparib	0.036961	0.600988	56992
KIF15	Vinblastine	-0.11845	0.003248	56992
KIF15	Vinorelbine	-0.06617	0.193612	56992
KIF15	Vorinostat	-0.28611	2.57E-16	56992
KIF15	WH-4-023	0.107834	0.131559	56992
KIF15	WZ-1-84	0.075215	0.329994	56992
KIF15	WZ3105	-0.27054	1.02E-15	56992

KIF15	XAV939	0.166529	5.63E-06	56992
KIF15	XL-184	-0.09605	0.021369	56992
KIF15	XMD11-85h	0.023663	0.908066	56992
KIF15	XMD13-2	-0.23287	8.5E-12	56992
KIF15	XMD14-99	-0.12215	0.000814	56992
KIF15	XMD15-27	-0.11386	0.005666	56992
KIF15	XMD8-85	-0.01776	0.864214	56992
KIF15	XMD8-92	-0.03373	0.77869	56992
KIF15	Y-39983	-0.23712	8.04E-12	56992
KIF15	YK 4-279	-0.02098	0.739955	56992
KIF15	YM155	-0.08694	0.055339	56992
KIF15	YM201636	-0.21051	1.28E-09	56992
KIF15	Z-LLNle-CHO	-0.00614	0.949708	56992
KIF15	ZG-10	-0.16406	0.003872	56992
KIF15	ZM-447439	-0.20016	4.74E-07	56992
KIF15	ZSTK474	-0.20267	4.93E-09	56992
KIF15	Zibotentan	0.02259	0.947871	56992
KIF15	piperlongumine	0.028085	0.556871	56992
KIF15	rTRAIL	0.020122	0.817482	56992
KIF15	selumetinib	0.259802	1.99E-14	56992
KIF18A	(5Z)-7-Oxozeaenol	0.073832	0.063704	81930
KIF18A	17-AAG	0.059647	0.116943	81930
KIF18A	5-Fluorouracil	0.04022	0.292975	81930
KIF18A	681640	0.001771	0.982713	81930
KIF18A	A-443654	-0.07336	0.64137	81930
KIF18A	A-770041	-0.11984	0.091647	81930
KIF18A	AC220	0.035091	0.488653	81930
KIF18A	AG-014699	-0.01354	0.784945	81930
KIF18A	AICAR	-0.03164	0.458508	81930
KIF18A	AKT inhibitor VIII	0.049625	0.275976	81930
KIF18A	AMG-706	0.014118	0.85176	81930
KIF18A	AP-24534	-0.04205	0.32803	81930
KIF18A	AR-42	-0.03755	0.318018	81930
KIF18A	AS601245	0.018025	0.781879	81930
KIF18A	AS605240	0.054813	0.199008	81930
KIF18A	AT-7519	-0.08499	0.01759	81930
KIF18A	ATRA	0.087392	0.048429	81930
KIF18A	AUY922	-0.01045	0.879818	81930
KIF18A	AZ628	0.135048	0.042394	81930
KIF18A	AZD6482	-0.02216	0.686339	81930
KIF18A	AZD7762	-0.10287	0.008164	81930
KIF18A	AZD8055	-0.06447	0.105912	81930
KIF18A	Afatinib	0.070764	0.056099	81930
KIF18A	Axitinib	-0.01116	0.871877	81930
KIF18A	BAY 61-3606	-0.01938	0.672244	81930
KIF18A	BEZ235	-0.07552	0.095947	81930
KIF18A	BHG712	0.018173	0.64752	81930
KIF18A	BI-2536	-0.14088	0.106523	81930
KIF18A	BIRB 0796	-0.01994	0.733174	81930
KIF18A	BIX02189	-0.02455	0.534181	81930

KIF18A	BMS-509744	-0.00934	0.936325	81930
KIF18A	BMS-536924	0.056157	0.360512	81930
KIF18A	BMS-708163	0.03816	0.36202	81930
KIF18A	BMS-754807	0.073474	0.145277	81930
KIF18A	BMS345541	-0.04716	0.205969	81930
KIF18A	BX-795	-0.08427	0.048395	81930
KIF18A	BX-912	-0.06809	0.059142	81930
KIF18A	Belinostat	-0.03339	0.396752	81930
KIF18A	Bexarotene	0.006796	0.95523	81930
KIF18A	Bicalutamide	-0.08089	0.055257	81930
KIF18A	Bleomycin	0.002322	0.977203	81930
KIF18A	Bleomycin (50 uM)	-0.04638	0.216112	81930
KIF18A	Bortezomib	-0.0377	0.676076	81930
KIF18A	Bosutinib	-0.05312	0.302945	81930
KIF18A	Bryostatin 1	0.041364	0.437918	81930
KIF18A	CAL-101	-0.05175	0.181175	81930
KIF18A	CAY10603	-0.05308	0.149584	81930
KIF18A	CCT007093	0.075364	0.071188	81930
KIF18A	CCT018159	-0.04244	0.416003	81930
KIF18A	CEP-701	-0.05026	0.227842	81930
KIF18A	CGP-082996	-0.0623	0.515029	81930
KIF18A	CGP-60474	-0.10053	0.189151	81930
KIF18A	CH5424802	-0.03241	0.610861	81930
KIF18A	CHIR-99021	-0.06509	0.088641	81930
KIF18A	CI-1040	0.109956	0.006251	81930
KIF18A	CMK	-0.09617	0.344038	81930
KIF18A	CP466722	-0.06616	0.069152	81930
KIF18A	CP724714	0.04495	0.404855	81930
KIF18A	CUDC-101	-0.03026	0.442038	81930
KIF18A	CX-5461	-0.00317	0.941581	81930
KIF18A	Camptothecin	-0.07419	0.079479	81930
KIF18A	Cetuximab	0.035306	0.410981	81930
KIF18A	Cisplatin	-0.04838	0.329784	81930
KIF18A	Crizotinib	-0.09444	0.402242	81930
KIF18A	Cyclopamine	-0.05763	0.59799	81930
KIF18A	Cytarabine	-0.05786	0.236937	81930
KIF18A	DMOG	-0.08	0.059293	81930
KIF18A	Dabrafenib	0.080308	0.050479	81930
KIF18A	Dasatinib	-0.06992	0.304689	81930
KIF18A	Docetaxel	-0.05674	0.138277	81930
KIF18A	Doxorubicin	-0.00844	0.920411	81930
KIF18A	EHT 1864	0.097211	0.068459	81930
KIF18A	EKB-569	0.006063	0.900804	81930
KIF18A	EX-527	0.014274	0.914823	81930
KIF18A	Elesclomol	-0.10721	0.009294	81930
KIF18A	Embelin	-0.01821	0.802521	81930
KIF18A	Epothilone B	-0.03902	0.460116	81930
KIF18A	Erlotinib	0.156545	0.018637	81930
KIF18A	Etoposide	-0.06626	0.140831	81930
KIF18A	FH535	0.042686	0.387568	81930

KIF18A	FK866	-0.09966	0.00562	81930
KIF18A	FMK	0.017084	0.834787	81930
KIF18A	FR-180204	0.069415	0.145195	81930
KIF18A	FTI-277	0.022924	0.612729	81930
KIF18A	Foretinib	-0.07328	0.066263	81930
KIF18A	GDC0449	0.023607	0.840817	81930
KIF18A	GDC0941	-0.06412	0.208264	81930
KIF18A	GNF-2	-0.05826	0.80029	81930
KIF18A	GSK-650394	-0.01922	0.831893	81930
KIF18A	GSK1070916	-0.0548	0.147909	81930
KIF18A	GSK1904529A	0.000405	0.994507	81930
KIF18A	GSK2126458	-0.01456	0.732151	81930
KIF18A	GSK269962A	-0.0498	0.324866	81930
KIF18A	GSK429286A	-0.08135	0.039825	81930
KIF18A	GSK690693	-0.06354	0.089865	81930
KIF18A	GW 441756	0.000505	0.997922	81930
KIF18A	GW-2580	0.012141	0.989269	81930
KIF18A	GW843682X	-0.14173	0.057064	81930
KIF18A	Gefitinib	0.086129	0.032858	81930
KIF18A	Gemcitabine	-0.07022	0.130625	81930
KIF18A	Genentech Cpd 10	-0.05926	0.115057	81930
KIF18A	HG-5-113-01	-0.06208	0.380899	81930
KIF18A	HG-5-88-01	0.037546	0.82236	81930
KIF18A	HG-6-64-1	-0.03751	0.45777	81930
KIF18A	I-BET-762	-0.00579	0.885508	81930
KIF18A	IOX2	-0.044	0.448405	81930
KIF18A	IPA-3	-0.04142	0.314791	81930
KIF18A	Imatinib	-0.0445	0.793507	81930
KIF18A	Ispinesib Mesylate	-0.02833	0.472255	81930
KIF18A	JNJ-26854165	-0.0011	0.989463	81930
KIF18A	JNK Inhibitor VIII	-0.0062	0.904689	81930
KIF18A	JNK-9L	0.009957	0.89418	81930
KIF18A	JQ1	0.064772	0.120027	81930
KIF18A	JQ12	-0.00093	0.991265	81930
KIF18A	JW-7-24-1	-0.05572	0.129496	81930
KIF18A	JW-7-52-1	-0.10062	0.243315	81930
KIF18A	KIN001-055	0.016479	0.843161	81930
KIF18A	KIN001-102	-0.03412	0.366516	81930
KIF18A	KIN001-135	0.099416	0.277849	81930
KIF18A	KIN001-236	0.02376	0.556174	81930
KIF18A	KIN001-244	-0.0266	0.504517	81930
KIF18A	KIN001-260	0.00057	0.988856	81930
KIF18A	KIN001-266	0.014496	0.795933	81930
KIF18A	KIN001-270	-0.08676	0.023428	81930
KIF18A	KU-55933	-0.04747	0.427291	81930
KIF18A	LAQ824	-0.04112	0.320236	81930
KIF18A	LFM-A13	0.025969	0.649117	81930
KIF18A	LY317615	-0.02265	0.643099	81930
KIF18A	Lapatinib	0.176911	0.002763	81930
KIF18A	Lenalidomide	-0.02195	0.812389	81930

KIF18A	Linifanib	0.04306	0.410767	81930
KIF18A	Lisitinib	0.080216	0.135923	81930
KIF18A	MG-132	-0.00236	0.986174	81930
KIF18A	MK-2206	-0.0754	0.118716	81930
KIF18A	MLN4924	0.010195	0.886553	81930
KIF18A	MP470	-0.00947	0.854717	81930
KIF18A	MPS-1-IN-1	0.009229	0.835286	81930
KIF18A	MS-275	-0.12823	0.075277	81930
KIF18A	Masitinib	0.000131	0.998079	81930
KIF18A	Methotrexate	-0.04798	0.212571	81930
KIF18A	Midostaurin	-0.03304	0.487509	81930
KIF18A	Mitomycin C	0.006968	0.91802	81930
KIF18A	NG-25	-0.02968	0.441834	81930
KIF18A	NPK76-II-72-1	-0.09517	0.00685	81930
KIF18A	NSC-207895	-0.10466	0.009639	81930
KIF18A	NSC-87877	0.063626	0.231667	81930
KIF18A	NU-7441	-0.03746	0.620531	81930
KIF18A	Navitoclax	-0.04952	0.200097	81930
KIF18A	Nilotinib	-0.02282	0.699255	81930
KIF18A	Nutlin-3a (-)	0.126361	0.002365	81930
KIF18A	OSI-027	-0.05234	0.15961	81930
KIF18A	OSI-930	0.007712	0.866446	81930
KIF18A	OSU-03012	-0.04823	0.330261	81930
KIF18A	Obatoclax Mesylate	-0.01027	0.862607	81930
KIF18A	Olaparib	-0.03973	0.378132	81930
KIF18A	PAC-1	-0.04661	0.281977	81930
KIF18A	PD-0325901	0.120593	0.001627	81930
KIF18A	PD-0332991	0.012074	0.866868	81930
KIF18A	PD-173074	-0.00758	0.978834	81930
KIF18A	PF-4708671	0.059799	0.50427	81930
KIF18A	PF-562271	-0.00604	0.935997	81930
KIF18A	PFI-1	0.05168	0.276958	81930
KIF18A	PHA-665752	0.015839	0.931022	81930
KIF18A	PHA-793887	-0.05447	0.1324	81930
KIF18A	PI-103	-0.05404	0.145462	81930
KIF18A	PIK-93	-0.06299	0.081455	81930
KIF18A	PLX4720	0.118013	0.001764	81930
KIF18A	Paclitaxel	-0.09313	0.325148	81930
KIF18A	Parthenolide	-0.05718	0.585982	81930
KIF18A	Pazopanib	-0.01555	0.813391	81930
KIF18A	Phenformin	0.018306	0.65747	81930
KIF18A	Pyrimethamine	-0.05185	0.661677	81930
KIF18A	QL-VIII-58	-0.12675	0.045322	81930
KIF18A	QL-X-138	-0.06572	0.076721	81930
KIF18A	QL-XI-92	-0.06851	0.061398	81930
KIF18A	QL-XII-47	-0.03946	0.344718	81930
KIF18A	QL-XII-61	-0.05523	0.421284	81930
KIF18A	QS11	-0.02371	0.741112	81930
KIF18A	RDEA119	0.150762	1.53E-05	81930
KIF18A	RO-3306	-0.03617	0.408545	81930

KIF18A	Rapamycin	-0.06777	0.555203	81930
KIF18A	Roscovitine	-0.00696	0.979146	81930
KIF18A	Ruxolitinib	-0.01543	0.809016	81930
KIF18A	S-Trityl-L-cysteine	-0.07268	0.364947	81930
KIF18A	SB 216763	0.018266	0.743023	81930
KIF18A	SB 505124	0.014404	0.885301	81930
KIF18A	SB52334	0.084091	0.046415	81930
KIF18A	SB590885	0.152314	0.000193	81930
KIF18A	SGC0946	0.032328	0.571769	81930
KIF18A	SL 0101-1	0.077101	0.29271	81930
KIF18A	SN-38	-0.06281	0.140607	81930
KIF18A	SNX-2112	-0.04338	0.25512	81930
KIF18A	STF-62247	-0.05472	0.177651	81930
KIF18A	Salubrinal	-0.0224	0.826632	81930
KIF18A	Saracatinib	-0.03436	0.708616	81930
KIF18A	Shikonin	-0.03814	0.490484	81930
KIF18A	Sorafenib	0.042212	0.728621	81930
KIF18A	Sunitinib	-0.04392	0.591305	81930
KIF18A	T0901317	-0.05878	0.150332	81930
KIF18A	TAE684	-0.03476	0.794683	81930
KIF18A	TAK-715	-0.01577	0.696032	81930
KIF18A	TG101348	-0.04612	0.216528	81930
KIF18A	TGX221	0.008108	0.91855	81930
KIF18A	THZ-2-102-1	-0.03134	0.416756	81930
KIF18A	THZ-2-49	-0.02868	0.467265	81930
KIF18A	TL-1-85	-0.03061	0.430828	81930
KIF18A	TL-2-105	-0.01909	0.643301	81930
KIF18A	TPCA-1	-0.04501	0.222844	81930
KIF18A	TW 37	-0.10463	0.00935	81930
KIF18A	Talazoparib	-0.02752	0.576369	81930
KIF18A	Tamoxifen	-0.00206	0.991495	81930
KIF18A	Temozolomide	-0.01612	0.857712	81930
KIF18A	Temsirolimus	-0.12185	0.003453	81930
KIF18A	Thapsigargin	-0.03375	0.625414	81930
KIF18A	Tipifarnib	0.009808	0.912783	81930
KIF18A	Tivozanib	-0.01901	0.783603	81930
KIF18A	Trametinib	0.128196	0.000368	81930
KIF18A	Tubastatin A	0.0104	0.79709	81930
KIF18A	UNC0638	-0.01231	0.757152	81930
KIF18A	UNC1215	0.02881	0.605304	81930
KIF18A	VNLG/124	0.041709	0.331114	81930
KIF18A	VX-11e	0.111714	0.007051	81930
KIF18A	VX-680	-0.10116	0.233072	81930
KIF18A	VX-702	0.035124	0.883998	81930
KIF18A	Veliparib	0.002477	0.978938	81930
KIF18A	Vinblastine	-0.08624	0.038933	81930
KIF18A	Vinorelbine	-0.05143	0.337539	81930
KIF18A	Vorinostat	-0.07605	0.040007	81930
KIF18A	WH-4-023	-0.03117	0.726252	81930
KIF18A	WZ-1-84	0.014435	0.878961	81930

KIF18A	WZ3105	-0.02497	0.516303	81930
KIF18A	XAV939	-0.05212	0.188897	81930
KIF18A	XL-184	-0.00523	0.93146	81930
KIF18A	XMD11-85h	0.025684	0.897816	81930
KIF18A	XMD13-2	-0.01716	0.669372	81930
KIF18A	XMD14-99	0.012765	0.772638	81930
KIF18A	XMD15-27	-0.03876	0.429112	81930
KIF18A	XMD8-85	0.012024	0.9114	81930
KIF18A	XMD8-92	-0.01936	0.890659	81930
KIF18A	Y-39983	-0.10055	0.005992	81930
KIF18A	YK 4-279	-0.02919	0.623758	81930
KIF18A	YM155	-0.09668	0.030497	81930
KIF18A	YM201636	-0.02072	0.610764	81930
KIF18A	Z-LLNle-CHO	-0.03465	0.679753	81930
KIF18A	ZG-10	-0.0802	0.208204	81930
KIF18A	ZM-447439	-0.02535	0.626671	81930
KIF18A	ZSTK474	-0.05158	0.1678	81930
KIF18A	Zibotentan	0.006155	0.990687	81930
KIF18A	piperlongumine	-0.08306	0.042242	81930
KIF18A	rTRAIL	0.049907	0.484303	81930
KIF18A	selumetinib	0.170039	9.68E-07	81930
KIF4A	(5Z)-7-Oxozeaenol	0.172217	2.2E-06	24137
KIF4A	17-AAG	0.092665	0.012627	24137
KIF4A	5-Fluorouracil	0.084066	0.021072	24137
KIF4A	681640	0.025319	0.699982	24137
KIF4A	A-443654	-0.00444	0.987966	24137
KIF4A	A-770041	-0.0115	0.915075	24137
KIF4A	AC220	0.026939	0.611912	24137
KIF4A	AG-014699	0.038909	0.38013	24137
KIF4A	AICAR	-0.00255	0.956318	24137
KIF4A	AKT inhibitor VIII	0.071652	0.100306	24137
KIF4A	AMG-706	0.011909	0.878796	24137
KIF4A	AP-24534	0.034064	0.439838	24137
KIF4A	AR-42	-0.03542	0.348152	24137
KIF4A	AS601245	0.062807	0.218897	24137
KIF4A	AS605240	0.075004	0.067449	24137
KIF4A	AT-7519	-0.02978	0.434891	24137
KIF4A	ATRA	0.01958	0.731305	24137
KIF4A	AUY922	0.079276	0.091492	24137
KIF4A	AZ628	0.207978	0.000763	24137
KIF4A	AZD6482	0.029529	0.568037	24137
KIF4A	AZD7762	-0.05244	0.204439	24137
KIF4A	AZD8055	0.028256	0.50775	24137
KIF4A	Afatinib	0.092658	0.010857	24137
KIF4A	Axitinib	-0.01932	0.764047	24137
KIF4A	BAY 61-3606	0.084687	0.033514	24137
KIF4A	BEZ235	0.02995	0.573383	24137
KIF4A	BHG712	0.056734	0.125988	24137
KIF4A	BI-2536	-0.09219	0.326957	24137
KIF4A	BIRB 0796	0.039463	0.45821	24137

KIF4A	BIX02189	0.00488	0.910306	24137
KIF4A	BMS-509744	0.01358	0.905335	24137
KIF4A	BMS-536924	0.06953	0.221145	24137
KIF4A	BMS-708163	0.053945	0.183512	24137
KIF4A	BMS-754807	0.090304	0.060914	24137
KIF4A	BMS345541	-0.04529	0.225858	24137
KIF4A	BX-795	-0.04039	0.396548	24137
KIF4A	BX-912	-0.05439	0.1364	24137
KIF4A	Belinostat	-0.02946	0.459195	24137
KIF4A	Bexarotene	0.011608	0.913082	24137
KIF4A	Bicalutamide	0.01334	0.802898	24137
KIF4A	Bleomycin	0.108101	0.019683	24137
KIF4A	Bleomycin (50 uM)	0.094971	0.008246	24137
KIF4A	Bortezomib	0.038415	0.669325	24137
KIF4A	Bosutinib	0.027118	0.652343	24137
KIF4A	Bryostatin 1	0.040477	0.448786	24137
KIF4A	CAL-101	0.015911	0.709107	24137
KIF4A	CAY10603	-0.04284	0.251553	24137
KIF4A	CCT007093	0.08273	0.045036	24137
KIF4A	CCT018159	0.040824	0.436191	24137
KIF4A	CEP-701	0.011823	0.80537	24137
KIF4A	CGP-082996	-0.03928	0.71366	24137
KIF4A	CGP-60474	0.033744	0.729542	24137
KIF4A	CH5424802	-0.02917	0.657637	24137
KIF4A	CHIR-99021	0.01291	0.76967	24137
KIF4A	CI-1040	0.181268	2.44E-06	24137
KIF4A	CMK	0.03637	0.784522	24137
KIF4A	CP466722	-0.02647	0.494546	24137
KIF4A	CP724714	0.057403	0.261286	24137
KIF4A	CUDC-101	-0.03294	0.399717	24137
KIF4A	CX-5461	-0.00965	0.819897	24137
KIF4A	Camptothecin	-0.01722	0.740662	24137
KIF4A	Cetuximab	0.075288	0.060266	24137
KIF4A	Cisplatin	-0.0177	0.762943	24137
KIF4A	Crizotinib	-0.02913	0.868158	24137
KIF4A	Cyclopamine	-0.01375	0.924033	24137
KIF4A	Cytarabine	0.024564	0.667746	24137
KIF4A	DMOG	-0.01285	0.816346	24137
KIF4A	Dabrafenib	0.13762	0.000348	24137
KIF4A	Dasatinib	0.034418	0.640957	24137
KIF4A	Docetaxel	0.027589	0.489422	24137
KIF4A	Doxorubicin	0.055609	0.353671	24137
KIF4A	EHT 1864	0.026187	0.730656	24137
KIF4A	EKB-569	0.010842	0.821416	24137
KIF4A	EX-527	-0.0239	0.84773	24137
KIF4A	Elesclomol	-0.0403	0.383919	24137
KIF4A	Embelin	0.051883	0.359956	24137
KIF4A	Epothilone B	0.03497	0.516842	24137
KIF4A	Erlotinib	0.095617	0.183014	24137
KIF4A	Etoposide	-0.02501	0.644663	24137

KIF4A	FH535	0.048576	0.314891	24137
KIF4A	FK866	-0.0483	0.199486	24137
KIF4A	FMK	0.064424	0.297962	24137
KIF4A	FR-180204	0.056802	0.252064	24137
KIF4A	FTI-277	0.100013	0.012052	24137
KIF4A	Foretinib	-0.00771	0.878123	24137
KIF4A	GDC0449	0.050941	0.577763	24137
KIF4A	GDC0941	0.003842	0.960277	24137
KIF4A	GNF-2	-0.00811	0.983292	24137
KIF4A	GSK-650394	0.02178	0.805286	24137
KIF4A	GSK1070916	-0.07347	0.047585	24137
KIF4A	GSK1904529A	-0.00634	0.911693	24137
KIF4A	GSK2126458	0.006842	0.877414	24137
KIF4A	GSK269962A	0.039292	0.462966	24137
KIF4A	GSK429286A	-0.05068	0.227782	24137
KIF4A	GSK690693	-0.0772	0.036575	24137
KIF4A	GW 441756	-0.02097	0.93525	24137
KIF4A	GW-2580	0.012176	0.989269	24137
KIF4A	GW843682X	-0.08684	0.296181	24137
KIF4A	Gefitinib	0.12347	0.001574	24137
KIF4A	Gemcitabine	-0.01201	0.841128	24137
KIF4A	Genentech Cpd 10	-0.07126	0.054697	24137
KIF4A	HG-5-113-01	-0.00764	0.935928	24137
KIF4A	HG-5-88-01	0.062919	0.636507	24137
KIF4A	HG-6-64-1	0.075713	0.087206	24137
KIF4A	I-BET-762	-0.00701	0.861251	24137
KIF4A	IOX2	-0.00425	0.958791	24137
KIF4A	IPA-3	0.012565	0.783803	24137
KIF4A	Imatinib	-0.00531	0.983232	24137
KIF4A	Ispinesib Mesylate	0.003172	0.9403	24137
KIF4A	JNJ-26854165	0.034497	0.523353	24137
KIF4A	JNK Inhibitor VIII	-0.00174	0.973883	24137
KIF4A	JNK-9L	0.0994	0.035339	24137
KIF4A	JQ1	0.074588	0.069907	24137
KIF4A	JQ12	0.071621	0.154729	24137
KIF4A	JW-7-24-1	-0.03727	0.323621	24137
KIF4A	JW-7-52-1	0.032577	0.781359	24137
KIF4A	KIN001-055	0.062871	0.259947	24137
KIF4A	KIN001-102	-0.01665	0.674962	24137
KIF4A	KIN001-135	0.041242	0.718349	24137
KIF4A	KIN001-236	0.011098	0.794708	24137
KIF4A	KIN001-244	-0.00695	0.87168	24137
KIF4A	KIN001-260	0.012662	0.759403	24137
KIF4A	KIN001-266	0.007563	0.89728	24137
KIF4A	KIN001-270	-0.07231	0.062727	24137
KIF4A	KU-55933	-0.01361	0.864523	24137
KIF4A	LAQ824	0.012072	0.791258	24137
KIF4A	LFM-A13	0.000201	0.997693	24137
KIF4A	LY317615	-0.00708	0.89669	24137
KIF4A	Lapatinib	0.099744	0.11121	24137

KIF4A	Lenalidomide	0.001571	0.988744	24137
KIF4A	Linifanib	0.058596	0.227035	24137
KIF4A	Lisitinib	0.074633	0.17486	24137
KIF4A	MG-132	0.003027	0.982185	24137
KIF4A	MK-2206	-0.04789	0.35362	24137
KIF4A	MLN4924	0.078571	0.13624	24137
KIF4A	MP470	-0.01934	0.686382	24137
KIF4A	MPS-1-IN-1	0.033997	0.396065	24137
KIF4A	MS-275	-0.06851	0.403868	24137
KIF4A	Masitinib	0.024966	0.544512	24137
KIF4A	Methotrexate	-0.00149	0.972277	24137
KIF4A	Midostaurin	0.059065	0.17863	24137
KIF4A	Mitomycin C	0.028354	0.618096	24137
KIF4A	NG-25	0.006228	0.880062	24137
KIF4A	NPK76-II-72-1	-0.0934	0.008021	24137
KIF4A	NSC-207895	-0.13375	0.00072	24137
KIF4A	NSC-87877	0.071649	0.171146	24137
KIF4A	NU-7441	0.008066	0.942656	24137
KIF4A	Navitoclax	-0.11347	0.002034	24137
KIF4A	Nilotinib	0.001152	0.985698	24137
KIF4A	Nutlin-3a (-)	0.19428	9.95E-07	24137
KIF4A	OSI-027	-0.0329	0.39158	24137
KIF4A	OSI-930	0.026593	0.52844	24137
KIF4A	OSU-03012	0.061439	0.195009	24137
KIF4A	Obatoclax Mesylate	0.073513	0.099368	24137
KIF4A	Olaparib	0.033599	0.465722	24137
KIF4A	PAC-1	0.024644	0.600353	24137
KIF4A	PD-0325901	0.181078	1.14E-06	24137
KIF4A	PD-0332991	0.098927	0.034858	24137
KIF4A	PD-173074	-0.00583	0.984441	24137
KIF4A	PF-4708671	0.045105	0.636298	24137
KIF4A	PF-562271	0.050673	0.356541	24137
KIF4A	PFI-1	0.103569	0.014992	24137
KIF4A	PHA-665752	-0.00178	0.991921	24137
KIF4A	PHA-793887	-0.01681	0.664073	24137
KIF4A	PI-103	-0.01967	0.621613	24137
KIF4A	PIK-93	-0.03441	0.357729	24137
KIF4A	PLX4720	0.118167	0.001732	24137
KIF4A	Paclitaxel	0.002194	0.991781	24137
KIF4A	Parthenolide	0.001141	0.994346	24137
KIF4A	Pazopanib	0.03263	0.581838	24137
KIF4A	Phenformin	-0.00588	0.892281	24137
KIF4A	Pyrimethamine	0.012432	0.934467	24137
KIF4A	QL-VIII-58	-0.00712	0.9471	24137
KIF4A	QL-X-138	-0.06695	0.071127	24137
KIF4A	QL-XI-92	-0.05189	0.16516	24137
KIF4A	QL-XII-47	-0.0175	0.699604	24137
KIF4A	QL-XII-61	-0.04543	0.524974	24137
KIF4A	QS11	-0.00518	0.953003	24137
KIF4A	RDEA119	0.240572	1.32E-12	24137

KIF4A	RO-3306	0.014968	0.750902	24137
KIF4A	Rapamycin	-0.02481	0.870904	24137
KIF4A	Roscovitine	0.099108	0.489944	24137
KIF4A	Ruxolitinib	-0.01374	0.832226	24137
KIF4A	S-Trityl-L-cysteine	0.033401	0.723203	24137
KIF4A	SB 216763	0.061789	0.206798	24137
KIF4A	SB 505124	0.038516	0.614649	24137
KIF4A	SB52334	0.029395	0.573834	24137
KIF4A	SB590885	0.137429	0.000996	24137
KIF4A	SGC0946	0.025695	0.670318	24137
KIF4A	SL 0101-1	0.087449	0.232512	24137
KIF4A	SN-38	0.038024	0.411576	24137
KIF4A	SNX-2112	0.002883	0.946532	24137
KIF4A	STF-62247	-0.01468	0.756273	24137
KIF4A	Salubrinal	0.019803	0.848386	24137
KIF4A	Saracatinib	0.001052	0.993533	24137
KIF4A	Shikonin	0.005871	0.931577	24137
KIF4A	Sorafenib	0.082844	0.393392	24137
KIF4A	Sunitinib	0.055689	0.480833	24137
KIF4A	T0901317	-0.02502	0.576504	24137
KIF4A	TAE684	0.002381	0.98995	24137
KIF4A	TAK-715	0.0025	0.953553	24137
KIF4A	TG101348	-0.01803	0.649804	24137
KIF4A	TGX221	0.067678	0.304633	24137
KIF4A	THZ-2-102-1	-0.0375	0.325745	24137
KIF4A	THZ-2-49	-0.01358	0.744486	24137
KIF4A	TL-1-85	0.000323	0.994217	24137
KIF4A	TL-2-105	-0.01561	0.708116	24137
KIF4A	TPCA-1	-0.01849	0.63512	24137
KIF4A	TW 37	-0.03187	0.522725	24137
KIF4A	Talazoparib	0.037496	0.426005	24137
KIF4A	Tamoxifen	0.049879	0.646381	24137
KIF4A	Temozolomide	0.034231	0.64843	24137
KIF4A	Temsirolimus	-0.01118	0.853315	24137
KIF4A	Thapsigargin	0.01442	0.867557	24137
KIF4A	Tipifarnib	0.068867	0.230942	24137
KIF4A	Tivozanib	-0.01637	0.817651	24137
KIF4A	Trametinib	0.267906	1.11E-14	24137
KIF4A	Tubastatin A	0.010148	0.801721	24137
KIF4A	UNC0638	-0.00534	0.896102	24137
KIF4A	UNC1215	0.063045	0.190087	24137
KIF4A	VNLG/124	0.04101	0.340376	24137
KIF4A	VX-11e	0.156946	8.24E-05	24137
KIF4A	VX-680	-0.03443	0.748686	24137
KIF4A	VX-702	0.04356	0.830523	24137
KIF4A	Veliparib	0.027598	0.707292	24137
KIF4A	Vinblastine	-0.00289	0.959782	24137
KIF4A	Vinorelbine	-0.00697	0.924351	24137
KIF4A	Vorinostat	-0.07393	0.046099	24137
KIF4A	WH-4-023	0.022446	0.809085	24137

KIF4A	WZ-1-84	0.035169	0.685979	24137
KIF4A	WZ3105	-0.03414	0.365042	24137
KIF4A	XAV939	0.052013	0.189742	24137
KIF4A	XL-184	0.040832	0.397403	24137
KIF4A	XMD11-85h	0.074698	0.623853	24137
KIF4A	XMD13-2	-0.01443	0.721611	24137
KIF4A	XMD14-99	0.050694	0.193909	24137
KIF4A	XMD15-27	-0.01854	0.734348	24137
KIF4A	XMD8-85	0.074556	0.354595	24137
KIF4A	XMD8-92	0.032822	0.787697	24137
KIF4A	Y-39983	-0.02875	0.479051	24137
KIF4A	YK 4-279	0.05733	0.266674	24137
KIF4A	YM155	-0.06609	0.168312	24137
KIF4A	YM201636	-0.03123	0.426585	24137
KIF4A	Z-LLNle-CHO	0.071759	0.331699	24137
KIF4A	ZG-10	-0.09196	0.139661	24137
KIF4A	ZM-447439	-0.0145	0.793748	24137
KIF4A	ZSTK474	-0.01249	0.7626	24137
KIF4A	Zibotentan	0.000213	0.999995	24137
KIF4A	piperlongumine	0.023248	0.635777	24137
KIF4A	rTRAIL	0.046761	0.519427	24137
KIF4A	selumetinib	0.238959	2.11E-12	24137
MAD2L1	(5Z)-7-Oxozeaenol	0.142492	0.000119	4085
MAD2L1	17-AAG	0.226595	1.94E-10	4085
MAD2L1	5-Fluorouracil	-0.09224	0.010956	4085
MAD2L1	681640	0.040229	0.51364	4085
MAD2L1	A-443654	0.001408	0.996809	4085
MAD2L1	A-770041	0.043088	0.628405	4085
MAD2L1	AC220	-0.03224	0.531357	4085
MAD2L1	AG-014699	0.079537	0.053623	4085
MAD2L1	AICAR	-0.1161	0.002452	4085
MAD2L1	AKT inhibitor VIII	0.116179	0.005074	4085
MAD2L1	AMG-706	0.075576	0.151897	4085
MAD2L1	AP-24534	-0.07482	0.063638	4085
MAD2L1	AR-42	-0.2014	5.4E-09	4085
MAD2L1	AS601245	0.100654	0.028418	4085
MAD2L1	AS605240	-0.02442	0.611725	4085
MAD2L1	AT-7519	-0.15412	9.52E-06	4085
MAD2L1	ATRA	-0.06769	0.145031	4085
MAD2L1	AUY922	0.054727	0.277874	4085
MAD2L1	AZ628	0.106151	0.126962	4085
MAD2L1	AZD6482	0.081848	0.049965	4085
MAD2L1	AZD7762	-0.18433	7.6E-07	4085
MAD2L1	AZD8055	-0.09522	0.014057	4085
MAD2L1	Afatinib	0.166323	1.83E-06	4085
MAD2L1	Axitinib	-0.07851	0.097132	4085
MAD2L1	BAY 61-3606	-0.05103	0.220752	4085
MAD2L1	BEZ235	0.059924	0.202194	4085
MAD2L1	BHG712	-0.12972	0.000239	4085
MAD2L1	BI-2536	-0.08703	0.359983	4085

MAD2L1	BIRB 0796	0.087488	0.064302	4085
MAD2L1	BIX02189	-0.13535	0.000133	4085
MAD2L1	BMS-509744	0.068167	0.454684	4085
MAD2L1	BMS-536924	0.00648	0.946909	4085
MAD2L1	BMS-708163	0.115305	0.002723	4085
MAD2L1	BMS-754807	0.040609	0.468498	4085
MAD2L1	BMS345541	-0.17497	4.49E-07	4085
MAD2L1	BX-795	-0.06153	0.167671	4085
MAD2L1	BX-912	-0.19078	2.6E-08	4085
MAD2L1	Belinostat	-0.15508	1.48E-05	4085
MAD2L1	Bexarotene	0.034934	0.658801	4085
MAD2L1	Bicalutamide	0.08247	0.050404	4085
MAD2L1	Bleomycin	0.078092	0.115552	4085
MAD2L1	Bleomycin (50 uM)	0.183449	1.45E-07	4085
MAD2L1	Bortezomib	0.088918	0.232389	4085
MAD2L1	Bosutinib	0.018359	0.776912	4085
MAD2L1	Bryostatin 1	0.095061	0.039577	4085
MAD2L1	CAL-101	-0.05988	0.117105	4085
MAD2L1	CAY10603	-0.18838	4.99E-08	4085
MAD2L1	CCT007093	0.121372	0.002413	4085
MAD2L1	CCT018159	0.049431	0.330721	4085
MAD2L1	CEP-701	-0.0966	0.013774	4085
MAD2L1	CGP-082996	0.041074	0.697245	4085
MAD2L1	CGP-60474	0.055334	0.534524	4085
MAD2L1	CH5424802	-0.02641	0.692129	4085
MAD2L1	CHIR-99021	0.076211	0.043136	4085
MAD2L1	CI-1040	0.125592	0.001538	4085
MAD2L1	CMK	0.005758	0.970753	4085
MAD2L1	CP466722	-0.16937	1.01E-06	4085
MAD2L1	CP724714	0.081264	0.082101	4085
MAD2L1	CUDC-101	-0.16147	5.21E-06	4085
MAD2L1	CX-5461	-0.15528	1.07E-05	4085
MAD2L1	Camptothecin	-0.09121	0.026709	4085
MAD2L1	Cetuximab	0.126233	0.000975	4085
MAD2L1	Cisplatin	0.043079	0.394636	4085
MAD2L1	Crizotinib	-0.04015	0.802061	4085
MAD2L1	Cyclopamine	0.013397	0.924843	4085
MAD2L1	Cytarabine	-0.03453	0.521228	4085
MAD2L1	DMOG	-0.05379	0.231807	4085
MAD2L1	Dabrafenib	0.097002	0.015584	4085
MAD2L1	Dasatinib	0.1272	0.044093	4085
MAD2L1	Docetaxel	0.147027	6.12E-05	4085
MAD2L1	Doxorubicin	0.021175	0.77918	4085
MAD2L1	EHT 1864	0.053088	0.392082	4085
MAD2L1	EKB-569	-0.02758	0.529699	4085
MAD2L1	EX-527	0.008579	0.952298	4085
MAD2L1	Elesclomol	0.019762	0.696677	4085
MAD2L1	Embelin	0.017169	0.815246	4085
MAD2L1	Epothilone B	0.062023	0.208686	4085
MAD2L1	Erlotinib	0.196862	0.002201	4085

MAD2L1	Etoposide	-0.08316	0.056194	4085
MAD2L1	FH535	0.09459	0.027752	4085
MAD2L1	FK866	-0.20131	6.69E-09	4085
MAD2L1	FMK	-0.00246	0.979478	4085
MAD2L1	FR-180204	-0.00085	0.991918	4085
MAD2L1	FTI-277	0.183808	1.55E-06	4085
MAD2L1	Foretinib	-0.10852	0.004318	4085
MAD2L1	GDC0449	0.033874	0.747895	4085
MAD2L1	GDC0941	-0.00448	0.95236	4085
MAD2L1	GNF-2	-0.01875	0.948211	4085
MAD2L1	GSK-650394	-0.00764	0.940921	4085
MAD2L1	GSK1070916	-0.19514	2.32E-08	4085
MAD2L1	GSK1904529A	0.107994	0.010607	4085
MAD2L1	GSK2126458	-0.08762	0.017865	4085
MAD2L1	GSK269962A	0.019976	0.745088	4085
MAD2L1	GSK429286A	-0.11817	0.001849	4085
MAD2L1	GSK690693	-0.1547	1.18E-05	4085
MAD2L1	GW 441756	0.015902	0.961176	4085
MAD2L1	GW-2580	0.017343	0.977243	4085
MAD2L1	GW843682X	-0.12777	0.092889	4085
MAD2L1	Gefitinib	0.187203	6.49E-07	4085
MAD2L1	Gemcitabine	-0.0588	0.218351	4085
MAD2L1	Genentech Cpd 10	-0.1656	2.29E-06	4085
MAD2L1	HG-5-113-01	-0.05089	0.491732	4085
MAD2L1	HG-5-88-01	0.033923	0.844594	4085
MAD2L1	HG-6-64-1	0.044956	0.357321	4085
MAD2L1	I-BET-762	-0.21548	2.02E-10	4085
MAD2L1	IOX2	0.041656	0.477119	4085
MAD2L1	IPA-3	-0.10812	0.004489	4085
MAD2L1	Imatinib	-0.05945	0.698493	4085
MAD2L1	Ispinesib Mesylate	-0.13179	0.000206	4085
MAD2L1	JNJ-26854165	0.048413	0.338433	4085
MAD2L1	JNK Inhibitor VIII	0.116352	0.00377	4085
MAD2L1	JNK-9L	0.029278	0.639445	4085
MAD2L1	JQ1	-0.01959	0.684846	4085
MAD2L1	JQ12	-0.01629	0.799946	4085
MAD2L1	JW-7-24-1	-0.15209	1.26E-05	4085
MAD2L1	JW-7-52-1	0.039346	0.724774	4085
MAD2L1	KIN001-055	0.051895	0.378837	4085
MAD2L1	KIN001-102	-0.1606	3.5E-06	4085
MAD2L1	KIN001-135	0.14888	0.078974	4085
MAD2L1	KIN001-236	-0.10987	0.002398	4085
MAD2L1	KIN001-244	-0.13166	0.000224	4085
MAD2L1	KIN001-260	-0.1427	5.18E-05	4085
MAD2L1	KIN001-266	-0.06148	0.181342	4085
MAD2L1	KIN001-270	-0.13858	0.000178	4085
MAD2L1	KU-55933	-0.04728	0.430272	4085
MAD2L1	LAQ824	-0.11707	0.001994	4085
MAD2L1	LFM-A13	0.076396	0.10622	4085
MAD2L1	LY317615	-0.06265	0.143038	4085

MAD2L1	Lapatinib	0.204067	0.000444	4085
MAD2L1	Lenalidomide	-0.0463	0.52224	4085
MAD2L1	Linifanib	-0.01081	0.870812	4085
MAD2L1	Lisitinib	0.009834	0.911937	4085
MAD2L1	MG-132	0.086419	0.292894	4085
MAD2L1	MK-2206	-0.05952	0.232873	4085
MAD2L1	MLN4924	0.083366	0.111532	4085
MAD2L1	MP470	-0.08261	0.048695	4085
MAD2L1	MPS-1-IN-1	-0.1079	0.003222	4085
MAD2L1	MS-275	-0.20469	0.002823	4085
MAD2L1	Masitinib	-0.12544	0.000527	4085
MAD2L1	Methotrexate	-0.17566	9.64E-07	4085
MAD2L1	Midostaurin	0.081347	0.054549	4085
MAD2L1	Mitomycin C	-0.03106	0.577048	4085
MAD2L1	NG-25	-0.1653	1.94E-06	4085
MAD2L1	NPK76-II-72-1	-0.27992	4.62E-17	4085
MAD2L1	NSC-207895	-0.13744	0.000495	4085
MAD2L1	NSC-87877	0.103191	0.040356	4085
MAD2L1	NU-7441	0.002418	0.982701	4085
MAD2L1	Navitoclax	-0.24085	9.56E-12	4085
MAD2L1	Nilotinib	-0.09969	0.025599	4085
MAD2L1	Nutlin-3a (-)	0.102704	0.015977	4085
MAD2L1	OSI-027	-0.1341	0.000148	4085
MAD2L1	OSI-930	-0.07965	0.036866	4085
MAD2L1	OSU-03012	-0.00431	0.9499	4085
MAD2L1	Obatoclax Mesylate	-0.00626	0.917902	4085
MAD2L1	Olaparib	0.065351	0.126633	4085
MAD2L1	PAC-1	-0.14346	0.0002	4085
MAD2L1	PD-0325901	0.213277	7.18E-09	4085
MAD2L1	PD-0332991	0.047332	0.392068	4085
MAD2L1	PD-173074	-0.02255	0.903328	4085
MAD2L1	PF-4708671	0.076719	0.363137	4085
MAD2L1	PF-562271	0.067711	0.197443	4085
MAD2L1	PFI-1	0.028081	0.595591	4085
MAD2L1	PHA-665752	0.103659	0.411373	4085
MAD2L1	PHA-793887	-0.18083	1.29E-07	4085
MAD2L1	PI-103	-0.14234	5.36E-05	4085
MAD2L1	PIK-93	-0.18795	4.05E-08	4085
MAD2L1	PLX4720	0.108863	0.004326	4085
MAD2L1	Paclitaxel	0.051276	0.660325	4085
MAD2L1	Parthenolide	-0.05891	0.572581	4085
MAD2L1	Pazopanib	0.066935	0.191483	4085
MAD2L1	Phenformin	-0.09988	0.006313	4085
MAD2L1	Pyrimethamine	-0.04225	0.731438	4085
MAD2L1	QL-VIII-58	0.086654	0.212076	4085
MAD2L1	QL-X-138	-0.1539	1.32E-05	4085
MAD2L1	QL-XI-92	-0.16525	2.21E-06	4085
MAD2L1	QL-XII-47	-0.10588	0.005337	4085
MAD2L1	QL-XII-61	-0.1425	0.013576	4085
MAD2L1	QS11	-0.06822	0.226392	4085

MAD2L1	RDEA119	0.23485	4.76E-12	4085
MAD2L1	RO-3306	0.116601	0.003796	4085
MAD2L1	Rapamycin	0.004021	0.983206	4085
MAD2L1	Roscovitine	0.068905	0.663652	4085
MAD2L1	Ruxolitinib	-0.04794	0.340388	4085
MAD2L1	S-Trityl-L-cysteine	-0.05406	0.528138	4085
MAD2L1	SB 216763	0.075927	0.112033	4085
MAD2L1	SB 505124	0.068144	0.301048	4085
MAD2L1	SB52334	-0.02275	0.677181	4085
MAD2L1	SB590885	0.130461	0.001965	4085
MAD2L1	SGC0946	0.085464	0.062646	4085
MAD2L1	SL 0101-1	0.101122	0.172468	4085
MAD2L1	SN-38	-8.9E-05	0.998946	4085
MAD2L1	SNX-2112	-0.14808	2.63E-05	4085
MAD2L1	STF-62247	-0.07603	0.054033	4085
MAD2L1	Salubrinal	-0.03566	0.705139	4085
MAD2L1	Saracatinib	0.049497	0.571783	4085
MAD2L1	Shikonin	-0.02528	0.670571	4085
MAD2L1	Sorafenib	0.007304	0.959139	4085
MAD2L1	Sunitinib	-0.03152	0.712743	4085
MAD2L1	T0901317	-0.09419	0.01571	4085
MAD2L1	TAE684	0.027736	0.840081	4085
MAD2L1	TAK-715	-0.11209	0.001675	4085
MAD2L1	TG101348	-0.17452	4.43E-07	4085
MAD2L1	TGX221	0.228154	7.63E-05	4085
MAD2L1	THZ-2-102-1	-0.2065	2.5E-09	4085
MAD2L1	THZ-2-49	-0.12017	0.000847	4085
MAD2L1	TL-1-85	-0.16817	1.33E-06	4085
MAD2L1	TL-2-105	-0.15836	8.26E-06	4085
MAD2L1	TPCA-1	-0.19434	1.32E-08	4085
MAD2L1	TW 37	-0.05364	0.238518	4085
MAD2L1	Talazoparib	0.010996	0.839223	4085
MAD2L1	Tamoxifen	0.077744	0.383244	4085
MAD2L1	Temozolomide	0.01354	0.884996	4085
MAD2L1	Temsirolimus	0.004421	0.945142	4085
MAD2L1	Thapsigargin	0.007631	0.933586	4085
MAD2L1	Tipifarnib	0.047963	0.454266	4085
MAD2L1	Tivozanib	-0.0511	0.359255	4085
MAD2L1	Trametinib	0.276121	0	4085
MAD2L1	Tubastatin A	-0.16775	1.31E-06	4085
MAD2L1	UNC0638	-0.13188	0.000126	4085
MAD2L1	UNC1215	0.147012	0.000893	4085
MAD2L1	VNLG/124	-0.03875	0.371978	4085
MAD2L1	VX-11e	0.064319	0.155248	4085
MAD2L1	VX-680	-0.09207	0.287615	4085
MAD2L1	VX-702	-0.00524	0.986254	4085
MAD2L1	Veliparib	0.056829	0.382325	4085
MAD2L1	Vinblastine	-0.06636	0.123512	4085
MAD2L1	Vinorelbine	-0.02162	0.731589	4085
MAD2L1	Vorinostat	-0.2402	1E-11	4085

MAD2L1	WH-4-023	0.099722	0.170331	4085
MAD2L1	WZ-1-84	0.169832	0.010802	4085
MAD2L1	WZ3105	-0.15225	1.17E-05	4085
MAD2L1	XAV939	0.112949	0.0027	4085
MAD2L1	XL-184	-0.03833	0.431499	4085
MAD2L1	XMD11-85h	0.014065	0.951462	4085
MAD2L1	XMD13-2	-0.15141	1.44E-05	4085
MAD2L1	XMD14-99	-0.06301	0.099485	4085
MAD2L1	XMD15-27	-0.04329	0.369072	4085
MAD2L1	XMD8-85	0.026805	0.785431	4085
MAD2L1	XMD8-92	-0.01216	0.937957	4085
MAD2L1	Y-39983	-0.15869	7.93E-06	4085
MAD2L1	YK 4-279	0.028208	0.637799	4085
MAD2L1	YM155	-0.07458	0.1115	4085
MAD2L1	YM201636	-0.15163	1.75E-05	4085
MAD2L1	Z-LLNle-CHO	0.061885	0.415844	4085
MAD2L1	ZG-10	-0.08027	0.207867	4085
MAD2L1	ZM-447439	-0.0986	0.019781	4085
MAD2L1	ZSTK474	-0.12001	0.00075	4085
MAD2L1	Zibotentan	0.024184	0.943268	4085
MAD2L1	piperlongumine	0.078597	0.05646	4085
MAD2L1	rTRAIL	0.040297	0.588777	4085
MAD2L1	selumetinib	0.242902	8.56E-13	4085
MCM10	(5Z)-7-Oxozeaenol	0.125199	0.000837	55388
MCM10	17-AAG	0.236548	2.76E-11	55388
MCM10	5-Fluorouracil	-0.1467	3.36E-05	55388
MCM10	681640	0.021061	0.755974	55388
MCM10	A-443654	-0.02478	0.914276	55388
MCM10	A-770041	0.014853	0.887936	55388
MCM10	AC220	-0.10082	0.01578	55388
MCM10	AG-014699	0.049315	0.252475	55388
MCM10	AICAR	-0.21551	5.01E-09	55388
MCM10	AKT inhibitor VIII	0.098742	0.018829	55388
MCM10	AMG-706	0.046047	0.441565	55388
MCM10	AP-24534	-0.14255	0.000172	55388
MCM10	AR-42	-0.22358	7.47E-11	55388
MCM10	AS601245	0.065382	0.196904	55388
MCM10	AS605240	-0.06799	0.101647	55388
MCM10	AT-7519	-0.20571	2.01E-09	55388
MCM10	ATRA	-0.10265	0.017573	55388
MCM10	AUY922	0.015334	0.812694	55388
MCM10	AZ628	0.076234	0.302826	55388
MCM10	AZD6482	0.042146	0.375882	55388
MCM10	AZD7762	-0.222	1.64E-09	55388
MCM10	AZD8055	-0.19415	1.72E-07	55388
MCM10	Afatinib	0.136569	0.000114	55388
MCM10	Axitinib	-0.17011	5.25E-05	55388
MCM10	BAY 61-3606	-0.10916	0.005112	55388
MCM10	BEZ235	0.028666	0.592093	55388
MCM10	BHG712	-0.20312	3.89E-09	55388

MCM10	BI-2536	-0.10895	0.231374	55388
MCM10	BIRB 0796	0.076593	0.112042	55388
MCM10	BIX02189	-0.22504	6.25E-11	55388
MCM10	BMS-509744	0.048434	0.616367	55388
MCM10	BMS-536924	-0.0366	0.609735	55388
MCM10	BMS-708163	0.125303	0.00108	55388
MCM10	BMS-754807	0.040899	0.465249	55388
MCM10	BMS345541	-0.25306	1.02E-13	55388
MCM10	BX-795	-0.13234	0.001005	55388
MCM10	BX-912	-0.26525	3.19E-15	55388
MCM10	Belinostat	-0.17887	4.74E-07	55388
MCM10	Bexarotene	0.009216	0.934816	55388
MCM10	Bicalutamide	0.074209	0.081823	55388
MCM10	Bleomycin	0.045246	0.417356	55388
MCM10	Bleomycin (50 uM)	0.168224	1.56E-06	55388
MCM10	Bortezomib	0.028977	0.759701	55388
MCM10	Bosutinib	-0.05668	0.263534	55388
MCM10	Bryostatin 1	0.11376	0.01223	55388
MCM10	CAL-101	-0.14179	7.83E-05	55388
MCM10	CAY10603	-0.2187	1.79E-10	55388
MCM10	CCT007093	0.120274	0.002638	55388
MCM10	CCT018159	-0.00035	0.995455	55388
MCM10	CEP-701	-0.14377	0.000156	55388
MCM10	CGP-082996	0.021067	0.864746	55388
MCM10	CGP-60474	0.055188	0.535658	55388
MCM10	CH5424802	-0.05389	0.327065	55388
MCM10	CHIR-99021	0.084418	0.023983	55388
MCM10	CI-1040	0.126388	0.001425	55388
MCM10	CMK	-0.01196	0.937058	55388
MCM10	CP466722	-0.27038	1.11E-15	55388
MCM10	CP724714	0.054801	0.286854	55388
MCM10	CUDC-101	-0.18383	1.74E-07	55388
MCM10	CX-5461	-0.22685	5E-11	55388
MCM10	Camptothecin	-0.11039	0.00592	55388
MCM10	Cetuximab	0.137282	0.000307	55388
MCM10	Cisplatin	-0.00133	0.983987	55388
MCM10	Crizotinib	-0.07948	0.520733	55388
MCM10	Cyclopamine	0.044019	0.706818	55388
MCM10	Cytarabine	-0.07474	0.109662	55388
MCM10	DMOG	-0.10919	0.007145	55388
MCM10	Dabrafenib	0.137789	0.000341	55388
MCM10	Dasatinib	0.072035	0.288957	55388
MCM10	Docetaxel	0.174329	1.69E-06	55388
MCM10	Doxorubicin	0.004782	0.955517	55388
MCM10	EHT 1864	-0.00653	0.944818	55388
MCM10	EKB-569	-0.12758	0.00066	55388
MCM10	EX-527	0.02422	0.845476	55388
MCM10	Elesclomol	-0.00506	0.925595	55388
MCM10	Embelin	0.046181	0.428549	55388
MCM10	Epothilone B	0.052187	0.300612	55388

MCM10	Erlotinib	0.148712	0.026894	55388
MCM10	Etoposide	-0.09881	0.020141	55388
MCM10	FH535	0.0972	0.023335	55388
MCM10	FK866	-0.24888	4.21E-13	55388
MCM10	FMK	-0.06243	0.316894	55388
MCM10	FR-180204	-0.06469	0.180868	55388
MCM10	FTI-277	0.186391	1.06E-06	55388
MCM10	Foretinib	-0.1603	1.24E-05	55388
MCM10	GDC0449	-0.0202	0.866977	55388
MCM10	GDC0941	-0.05969	0.248968	55388
MCM10	GNF-2	-0.06584	0.769374	55388
MCM10	GSK-650394	-0.01749	0.850771	55388
MCM10	GSK1070916	-0.2834	1.03E-16	55388
MCM10	GSK1904529A	0.089555	0.038002	55388
MCM10	GSK2126458	-0.15519	1.35E-05	55388
MCM10	GSK269962A	-0.01719	0.785618	55388
MCM10	GSK429286A	-0.19349	1.06E-07	55388
MCM10	GSK690693	-0.20919	1.63E-09	55388
MCM10	GW 441756	-0.03806	0.846614	55388
MCM10	GW-2580	-0.01915	0.969081	55388
MCM10	GW843682X	-0.14261	0.055411	55388
MCM10	Gefitinib	0.143515	0.000197	55388
MCM10	Gemcitabine	-0.07336	0.112278	55388
MCM10	Genentech Cpd 10	-0.23896	3.36E-12	55388
MCM10	HG-5-113-01	-0.12459	0.045907	55388
MCM10	HG-5-88-01	-0.00512	0.983427	55388
MCM10	HG-6-64-1	-0.00642	0.919017	55388
MCM10	I-BET-762	-0.27546	1.68E-16	55388
MCM10	IOX2	0.09075	0.076795	55388
MCM10	IPA-3	-0.14849	6.71E-05	55388
MCM10	Imatinib	-0.13618	0.131427	55388
MCM10	Ispinesib Mesylate	-0.16861	1.46E-06	55388
MCM10	JNJ-26854165	0.064917	0.174851	55388
MCM10	JNK Inhibitor VIII	0.087534	0.033494	55388
MCM10	JNK-9L	-0.02151	0.746643	55388
MCM10	JQ1	-0.05648	0.18273	55388
MCM10	JQ12	-0.06411	0.211985	55388
MCM10	JW-7-24-1	-0.22533	3.92E-11	55388
MCM10	JW-7-52-1	-0.01978	0.877446	55388
MCM10	KIN001-055	0.005334	0.954474	55388
MCM10	KIN001-102	-0.25406	6.03E-14	55388
MCM10	KIN001-135	0.133533	0.124167	55388
MCM10	KIN001-236	-0.18209	2.11E-07	55388
MCM10	KIN001-244	-0.20825	2.16E-09	55388
MCM10	KIN001-260	-0.19146	3.49E-08	55388
MCM10	KIN001-266	-0.09852	0.023055	55388
MCM10	KIN001-270	-0.1765	1.27E-06	55388
MCM10	KU-55933	-0.10481	0.032202	55388
MCM10	LAQ824	-0.17251	2.88E-06	55388
MCM10	LFM-A13	0.074266	0.1173	55388

MCM10	LY317615	-0.11284	0.004537	55388
MCM10	Lapatinib	0.167422	0.004908	55388
MCM10	Lenalidomide	-0.06687	0.29361	55388
MCM10	Linifanib	-0.06429	0.1779	55388
MCM10	Lisitinib	0.023111	0.757405	55388
MCM10	MG-132	0.029261	0.783809	55388
MCM10	MK-2206	-0.11879	0.008644	55388
MCM10	MLN4924	0.038871	0.511926	55388
MCM10	MP470	-0.12164	0.003422	55388
MCM10	MPS-1-IN-1	-0.16043	7.36E-06	55388
MCM10	MS-275	-0.20203	0.003238	55388
MCM10	Masitinib	-0.19257	4.56E-08	55388
MCM10	Methotrexate	-0.23365	3.77E-11	55388
MCM10	Midostaurin	0.061487	0.159783	55388
MCM10	Mitomycin C	-0.07514	0.109524	55388
MCM10	NG-25	-0.24219	1.08E-12	55388
MCM10	NPK76-II-72-1	-0.3404	3.02E-25	55388
MCM10	NSC-207895	-0.16049	4.2E-05	55388
MCM10	NSC-87877	0.115755	0.02088	55388
MCM10	NU-7441	-0.0337	0.663646	55388
MCM10	Navitoclax	-0.32385	7.2E-21	55388
MCM10	Nilotinib	-0.1586	0.000135	55388
MCM10	Nutlin-3a (-)	-0.00877	0.884933	55388
MCM10	OSI-027	-0.19828	1.07E-08	55388
MCM10	OSI-930	-0.16283	7.02E-06	55388
MCM10	OSU-03012	-0.0509	0.300536	55388
MCM10	Obatoclax Mesylate	-0.03744	0.448508	55388
MCM10	Olaparib	0.037321	0.411364	55388
MCM10	PAC-1	-0.15239	7.09E-05	55388
MCM10	PD-0325901	0.222071	1.57E-09	55388
MCM10	PD-0332991	-0.05641	0.288598	55388
MCM10	PD-173074	-0.05404	0.688988	55388
MCM10	PF-4708671	0.016876	0.901566	55388
MCM10	PF-562271	0.057354	0.287134	55388
MCM10	PFI-1	-0.01851	0.741847	55388
MCM10	PHA-665752	0.063457	0.659103	55388
MCM10	PHA-793887	-0.22954	1.16E-11	55388
MCM10	PI-103	-0.20688	2.19E-09	55388
MCM10	PIK-93	-0.27002	8.74E-16	55388
MCM10	PLX4720	0.154881	2.21E-05	55388
MCM10	Paclitaxel	0.027904	0.835741	55388
MCM10	Parthenolide	-0.03644	0.753023	55388
MCM10	Pazopanib	0.045034	0.416541	55388
MCM10	Phenformin	-0.11982	0.000938	55388
MCM10	Pyrimethamine	-0.05094	0.668352	55388
MCM10	QL-VIII-58	0.028036	0.757286	55388
MCM10	QL-X-138	-0.21529	5.3E-10	55388
MCM10	QL-XI-92	-0.23808	3.21E-12	55388
MCM10	QL-XII-47	-0.13342	0.000352	55388
MCM10	QL-XII-61	-0.12805	0.02888	55388

MCM10	QS11	-0.08811	0.102439	55388
MCM10	RDEA119	0.240693	1.29E-12	55388
MCM10	RO-3306	0.121048	0.0026	55388
MCM10	Rapamycin	-0.01103	0.94959	55388
MCM10	Roscovitine	0.115608	0.387725	55388
MCM10	Ruxolitinib	-0.09517	0.029258	55388
MCM10	S-Trityl-L-cysteine	-0.09726	0.20027	55388
MCM10	SB 216763	0.075786	0.112814	55388
MCM10	SB 505124	0.05156	0.464347	55388
MCM10	SB52334	-0.02735	0.60624	55388
MCM10	SB590885	0.171361	1.79E-05	55388
MCM10	SGC0946	0.098728	0.027998	55388
MCM10	SL 0101-1	0.061282	0.404147	55388
MCM10	SN-38	-0.01257	0.817003	55388
MCM10	SNX-2112	-0.20525	2.94E-09	55388
MCM10	STF-62247	-0.13267	0.000481	55388
MCM10	Salubrinal	-0.06917	0.4136	55388
MCM10	Saracatinib	-0.02053	0.833059	55388
MCM10	Shikonin	-0.06605	0.183355	55388
MCM10	Sorafenib	-0.01157	0.934528	55388
MCM10	Sunitinib	-0.08964	0.207948	55388
MCM10	T0901317	-0.12929	0.000638	55388
MCM10	TAE684	0.031576	0.814975	55388
MCM10	TAK-715	-0.1824	1.64E-07	55388
MCM10	TG101348	-0.25383	6.34E-14	55388
MCM10	TGX221	0.208716	0.000342	55388
MCM10	THZ-2-102-1	-0.24157	2.07E-12	55388
MCM10	THZ-2-49	-0.1336	0.000195	55388
MCM10	TL-1-85	-0.23385	7.4E-12	55388
MCM10	TL-2-105	-0.21287	1.05E-09	55388
MCM10	TPCA-1	-0.28523	1.49E-17	55388
MCM10	TW 37	-0.08621	0.038593	55388
MCM10	Talazoparib	-0.01627	0.755926	55388
MCM10	Tamoxifen	0.065675	0.50408	55388
MCM10	Temozolomide	0.017364	0.844282	55388
MCM10	Temsirolimus	-0.04674	0.350381	55388
MCM10	Thapsigargin	-0.02749	0.710005	55388
MCM10	Tipifarnib	0.005277	0.955803	55388
MCM10	Tivozanib	-0.125	0.004263	55388
MCM10	Trametinib	0.270009	0	55388
MCM10	Tubastatin A	-0.21677	2.35E-10	55388
MCM10	UNC0638	-0.16151	2.06E-06	55388
MCM10	UNC1215	0.108384	0.015074	55388
MCM10	VNLG/124	-0.10664	0.005683	55388
MCM10	VX-11e	0.007093	0.912304	55388
MCM10	VX-680	-0.12241	0.133312	55388
MCM10	VX-702	-0.0459	0.813357	55388
MCM10	Veliparib	0.01713	0.835065	55388
MCM10	Vinblastine	-0.093	0.024656	55388
MCM10	Vinorelbine	-0.05292	0.319997	55388

MCM10	Vorinostat	-0.32309	8.34E-21	55388
MCM10	WH-4-023	0.054728	0.499722	55388
MCM10	WZ-1-84	0.088637	0.237184	55388
MCM10	WZ3105	-0.2346	5.18E-12	55388
MCM10	XAV939	0.099162	0.008952	55388
MCM10	XL-184	-0.08351	0.050681	55388
MCM10	XMD11-85h	0.004256	0.986281	55388
MCM10	XMD13-2	-0.19113	2.96E-08	55388
MCM10	XMD14-99	-0.11442	0.001775	55388
MCM10	XMD15-27	-0.10479	0.012029	55388
MCM10	XMD8-85	0.00332	0.975412	55388
MCM10	XMD8-92	-0.01898	0.893296	55388
MCM10	Y-39983	-0.23332	1.81E-11	55388
MCM10	YK 4-279	0.016094	0.808854	55388
MCM10	YM155	-0.06225	0.200304	55388
MCM10	YM201636	-0.22636	5.72E-11	55388
MCM10	Z-LLNle-CHO	-0.0168	0.854475	55388
MCM10	ZG-10	-0.10241	0.093354	55388
MCM10	ZM-447439	-0.17955	7.08E-06	55388
MCM10	ZSTK474	-0.19229	3.13E-08	55388
MCM10	Zibotentan	-0.04998	0.812258	55388
MCM10	piperlongumine	0.064251	0.129298	55388
MCM10	rTRAIL	0.024208	0.772033	55388
MCM10	selumetinib	0.260097	1.99E-14	55388
MND1	(5Z)-7-Oxozeaenol	0.08886	0.022163	84057
MND1	17-AAG	0.249231	1.98E-12	84057
MND1	5-Fluorouracil	-0.19635	1.74E-08	84057
MND1	681640	0.024066	0.716202	84057
MND1	A-443654	-0.04242	0.824313	84057
MND1	A-770041	-0.06463	0.426196	84057
MND1	AC220	-0.06624	0.137873	84057
MND1	AG-014699	0.084724	0.039196	84057
MND1	AICAR	-0.19922	7.84E-08	84057
MND1	AKT inhibitor VIII	0.119633	0.00378	84057
MND1	AMG-706	0.090616	0.073497	84057
MND1	AP-24534	-0.16718	7.58E-06	84057
MND1	AR-42	-0.27324	7.99E-16	84057
MND1	AS601245	0.030376	0.606052	84057
MND1	AS605240	-0.10584	0.007307	84057
MND1	AT-7519	-0.26007	1.61E-14	84057
MND1	ATRA	-0.11429	0.007004	84057
MND1	AUY922	-0.02605	0.657743	84057
MND1	AZ628	0.067111	0.376263	84057
MND1	AZD6482	0.025151	0.638425	84057
MND1	AZD7762	-0.25962	1E-12	84057
MND1	AZD8055	-0.23199	2.61E-10	84057
MND1	Afatinib	0.203442	3.27E-09	84057
MND1	Axitinib	-0.10886	0.015433	84057
MND1	BAY 61-3606	-0.15217	6.09E-05	84057
MND1	BEZ235	-0.01033	0.864493	84057

MND1	BHG712	-0.26096	1.59E-14	84057
MND1	BI-2536	-0.14197	0.103639	84057
MND1	BIRB 0796	0.110417	0.016391	84057
MND1	BIX02189	-0.25522	7.92E-14	84057
MND1	BMS-509744	0.037023	0.712139	84057
MND1	BMS-536924	-0.00367	0.969781	84057
MND1	BMS-708163	0.149221	8.99E-05	84057
MND1	BMS-754807	0.05237	0.3291	84057
MND1	BMS345541	-0.28548	2.38E-17	84057
MND1	BX-795	-0.12577	0.001876	84057
MND1	BX-912	-0.30154	1.37E-19	84057
MND1	Belinostat	-0.24023	6.05E-12	84057
MND1	Bexarotene	-0.01081	0.91994	84057
MND1	Bicalutamide	0.108465	0.008002	84057
MND1	Bleomycin	0.011347	0.875528	84057
MND1	Bleomycin (50 uM)	0.179404	2.8E-07	84057
MND1	Bortezomib	0.075596	0.325974	84057
MND1	Bosutinib	-0.0699	0.147802	84057
MND1	Bryostatin 1	0.096092	0.037129	84057
MND1	CAL-101	-0.17822	4.67E-07	84057
MND1	CAY10603	-0.26955	1.83E-15	84057
MND1	CCT007093	0.125742	0.001639	84057
MND1	CCT018159	0.006015	0.932805	84057
MND1	CEP-701	-0.20256	4.46E-08	84057
MND1	CGP-082996	-0.07354	0.41877	84057
MND1	CGP-60474	-0.00953	0.936125	84057
MND1	CH5424802	-0.02419	0.721986	84057
MND1	CHIR-99021	0.069762	0.066338	84057
MND1	CI-1040	0.075122	0.074592	84057
MND1	CMK	-0.06519	0.566993	84057
MND1	CP466722	-0.2842	2.66E-17	84057
MND1	CP724714	0.085721	0.062612	84057
MND1	CUDC-101	-0.24778	7.35E-13	84057
MND1	CX-5461	-0.24994	3.45E-13	84057
MND1	Camptothecin	-0.15849	4.44E-05	84057
MND1	Cetuximab	0.187139	4.98E-07	84057
MND1	Cisplatin	0.027006	0.617431	84057
MND1	Crizotinib	-0.01466	0.946299	84057
MND1	Cyclopamine	-0.07942	0.424508	84057
MND1	Cytarabine	-0.14094	0.000832	84057
MND1	DMOG	-0.12457	0.001791	84057
MND1	Dabrafenib	0.057099	0.183513	84057
MND1	Dasatinib	0.056738	0.414297	84057
MND1	Docetaxel	0.173321	1.95E-06	84057
MND1	Doxorubicin	-0.04623	0.463652	84057
MND1	EHT 1864	0.0201	0.806162	84057
MND1	EKB-569	-0.14754	6.82E-05	84057
MND1	EX-527	0.049159	0.623207	84057
MND1	Elesclomol	0.022574	0.650774	84057
MND1	Embelin	-0.02247	0.749308	84057

MND1	Epothilone B	0.015554	0.798008	84057
MND1	Erlotinib	0.207672	0.001115	84057
MND1	Etoposide	-0.14395	0.000404	84057
MND1	FH535	0.057642	0.217008	84057
MND1	FK866	-0.27525	7.41E-16	84057
MND1	FMK	-0.05923	0.350076	84057
MND1	FR-180204	-0.05785	0.242417	84057
MND1	FTI-277	0.194632	3.31E-07	84057
MND1	Foretinib	-0.16968	3.42E-06	84057
MND1	GDC0449	-0.00367	0.982314	84057
MND1	GDC0941	-0.05178	0.336234	84057
MND1	GNF-2	-0.05122	0.834388	84057
MND1	GSK-650394	-0.06471	0.314911	84057
MND1	GSK1070916	-0.28152	1.72E-16	84057
MND1	GSK1904529A	0.134593	0.001119	84057
MND1	GSK2126458	-0.20934	2.11E-09	84057
MND1	GSK269962A	0.018456	0.767172	84057
MND1	GSK429286A	-0.15919	1.66E-05	84057
MND1	GSK690693	-0.23707	5.64E-12	84057
MND1	GW 441756	0.025629	0.911881	84057
MND1	GW-2580	0.041756	0.903239	84057
MND1	GW843682X	-0.20165	0.005854	84057
MND1	Gefitinib	0.190744	3.82E-07	84057
MND1	Gemcitabine	-0.13512	0.001467	84057
MND1	Genentech Cpd 10	-0.24632	6.46E-13	84057
MND1	HG-5-113-01	-0.10925	0.085991	84057
MND1	HG-5-88-01	0.027436	0.882179	84057
MND1	HG-6-64-1	-0.05647	0.227235	84057
MND1	I-BET-762	-0.3389	5.75E-25	84057
MND1	IOX2	0.038349	0.52044	84057
MND1	IPA-3	-0.20646	1.5E-08	84057
MND1	Imatinib	-0.1008	0.356447	84057
MND1	Ispinesib Mesylate	-0.24354	1.25E-12	84057
MND1	JNJ-26854165	0.024494	0.671926	84057
MND1	JNK Inhibitor VIII	0.130903	0.000987	84057
MND1	JNK-9L	-0.04807	0.387579	84057
MND1	JQ1	-0.07974	0.0506	84057
MND1	JQ12	-0.07651	0.124661	84057
MND1	JW-7-24-1	-0.26973	1.3E-15	84057
MND1	JW-7-52-1	-0.07867	0.401011	84057
MND1	KIN001-055	0.027614	0.701807	84057
MND1	KIN001-102	-0.29183	2.79E-18	84057
MND1	KIN001-135	0.147175	0.083304	84057
MND1	KIN001-236	-0.20408	4.76E-09	84057
MND1	KIN001-244	-0.24821	5.57E-13	84057
MND1	KIN001-260	-0.212	8.13E-10	84057
MND1	KIN001-266	-0.11196	0.008835	84057
MND1	KIN001-270	-0.18054	6.99E-07	84057
MND1	KU-55933	-0.10049	0.041376	84057
MND1	LAQ824	-0.20577	1.75E-08	84057

MND1	LFM-A13	0.108411	0.016441	84057
MND1	LY317615	-0.13756	0.000413	84057
MND1	Lapatinib	0.265172	2.59E-06	84057
MND1	Lenalidomide	-0.01532	0.879899	84057
MND1	Linifanib	-0.037	0.495099	84057
MND1	Lisitinib	0.031667	0.650134	84057
MND1	MG-132	0.095181	0.23283	84057
MND1	MK-2206	-0.13916	0.001684	84057
MND1	MLN4924	0.033477	0.581043	84057
MND1	MP470	-0.10497	0.011628	84057
MND1	MPS-1-IN-1	-0.16672	3.02E-06	84057
MND1	MS-275	-0.18854	0.006118	84057
MND1	Masitinib	-0.21501	7.88E-10	84057
MND1	Methotrexate	-0.32471	4.67E-21	84057
MND1	Midostaurin	0.062703	0.150978	84057
MND1	Mitomycin C	-0.10353	0.019717	84057
MND1	NG-25	-0.29218	2.92E-18	84057
MND1	NPK76-II-72-1	-0.36828	1.01E-29	84057
MND1	NSC-207895	-0.14353	0.000267	84057
MND1	NSC-87877	0.097449	0.054511	84057
MND1	NU-7441	-0.0518	0.44956	84057
MND1	Navitoclax	-0.26642	3.36E-14	84057
MND1	Nilotinib	-0.15771	0.000149	84057
MND1	Nutlin-3a (-)	-0.00342	0.958124	84057
MND1	OSI-027	-0.23978	2.76E-12	84057
MND1	OSI-930	-0.15969	1.08E-05	84057
MND1	OSU-03012	-0.11818	0.005885	84057
MND1	Obatoclax Mesylate	-0.10177	0.016265	84057
MND1	Olaparib	0.029347	0.529393	84057
MND1	PAC-1	-0.19085	3.91E-07	84057
MND1	PD-0325901	0.188917	3.61E-07	84057
MND1	PD-0332991	-0.07058	0.162347	84057
MND1	PD-173074	8.3E-05	0.999729	84057
MND1	PF-4708671	0.050565	0.579952	84057
MND1	PF-562271	0.035356	0.550583	84057
MND1	PFI-1	-0.01749	0.75716	84057
MND1	PHA-665752	0.104376	0.40662	84057
MND1	PHA-793887	-0.29921	2.16E-19	84057
MND1	PI-103	-0.25532	7.68E-14	84057
MND1	PIK-93	-0.32548	6.5E-23	84057
MND1	PLX4720	0.080985	0.041205	84057
MND1	Paclitaxel	-0.03966	0.74713	84057
MND1	Parthenolide	-0.10893	0.24144	84057
MND1	Pazopanib	0.072768	0.148243	84057
MND1	Phenformin	-0.24373	2.67E-12	84057
MND1	Pyrimethamine	-0.1385	0.161312	84057
MND1	QL-VIII-58	-0.02961	0.740852	84057
MND1	QL-X-138	-0.24976	3.68E-13	84057
MND1	QL-XI-92	-0.25061	1.88E-13	84057
MND1	QL-XII-47	-0.16598	6.31E-06	84057

MND1	QL-XII-61	-0.20526	0.000178	84057
MND1	QS11	-0.12579	0.016611	84057
MND1	RDEA119	0.211268	6.29E-10	84057
MND1	RO-3306	0.167758	2.09E-05	84057
MND1	Rapamycin	-0.09583	0.355597	84057
MND1	Roscovitine	0.044055	0.813333	84057
MND1	Ruxolitinib	-0.08257	0.066049	84057
MND1	S-Trityl-L-cysteine	-0.16763	0.016832	84057
MND1	SB 216763	0.126308	0.005483	84057
MND1	SB 505124	0.063783	0.34165	84057
MND1	SB52334	-0.00518	0.937699	84057
MND1	SB590885	0.107281	0.01463	84057
MND1	SGC0946	0.106459	0.016553	84057
MND1	SL 0101-1	0.064696	0.374169	84057
MND1	SN-38	-0.05554	0.201099	84057
MND1	SNX-2112	-0.2654	6.77E-15	84057
MND1	STF-62247	-0.12045	0.00163	84057
MND1	Salubrinal	-0.13926	0.063455	84057
MND1	Saracatinib	0.030754	0.743343	84057
MND1	Shikonin	-0.09796	0.034256	84057
MND1	Sorafenib	-0.03049	0.814148	84057
MND1	Sunitinib	-0.13696	0.037231	84057
MND1	T0901317	-0.20268	2.54E-08	84057
MND1	TAE684	0.072329	0.496674	84057
MND1	TAK-715	-0.23726	5.3E-12	84057
MND1	TG101348	-0.27363	4.38E-16	84057
MND1	TGX221	0.188108	0.001439	84057
MND1	THZ-2-102-1	-0.31238	1.83E-20	84057
MND1	THZ-2-49	-0.25047	4.71E-13	84057
MND1	TL-1-85	-0.29497	1.45E-18	84057
MND1	TL-2-105	-0.23108	2.81E-11	84057
MND1	TPCA-1	-0.32018	3.97E-22	84057
MND1	TW 37	-0.09989	0.013776	84057
MND1	Talazoparib	-0.01474	0.780429	84057
MND1	Tamoxifen	0.009931	0.951946	84057
MND1	Temozolomide	0.017388	0.843983	84057
MND1	Temsirolimus	-0.09766	0.023558	84057
MND1	Thapsigargin	-0.03917	0.556248	84057
MND1	Tipifarnib	-0.05718	0.346942	84057
MND1	Tivozanib	-0.07409	0.136731	84057
MND1	Trametinib	0.260412	4.52E-14	84057
MND1	Tubastatin A	-0.24809	2.77E-13	84057
MND1	UNC0638	-0.1988	3.47E-09	84057
MND1	UNC1215	0.190125	3.97E-05	84057
MND1	VNLG/124	-0.1257	0.000925	84057
MND1	VX-11e	-0.01854	0.74145	84057
MND1	VX-680	-0.17116	0.023161	84057
MND1	VX-702	0.014145	0.960225	84057
MND1	Veliparib	0.068848	0.278934	84057
MND1	Vinblastine	-0.14438	0.000252	84057

MND1	Vinorelbine	-0.0952	0.046227	84057
MND1	Vorinostat	-0.32976	1.08E-21	84057
MND1	WH-4-023	0.037614	0.662367	84057
MND1	WZ-1-84	0.136873	0.047751	84057
MND1	WZ3105	-0.27973	8.84E-17	84057
MND1	XAV939	0.142663	0.000117	84057
MND1	XL-184	-0.10708	0.009048	84057
MND1	XMD11-85h	-0.02581	0.896902	84057
MND1	XMD13-2	-0.2295	1.75E-11	84057
MND1	XMD14-99	-0.11329	0.001981	84057
MND1	XMD15-27	-0.06023	0.184651	84057
MND1	XMD8-85	-0.06109	0.467661	84057
MND1	XMD8-92	-0.03608	0.761967	84057
MND1	Y-39983	-0.21634	5.48E-10	84057
MND1	YK 4-279	-0.00069	0.992992	84057
MND1	YM155	-0.11298	0.009507	84057
MND1	YM201636	-0.25092	2.89E-13	84057
MND1	Z-LLNle-CHO	-0.00291	0.975703	84057
MND1	ZG-10	-0.12843	0.0284	84057
MND1	ZM-447439	-0.19643	7.84E-07	84057
MND1	ZSTK474	-0.2471	5.25E-13	84057
MND1	Zibotentan	0.049352	0.813359	84057
MND1	piperlongumine	0.058323	0.17321	84057
MND1	rTRAIL	0.020391	0.81441	84057
MND1	selumetinib	0.214714	3.5E-10	84057
NCAPG	(5Z)-7-Oxozeaenol	0.114837	0.002386	64151
NCAPG	17-AAG	0.277892	0	64151
NCAPG	5-Fluorouracil	-0.07751	0.034295	64151
NCAPG	681640	0.009878	0.896733	64151
NCAPG	A-443654	-0.00473	0.986826	64151
NCAPG	A-770041	0.054435	0.52134	64151
NCAPG	AC220	-0.05608	0.221041	64151
NCAPG	AG-014699	0.013656	0.783302	64151
NCAPG	AICAR	-0.12146	0.001484	64151
NCAPG	AKT inhibitor VIII	0.125967	0.002187	64151
NCAPG	AMG-706	0.02697	0.696992	64151
NCAPG	AP-24534	-0.0712	0.079221	64151
NCAPG	AR-42	-0.19673	1.26E-08	64151
NCAPG	AS601245	0.105968	0.020041	64151
NCAPG	AS605240	-0.00231	0.966689	64151
NCAPG	AT-7519	-0.18369	1.02E-07	64151
NCAPG	ATRA	-0.09209	0.036104	64151
NCAPG	AUY922	0.059143	0.233813	64151
NCAPG	AZ628	0.164019	0.010542	64151
NCAPG	AZD6482	0.025384	0.634647	64151
NCAPG	AZD7762	-0.15359	4.7E-05	64151
NCAPG	AZD8055	-0.14637	0.000106	64151
NCAPG	Afatinib	0.212738	5.33E-10	64151
NCAPG	Axitinib	-0.07795	0.100442	64151
NCAPG	BAY 61-3606	-0.06567	0.107244	64151

NCAPG	BEZ235	0.002615	0.966788	64151
NCAPG	BHG712	-0.12261	0.000538	64151
NCAPG	BI-2536	-0.08406	0.381034	64151
NCAPG	BIRB 0796	0.051197	0.317381	64151
NCAPG	BIX02189	-0.16118	4.36E-06	64151
NCAPG	BMS-509744	0.029963	0.773349	64151
NCAPG	BMS-536924	-0.00553	0.954909	64151
NCAPG	BMS-708163	0.114583	0.002904	64151
NCAPG	BMS-754807	0.021591	0.728906	64151
NCAPG	BMS345541	-0.16577	1.86E-06	64151
NCAPG	BX-795	-0.11689	0.004102	64151
NCAPG	BX-912	-0.2422	8.11E-13	64151
NCAPG	Belinostat	-0.17173	1.4E-06	64151
NCAPG	Bexarotene	0.032874	0.679727	64151
NCAPG	Bicalutamide	0.035044	0.453618	64151
NCAPG	Bleomycin	0.103602	0.026543	64151
NCAPG	Bleomycin (50 uM)	0.152724	1.43E-05	64151
NCAPG	Bortezomib	0.105547	0.139111	64151
NCAPG	Bosutinib	0.002285	0.975634	64151
NCAPG	Bryostatin 1	0.084289	0.073445	64151
NCAPG	CAL-101	-0.11606	0.001421	64151
NCAPG	CAY10603	-0.20356	3.37E-09	64151
NCAPG	CCT007093	0.12173	0.002347	64151
NCAPG	CCT018159	0.054112	0.277079	64151
NCAPG	CEP-701	-0.12115	0.001665	64151
NCAPG	CGP-082996	0.018583	0.881631	64151
NCAPG	CGP-60474	0.038149	0.691171	64151
NCAPG	CH5424802	-0.07144	0.162282	64151
NCAPG	CHIR-99021	0.048027	0.220984	64151
NCAPG	CI-1040	0.15828	4.54E-05	64151
NCAPG	CMK	0.010025	0.949537	64151
NCAPG	CP466722	-0.20146	4.35E-09	64151
NCAPG	CP724714	0.123429	0.003203	64151
NCAPG	CUDC-101	-0.17692	5.21E-07	64151
NCAPG	CX-5461	-0.17759	3.94E-07	64151
NCAPG	Camptothecin	-0.11892	0.002819	64151
NCAPG	Cetuximab	0.152078	5.49E-05	64151
NCAPG	Cisplatin	-0.02533	0.644417	64151
NCAPG	Crizotinib	-0.05405	0.718938	64151
NCAPG	Cyclopamine	-0.0105	0.942041	64151
NCAPG	Cytarabine	-0.03131	0.567556	64151
NCAPG	DMOG	-0.08707	0.037546	64151
NCAPG	Dabrafenib	0.07688	0.062734	64151
NCAPG	Dasatinib	0.110073	0.086682	64151
NCAPG	Docetaxel	0.171007	2.7E-06	64151
NCAPG	Doxorubicin	0.002237	0.97957	64151
NCAPG	EHT 1864	0.035616	0.610665	64151
NCAPG	EKB-569	-0.01916	0.677307	64151
NCAPG	EX-527	-0.01635	0.900101	64151
NCAPG	Elesclomol	-0.01451	0.780341	64151

NCAPG	Embelin	0.047028	0.417482	64151
NCAPG	Epothilone B	0.080857	0.087306	64151
NCAPG	Erlotinib	0.237664	0.000151	64151
NCAPG	Etoposide	-0.09997	0.018585	64151
NCAPG	FH535	0.149444	0.000247	64151
NCAPG	FK866	-0.22442	7.92E-11	64151
NCAPG	FMK	-0.0046	0.95988	64151
NCAPG	FR-180204	-0.0204	0.738966	64151
NCAPG	FTI-277	0.165301	1.83E-05	64151
NCAPG	Foretinib	-0.1466	7.15E-05	64151
NCAPG	GDC0449	-0.02291	0.846826	64151
NCAPG	GDC0941	-0.05119	0.343576	64151
NCAPG	GNF-2	-0.03172	0.909896	64151
NCAPG	GSK-650394	0.017838	0.845765	64151
NCAPG	GSK1070916	-0.23172	2.07E-11	64151
NCAPG	GSK1904529A	0.068	0.125268	64151
NCAPG	GSK2126458	-0.12128	0.000812	64151
NCAPG	GSK269962A	0.00191	0.978763	64151
NCAPG	GSK429286A	-0.13977	0.000179	64151
NCAPG	GSK690693	-0.19135	3.99E-08	64151
NCAPG	GW 441756	0.001231	0.995843	64151
NCAPG	GW-2580	-0.00246	0.99615	64151
NCAPG	GW843682X	-0.10343	0.193691	64151
NCAPG	Gefitinib	0.191488	3.42E-07	64151
NCAPG	Gemcitabine	-0.05587	0.247126	64151
NCAPG	Genentech Cpd 10	-0.19379	2.4E-08	64151
NCAPG	HG-5-113-01	-0.08034	0.231777	64151
NCAPG	HG-5-88-01	-0.0524	0.72217	64151
NCAPG	HG-6-64-1	0.020485	0.713265	64151
NCAPG	I-BET-762	-0.21802	1.2E-10	64151
NCAPG	IOX2	0.021203	0.753618	64151
NCAPG	IPA-3	-0.11677	0.002035	64151
NCAPG	Imatinib	-0.04284	0.804496	64151
NCAPG	Ispinesib Mesylate	-0.13481	0.000143	64151
NCAPG	JNJ-26854165	0.032414	0.556394	64151
NCAPG	JNK Inhibitor VIII	0.071251	0.089176	64151
NCAPG	JNK-9L	0.055016	0.305367	64151
NCAPG	JQ1	-0.01118	0.830502	64151
NCAPG	JQ12	-0.01073	0.876452	64151
NCAPG	JW-7-24-1	-0.22101	9.52E-11	64151
NCAPG	JW-7-52-1	0.020548	0.871893	64151
NCAPG	KIN001-055	0.059559	0.294244	64151
NCAPG	KIN001-102	-0.16924	9.55E-07	64151
NCAPG	KIN001-135	0.105081	0.24348	64151
NCAPG	KIN001-236	-0.1306	0.000264	64151
NCAPG	KIN001-244	-0.16655	2.27E-06	64151
NCAPG	KIN001-260	-0.15491	1.02E-05	64151
NCAPG	KIN001-266	-0.07404	0.099969	64151
NCAPG	KIN001-270	-0.16178	9.98E-06	64151
NCAPG	KU-55933	-0.07753	0.137716	64151

NCAPG	LAQ824	-0.09596	0.012605	64151
NCAPG	LFM-A13	0.057673	0.241036	64151
NCAPG	LY317615	-0.03003	0.522713	64151
NCAPG	Lapatinib	0.269999	1.61E-06	64151
NCAPG	Lenalidomide	-0.04188	0.577556	64151
NCAPG	Linifanib	-0.00762	0.912847	64151
NCAPG	Lisitinib	0.000888	0.992001	64151
NCAPG	MG-132	0.092492	0.248991	64151
NCAPG	MK-2206	-0.09099	0.052722	64151
NCAPG	MLN4924	0.10696	0.035388	64151
NCAPG	MP470	-0.09061	0.02987	64151
NCAPG	MPS-1-IN-1	-0.14684	4.44E-05	64151
NCAPG	MS-275	-0.13973	0.049046	64151
NCAPG	Masitinib	-0.14431	5.73E-05	64151
NCAPG	Methotrexate	-0.22558	1.85E-10	64151
NCAPG	Midostaurin	0.0599	0.172099	64151
NCAPG	Mitomycin C	-0.01275	0.838158	64151
NCAPG	NG-25	-0.17032	9.09E-07	64151
NCAPG	NPK76-II-72-1	-0.29014	2.62E-18	64151
NCAPG	NSC-207895	-0.18924	1.34E-06	64151
NCAPG	NSC-87877	0.086123	0.093588	64151
NCAPG	NU-7441	-0.03192	0.685882	64151
NCAPG	Navitoclax	-0.29375	3.7E-17	64151
NCAPG	Nilotinib	-0.08421	0.067005	64151
NCAPG	Nutlin-3a (-)	0.039418	0.43056	64151
NCAPG	OSI-027	-0.19777	1.17E-08	64151
NCAPG	OSI-930	-0.05546	0.158653	64151
NCAPG	OSU-03012	-0.00355	0.959665	64151
NCAPG	Obatoclax Mesylate	0.009531	0.87289	64151
NCAPG	Olaparib	-0.02073	0.671094	64151
NCAPG	PAC-1	-0.11796	0.002651	64151
NCAPG	PD-0325901	0.235602	1.31E-10	64151
NCAPG	PD-0332991	0.020315	0.75839	64151
NCAPG	PD-173074	-0.01767	0.925057	64151
NCAPG	PF-4708671	0.065946	0.45116	64151
NCAPG	PF-562271	0.042474	0.452547	64151
NCAPG	PFI-1	0.009224	0.880373	64151
NCAPG	PHA-665752	0.053762	0.715304	64151
NCAPG	PHA-793887	-0.19433	1.23E-08	64151
NCAPG	PI-103	-0.20804	1.76E-09	64151
NCAPG	PIK-93	-0.23204	7.28E-12	64151
NCAPG	PLX4720	0.08112	0.040843	64151
NCAPG	Paclitaxel	0.033284	0.795607	64151
NCAPG	Parthenolide	-0.01136	0.932783	64151
NCAPG	Pazopanib	0.019281	0.762661	64151
NCAPG	Phenformin	-0.09975	0.006383	64151
NCAPG	Pyrimethamine	-0.03494	0.785033	64151
NCAPG	QL-VIII-58	0.017728	0.85705	64151
NCAPG	QL-X-138	-0.22755	4.55E-11	64151
NCAPG	QL-XI-92	-0.20325	3.86E-09	64151

NCAPG	QL-XII-47	-0.11751	0.001831	64151
NCAPG	QL-XII-61	-0.15019	0.008737	64151
NCAPG	QS11	-0.05475	0.358568	64151
NCAPG	RDEA119	0.25661	2.94E-14	64151
NCAPG	RO-3306	0.117994	0.003368	64151
NCAPG	Rapamycin	-0.00232	0.990215	64151
NCAPG	Roscovitine	0.116407	0.381948	64151
NCAPG	Ruxolitinib	-0.057	0.240866	64151
NCAPG	S-Trityl-L-cysteine	-0.02366	0.810121	64151
NCAPG	SB 216763	0.08636	0.067624	64151
NCAPG	SB 505124	0.031549	0.696286	64151
NCAPG	SB52334	-0.08726	0.037244	64151
NCAPG	SB590885	0.136316	0.001117	64151
NCAPG	SGC0946	0.061891	0.210961	64151
NCAPG	SL 0101-1	0.051295	0.491186	64151
NCAPG	SN-38	-0.03265	0.49121	64151
NCAPG	SNX-2112	-0.14248	5.44E-05	64151
NCAPG	STF-62247	-0.08929	0.022179	64151
NCAPG	Salubrinal	-0.00673	0.95414	64151
NCAPG	Saracatinib	0.065103	0.435668	64151
NCAPG	Shikonin	-0.06074	0.228648	64151
NCAPG	Sorafenib	0.059119	0.591235	64151
NCAPG	Sunitinib	-0.01474	0.879258	64151
NCAPG	T0901317	-0.12779	0.000749	64151
NCAPG	TAE684	0.031384	0.816499	64151
NCAPG	TAK-715	-0.11154	0.00177	64151
NCAPG	TG101348	-0.20794	1.24E-09	64151
NCAPG	TGX221	0.151138	0.012527	64151
NCAPG	THZ-2-102-1	-0.20826	1.79E-09	64151
NCAPG	THZ-2-49	-0.1017	0.005137	64151
NCAPG	TL-1-85	-0.16581	1.91E-06	64151
NCAPG	TL-2-105	-0.16445	3.49E-06	64151
NCAPG	TPCA-1	-0.21782	1.47E-10	64151
NCAPG	TW 37	-0.05838	0.192459	64151
NCAPG	Talazoparib	-0.0174	0.738054	64151
NCAPG	Tamoxifen	0.00378	0.983058	64151
NCAPG	Temozolomide	-0.01189	0.901057	64151
NCAPG	Temsirolimus	-0.05861	0.219652	64151
NCAPG	Thapsigargin	0.026261	0.724965	64151
NCAPG	Tipifarnib	0.048206	0.450715	64151
NCAPG	Tivozanib	-0.07798	0.112254	64151
NCAPG	Trametinib	0.295417	0	64151
NCAPG	Tubastatin A	-0.20353	3.02E-09	64151
NCAPG	UNC0638	-0.19843	3.73E-09	64151
NCAPG	UNC1215	0.10072	0.024489	64151
NCAPG	VNLG/124	-0.07491	0.060944	64151
NCAPG	VX-11e	0.081021	0.063303	64151
NCAPG	VX-680	-0.09594	0.264193	64151
NCAPG	VX-702	-0.05155	0.774175	64151
NCAPG	Veliparib	0.033157	0.643646	64151

NCAPG	Vinblastine	-0.06511	0.13168	64151
NCAPG	Vinorelbine	-0.0328	0.577849	64151
NCAPG	Vorinostat	-0.24892	1.59E-12	64151
NCAPG	WH-4-023	0.093009	0.208028	64151
NCAPG	WZ-1-84	0.119295	0.0931	64151
NCAPG	WZ3105	-0.17532	3.74E-07	64151
NCAPG	XAV939	0.088042	0.02143	64151
NCAPG	XL-184	-0.06014	0.183141	64151
NCAPG	XMD11-85h	-0.01445	0.949867	64151
NCAPG	XMD13-2	-0.19452	1.63E-08	64151
NCAPG	XMD14-99	-0.1073	0.003519	64151
NCAPG	XMD15-27	-0.08816	0.039562	64151
NCAPG	XMD8-85	0.05845	0.491301	64151
NCAPG	XMD8-92	-0.0461	0.679106	64151
NCAPG	Y-39983	-0.18535	1.4E-07	64151
NCAPG	YK 4-279	0.062736	0.216777	64151
NCAPG	YM155	-0.09958	0.02506	64151
NCAPG	YM201636	-0.19172	3.78E-08	64151
NCAPG	Z-LLNle-CHO	0.071614	0.33278	64151
NCAPG	ZG-10	-0.14264	0.013384	64151
NCAPG	ZM-447439	-0.11625	0.005038	64151
NCAPG	ZSTK474	-0.16523	2.39E-06	64151
NCAPG	Zibotentan	-0.02157	0.949888	64151
NCAPG	piperlongumine	0.032377	0.488452	64151
NCAPG	rTRAIL	0.078062	0.212735	64151
NCAPG	selumetinib	0.244376	6.13E-13	64151
NDC80	(5Z)-7-Oxozeaenol	0.178608	8.41E-07	10403
NDC80	17-AAG	0.14247	9.03E-05	10403
NDC80	5-Fluorouracil	-0.01422	0.724751	10403
NDC80	681640	-0.00708	0.924328	10403
NDC80	A-443654	-0.06056	0.714829	10403
NDC80	A-770041	-0.05884	0.479206	10403
NDC80	AC220	-0.01319	0.827179	10403
NDC80	AG-014699	0.037002	0.407424	10403
NDC80	AICAR	-0.10045	0.009448	10403
NDC80	AKT inhibitor VIII	0.067801	0.122535	10403
NDC80	AMG-706	0.036227	0.56998	10403
NDC80	AP-24534	-0.07548	0.061015	10403
NDC80	AR-42	-0.10002	0.005052	10403
NDC80	AS601245	0.063141	0.215874	10403
NDC80	AS605240	0.02366	0.623914	10403
NDC80	AT-7519	-0.17926	2.13E-07	10403
NDC80	ATRA	-0.02764	0.609999	10403
NDC80	AUY922	-0.01064	0.876927	10403
NDC80	AZ628	0.185924	0.003103	10403
NDC80	AZD6482	0.035726	0.468362	10403
NDC80	AZD7762	-0.15837	2.59E-05	10403
NDC80	AZD8055	-0.08699	0.025715	10403
NDC80	Afatinib	0.091677	0.011784	10403
NDC80	Axitinib	-0.05635	0.265845	10403

NDC80	BAY 61-3606	-0.02153	0.635307	10403
NDC80	BEZ235	-0.0224	0.685577	10403
NDC80	BHG712	-0.05089	0.173251	10403
NDC80	BI-2536	-0.13662	0.118532	10403
NDC80	BIRB 0796	0.012207	0.841308	10403
NDC80	BIX02189	-0.10086	0.005142	10403
NDC80	BMS-509744	0.058789	0.53177	10403
NDC80	BMS-536924	0.037394	0.597262	10403
NDC80	BMS-708163	0.081389	0.038155	10403
NDC80	BMS-754807	0.078458	0.114814	10403
NDC80	BMS345541	-0.14105	5.67E-05	10403
NDC80	BX-795	-0.09524	0.022985	10403
NDC80	BX-912	-0.15753	5.29E-06	10403
NDC80	Belinostat	-0.09869	0.007172	10403
NDC80	Bexarotene	0.025815	0.761365	10403
NDC80	Bicalutamide	0.01793	0.725576	10403
NDC80	Bleomycin	0.034261	0.563543	10403
NDC80	Bleomycin (50 uM)	0.044042	0.241472	10403
NDC80	Bortezomib	0.036325	0.688813	10403
NDC80	Bosutinib	-0.06198	0.211034	10403
NDC80	Bryostatin 1	0.083881	0.075017	10403
NDC80	CAL-101	-0.08131	0.029156	10403
NDC80	CAY10603	-0.11253	0.001494	10403
NDC80	CCT007093	0.079016	0.057122	10403
NDC80	CCT018159	-0.02234	0.701188	10403
NDC80	CEP-701	-0.05465	0.186611	10403
NDC80	CGP-082996	-0.06428	0.497831	10403
NDC80	CGP-60474	-0.03541	0.715098	10403
NDC80	CH5424802	-0.00987	0.902251	10403
NDC80	CHIR-99021	0.046356	0.239293	10403
NDC80	CI-1040	0.156547	5.56E-05	10403
NDC80	CMK	-0.00814	0.95804	10403
NDC80	CP466722	-0.12889	0.000243	10403
NDC80	CP724714	0.083979	0.069813	10403
NDC80	CUDC-101	-0.12138	0.000743	10403
NDC80	CX-5461	-0.0832	0.022928	10403
NDC80	Camptothecin	-0.09711	0.017276	10403
NDC80	Cetuximab	0.065179	0.108939	10403
NDC80	Cisplatin	-0.039	0.446901	10403
NDC80	Crizotinib	-0.01494	0.945293	10403
NDC80	Cyclopamine	-0.045	0.699296	10403
NDC80	Cytarabine	-0.04974	0.324047	10403
NDC80	DMOG	-0.09905	0.015855	10403
NDC80	Dabrafenib	0.168049	8.01E-06	10403
NDC80	Dasatinib	0.013938	0.859871	10403
NDC80	Docetaxel	0.001308	0.976037	10403
NDC80	Doxorubicin	-0.02778	0.695811	10403
NDC80	EHT 1864	0.084262	0.124053	10403
NDC80	EKB-569	-0.0552	0.172565	10403
NDC80	EX-527	0.003877	0.979279	10403

NDC80	Elesclomol	-0.06977	0.107044	10403
NDC80	Embelin	0.01302	0.867845	10403
NDC80	Epothilone B	-0.00986	0.877623	10403
NDC80	Erlotinib	0.147836	0.028025	10403
NDC80	Etoposide	-0.11445	0.006028	10403
NDC80	FH535	0.093015	0.030839	10403
NDC80	FK866	-0.16852	1.5E-06	10403
NDC80	FMK	0.032715	0.653417	10403
NDC80	FR-180204	0.013099	0.842984	10403
NDC80	FTI-277	0.145087	0.000188	10403
NDC80	Foretinib	-0.08435	0.03128	10403
NDC80	GDC0449	0.00525	0.972896	10403
NDC80	GDC0941	-0.03994	0.488228	10403
NDC80	GNF-2	-0.05179	0.831145	10403
NDC80	GSK-650394	-0.01757	0.849882	10403
NDC80	GSK1070916	-0.14807	3.04E-05	10403
NDC80	GSK1904529A	0.059778	0.183675	10403
NDC80	GSK2126458	-0.07302	0.05147	10403
NDC80	GSK269962A	0.028886	0.615357	10403
NDC80	GSK429286A	-0.08754	0.02579	10403
NDC80	GSK690693	-0.14361	5.13E-05	10403
NDC80	GW 441756	-0.01548	0.961858	10403
NDC80	GW-2580	0.033075	0.938104	10403
NDC80	GW843682X	-0.14579	0.048269	10403
NDC80	Gefitinib	0.07924	0.051228	10403
NDC80	Gemcitabine	-0.08785	0.049928	10403
NDC80	Genentech Cpd 10	-0.1323	0.000197	10403
NDC80	HG-5-113-01	-0.02657	0.751528	10403
NDC80	HG-5-88-01	0.046364	0.764129	10403
NDC80	HG-6-64-1	-0.01491	0.799816	10403
NDC80	I-BET-762	-0.13663	7.58E-05	10403
NDC80	IOX2	-0.01626	0.818766	10403
NDC80	IPA-3	-0.09361	0.014932	10403
NDC80	Imatinib	-0.07692	0.556712	10403
NDC80	Ispinesib Mesylate	-0.10205	0.004674	10403
NDC80	JNJ-26854165	0.014517	0.817605	10403
NDC80	JNK Inhibitor VIII	0.011382	0.818099	10403
NDC80	JNK-9L	-0.00566	0.944609	10403
NDC80	JQ1	0.013684	0.789089	10403
NDC80	JQ12	0.025219	0.677259	10403
NDC80	JW-7-24-1	-0.12205	0.000525	10403
NDC80	JW-7-52-1	-0.03999	0.720051	10403
NDC80	KIN001-055	0.009813	0.914551	10403
NDC80	KIN001-102	-0.12542	0.000337	10403
NDC80	KIN001-135	0.098385	0.285196	10403
NDC80	KIN001-236	-0.06407	0.087477	10403
NDC80	KIN001-244	-0.08655	0.017901	10403
NDC80	KIN001-260	-0.06975	0.056695	10403
NDC80	KIN001-266	-0.01505	0.78739	10403
NDC80	KIN001-270	-0.102	0.006878	10403

NDC80	KU-55933	-0.0731	0.167749	10403
NDC80	LAQ824	-0.06953	0.078758	10403
NDC80	LFM-A13	0.073343	0.122394	10403
NDC80	LY317615	-0.04505	0.312809	10403
NDC80	Lapatinib	0.173106	0.003492	10403
NDC80	Lenalidomide	-0.03793	0.62865	10403
NDC80	Linifanib	0.015002	0.81562	10403
NDC80	Lisitinib	0.068905	0.221732	10403
NDC80	MG-132	0.053012	0.569659	10403
NDC80	MK-2206	-0.0922	0.049314	10403
NDC80	MLN4924	0.030614	0.618631	10403
NDC80	MP470	-0.00386	0.943191	10403
NDC80	MPS-1-IN-1	-0.01778	0.67408	10403
NDC80	MS-275	-0.13875	0.050882	10403
NDC80	Masitinib	-0.07346	0.050209	10403
NDC80	Methotrexate	-0.13678	0.000164	10403
NDC80	Midostaurin	0.023371	0.638865	10403
NDC80	Mitomycin C	-0.03449	0.528174	10403
NDC80	NG-25	-0.10837	0.002271	10403
NDC80	NPK76-II-72-1	-0.19569	8.81E-09	10403
NDC80	NSC-207895	-0.104	0.010148	10403
NDC80	NSC-87877	0.089899	0.078334	10403
NDC80	NU-7441	-0.0254	0.7629	10403
NDC80	Navitoclax	-0.17033	2.25E-06	10403
NDC80	Nilotinib	-0.07828	0.093001	10403
NDC80	Nutlin-3a (-)	0.148209	0.000278	10403
NDC80	OSI-027	-0.09224	0.010347	10403
NDC80	OSI-930	-0.03141	0.449564	10403
NDC80	OSU-03012	-0.03997	0.435386	10403
NDC80	Obatoclax Mesylate	-0.00824	0.891436	10403
NDC80	Olaparib	-0.00131	0.980895	10403
NDC80	PAC-1	-0.0651	0.117995	10403
NDC80	PD-0325901	0.204284	3.26E-08	10403
NDC80	PD-0332991	0.05518	0.302271	10403
NDC80	PD-173074	-0.02846	0.867408	10403
NDC80	PF-4708671	0.080412	0.342017	10403
NDC80	PF-562271	0.043515	0.439687	10403
NDC80	PFI-1	0.014249	0.80523	10403
NDC80	PHA-665752	0.073925	0.593248	10403
NDC80	PHA-793887	-0.14557	2.54E-05	10403
NDC80	PI-103	-0.11513	0.001216	10403
NDC80	PIK-93	-0.15526	7.02E-06	10403
NDC80	PLX4720	0.164072	5.95E-06	10403
NDC80	Paclitaxel	-0.06666	0.533574	10403
NDC80	Parthenolide	-0.00849	0.951915	10403
NDC80	Pazopanib	0.043497	0.435412	10403
NDC80	Phenformin	-0.07311	0.049609	10403
NDC80	Pyrimethamine	-0.05388	0.647799	10403
NDC80	QL-VIII-58	-0.03795	0.652042	10403
NDC80	QL-X-138	-0.13295	0.000191	10403

NDC80	QL-XI-92	-0.13454	0.000137	10403
NDC80	QL-XII-47	-0.08172	0.035258	10403
NDC80	QL-XII-61	-0.08508	0.175865	10403
NDC80	QS11	-0.04568	0.463909	10403
NDC80	RDEA119	0.249252	1.77E-13	10403
NDC80	RO-3306	0.026944	0.550287	10403
NDC80	Rapamycin	-0.10052	0.322688	10403
NDC80	Roscovitine	0.069301	0.66276	10403
NDC80	Ruxolitinib	-0.03683	0.489635	10403
NDC80	S-Trityl-L-cysteine	-0.06359	0.441914	10403
NDC80	SB 216763	0.099568	0.03246	10403
NDC80	SB 505124	0.051638	0.464265	10403
NDC80	SB52334	0.036802	0.461813	10403
NDC80	SB590885	0.183063	3.48E-06	10403
NDC80	SGC0946	0.065813	0.176032	10403
NDC80	SL 0101-1	0.06816	0.350389	10403
NDC80	SN-38	-0.02042	0.686919	10403
NDC80	SNX-2112	-0.11909	0.000834	10403
NDC80	STF-62247	-0.07166	0.070936	10403
NDC80	Salubrinal	-0.02083	0.83934	10403
NDC80	Saracatinib	-0.01624	0.869953	10403
NDC80	Shikonin	-0.02605	0.659856	10403
NDC80	Sorafenib	0.032509	0.799031	10403
NDC80	Sunitinib	-0.04468	0.583799	10403
NDC80	T0901317	-0.07508	0.05973	10403
NDC80	TAE684	0.056689	0.618371	10403
NDC80	TAK-715	-0.09858	0.00604	10403
NDC80	TG101348	-0.13313	0.000145	10403
NDC80	TGX221	0.152636	0.01163	10403
NDC80	THZ-2-102-1	-0.14468	4.09E-05	10403
NDC80	THZ-2-49	-0.05529	0.142429	10403
NDC80	TL-1-85	-0.11064	0.001886	10403
NDC80	TL-2-105	-0.10437	0.004182	10403
NDC80	TPCA-1	-0.12909	0.000213	10403
NDC80	TW 37	-0.06621	0.129175	10403
NDC80	Talazoparib	-0.00789	0.887018	10403
NDC80	Tamoxifen	-0.01644	0.912118	10403
NDC80	Temozolomide	0.001683	0.987981	10403
NDC80	Temsirolimus	-0.06375	0.174252	10403
NDC80	Thapsigargin	-0.04798	0.445474	10403
NDC80	Tipifarnib	0.03112	0.665256	10403
NDC80	Tivozanib	-0.06466	0.209977	10403
NDC80	Trametinib	0.272103	0	10403
NDC80	Tubastatin A	-0.06577	0.06994	10403
NDC80	UNC0638	-0.09437	0.00715	10403
NDC80	UNC1215	0.092146	0.04162	10403
NDC80	VNLG/124	-0.01965	0.677795	10403
NDC80	VX-11e	0.132578	0.00106	10403
NDC80	VX-680	-0.06814	0.462417	10403
NDC80	VX-702	-0.02131	0.934547	10403

NDC80	Veliparib	0.002338	0.98004	10403
NDC80	Vinblastine	-0.10165	0.013031	10403
NDC80	Vinorelbine	-0.10129	0.032328	10403
NDC80	Vorinostat	-0.16152	7E-06	10403
NDC80	WH-4-023	0.035975	0.678813	10403
NDC80	WZ-1-84	0.050151	0.539403	10403
NDC80	WZ3105	-0.13633	9.42E-05	10403
NDC80	XAV939	0.030969	0.454662	10403
NDC80	XL-184	-0.0243	0.641172	10403
NDC80	XMD11-85h	0.033065	0.862989	10403
NDC80	XMD13-2	-0.10117	0.004528	10403
NDC80	XMD14-99	0.015202	0.726968	10403
NDC80	XMD15-27	-0.0356	0.474581	10403
NDC80	XMD8-85	0.016504	0.875329	10403
NDC80	XMD8-92	-0.00198	0.989489	10403
NDC80	Y-39983	-0.11765	0.001146	10403
NDC80	YK 4-279	0.010626	0.879142	10403
NDC80	YM155	-0.09855	0.026912	10403
NDC80	YM201636	-0.09141	0.011892	10403
NDC80	Z-LLNle-CHO	-0.02332	0.790488	10403
NDC80	ZG-10	-0.08732	0.164395	10403
NDC80	ZM-447439	-0.09048	0.034801	10403
NDC80	ZSTK474	-0.11513	0.001247	10403
NDC80	Zibotentan	0.005217	0.991093	10403
NDC80	piperlongumine	0.021249	0.668708	10403
NDC80	rTRAIL	0.030231	0.706907	10403
NDC80	selumetinib	0.271311	0	10403
NEIL3	(5Z)-7-Oxozeaenol	0.10982	0.003823	55247
NEIL3	17-AAG	0.102539	0.005564	55247
NEIL3	5-Fluorouracil	-0.06468	0.080614	55247
NEIL3	681640	-0.04518	0.458097	55247
NEIL3	A-443654	-0.06807	0.669833	55247
NEIL3	A-770041	-0.02099	0.831808	55247
NEIL3	AC220	-0.04626	0.333178	55247
NEIL3	AG-014699	0.050776	0.237132	55247
NEIL3	AICAR	-0.18069	1.31E-06	55247
NEIL3	AKT inhibitor VIII	0.062388	0.159641	55247
NEIL3	AMG-706	0.010052	0.900605	55247
NEIL3	AP-24534	-0.115	0.002925	55247
NEIL3	AR-42	-0.13713	9.59E-05	55247
NEIL3	AS601245	0.030308	0.607103	55247
NEIL3	AS605240	-0.04156	0.352307	55247
NEIL3	AT-7519	-0.1926	2.23E-08	55247
NEIL3	ATRA	-0.1074	0.012142	55247
NEIL3	AUY922	-0.01929	0.756878	55247
NEIL3	AZ628	0.047161	0.56315	55247
NEIL3	AZD6482	-0.00796	0.897668	55247
NEIL3	AZD7762	-0.24159	4.03E-11	55247
NEIL3	AZD8055	-0.16339	1.33E-05	55247
NEIL3	Afatinib	0.130676	0.000231	55247

NEIL3	Axitinib	-0.08795	0.057734	55247
NEIL3	BAY 61-3606	-0.07529	0.06134	55247
NEIL3	BEZ235	-0.07623	0.092592	55247
NEIL3	BHG712	-0.13478	0.000132	55247
NEIL3	BI-2536	-0.16587	0.05253	55247
NEIL3	BIRB 0796	0.052385	0.304456	55247
NEIL3	BIX02189	-0.21009	1.23E-09	55247
NEIL3	BMS-509744	0.000131	0.999989	55247
NEIL3	BMS-536924	-0.00806	0.934271	55247
NEIL3	BMS-708163	0.09777	0.011948	55247
NEIL3	BMS-754807	0.056621	0.282955	55247
NEIL3	BMS345541	-0.2214	1.04E-10	55247
NEIL3	BX-795	-0.17362	8.49E-06	55247
NEIL3	BX-912	-0.24836	1.94E-13	55247
NEIL3	Belinostat	-0.10681	0.003471	55247
NEIL3	Bexarotene	-0.03634	0.640536	55247
NEIL3	Bicalutamide	0.016043	0.758667	55247
NEIL3	Bleomycin	-0.0085	0.907619	55247
NEIL3	Bleomycin (50 uM)	0.05121	0.168628	55247
NEIL3	Bortezomib	-0.00311	0.978117	55247
NEIL3	Bosutinib	-0.08264	0.076041	55247
NEIL3	Bryostatin 1	0.017271	0.780819	55247
NEIL3	CAL-101	-0.13394	0.000201	55247
NEIL3	CAY10603	-0.14568	3.14E-05	55247
NEIL3	CCT007093	0.056554	0.191066	55247
NEIL3	CCT018159	-0.03538	0.51238	55247
NEIL3	CEP-701	-0.19271	2.14E-07	55247
NEIL3	CGP-082996	-0.08004	0.368451	55247
NEIL3	CGP-60474	-0.06549	0.445796	55247
NEIL3	CH5424802	-0.13838	0.000976	55247
NEIL3	CHIR-99021	0.015621	0.719938	55247
NEIL3	CI-1040	0.109907	0.006272	55247
NEIL3	CMK	-0.04918	0.69221	55247
NEIL3	CP466722	-0.22896	1.84E-11	55247
NEIL3	CP724714	0.09753	0.028728	55247
NEIL3	CUDC-101	-0.12476	0.000516	55247
NEIL3	CX-5461	-0.13801	0.000102	55247
NEIL3	Camptothecin	-0.18966	7.49E-07	55247
NEIL3	Cetuximab	0.104525	0.007215	55247
NEIL3	Cisplatin	-0.03368	0.518589	55247
NEIL3	Crizotinib	-0.11355	0.268738	55247
NEIL3	Cyclopamine	-0.0902	0.345783	55247
NEIL3	Cytarabine	-0.18673	4.93E-06	55247
NEIL3	DMOG	-0.19845	1.55E-07	55247
NEIL3	Dabrafenib	0.086788	0.032911	55247
NEIL3	Dasatinib	0.025399	0.737867	55247
NEIL3	Docetaxel	0.09123	0.014604	55247
NEIL3	Doxorubicin	-0.04467	0.482734	55247
NEIL3	EHT 1864	0.013435	0.880596	55247
NEIL3	EKB-569	-0.11593	0.002125	55247

NEIL3	EX-527	-0.02145	0.863096	55247
NEIL3	Elesclomol	0.002792	0.958997	55247
NEIL3	Embelin	-0.05005	0.382396	55247
NEIL3	Epothilone B	0.002482	0.969047	55247
NEIL3	Erlotinib	0.140806	0.037809	55247
NEIL3	Etoposide	-0.15814	9.39E-05	55247
NEIL3	FH535	0.081302	0.064897	55247
NEIL3	FK866	-0.17482	5.76E-07	55247
NEIL3	FMK	-0.09874	0.069192	55247
NEIL3	FR-180204	-0.04442	0.396976	55247
NEIL3	FTI-277	0.096957	0.015267	55247
NEIL3	Foretinib	-0.12796	0.000613	55247
NEIL3	GDC0449	-0.01094	0.937271	55247
NEIL3	GDC0941	-0.0798	0.096106	55247
NEIL3	GNF-2	-0.06277	0.780166	55247
NEIL3	GSK-650394	-0.07819	0.200022	55247
NEIL3	GSK1070916	-0.23193	1.99E-11	55247
NEIL3	GSK1904529A	0.077054	0.078663	55247
NEIL3	GSK2126458	-0.10967	0.002629	55247
NEIL3	GSK269962A	-0.05215	0.298044	55247
NEIL3	GSK429286A	-0.15234	3.99E-05	55247
NEIL3	GSK690693	-0.15702	8.55E-06	55247
NEIL3	GW 441756	-0.01824	0.948577	55247
NEIL3	GW-2580	0.031621	0.942331	55247
NEIL3	GW843682X	-0.17271	0.018349	55247
NEIL3	Gefitinib	0.116459	0.003013	55247
NEIL3	Gemcitabine	-0.15209	0.000304	55247
NEIL3	Genentech Cpd 10	-0.21569	4.16E-10	55247
NEIL3	HG-5-113-01	-0.11495	0.068875	55247
NEIL3	HG-5-88-01	-0.03765	0.821768	55247
NEIL3	HG-6-64-1	-0.06482	0.154946	55247
NEIL3	I-BET-762	-0.20353	2.09E-09	55247
NEIL3	IOX2	0.043401	0.456112	55247
NEIL3	IPA-3	-0.14175	0.000148	55247
NEIL3	Imatinib	-0.07743	0.552973	55247
NEIL3	Ispinesib Mesylate	-0.1477	2.78E-05	55247
NEIL3	JNJ-26854165	-0.0308	0.579497	55247
NEIL3	JNK Inhibitor VIII	0.038857	0.380726	55247
NEIL3	JNK-9L	-0.02814	0.652731	55247
NEIL3	JQ1	-0.07047	0.088636	55247
NEIL3	JQ12	-0.062	0.229972	55247
NEIL3	JW-7-24-1	-0.16462	2.08E-06	55247
NEIL3	JW-7-52-1	-0.05217	0.621014	55247
NEIL3	KIN001-055	0.007112	0.937802	55247
NEIL3	KIN001-102	-0.22405	4.91E-11	55247
NEIL3	KIN001-135	0.100736	0.269746	55247
NEIL3	KIN001-236	-0.19077	4.98E-08	55247
NEIL3	KIN001-244	-0.17462	6.8E-07	55247
NEIL3	KIN001-260	-0.17827	3.14E-07	55247
NEIL3	KIN001-266	-0.06544	0.151181	55247

NEIL3	KIN001-270	-0.11464	0.00218	55247
NEIL3	KU-55933	-0.11427	0.017579	55247
NEIL3	LAQ824	-0.10456	0.006209	55247
NEIL3	LFM-A13	0.002871	0.966018	55247
NEIL3	LY317615	-0.15581	5.02E-05	55247
NEIL3	Lapatinib	0.176275	0.00287	55247
NEIL3	Lenalidomide	0.011552	0.914616	55247
NEIL3	Linifanib	-0.03367	0.541234	55247
NEIL3	Lisitinib	0.045446	0.474193	55247
NEIL3	MG-132	0.019396	0.868005	55247
NEIL3	MK-2206	-0.12789	0.004357	55247
NEIL3	MLN4924	-0.00125	0.986131	55247
NEIL3	MP470	0.002307	0.965269	55247
NEIL3	MPS-1-IN-1	-0.13657	0.000157	55247
NEIL3	MS-275	-0.21263	0.001895	55247
NEIL3	Masitinib	-0.12167	0.000796	55247
NEIL3	Methotrexate	-0.31524	7.76E-20	55247
NEIL3	Midostaurin	-0.04654	0.307163	55247
NEIL3	Mitomycin C	-0.13044	0.002539	55247
NEIL3	NG-25	-0.14267	4.56E-05	55247
NEIL3	NPK76-II-72-1	-0.28002	4.5E-17	55247
NEIL3	NSC-207895	-0.08394	0.043376	55247
NEIL3	NSC-87877	0.095164	0.060861	55247
NEIL3	NU-7441	-0.05465	0.416049	55247
NEIL3	Navitoclax	-0.23854	1.54E-11	55247
NEIL3	Nilotinib	-0.10801	0.014158	55247
NEIL3	Nutlin-3a (-)	0.059716	0.198107	55247
NEIL3	OSI-027	-0.12793	0.000305	55247
NEIL3	OSI-930	-0.06986	0.07008	55247
NEIL3	OSU-03012	-0.09373	0.034122	55247
NEIL3	Obatoclax Mesylate	-0.04481	0.352285	55247
NEIL3	Olaparib	0.004552	0.934227	55247
NEIL3	PAC-1	-0.16094	2.5E-05	55247
NEIL3	PD-0325901	0.147791	8.91E-05	55247
NEIL3	PD-0332991	-0.13725	0.00196	55247
NEIL3	PD-173074	-0.03233	0.841615	55247
NEIL3	PF-4708671	0.070856	0.407267	55247
NEIL3	PF-562271	-0.02666	0.669433	55247
NEIL3	PFI-1	-0.04095	0.407747	55247
NEIL3	PHA-665752	0.057515	0.695998	55247
NEIL3	PHA-793887	-0.18313	8.72E-08	55247
NEIL3	PI-103	-0.13449	0.000141	55247
NEIL3	PIK-93	-0.24038	1.14E-12	55247
NEIL3	PLX4720	0.111476	0.003369	55247
NEIL3	Paclitaxel	-0.06126	0.580769	55247
NEIL3	Parthenolide	-0.11868	0.195667	55247
NEIL3	Pazopanib	-0.00301	0.968857	55247
NEIL3	Phenformin	-0.08908	0.015453	55247
NEIL3	Pyrimethamine	-0.10715	0.303232	55247
NEIL3	QL-VIII-58	-0.07315	0.310903	55247

NEIL3	QL-X-138	-0.15513	1.12E-05	55247
NEIL3	QL-XI-92	-0.19352	2.26E-08	55247
NEIL3	QL-XII-47	-0.10395	0.006339	55247
NEIL3	QL-XII-61	-0.14443	0.012249	55247
NEIL3	QS11	-0.07931	0.15059	55247
NEIL3	RDEA119	0.185242	7.38E-08	55247
NEIL3	RO-3306	0.024257	0.592755	55247
NEIL3	Rapamycin	-0.05619	0.642562	55247
NEIL3	Roscovitine	0.037619	0.847233	55247
NEIL3	Ruxolitinib	-0.07095	0.125817	55247
NEIL3	S-Trityl-L-cysteine	-0.10349	0.167208	55247
NEIL3	SB 216763	0.026101	0.626887	55247
NEIL3	SB 505124	0.010384	0.923217	55247
NEIL3	SB52334	0.069105	0.114729	55247
NEIL3	SB590885	0.135436	0.001225	55247
NEIL3	SGC0946	0.0526	0.307157	55247
NEIL3	SL 0101-1	0.054923	0.464551	55247
NEIL3	SN-38	-0.11747	0.002591	55247
NEIL3	SNX-2112	-0.18042	2.24E-07	55247
NEIL3	STF-62247	-0.17933	1.26E-06	55247
NEIL3	Salubrinal	-0.09142	0.254751	55247
NEIL3	Saracatinib	-0.0053	0.962621	55247
NEIL3	Shikonin	-0.05702	0.264275	55247
NEIL3	Sorafenib	-0.00652	0.964024	55247
NEIL3	Sunitinib	-0.1555	0.015469	55247
NEIL3	T0901317	-0.15393	3.64E-05	55247
NEIL3	TAE684	-0.06802	0.52933	55247
NEIL3	TAK-715	-0.16475	2.53E-06	55247
NEIL3	TG101348	-0.20265	3.41E-09	55247
NEIL3	TGX221	0.069031	0.294188	55247
NEIL3	THZ-2-102-1	-0.16395	2.94E-06	55247
NEIL3	THZ-2-49	-0.19796	1.72E-08	55247
NEIL3	TL-1-85	-0.15612	7.82E-06	55247
NEIL3	TL-2-105	-0.16302	4.28E-06	55247
NEIL3	TPCA-1	-0.20286	2.76E-09	55247
NEIL3	TW 37	-0.05935	0.183976	55247
NEIL3	Talazoparib	-0.05774	0.193042	55247
NEIL3	Tamoxifen	-0.04885	0.651968	55247
NEIL3	Temozolomide	-0.05127	0.448059	55247
NEIL3	Temsirolimus	-0.1082	0.010883	55247
NEIL3	Thapsigargin	0.003755	0.969469	55247
NEIL3	Tipifarnib	-0.01232	0.888045	55247
NEIL3	Tivozanib	-0.05981	0.259706	55247
NEIL3	Trametinib	0.24718	9.29E-13	55247
NEIL3	Tubastatin A	-0.17929	2.11E-07	55247
NEIL3	UNC0638	-0.14393	2.61E-05	55247
NEIL3	UNC1215	0.10334	0.020738	55247
NEIL3	VNLG/124	-0.13069	0.000551	55247
NEIL3	VX-11e	0.057739	0.210317	55247
NEIL3	VX-680	-0.18376	0.013724	55247

NEIL3	VX-702	-0.01567	0.95291	55247
NEIL3	Veliparib	0.03016	0.67977	55247
NEIL3	Vinblastine	-0.16962	1.32E-05	55247
NEIL3	Vinorelbine	-0.07554	0.127452	55247
NEIL3	Vorinostat	-0.21071	2.99E-09	55247
NEIL3	WH-4-023	0.011286	0.90772	55247
NEIL3	WZ-1-84	-0.00263	0.979391	55247
NEIL3	WZ3105	-0.20546	1.95E-09	55247
NEIL3	XAV939	0.034867	0.395484	55247
NEIL3	XL-184	-0.07642	0.077595	55247
NEIL3	XMD11-85h	-0.07957	0.584688	55247
NEIL3	XMD13-2	-0.17691	3.21E-07	55247
NEIL3	XMD14-99	-0.14085	9.83E-05	55247
NEIL3	XMD15-27	-0.12295	0.002521	55247
NEIL3	XMD8-85	-0.09669	0.203383	55247
NEIL3	XMD8-92	-0.06608	0.519468	55247
NEIL3	Y-39983	-0.21926	3.15E-10	55247
NEIL3	YK 4-279	-0.04834	0.367221	55247
NEIL3	YM155	-0.07232	0.124696	55247
NEIL3	YM201636	-0.17818	3.55E-07	55247
NEIL3	Z-LLNle-CHO	-0.06354	0.401461	55247
NEIL3	ZG-10	-0.13401	0.021366	55247
NEIL3	ZM-447439	-0.18113	5.79E-06	55247
NEIL3	ZSTK474	-0.16687	1.87E-06	55247
NEIL3	Zibotentan	-0.03712	0.882923	55247
NEIL3	piperlongumine	-0.00457	0.93724	55247
NEIL3	rTRAIL	1.89E-05	0.999712	55247
NEIL3	selumetinib	0.182279	1.34E-07	55247
NUF2	(5Z)-7-Oxozeaenol	0.162705	8.74E-06	83540
NUF2	17-AAG	0.227783	1.54E-10	83540
NUF2	5-Fluorouracil	-0.06662	0.071245	83540
NUF2	681640	0.049848	0.40394	83540
NUF2	A-443654	-0.05298	0.760534	83540
NUF2	A-770041	0.01898	0.851581	83540
NUF2	AC220	0.006006	0.929536	83540
NUF2	AG-014699	0.073514	0.076371	83540
NUF2	AICAR	-0.10785	0.005085	83540
NUF2	AKT inhibitor VIII	0.141846	0.000494	83540
NUF2	AMG-706	0.088902	0.07972	83540
NUF2	AP-24534	-0.0203	0.662331	83540
NUF2	AR-42	-0.17706	3.47E-07	83540
NUF2	AS601245	0.119576	0.007344	83540
NUF2	AS605240	0.006055	0.910254	83540
NUF2	AT-7519	-0.16648	1.57E-06	83540
NUF2	ATRA	-0.02665	0.625477	83540
NUF2	AUY922	0.069444	0.149924	83540
NUF2	AZ628	0.17033	0.007428	83540
NUF2	AZD6482	0.069122	0.107129	83540
NUF2	AZD7762	-0.11839	0.00208	83540
NUF2	AZD8055	-0.07368	0.061778	83540

NUF2	Afatinib	0.180199	2.02E-07	83540
NUF2	Axitinib	-0.01557	0.815308	83540
NUF2	BAY 61-3606	-0.03945	0.358731	83540
NUF2	BEZ235	0.043796	0.379213	83540
NUF2	BHG712	-0.07158	0.050363	83540
NUF2	BI-2536	-0.07598	0.440105	83540
NUF2	BIRB 0796	0.091712	0.051497	83540
NUF2	BIX02189	-0.0924	0.010754	83540
NUF2	BMS-509744	0.030174	0.771611	83540
NUF2	BMS-536924	0.009703	0.921981	83540
NUF2	BMS-708163	0.127746	0.000859	83540
NUF2	BMS-754807	0.042569	0.443814	83540
NUF2	BMS345541	-0.15843	5.41E-06	83540
NUF2	BX-795	-0.07005	0.109463	83540
NUF2	BX-912	-0.18041	1.53E-07	83540
NUF2	Belinostat	-0.15483	1.53E-05	83540
NUF2	Bexarotene	0.046535	0.515252	83540
NUF2	Bicalutamide	0.048918	0.274571	83540
NUF2	Bleomycin	0.089502	0.063785	83540
NUF2	Bleomycin (50 uM)	0.14388	4.55E-05	83540
NUF2	Bortezomib	0.064653	0.419821	83540
NUF2	Bosutinib	0.032438	0.573958	83540
NUF2	Bryostatin 1	0.088674	0.057302	83540
NUF2	CAL-101	-0.09132	0.01345	83540
NUF2	CAY10603	-0.18161	1.55E-07	83540
NUF2	CCT007093	0.126602	0.001507	83540
NUF2	CCT018159	0.027985	0.619462	83540
NUF2	CEP-701	-0.0685	0.091096	83540
NUF2	CGP-082996	-0.00513	0.970049	83540
NUF2	CGP-60474	-0.01939	0.856763	83540
NUF2	CH5424802	-0.06476	0.216892	83540
NUF2	CHIR-99021	0.040315	0.311278	83540
NUF2	CI-1040	0.16167	3.05E-05	83540
NUF2	CMK	0.006306	0.967793	83540
NUF2	CP466722	-0.16033	3.92E-06	83540
NUF2	CP724714	0.084297	0.068323	83540
NUF2	CUDC-101	-0.15179	1.96E-05	83540
NUF2	CX-5461	-0.14833	2.74E-05	83540
NUF2	Camptothecin	-0.08851	0.032169	83540
NUF2	Cetuximab	0.132588	0.000512	83540
NUF2	Cisplatin	0.002099	0.974797	83540
NUF2	Crizotinib	-0.04128	0.79553	83540
NUF2	Cyclopamine	-0.0143	0.9213	83540
NUF2	Cytarabine	-0.01325	0.829197	83540
NUF2	DMOG	-0.07193	0.095228	83540
NUF2	Dabrafenib	0.142381	0.000205	83540
NUF2	Dasatinib	0.094646	0.148764	83540
NUF2	Docetaxel	0.145777	7.08E-05	83540
NUF2	Doxorubicin	0.020384	0.788906	83540
NUF2	EHT 1864	0.094537	0.077927	83540

NUF2	EKB-569	-0.00663	0.891261	83540
NUF2	EX-527	0.019796	0.874962	83540
NUF2	Elesclomol	0.008778	0.869613	83540
NUF2	Embelin	0.035293	0.573292	83540
NUF2	Epothilone B	0.064444	0.188613	83540
NUF2	Erlotinib	0.140869	0.037694	83540
NUF2	Etoposide	-0.09986	0.018723	83540
NUF2	FH535	0.118389	0.004614	83540
NUF2	FK866	-0.2099	1.36E-09	83540
NUF2	FMK	0.009983	0.910838	83540
NUF2	FR-180204	0.039034	0.467557	83540
NUF2	FTI-277	0.16547	1.79E-05	83540
NUF2	Foretinib	-0.07787	0.049359	83540
NUF2	GDC0449	0.047872	0.61033	83540
NUF2	GDC0941	-0.00983	0.89148	83540
NUF2	GNF-2	0.023843	0.934378	83540
NUF2	GSK-650394	0.033807	0.664782	83540
NUF2	GSK1070916	-0.17975	3.01E-07	83540
NUF2	GSK1904529A	0.090151	0.036601	83540
NUF2	GSK2126458	-0.09806	0.007592	83540
NUF2	GSK269962A	0.029018	0.613706	83540
NUF2	GSK429286A	-0.11382	0.002821	83540
NUF2	GSK690693	-0.18195	1.94E-07	83540
NUF2	GW 441756	0.005418	0.991337	83540
NUF2	GW-2580	0.031551	0.942708	83540
NUF2	GW843682X	-0.10632	0.178086	83540
NUF2	Gefitinib	0.17209	5.63E-06	83540
NUF2	Gemcitabine	-0.05327	0.272849	83540
NUF2	Genentech Cpd 10	-0.16476	2.59E-06	83540
NUF2	HG-5-113-01	-0.06437	0.360153	83540
NUF2	HG-5-88-01	-0.0162	0.936049	83540
NUF2	HG-6-64-1	0.085463	0.048328	83540
NUF2	I-BET-762	-0.17858	1.72E-07	83540
NUF2	IOX2	0.019057	0.784304	83540
NUF2	IPA-3	-0.10791	0.00457	83540
NUF2	Imatinib	-0.0093	0.967994	83540
NUF2	Ispinesib Mesylate	-0.09897	0.006166	83540
NUF2	JNJ-26854165	0.097776	0.027365	83540
NUF2	JNK Inhibitor VIII	0.101124	0.01278	83540
NUF2	JNK-9L	0.042355	0.461654	83540
NUF2	JQ1	0.018899	0.696865	83540
NUF2	JQ12	0.007146	0.918477	83540
NUF2	JW-7-24-1	-0.15391	9.79E-06	83540
NUF2	JW-7-52-1	0.035646	0.753387	83540
NUF2	KIN001-055	0.073749	0.16525	83540
NUF2	KIN001-102	-0.14335	3.77E-05	83540
NUF2	KIN001-135	0.078203	0.420727	83540
NUF2	KIN001-236	-0.08895	0.015114	83540
NUF2	KIN001-244	-0.1247	0.00049	83540
NUF2	KIN001-260	-0.11925	0.000804	83540

NUF2	KIN001-266	-0.0439	0.365726	83540
NUF2	KIN001-270	-0.15663	1.94E-05	83540
NUF2	KU-55933	-0.03839	0.548267	83540
NUF2	LAQ824	-0.09669	0.011899	83540
NUF2	LFM-A13	0.077375	0.101175	83540
NUF2	LY317615	-0.04019	0.374791	83540
NUF2	Lapatinib	0.192914	0.000969	83540
NUF2	Lenalidomide	-0.00921	0.935175	83540
NUF2	Linifanib	0.049302	0.33057	83540
NUF2	Lisitinib	0.017453	0.826478	83540
NUF2	MG-132	0.051053	0.58694	83540
NUF2	MK-2206	-0.07165	0.140811	83540
NUF2	MLN4924	0.092814	0.071951	83540
NUF2	MP470	-0.09242	0.026791	83540
NUF2	MPS-1-IN-1	-0.10759	0.003311	83540
NUF2	MS-275	-0.15323	0.02856	83540
NUF2	Masitinib	-0.0676	0.073458	83540
NUF2	Methotrexate	-0.15478	1.79E-05	83540
NUF2	Midostaurin	0.065824	0.12995	83540
NUF2	Mitomycin C	-0.03095	0.578707	83540
NUF2	NG-25	-0.12205	0.00054	83540
NUF2	NPK76-II-72-1	-0.23595	2.5E-12	83540
NUF2	NSC-207895	-0.14632	0.000198	83540
NUF2	NSC-87877	0.132306	0.008567	83540
NUF2	NU-7441	-0.00516	0.964206	83540
NUF2	Navitoclax	-0.2146	1.64E-09	83540
NUF2	Nilotinib	-0.03406	0.535621	83540
NUF2	Nutlin-3a (-)	0.125343	0.002586	83540
NUF2	OSI-027	-0.14093	6.44E-05	83540
NUF2	OSI-930	-0.02114	0.625037	83540
NUF2	OSU-03012	-0.00765	0.906929	83540
NUF2	Obatoclax Mesylate	0.01577	0.779963	83540
NUF2	Olaparib	0.019163	0.697775	83540
NUF2	PAC-1	-0.08058	0.047874	83540
NUF2	PD-0325901	0.20995	1.27E-08	83540
NUF2	PD-0332991	0.041362	0.466998	83540
NUF2	PD-173074	0.019687	0.91378	83540
NUF2	PF-4708671	0.094885	0.233459	83540
NUF2	PF-562271	0.049408	0.371422	83540
NUF2	PFI-1	0.064245	0.160658	83540
NUF2	PHA-665752	0.123119	0.279668	83540
NUF2	PHA-793887	-0.17196	5.42E-07	83540
NUF2	PI-103	-0.16179	3.83E-06	83540
NUF2	PIK-93	-0.19256	1.81E-08	83540
NUF2	PLX4720	0.128169	0.000602	83540
NUF2	Paclitaxel	0.016579	0.914343	83540
NUF2	Parthenolide	-0.03905	0.733782	83540
NUF2	Pazopanib	0.081612	0.097254	83540
NUF2	Phenformin	-0.0891	0.015421	83540
NUF2	Pyrimethamine	-0.05329	0.650298	83540

NUF2	QL-VIII-58	7.13E-05	0.999404	83540
NUF2	QL-X-138	-0.18275	1.75E-07	83540
NUF2	QL-XI-92	-0.1463	3.11E-05	83540
NUF2	QL-XII-47	-0.11437	0.002467	83540
NUF2	QL-XII-61	-0.13227	0.02319	83540
NUF2	QS11	-0.07563	0.172021	83540
NUF2	RDEA119	0.257581	2.32E-14	83540
NUF2	RO-3306	0.104893	0.009571	83540
NUF2	Rapamycin	-0.04762	0.706514	83540
NUF2	Roscovitine	0.021764	0.918154	83540
NUF2	Ruxolitinib	-0.05546	0.255876	83540
NUF2	S-Trityl-L-cysteine	-0.05326	0.535606	83540
NUF2	SB 216763	0.110701	0.016195	83540
NUF2	SB 505124	0.082909	0.18123	83540
NUF2	SB52334	-0.00446	0.946446	83540
NUF2	SB590885	0.150236	0.000247	83540
NUF2	SGC0946	0.07842	0.09306	83540
NUF2	SL 0101-1	0.097856	0.185569	83540
NUF2	SN-38	-0.00749	0.89398	83540
NUF2	SNX-2112	-0.12105	0.000676	83540
NUF2	STF-62247	-0.06883	0.083686	83540
NUF2	Salubrinal	-0.00424	0.969435	83540
NUF2	Saracatinib	0.043908	0.623475	83540
NUF2	Shikonin	-0.04745	0.371499	83540
NUF2	Sorafenib	0.068745	0.508562	83540
NUF2	Sunitinib	0.000321	0.997857	83540
NUF2	T0901317	-0.11275	0.003288	83540
NUF2	TAE684	-0.02968	0.828112	83540
NUF2	TAK-715	-0.09101	0.011593	83540
NUF2	TG101348	-0.15244	1.18E-05	83540
NUF2	TGX221	0.176342	0.002999	83540
NUF2	THZ-2-102-1	-0.17154	9.44E-07	83540
NUF2	THZ-2-49	-0.09475	0.009403	83540
NUF2	TL-1-85	-0.10522	0.003237	83540
NUF2	TL-2-105	-0.11143	0.002128	83540
NUF2	TPCA-1	-0.15709	5.44E-06	83540
NUF2	TW 37	-0.05603	0.214433	83540
NUF2	Talazoparib	-0.02123	0.676773	83540
NUF2	Tamoxifen	0.051413	0.629552	83540
NUF2	Temozolomide	-0.00648	0.950274	83540
NUF2	Temsirolimus	-0.03706	0.476231	83540
NUF2	Thapsigargin	0.002309	0.981564	83540
NUF2	Tipifarnib	0.07888	0.152812	83540
NUF2	Tivozanib	-0.00079	0.992371	83540
NUF2	Trametinib	0.313489	0	83540
NUF2	Tubastatin A	-0.1512	1.44E-05	83540
NUF2	UNC0638	-0.14108	3.82E-05	83540
NUF2	UNC1215	0.113039	0.01105	83540
NUF2	VNLG/124	-0.02452	0.594115	83540
NUF2	VX-11e	0.10679	0.010545	83540

NUF2	VX-680	-0.06674	0.473567	83540
NUF2	VX-702	0.010488	0.974173	83540
NUF2	Veliparib	0.070056	0.270186	83540
NUF2	Vinblastine	-0.03672	0.431643	83540
NUF2	Vinorelbine	-0.0182	0.780593	83540
NUF2	Vorinostat	-0.22756	1.27E-10	83540
NUF2	WH-4-023	0.105495	0.142078	83540
NUF2	WZ-1-84	0.106752	0.139533	83540
NUF2	WZ3105	-0.15948	4.16E-06	83540
NUF2	XAV939	0.093791	0.013871	83540
NUF2	XL-184	0.013408	0.812025	83540
NUF2	XMD11-85h	0.034359	0.856377	83540
NUF2	XMD13-2	-0.13319	0.000151	83540
NUF2	XMD14-99	-0.08278	0.026818	83540
NUF2	XMD15-27	-0.05716	0.212045	83540
NUF2	XMD8-85	0.032667	0.730504	83540
NUF2	XMD8-92	-0.00076	0.995583	83540
NUF2	Y-39983	-0.16199	4.97E-06	83540
NUF2	YK 4-279	0.073819	0.134164	83540
NUF2	YM155	-0.10603	0.015856	83540
NUF2	YM201636	-0.14426	4.63E-05	83540
NUF2	Z-LLNle-CHO	0.050727	0.522248	83540
NUF2	ZG-10	-0.12286	0.037403	83540
NUF2	ZM-447439	-0.06407	0.1552	83540
NUF2	ZSTK474	-0.12502	0.000431	83540
NUF2	Zibotentan	0.033093	0.906877	83540
NUF2	piperlongumine	0.04197	0.350311	83540
NUF2	rTRAIL	0.069136	0.290128	83540
NUF2	selumetinib	0.276291	0	83540
OIP5	(5Z)-7-Oxozeaenol	0.226556	1.7E-10	11339
OIP5	17-AAG	0.130729	0.000348	11339
OIP5	5-Fluorouracil	-0.03031	0.43554	11339
OIP5	681640	0.000912	0.991215	11339
OIP5	A-443654	-0.05626	0.739341	11339
OIP5	A-770041	-0.02771	0.773135	11339
OIP5	AC220	-0.00998	0.875487	11339
OIP5	AG-014699	0.075907	0.066516	11339
OIP5	AICAR	-0.09231	0.017906	11339
OIP5	AKT inhibitor VIII	0.039789	0.398778	11339
OIP5	AMG-706	0.050285	0.389731	11339
OIP5	AP-24534	-0.00383	0.9425	11339
OIP5	AR-42	-0.10788	0.002413	11339
OIP5	AS601245	0.07467	0.12899	11339
OIP5	AS605240	0.019758	0.687749	11339
OIP5	AT-7519	-0.14106	5.46E-05	11339
OIP5	ATRA	-0.06807	0.142294	11339
OIP5	AUY922	0.056583	0.259112	11339
OIP5	AZ628	0.209782	0.00067	11339
OIP5	AZD6482	0.06511	0.134693	11339
OIP5	AZD7762	-0.0851	0.031383	11339

OIP5	AZD8055	-0.08983	0.020989	11339
OIP5	Afatinib	0.019505	0.635495	11339
OIP5	Axitinib	-0.04334	0.416752	11339
OIP5	BAY 61-3606	0.021579	0.634457	11339
OIP5	BEZ235	0.036669	0.474593	11339
OIP5	BHG712	-0.01981	0.616325	11339
OIP5	BI-2536	-0.10072	0.274119	11339
OIP5	BIRB 0796	0.039841	0.453045	11339
OIP5	BIX02189	-0.05678	0.129396	11339
OIP5	BMS-509744	0.050443	0.600638	11339
OIP5	BMS-536924	-0.00251	0.980269	11339
OIP5	BMS-708163	0.086477	0.027315	11339
OIP5	BMS-754807	0.050303	0.351869	11339
OIP5	BMS345541	-0.13715	9.26E-05	11339
OIP5	BX-795	-0.03001	0.549769	11339
OIP5	BX-912	-0.10618	0.002617	11339
OIP5	Belinostat	-0.0764	0.040214	11339
OIP5	Bexarotene	-0.0067	0.956133	11339
OIP5	Bicalutamide	0.030863	0.519045	11339
OIP5	Bleomycin	0.092119	0.054489	11339
OIP5	Bleomycin (50 uM)	0.146552	3.22E-05	11339
OIP5	Bortezomib	0.048388	0.571302	11339
OIP5	Bosutinib	-0.0547	0.284176	11339
OIP5	Bryostatin 1	0.06704	0.170407	11339
OIP5	CAL-101	-0.04485	0.252931	11339
OIP5	CAY10603	-0.0874	0.014769	11339
OIP5	CCT007093	0.033442	0.472079	11339
OIP5	CCT018159	-0.00192	0.979625	11339
OIP5	CEP-701	-0.03038	0.490197	11339
OIP5	CGP-082996	-0.01743	0.889477	11339
OIP5	CGP-60474	0.022073	0.836056	11339
OIP5	CH5424802	-0.01333	0.861929	11339
OIP5	CHIR-99021	0.134993	0.000174	11339
OIP5	CI-1040	0.150011	0.000118	11339
OIP5	CMK	0.050004	0.685618	11339
OIP5	CP466722	-0.10986	0.001923	11339
OIP5	CP724714	0.015298	0.816984	11339
OIP5	CUDC-101	-0.12704	0.000401	11339
OIP5	CX-5461	-0.05339	0.156881	11339
OIP5	Camptothecin	-0.00633	0.912627	11339
OIP5	Cetuximab	0.059465	0.146758	11339
OIP5	Cisplatin	0.033285	0.523797	11339
OIP5	Crizotinib	0.002461	0.992818	11339
OIP5	Cyclopamine	-0.0153	0.915736	11339
OIP5	Cytarabine	0.028686	0.606827	11339
OIP5	DMOG	-0.02847	0.567457	11339
OIP5	Dabrafenib	0.173604	3.71E-06	11339
OIP5	Dasatinib	0.064641	0.346218	11339
OIP5	Docetaxel	0.033603	0.393815	11339
OIP5	Doxorubicin	0.049649	0.422216	11339

OIP5	EHT 1864	0.001805	0.985214	11339
OIP5	EKB-569	-0.0825	0.034091	11339
OIP5	EX-527	-0.01785	0.888041	11339
OIP5	Elesclomol	-0.02071	0.680574	11339
OIP5	Embelin	0.054071	0.335576	11339
OIP5	Epothilone B	-0.01282	0.83585	11339
OIP5	Erlotinib	0.075445	0.302049	11339
OIP5	Etoposide	-0.03389	0.507703	11339
OIP5	FH535	0.062394	0.176803	11339
OIP5	FK866	-0.13734	0.000104	11339
OIP5	FMK	0.061901	0.321841	11339
OIP5	FR-180204	0.036897	0.498234	11339
OIP5	FTI-277	0.149547	0.000115	11339
OIP5	Foretinib	-0.03136	0.483521	11339
OIP5	GDC0449	0.017635	0.889536	11339
OIP5	GDC0941	-0.02985	0.630206	11339
OIP5	GNF-2	-0.01104	0.977257	11339
OIP5	GSK-650394	-0.01838	0.840566	11339
OIP5	GSK1070916	-0.12353	0.000578	11339
OIP5	GSK1904529A	0.030204	0.540441	11339
OIP5	GSK2126458	-0.07503	0.044874	11339
OIP5	GSK269962A	0.075452	0.095969	11339
OIP5	GSK429286A	-0.03469	0.432811	11339
OIP5	GSK690693	-0.13304	0.000188	11339
OIP5	GW 441756	-0.01119	0.976257	11339
OIP5	GW-2580	0.040961	0.907972	11339
OIP5	GW843682X	-0.14268	0.055385	11339
OIP5	Gefitinib	0.006113	0.899058	11339
OIP5	Gemcitabine	-0.02896	0.591886	11339
OIP5	Genentech Cpd 10	-0.11736	0.001055	11339
OIP5	HG-5-113-01	-0.04549	0.547319	11339
OIP5	HG-5-88-01	0.047681	0.756958	11339
OIP5	HG-6-64-1	0.062016	0.176954	11339
OIP5	I-BET-762	-0.10861	0.001827	11339
OIP5	IOX2	0.040196	0.49646	11339
OIP5	IPA-3	-0.02148	0.6237	11339
OIP5	Imatinib	-0.01014	0.964787	11339
OIP5	Ispinesib Mesylate	-0.11658	0.001125	11339
OIP5	JNJ-26854165	0.056214	0.254277	11339
OIP5	JNK Inhibitor VIII	0.031553	0.487168	11339
OIP5	JNK-9L	0.034884	0.562474	11339
OIP5	JQ1	0.019696	0.683712	11339
OIP5	JQ12	0.008934	0.897416	11339
OIP5	JW-7-24-1	-0.10762	0.002369	11339
OIP5	JW-7-52-1	0.00131	0.993455	11339
OIP5	KIN001-055	0.00808	0.928642	11339
OIP5	KIN001-102	-0.12882	0.000228	11339
OIP5	KIN001-135	0.150716	0.07548	11339
OIP5	KIN001-236	-0.05366	0.157698	11339
OIP5	KIN001-244	-0.0536	0.155879	11339

OIP5	KIN001-260	-0.06056	0.101227	11339
OIP5	KIN001-266	-0.00902	0.876731	11339
OIP5	KIN001-270	-0.08875	0.020115	11339
OIP5	KU-55933	-0.05502	0.336901	11339
OIP5	LAQ824	-0.08891	0.021511	11339
OIP5	LFM-A13	0.058729	0.230581	11339
OIP5	LY317615	-0.04489	0.314641	11339
OIP5	Lapatinib	0.088761	0.159982	11339
OIP5	Lenalidomide	-0.08202	0.165533	11339
OIP5	Linifanib	0.025754	0.660807	11339
OIP5	Lisitinib	0.025502	0.730919	11339
OIP5	MG-132	0.036373	0.720305	11339
OIP5	MK-2206	-0.08856	0.060726	11339
OIP5	MLN4924	0.076857	0.146856	11339
OIP5	MP470	-0.03302	0.468502	11339
OIP5	MPS-1-IN-1	-0.04356	0.266477	11339
OIP5	MS-275	-0.12809	0.075515	11339
OIP5	Masitinib	-0.04625	0.234896	11339
OIP5	Methotrexate	-0.10595	0.003934	11339
OIP5	Midostaurin	0.1031	0.012427	11339
OIP5	Mitomycin C	-0.02504	0.665518	11339
OIP5	NG-25	-0.07927	0.028389	11339
OIP5	NPK76-II-72-1	-0.19032	2.31E-08	11339
OIP5	NSC-207895	-0.10953	0.006449	11339
OIP5	NSC-87877	0.094196	0.064113	11339
OIP5	NU-7441	0.01099	0.915241	11339
OIP5	Navitoclax	-0.13064	0.00035	11339
OIP5	Nilotinib	-0.03516	0.52033	11339
OIP5	Nutlin-3a (-)	0.186332	2.96E-06	11339
OIP5	OSI-027	-0.09621	0.007366	11339
OIP5	OSI-930	-0.01349	0.7639	11339
OIP5	OSU-03012	-0.01037	0.869075	11339
OIP5	Obatoclax Mesylate	0.042173	0.386325	11339
OIP5	Olaparib	0.084959	0.040898	11339
OIP5	PAC-1	-0.01998	0.680796	11339
OIP5	PD-0325901	0.205085	2.87E-08	11339
OIP5	PD-0332991	0.089743	0.060734	11339
OIP5	PD-173074	0.002902	0.992679	11339
OIP5	PF-4708671	0.05445	0.546323	11339
OIP5	PF-562271	0.043738	0.437783	11339
OIP5	PFI-1	0.073159	0.102927	11339
OIP5	PHA-665752	0.102846	0.413395	11339
OIP5	PHA-793887	-0.11622	0.000864	11339
OIP5	PI-103	-0.07601	0.036692	11339
OIP5	PIK-93	-0.12863	0.000224	11339
OIP5	PLX4720	0.133918	0.000316	11339
OIP5	Paclitaxel	-0.02748	0.838716	11339
OIP5	Parthenolide	-0.02516	0.839241	11339
OIP5	Pazopanib	0.064988	0.206328	11339
OIP5	Phenformin	-0.079	0.032949	11339

OIP5	Pyrimethamine	-0.01374	0.926526	11339
OIP5	QL-VIII-58	0.045636	0.576159	11339
OIP5	QL-X-138	-0.12101	0.00073	11339
OIP5	QL-XI-92	-0.08311	0.021596	11339
OIP5	QL-XII-47	-0.08635	0.02536	11339
OIP5	QL-XII-61	-0.06913	0.29263	11339
OIP5	QS11	-0.03033	0.652928	11339
OIP5	RDEA119	0.23575	3.91E-12	11339
OIP5	RO-3306	0.013596	0.774304	11339
OIP5	Rapamycin	-0.02671	0.860326	11339
OIP5	Roscovitine	0.053254	0.756324	11339
OIP5	Ruxolitinib	-0.02842	0.61758	11339
OIP5	S-Trityl-L-cysteine	-0.06213	0.454756	11339
OIP5	SB 216763	0.06324	0.194493	11339
OIP5	SB 505124	0.050673	0.473878	11339
OIP5	SB52334	0.041088	0.400869	11339
OIP5	SB590885	0.149957	0.000255	11339
OIP5	SGC0946	0.061889	0.210961	11339
OIP5	SL 0101-1	0.068904	0.345451	11339
OIP5	SN-38	0.073146	0.07874	11339
OIP5	SNX-2112	-0.07811	0.032761	11339
OIP5	STF-62247	-0.03837	0.363334	11339
OIP5	Salubrinal	-0.05066	0.571052	11339
OIP5	Saracatinib	-0.02824	0.764819	11339
OIP5	Shikonin	0.031066	0.590092	11339
OIP5	Sorafenib	0.033831	0.790956	11339
OIP5	Sunitinib	-0.02731	0.755817	11339
OIP5	T0901317	-0.08073	0.041168	11339
OIP5	TAE684	0.033705	0.801229	11339
OIP5	TAK-715	-0.11459	0.001294	11339
OIP5	TG101348	-0.08679	0.01537	11339
OIP5	TGX221	0.133822	0.029196	11339
OIP5	THZ-2-102-1	-0.13795	9.53E-05	11339
OIP5	THZ-2-49	-0.06811	0.067022	11339
OIP5	TL-1-85	-0.07156	0.050303	11339
OIP5	TL-2-105	-0.07306	0.049845	11339
OIP5	TPCA-1	-0.1109	0.001588	11339
OIP5	TW 37	-0.03651	0.453487	11339
OIP5	Talazoparib	0.082444	0.050364	11339
OIP5	Tamoxifen	0.033603	0.787996	11339
OIP5	Temozolomide	0.032014	0.672955	11339
OIP5	Temsirolimus	-0.02708	0.622609	11339
OIP5	Thapsigargin	0.010772	0.904402	11339
OIP5	Tipifarnib	0.029455	0.685621	11339
OIP5	Tivozanib	0.001261	0.987102	11339
OIP5	Trametinib	0.277302	0	11339
OIP5	Tubastatin A	-0.05631	0.12399	11339
OIP5	UNC0638	-0.0265	0.484549	11339
OIP5	UNC1215	0.080887	0.0799	11339
OIP5	VNLG/124	-0.03137	0.480957	11339

OIP5	VX-11e	0.110779	0.007591	11339
OIP5	VX-680	-0.06137	0.516278	11339
OIP5	VX-702	-0.05023	0.781002	11339
OIP5	Veliparib	-0.0111	0.896633	11339
OIP5	Vinblastine	-0.06736	0.117278	11339
OIP5	Vinorelbine	-0.04679	0.39395	11339
OIP5	Vorinostat	-0.17307	1.37E-06	11339
OIP5	WH-4-023	0.069699	0.369852	11339
OIP5	WZ-1-84	0.022133	0.808426	11339
OIP5	WZ3105	-0.12677	0.000298	11339
OIP5	XAV939	0.053827	0.17405	11339
OIP5	XL-184	0.004383	0.942639	11339
OIP5	XMD11-85h	0.093195	0.481812	11339
OIP5	XMD13-2	-0.11224	0.001548	11339
OIP5	XMD14-99	0.027762	0.500626	11339
OIP5	XMD15-27	-0.01074	0.853428	11339
OIP5	XMD8-85	0.049405	0.570249	11339
OIP5	XMD8-92	0.031061	0.798567	11339
OIP5	Y-39983	-0.06748	0.073094	11339
OIP5	YK 4-279	0.014412	0.831595	11339
OIP5	YM155	-0.05003	0.325564	11339
OIP5	YM201636	-0.09026	0.013095	11339
OIP5	Z-LLNle-CHO	0.041679	0.608991	11339
OIP5	ZG-10	-0.04219	0.555294	11339
OIP5	ZM-447439	-0.0649	0.149094	11339
OIP5	ZSTK474	-0.09626	0.007501	11339
OIP5	Zibotentan	0.016751	0.96343	11339
OIP5	piperlongumine	0.069748	0.095505	11339
OIP5	rTRAIL	0.043122	0.555049	11339
OIP5	selumetinib	0.246905	3.36E-13	11339
PLK4	(5Z)-7-Oxozeaenol	0.120223	0.001401	10733
PLK4	17-AAG	0.212566	2.63E-09	10733
PLK4	5-Fluorouracil	-0.17115	1.08E-06	10733
PLK4	681640	0.006478	0.930495	10733
PLK4	A-443654	-0.07387	0.637899	10733
PLK4	A-770041	-0.04545	0.604231	10733
PLK4	AC220	-0.05368	0.245159	10733
PLK4	AG-014699	0.100638	0.012872	10733
PLK4	AICAR	-0.224	1.1E-09	10733
PLK4	AKT inhibitor VIII	0.057326	0.200919	10733
PLK4	AMG-706	0.078165	0.134388	10733
PLK4	AP-24534	-0.17464	2.67E-06	10733
PLK4	AR-42	-0.23519	6.49E-12	10733
PLK4	AS601245	0.032226	0.578848	10733
PLK4	AS605240	-0.08039	0.04786	10733
PLK4	AT-7519	-0.28238	4.79E-17	10733
PLK4	ATRA	-0.07796	0.08424	10733
PLK4	AUY922	-0.04885	0.343914	10733
PLK4	AZ628	0.09177	0.199487	10733
PLK4	AZD6482	-0.03297	0.511309	10733

PLK4	AZD7762	-0.25486	2.57E-12	10733
PLK4	AZD8055	-0.19651	1.19E-07	10733
PLK4	Afatinib	0.142108	5.62E-05	10733
PLK4	Axitinib	-0.08985	0.051644	10733
PLK4	BAY 61-3606	-0.14852	9.41E-05	10733
PLK4	BEZ235	-0.00181	0.977768	10733
PLK4	BHG712	-0.22536	5.01E-11	10733
PLK4	BI-2536	-0.20829	0.015438	10733
PLK4	BIRB 0796	0.10207	0.027966	10733
PLK4	BIX02189	-0.264	9.72E-15	10733
PLK4	BMS-509744	0.019153	0.862352	10733
PLK4	BMS-536924	0.018889	0.822797	10733
PLK4	BMS-708163	0.138464	0.000285	10733
PLK4	BMS-754807	0.081787	0.097188	10733
PLK4	BMS345541	-0.28533	2.48E-17	10733
PLK4	BX-795	-0.12154	0.002736	10733
PLK4	BX-912	-0.2877	7.55E-18	10733
PLK4	Belinostat	-0.20099	1.25E-08	10733
PLK4	Bexarotene	0.018482	0.843961	10733
PLK4	Bicalutamide	0.07912	0.061363	10733
PLK4	Bleomycin	-0.02033	0.754806	10733
PLK4	Bleomycin (50 uM)	0.143073	5.03E-05	10733
PLK4	Bortezomib	-0.0066	0.952725	10733
PLK4	Bosutinib	-0.10056	0.024649	10733
PLK4	Bryostatin 1	0.104346	0.022633	10733
PLK4	CAL-101	-0.21264	1.07E-09	10733
PLK4	CAY10603	-0.23848	2.82E-12	10733
PLK4	CCT007093	0.113429	0.004716	10733
PLK4	CCT018159	-0.0025	0.974022	10733
PLK4	CEP-701	-0.18151	1.15E-06	10733
PLK4	CGP-082996	-0.052	0.60496	10733
PLK4	CGP-60474	-0.08128	0.317599	10733
PLK4	CH5424802	-0.09887	0.035007	10733
PLK4	CHIR-99021	0.094576	0.010783	10733
PLK4	CI-1040	0.10416	0.010055	10733
PLK4	CMK	-0.08341	0.432572	10733
PLK4	CP466722	-0.28213	4.72E-17	10733
PLK4	CP724714	0.064016	0.19892	10733
PLK4	CUDC-101	-0.22689	6.51E-11	10733
PLK4	CX-5461	-0.22987	2.72E-11	10733
PLK4	Camptothecin	-0.132	0.000804	10733
PLK4	Cetuximab	0.150644	6.51E-05	10733
PLK4	Cisplatin	0.033424	0.521717	10733
PLK4	Crizotinib	-0.10649	0.3171	10733
PLK4	Cyclopamine	-0.08538	0.38028	10733
PLK4	Cytarabine	-0.09884	0.0262	10733
PLK4	DMOG	-0.15668	5.28E-05	10733
PLK4	Dabrafenib	0.122863	0.001599	10733
PLK4	Dasatinib	0.021796	0.776926	10733
PLK4	Docetaxel	0.170379	2.95E-06	10733

PLK4	Doxorubicin	-0.06599	0.248597	10733
PLK4	EHT 1864	0.059231	0.328239	10733
PLK4	EKB-569	-0.1639	8.15E-06	10733
PLK4	EX-527	0.024606	0.840666	10733
PLK4	Elesclomol	-0.00026	0.995665	10733
PLK4	Embelin	-0.04787	0.407311	10733
PLK4	Epothilone B	0.008624	0.892274	10733
PLK4	Erlotinib	0.202085	0.001583	10733
PLK4	Etoposide	-0.16625	3.91E-05	10733
PLK4	FH535	0.084622	0.052914	10733
PLK4	FK866	-0.24846	4.63E-13	10733
PLK4	FMK	-0.12646	0.012063	10733
PLK4	FR-180204	-0.07381	0.117749	10733
PLK4	FTI-277	0.172936	6.81E-06	10733
PLK4	Foretinib	-0.1627	8.96E-06	10733
PLK4	GDC0449	-0.00928	0.948573	10733
PLK4	GDC0941	-0.09957	0.029214	10733
PLK4	GNF-2	-0.05245	0.829085	10733
PLK4	GSK-650394	-0.07657	0.212011	10733
PLK4	GSK1070916	-0.28798	2.97E-17	10733
PLK4	GSK1904529A	0.097444	0.022745	10733
PLK4	GSK2126458	-0.21502	7.19E-10	10733
PLK4	GSK269962A	-0.00177	0.980495	10733
PLK4	GSK429286A	-0.17493	1.8E-06	10733
PLK4	GSK690693	-0.25068	2.62E-13	10733
PLK4	GW 441756	-0.0071	0.989034	10733
PLK4	GW-2580	0.020094	0.967521	10733
PLK4	GW843682X	-0.22892	0.001699	10733
PLK4	Gefitinib	0.149231	0.000103	10733
PLK4	Gemcitabine	-0.14662	0.000515	10733
PLK4	Genentech Cpd 10	-0.25962	3.03E-14	10733
PLK4	HG-5-113-01	-0.12241	0.050638	10733
PLK4	HG-5-88-01	-0.02864	0.875654	10733
PLK4	HG-6-64-1	-0.05142	0.280609	10733
PLK4	I-BET-762	-0.30529	3.29E-20	10733
PLK4	IOX2	0.072944	0.167757	10733
PLK4	IPA-3	-0.21053	7.55E-09	10733
PLK4	Imatinib	-0.10748	0.309363	10733
PLK4	Ispinesib Mesylate	-0.23525	7.5E-12	10733
PLK4	JNJ-26854165	0.027719	0.626294	10733
PLK4	JNK Inhibitor VIII	0.125006	0.001714	10733
PLK4	JNK-9L	-0.03884	0.507984	10733
PLK4	JQ1	-0.0642	0.123685	10733
PLK4	JQ12	-0.08296	0.092197	10733
PLK4	JW-7-24-1	-0.23539	4.55E-12	10733
PLK4	JW-7-52-1	-0.05328	0.61077	10733
PLK4	KIN001-055	0.008538	0.924731	10733
PLK4	KIN001-102	-0.29662	6.89E-19	10733
PLK4	KIN001-135	0.16561	0.049515	10733
PLK4	KIN001-236	-0.21515	5.89E-10	10733

PLK4	KIN001-244	-0.25067	3.17E-13	10733
PLK4	KIN001-260	-0.20025	7.28E-09	10733
PLK4	KIN001-266	-0.07929	0.074995	10733
PLK4	KIN001-270	-0.17236	2.28E-06	10733
PLK4	KU-55933	-0.12159	0.01044	10733
PLK4	LAQ824	-0.19176	1.63E-07	10733
PLK4	LFM-A13	0.075798	0.109442	10733
PLK4	LY317615	-0.13965	0.000326	10733
PLK4	Lapatinib	0.195528	0.000806	10733
PLK4	Lenalidomide	-0.05892	0.377518	10733
PLK4	Linifanib	-0.03968	0.456049	10733
PLK4	Lisitinib	0.057053	0.338518	10733
PLK4	MG-132	0.004069	0.977186	10733
PLK4	MK-2206	-0.15313	0.000457	10733
PLK4	MLN4924	0.057385	0.301114	10733
PLK4	MP470	-0.0804	0.055509	10733
PLK4	MPS-1-IN-1	-0.14158	8.55E-05	10733
PLK4	MS-275	-0.18396	0.007411	10733
PLK4	Masitinib	-0.18347	2.07E-07	10733
PLK4	Methotrexate	-0.27804	1.84E-15	10733
PLK4	Midostaurin	0.053226	0.232022	10733
PLK4	Mitomycin C	-0.12094	0.005323	10733
PLK4	NG-25	-0.25747	2.97E-14	10733
PLK4	NPK76-II-72-1	-0.36312	7.37E-29	10733
PLK4	NSC-207895	-0.15286	9.78E-05	10733
PLK4	NSC-87877	0.106177	0.034686	10733
PLK4	NU-7441	-0.06475	0.316319	10733
PLK4	Navitoclax	-0.28852	1.49E-16	10733
PLK4	Nilotinib	-0.13072	0.002232	10733
PLK4	Nutlin-3a (-)	0.067164	0.139935	10733
PLK4	OSI-027	-0.2194	1.97E-10	10733
PLK4	OSI-930	-0.13572	0.000214	10733
PLK4	OSU-03012	-0.11488	0.007616	10733
PLK4	Obatoclax Mesylate	-0.11036	0.008349	10733
PLK4	Olaparib	0.045618	0.303699	10733
PLK4	PAC-1	-0.22853	6.64E-10	10733
PLK4	PD-0325901	0.226768	6.73E-10	10733
PLK4	PD-0332991	-0.02315	0.720325	10733
PLK4	PD-173074	-0.00663	0.982618	10733
PLK4	PF-4708671	0.054726	0.545355	10733
PLK4	PF-562271	0.046881	0.399507	10733
PLK4	PFI-1	-0.00795	0.89749	10733
PLK4	PHA-665752	0.043229	0.780391	10733
PLK4	PHA-793887	-0.28799	5.67E-18	10733
PLK4	PI-103	-0.24162	1.79E-12	10733
PLK4	PIK-93	-0.33068	1.15E-23	10733
PLK4	PLX4720	0.137265	0.000211	10733
PLK4	Paclitaxel	-0.07638	0.454517	10733
PLK4	Parthenolide	-0.12067	0.186583	10733
PLK4	Pazopanib	0.062141	0.231156	10733

PLK4	Phenformin	-0.16568	3.38E-06	10733
PLK4	Pyrimethamine	-0.05424	0.645324	10733
PLK4	QL-VIII-58	-0.02363	0.801362	10733
PLK4	QL-X-138	-0.24342	1.53E-12	10733
PLK4	QL-XI-92	-0.25567	5.75E-14	10733
PLK4	QL-XII-47	-0.16229	1.04E-05	10733
PLK4	QL-XII-61	-0.19467	0.000424	10733
PLK4	QS11	-0.10411	0.049442	10733
PLK4	RDEA119	0.233497	6.35E-12	10733
PLK4	RO-3306	0.099574	0.014201	10733
PLK4	Rapamycin	-0.10657	0.285452	10733
PLK4	Roscovitine	0.027601	0.896026	10733
PLK4	Ruxolitinib	-0.08274	0.065262	10733
PLK4	S-Trityl-L-cysteine	-0.1699	0.015543	10733
PLK4	SB 216763	0.084236	0.075124	10733
PLK4	SB 505124	0.054361	0.435165	10733
PLK4	SB52334	0.012435	0.836164	10733
PLK4	SB590885	0.155592	0.000131	10733
PLK4	SGC0946	0.084936	0.064687	10733
PLK4	SL 0101-1	0.061753	0.400464	10733
PLK4	SN-38	-0.06536	0.123305	10733
PLK4	SNX-2112	-0.27861	2.25E-16	10733
PLK4	STF-62247	-0.1751	2.4E-06	10733
PLK4	Salubrinal	-0.11719	0.128927	10733
PLK4	Saracatinib	-0.00852	0.936506	10733
PLK4	Shikonin	-0.09371	0.043967	10733
PLK4	Sorafenib	-0.02707	0.835504	10733
PLK4	Sunitinib	-0.14408	0.026756	10733
PLK4	T0901317	-0.17736	1.42E-06	10733
PLK4	TAE684	-0.0092	0.956447	10733
PLK4	TAK-715	-0.23838	4.15E-12	10733
PLK4	TG101348	-0.25683	3.07E-14	10733
PLK4	TGX221	0.130879	0.033547	10733
PLK4	THZ-2-102-1	-0.27313	1.21E-15	10733
PLK4	THZ-2-49	-0.21659	5.62E-10	10733
PLK4	TL-1-85	-0.24266	1.07E-12	10733
PLK4	TL-2-105	-0.204	5.42E-09	10733
PLK4	TPCA-1	-0.2922	2.05E-18	10733
PLK4	TW 37	-0.03156	0.528366	10733
PLK4	Talazoparib	-0.00191	0.973504	10733
PLK4	Tamoxifen	0.001792	0.993474	10733
PLK4	Temozolomide	-0.02805	0.719372	10733
PLK4	Temsirolimus	-0.10493	0.013949	10733
PLK4	Thapsigargin	-0.00833	0.926096	10733
PLK4	Tipifarnib	-0.03872	0.569552	10733
PLK4	Tivozanib	-0.08333	0.085064	10733
PLK4	Trametinib	0.280172	0	10733
PLK4	Tubastatin A	-0.23677	3.57E-12	10733
PLK4	UNC0638	-0.16703	8.75E-07	10733
PLK4	UNC1215	0.134677	0.002384	10733

PLK4	VNLG/124	-0.09157	0.019387	10733
PLK4	VX-11e	0.023189	0.670934	10733
PLK4	VX-680	-0.18217	0.014376	10733
PLK4	VX-702	-0.01993	0.939314	10733
PLK4	Veliparib	0.044751	0.512902	10733
PLK4	Vinblastine	-0.12369	0.002026	10733
PLK4	Vinorelbine	-0.11407	0.014521	10733
PLK4	Vorinostat	-0.28147	8.4E-16	10733
PLK4	WH-4-023	0.055171	0.495354	10733
PLK4	WZ-1-84	0.082072	0.281438	10733
PLK4	WZ3105	-0.27134	8.34E-16	10733
PLK4	XAV939	0.085748	0.025367	10733
PLK4	XL-184	-0.09819	0.018248	10733
PLK4	XMD11-85h	-0.03371	0.85892	10733
PLK4	XMD13-2	-0.23977	1.88E-12	10733
PLK4	XMD14-99	-0.14221	8.35E-05	10733
PLK4	XMD15-27	-0.12651	0.001814	10733
PLK4	XMD8-85	-0.06681	0.419305	10733
PLK4	XMD8-92	-0.0626	0.551204	10733
PLK4	Y-39983	-0.25528	1.45E-13	10733
PLK4	YK 4-279	-0.00342	0.965391	10733
PLK4	YM155	-0.10944	0.012394	10733
PLK4	YM201636	-0.2404	2.92E-12	10733
PLK4	Z-LLNle-CHO	-0.10271	0.13794	10733
PLK4	ZG-10	-0.14627	0.01099	10733
PLK4	ZM-447439	-0.16745	3E-05	10733
PLK4	ZSTK474	-0.2579	4.09E-14	10733
PLK4	Zibotentan	-0.00785	0.987731	10733
PLK4	piperlongumine	0.025387	0.601259	10733
PLK4	rTRAIL	0.038166	0.612319	10733
PLK4	selumetinib	0.243104	8.16E-13	10733
RAD51	(5Z)-7-Oxozeaenol	0.132468	0.000378	5888
RAD51	17-AAG	0.148426	4.38E-05	5888
RAD51	5-Fluorouracil	-0.1299	0.000263	5888
RAD51	681640	-0.03252	0.610518	5888
RAD51	A-443654	-0.03528	0.864752	5888
RAD51	A-770041	-0.03111	0.742687	5888
RAD51	AC220	-0.05603	0.221487	5888
RAD51	AG-014699	0.091444	0.025021	5888
RAD51	AICAR	-0.22194	1.57E-09	5888
RAD51	AKT inhibitor VIII	0.063848	0.148698	5888
RAD51	AMG-706	0.041345	0.502945	5888
RAD51	AP-24534	-0.10757	0.005612	5888
RAD51	AR-42	-0.1648	2.27E-06	5888
RAD51	AS601245	0.068723	0.170718	5888
RAD51	AS605240	-0.0449	0.308547	5888
RAD51	AT-7519	-0.21159	6.58E-10	5888
RAD51	ATRA	-0.13372	0.001228	5888
RAD51	AUY922	0.030326	0.598416	5888
RAD51	AZ628	0.09252	0.1955	5888

RAD51	AZD6482	0.007257	0.90731	5888
RAD51	AZD7762	-0.20691	2.22E-08	5888
RAD51	AZD8055	-0.16475	1.11E-05	5888
RAD51	Afatinib	0.017807	0.667506	5888
RAD51	Axitinib	-0.11889	0.007295	5888
RAD51	BAY 61-3606	-0.03206	0.463646	5888
RAD51	BEZ235	0.033539	0.519012	5888
RAD51	BHG712	-0.15952	4.95E-06	5888
RAD51	BI-2536	-0.11934	0.18157	5888
RAD51	BIRB 0796	0.074996	0.121067	5888
RAD51	BIX02189	-0.17016	1.16E-06	5888
RAD51	BMS-509744	0.04168	0.672272	5888
RAD51	BMS-536924	-0.00098	0.991699	5888
RAD51	BMS-708163	0.072163	0.068377	5888
RAD51	BMS-754807	0.064796	0.208516	5888
RAD51	BMS345541	-0.22681	3.42E-11	5888
RAD51	BX-795	-0.10387	0.012023	5888
RAD51	BX-912	-0.20941	8.24E-10	5888
RAD51	Belinostat	-0.12425	0.000605	5888
RAD51	Bexarotene	0.007203	0.952291	5888
RAD51	Bicalutamide	0.059794	0.171487	5888
RAD51	Bleomycin	0.078208	0.114918	5888
RAD51	Bleomycin (50 uM)	0.181611	1.97E-07	5888
RAD51	Bortezomib	0.048549	0.569832	5888
RAD51	Bosutinib	-0.10584	0.016961	5888
RAD51	Bryostatin 1	0.073432	0.126198	5888
RAD51	CAL-101	-0.12902	0.000352	5888
RAD51	CAY10603	-0.15277	1.21E-05	5888
RAD51	CCT007093	0.038678	0.396239	5888
RAD51	CCT018159	-0.00582	0.935485	5888
RAD51	CEP-701	-0.14182	0.000195	5888
RAD51	CGP-082996	-0.04276	0.682769	5888
RAD51	CGP-60474	0.034118	0.726717	5888
RAD51	CH5424802	-0.05909	0.2697	5888
RAD51	CHIR-99021	0.102253	0.005495	5888
RAD51	CI-1040	0.067159	0.116778	5888
RAD51	CMK	-0.02262	0.87513	5888
RAD51	CP466722	-0.20341	3.01E-09	5888
RAD51	CP724714	-0.02606	0.669863	5888
RAD51	CUDC-101	-0.17663	5.45E-07	5888
RAD51	CX-5461	-0.15018	2.14E-05	5888
RAD51	Camptothecin	-0.06248	0.1482	5888
RAD51	Cetuximab	0.07258	0.071164	5888
RAD51	Cisplatin	0.0521	0.290262	5888
RAD51	Crizotinib	-0.06396	0.644904	5888
RAD51	Cyclopamine	-0.0512	0.650581	5888
RAD51	Cytarabine	-0.03533	0.510435	5888
RAD51	DMOG	-0.05104	0.26008	5888
RAD51	Dabrafenib	0.140349	0.000258	5888
RAD51	Dasatinib	0.041561	0.564423	5888

RAD51	Docetaxel	0.093473	0.012289	5888
RAD51	Doxorubicin	0.033022	0.629628	5888
RAD51	EHT 1864	-0.01357	0.879546	5888
RAD51	EKB-569	-0.19185	1.2E-07	5888
RAD51	EX-527	0.015716	0.90505	5888
RAD51	Elesclomol	0.006712	0.90162	5888
RAD51	Embelin	0.025394	0.709407	5888
RAD51	Epothilone B	0.018802	0.749421	5888
RAD51	Erlotinib	0.102558	0.14947	5888
RAD51	Etoposide	-0.04313	0.375595	5888
RAD51	FH535	0.095083	0.026813	5888
RAD51	FK866	-0.1466	3.24E-05	5888
RAD51	FMK	-0.00883	0.921523	5888
RAD51	FR-180204	-0.05743	0.246113	5888
RAD51	FTI-277	0.16788	1.31E-05	5888
RAD51	Foretinib	-0.09346	0.015686	5888
RAD51	GDC0449	-0.01585	0.90393	5888
RAD51	GDC0941	-0.06019	0.243718	5888
RAD51	GNF-2	-0.09491	0.604754	5888
RAD51	GSK-650394	-0.01492	0.876887	5888
RAD51	GSK1070916	-0.22926	3.47E-11	5888
RAD51	GSK1904529A	0.0797	0.067733	5888
RAD51	GSK2126458	-0.15581	1.24E-05	5888
RAD51	GSK269962A	0.047294	0.353757	5888
RAD51	GSK429286A	-0.08659	0.027588	5888
RAD51	GSK690693	-0.19321	2.88E-08	5888
RAD51	GW 441756	-0.01885	0.947563	5888
RAD51	GW-2580	0.04457	0.888023	5888
RAD51	GW843682X	-0.15169	0.0396	5888
RAD51	Gefitinib	0.034628	0.424675	5888
RAD51	Gemcitabine	-0.04211	0.405594	5888
RAD51	Genentech Cpd 10	-0.1863	8.64E-08	5888
RAD51	HG-5-113-01	-0.08288	0.215811	5888
RAD51	HG-5-88-01	-0.03077	0.867689	5888
RAD51	HG-6-64-1	0.007625	0.901778	5888
RAD51	I-BET-762	-0.23295	5.11E-12	5888
RAD51	IOX2	0.071169	0.180294	5888
RAD51	IPA-3	-0.10519	0.005784	5888
RAD51	Imatinib	-0.12196	0.20875	5888
RAD51	Ispinesib Mesylate	-0.16907	1.36E-06	5888
RAD51	JNJ-26854165	0.046585	0.360377	5888
RAD51	JNK Inhibitor VIII	0.064878	0.124973	5888
RAD51	JNK-9L	0.000242	0.998097	5888
RAD51	JQ1	-0.08176	0.044309	5888
RAD51	JQ12	-0.0399	0.475546	5888
RAD51	JW-7-24-1	-0.17486	4.3E-07	5888
RAD51	JW-7-52-1	-0.03354	0.773804	5888
RAD51	KIN001-055	-0.00747	0.935319	5888
RAD51	KIN001-102	-0.22563	3.53E-11	5888
RAD51	KIN001-135	0.129967	0.134889	5888

RAD51	KIN001-236	-0.15797	8.03E-06	5888
RAD51	KIN001-244	-0.15203	1.74E-05	5888
RAD51	KIN001-260	-0.13777	9.64E-05	5888
RAD51	KIN001-266	-0.05392	0.250899	5888
RAD51	KIN001-270	-0.09508	0.012321	5888
RAD51	KU-55933	-0.08644	0.090252	5888
RAD51	LAQ824	-0.13986	0.000183	5888
RAD51	LFM-A13	0.086228	0.064215	5888
RAD51	LY317615	-0.13133	0.000807	5888
RAD51	Lapatinib	0.081947	0.197635	5888
RAD51	Lenalidomide	-0.11245	0.032663	5888
RAD51	Linifanib	-0.03431	0.533389	5888
RAD51	Lisitinib	0.032781	0.635984	5888
RAD51	MG-132	0.060046	0.507676	5888
RAD51	MK-2206	-0.13314	0.002896	5888
RAD51	MLN4924	0.072462	0.17532	5888
RAD51	MP470	-0.07334	0.082259	5888
RAD51	MPS-1-IN-1	-0.10691	0.003531	5888
RAD51	MS-275	-0.18131	0.008536	5888
RAD51	Masitinib	-0.16404	4.05E-06	5888
RAD51	Methotrexate	-0.22522	1.98E-10	5888
RAD51	Midostaurin	0.080234	0.058281	5888
RAD51	Mitomycin C	-0.04402	0.396407	5888
RAD51	NG-25	-0.19618	1.19E-08	5888
RAD51	NPK76-II-72-1	-0.27688	1.07E-16	5888
RAD51	NSC-207895	-0.09945	0.014606	5888
RAD51	NSC-87877	0.101264	0.044829	5888
RAD51	NU-7441	-0.02507	0.767069	5888
RAD51	Navitoclax	-0.22781	1.33E-10	5888
RAD51	Nilotinib	-0.141	0.00086	5888
RAD51	Nutlin-3a (-)	0.072269	0.108186	5888
RAD51	OSI-027	-0.13472	0.000138	5888
RAD51	OSI-930	-0.10509	0.004882	5888
RAD51	OSU-03012	-0.05164	0.292446	5888
RAD51	Obatoclax Mesylate	-0.02003	0.714459	5888
RAD51	Olaparib	0.104435	0.010492	5888
RAD51	PAC-1	-0.12412	0.001498	5888
RAD51	PD-0325901	0.163131	1.32E-05	5888
RAD51	PD-0332991	-0.02838	0.647788	5888
RAD51	PD-173074	-0.02768	0.871207	5888
RAD51	PF-4708671	0.019672	0.882403	5888
RAD51	PF-562271	0.032034	0.595656	5888
RAD51	PFI-1	-0.00651	0.916342	5888
RAD51	PHA-665752	0.068316	0.628361	5888
RAD51	PHA-793887	-0.20398	2.06E-09	5888
RAD51	PI-103	-0.13537	0.000127	5888
RAD51	PIK-93	-0.24136	9.14E-13	5888
RAD51	PLX4720	0.127279	0.000664	5888
RAD51	Paclitaxel	-0.03204	0.805677	5888
RAD51	Parthenolide	-0.05325	0.619122	5888

RAD51	Pazopanib	0.089722	0.063253	5888
RAD51	Phenformin	-0.12822	0.000382	5888
RAD51	Pyrimethamine	-0.06297	0.586723	5888
RAD51	QL-VIII-58	0.035657	0.674851	5888
RAD51	QL-X-138	-0.16249	4E-06	5888
RAD51	QL-XI-92	-0.18074	1.97E-07	5888
RAD51	QL-XII-47	-0.10698	0.004853	5888
RAD51	QL-XII-61	-0.125	0.033782	5888
RAD51	QS11	-0.05354	0.371718	5888
RAD51	RDEA119	0.175183	3.98E-07	5888
RAD51	RO-3306	0.071465	0.086158	5888
RAD51	Rapamycin	-0.03361	0.813116	5888
RAD51	Roscovitine	0.089971	0.552846	5888
RAD51	Ruxolitinib	-0.04811	0.337993	5888
RAD51	S-Trityl-L-cysteine	-0.11037	0.136544	5888
RAD51	SB 216763	0.057497	0.244077	5888
RAD51	SB 505124	0.03103	0.701542	5888
RAD51	SB52334	0.055598	0.224676	5888
RAD51	SB590885	0.137873	0.000958	5888
RAD51	SGC0946	0.108573	0.014486	5888
RAD51	SL 0101-1	0.051621	0.488917	5888
RAD51	SN-38	0.024165	0.625172	5888
RAD51	SNX-2112	-0.1641	2.82E-06	5888
RAD51	STF-62247	-0.0997	0.010082	5888
RAD51	Salubrinal	-0.0904	0.261185	5888
RAD51	Saracatinib	-0.04845	0.581468	5888
RAD51	Shikonin	-0.05479	0.287687	5888
RAD51	Sorafenib	-0.04037	0.744451	5888
RAD51	Sunitinib	-0.10962	0.109004	5888
RAD51	T0901317	-0.11013	0.00416	5888
RAD51	TAE684	0.015484	0.922325	5888
RAD51	TAK-715	-0.18383	1.3E-07	5888
RAD51	TG101348	-0.21082	7.12E-10	5888
RAD51	TGX221	0.167174	0.00519	5888
RAD51	THZ-2-102-1	-0.22058	1.66E-10	5888
RAD51	THZ-2-49	-0.13813	0.000114	5888
RAD51	TL-1-85	-0.19606	1.31E-08	5888
RAD51	TL-2-105	-0.14436	5.33E-05	5888
RAD51	TPCA-1	-0.22177	6.58E-11	5888
RAD51	TW 37	-0.02169	0.681251	5888
RAD51	Talazoparib	0.101003	0.013463	5888
RAD51	Tamoxifen	0.0045	0.978961	5888
RAD51	Temozolomide	0.022935	0.781401	5888
RAD51	Temsirolimus	-0.04605	0.358811	5888
RAD51	Thapsigargin	0.015243	0.857043	5888
RAD51	Tipifarnib	0.012242	0.889165	5888
RAD51	Tivozanib	-0.05525	0.30951	5888
RAD51	Trametinib	0.231586	2.63E-11	5888
RAD51	Tubastatin A	-0.15318	1.09E-05	5888
RAD51	UNC0638	-0.12899	0.00018	5888

RAD51	UNC1215	0.113382	0.010822	5888
RAD51	VNLG/124	-0.10086	0.009313	5888
RAD51	VX-11e	0.020933	0.70393	5888
RAD51	VX-680	-0.11357	0.170316	5888
RAD51	VX-702	-0.03516	0.883641	5888
RAD51	Veliparib	-0.00158	0.986855	5888
RAD51	Vinblastine	-0.12496	0.001805	5888
RAD51	Vinorelbine	-0.06441	0.20948	5888
RAD51	Vorinostat	-0.25421	5.03E-13	5888
RAD51	WH-4-023	0.027967	0.757692	5888
RAD51	WZ-1-84	0.032237	0.712454	5888
RAD51	WZ3105	-0.22686	2.7E-11	5888
RAD51	XAV939	0.084766	0.027162	5888
RAD51	XL-184	-0.0628	0.161824	5888
RAD51	XMD11-85h	0.036729	0.847526	5888
RAD51	XMD13-2	-0.18155	1.51E-07	5888
RAD51	XMD14-99	-0.07269	0.054395	5888
RAD51	XMD15-27	-0.09017	0.034482	5888
RAD51	XMD8-85	-0.0294	0.762014	5888
RAD51	XMD8-92	-0.02044	0.88257	5888
RAD51	Y-39983	-0.16187	5.06E-06	5888
RAD51	YK 4-279	-0.01338	0.844447	5888
RAD51	YM155	-0.03614	0.507832	5888
RAD51	YM201636	-0.1449	4.27E-05	5888
RAD51	Z-LLNle-CHO	-0.01649	0.856486	5888
RAD51	ZG-10	-0.11356	0.057888	5888
RAD51	ZM-447439	-0.17	2.21E-05	5888
RAD51	ZSTK474	-0.1742	6.16E-07	5888
RAD51	Zibotentan	0.001809	0.996163	5888
RAD51	piperlongumine	0.071366	0.086788	5888
RAD51	rTRAIL	0.030177	0.70773	5888
RAD51	selumetinib	0.198965	7.11E-09	5888
RTKN2	(5Z)-7-Oxozeaenol	0.193435	7.62E-08	219790
RTKN2	17-AAG	0.172436	1.74E-06	219790
RTKN2	5-Fluorouracil	0.037738	0.3255	219790
RTKN2	681640	0.023431	0.724326	219790
RTKN2	A-443654	-0.01459	0.954968	219790
RTKN2	A-770041	0.006643	0.952131	219790
RTKN2	AC220	0.043175	0.373501	219790
RTKN2	AG-014699	0.014701	0.765685	219790
RTKN2	AICAR	-0.02127	0.629817	219790
RTKN2	AKT inhibitor VIII	-0.03337	0.490336	219790
RTKN2	AMG-706	0.079389	0.127122	219790
RTKN2	AP-24534	0.06083	0.140306	219790
RTKN2	AR-42	-0.06944	0.055781	219790
RTKN2	AS601245	0.044549	0.41381	219790
RTKN2	AS605240	0.032777	0.476979	219790
RTKN2	AT-7519	-0.05833	0.110756	219790
RTKN2	ATRA	0.059558	0.210004	219790
RTKN2	AUY922	0.09277	0.041259	219790

RTKN2	AZ628	0.196636	0.001605	219790
RTKN2	AZD6482	0.038492	0.426698	219790
RTKN2	AZD7762	0.037448	0.380631	219790
RTKN2	AZD8055	0.066713	0.093339	219790
RTKN2	Afatinib	0.027179	0.496806	219790
RTKN2	Axitinib	0.064154	0.194654	219790
RTKN2	BAY 61-3606	0.021342	0.638434	219790
RTKN2	BEZ235	0.113758	0.007351	219790
RTKN2	BHG712	0.035916	0.34891	219790
RTKN2	BI-2536	-0.0501	0.65126	219790
RTKN2	BIRB 0796	0.046848	0.365426	219790
RTKN2	BIX02189	0.013515	0.74336	219790
RTKN2	BMS-509744	-0.02372	0.827079	219790
RTKN2	BMS-536924	0.028196	0.716304	219790
RTKN2	BMS-708163	-0.015	0.742167	219790
RTKN2	BMS-754807	-0.05949	0.25533	219790
RTKN2	BMS345541	-0.05234	0.15712	219790
RTKN2	BX-795	0.038111	0.4294	219790
RTKN2	BX-912	-0.07398	0.039431	219790
RTKN2	Belinostat	-0.07074	0.058781	219790
RTKN2	Bexarotene	0.109678	0.032741	219790
RTKN2	Bicalutamide	0.002955	0.959079	219790
RTKN2	Bleomycin	0.071938	0.154048	219790
RTKN2	Bleomycin (50 uM)	0.137493	0.000101	219790
RTKN2	Bortezomib	0.145258	0.02949	219790
RTKN2	Bosutinib	0.08188	0.079088	219790
RTKN2	Bryostatin 1	0.053218	0.296792	219790
RTKN2	CAL-101	-0.00869	0.843933	219790
RTKN2	CAY10603	-0.05243	0.155059	219790
RTKN2	CCT007093	0.028504	0.548137	219790
RTKN2	CCT018159	0.124798	0.004499	219790
RTKN2	CEP-701	0.07445	0.06398	219790
RTKN2	CGP-082996	-0.00879	0.947569	219790
RTKN2	CGP-60474	0.094982	0.222486	219790
RTKN2	CH5424802	-0.0027	0.974594	219790
RTKN2	CHIR-99021	0.054041	0.164198	219790
RTKN2	CI-1040	0.167965	1.41E-05	219790
RTKN2	CMK	0.033003	0.807913	219790
RTKN2	CP466722	-0.05604	0.127288	219790
RTKN2	CP724714	-0.05862	0.24893	219790
RTKN2	CUDC-101	-0.08123	0.027037	219790
RTKN2	CX-5461	-0.08383	0.021865	219790
RTKN2	Camptothecin	0.032884	0.489338	219790
RTKN2	Cetuximab	0.053728	0.193511	219790
RTKN2	Cisplatin	0.034788	0.503927	219790
RTKN2	Crizotinib	0.09195	0.421909	219790
RTKN2	Cyclopamine	0.074852	0.458899	219790
RTKN2	Cytarabine	0.043567	0.398529	219790
RTKN2	DMOG	0.018369	0.729721	219790
RTKN2	Dabrafenib	0.125092	0.00129	219790

RTKN2	Dasatinib	0.066545	0.33	219790
RTKN2	Docetaxel	0.144734	8.01E-05	219790
RTKN2	Doxorubicin	0.027286	0.701161	219790
RTKN2	EHT 1864	0.057718	0.341996	219790
RTKN2	EKB-569	-0.06269	0.116759	219790
RTKN2	EX-527	0.023498	0.849445	219790
RTKN2	Elesclomol	0.047924	0.29072	219790
RTKN2	Embelin	0.040366	0.505029	219790
RTKN2	Epothilone B	0.012009	0.849324	219790
RTKN2	Erlotinib	0.022196	0.786386	219790
RTKN2	Etoposide	0.016203	0.777876	219790
RTKN2	FH535	0.129101	0.001829	219790
RTKN2	FK866	-0.0487	0.195684	219790
RTKN2	FMK	-0.0069	0.940099	219790
RTKN2	FR-180204	0.009482	0.891587	219790
RTKN2	FTI-277	0.090182	0.02478	219790
RTKN2	Foretinib	-0.02196	0.639849	219790
RTKN2	GDC0449	0.08364	0.273525	219790
RTKN2	GDC0941	0.00785	0.913933	219790
RTKN2	GNF-2	0.046938	0.85036	219790
RTKN2	GSK-650394	-0.00923	0.929924	219790
RTKN2	GSK1070916	-0.1321	0.00022	219790
RTKN2	GSK1904529A	-0.03435	0.476614	219790
RTKN2	GSK2126458	-0.06935	0.065259	219790
RTKN2	GSK269962A	0.072841	0.111008	219790
RTKN2	GSK429286A	0.000614	0.990509	219790
RTKN2	GSK690693	-0.11805	0.001001	219790
RTKN2	GW 441756	0.037638	0.848405	219790
RTKN2	GW-2580	-0.04526	0.879403	219790
RTKN2	GW843682X	-0.04906	0.605013	219790
RTKN2	Gefitinib	0.050819	0.226934	219790
RTKN2	Gemcitabine	0.017135	0.765511	219790
RTKN2	Genentech Cpd 10	-0.10093	0.00529	219790
RTKN2	HG-5-113-01	-0.06864	0.322107	219790
RTKN2	HG-5-88-01	-0.0359	0.830258	219790
RTKN2	HG-6-64-1	0.071555	0.109154	219790
RTKN2	I-BET-762	-0.00319	0.937496	219790
RTKN2	IOX2	0.043142	0.45922	219790
RTKN2	IPA-3	0.012624	0.782533	219790
RTKN2	Imatinib	0.070433	0.614803	219790
RTKN2	Ispinesib Mesylate	-0.04808	0.205573	219790
RTKN2	JNJ-26854165	0.094407	0.034218	219790
RTKN2	JNK Inhibitor VIII	0.061023	0.151486	219790
RTKN2	JNK-9L	0.057389	0.280743	219790
RTKN2	JQ1	0.044366	0.309334	219790
RTKN2	JQ12	0.023541	0.699943	219790
RTKN2	JW-7-24-1	-0.03689	0.328854	219790
RTKN2	JW-7-52-1	0.078451	0.403215	219790
RTKN2	KIN001-055	0.016126	0.847417	219790
RTKN2	KIN001-102	-0.08066	0.023899	219790

RTKN2	KIN001-135	0.020504	0.876037	219790
RTKN2	KIN001-236	-0.03678	0.346298	219790
RTKN2	KIN001-244	0.006553	0.88033	219790
RTKN2	KIN001-260	0.046178	0.220915	219790
RTKN2	KIN001-266	-0.07341	0.10325	219790
RTKN2	KIN001-270	-0.0435	0.28586	219790
RTKN2	KU-55933	0.019492	0.794189	219790
RTKN2	LAQ824	-0.05806	0.148032	219790
RTKN2	LFM-A13	0.056112	0.255233	219790
RTKN2	LY317615	-0.05539	0.201846	219790
RTKN2	Lapatinib	0.020888	0.76659	219790
RTKN2	Lenalidomide	0.06873	0.27615	219790
RTKN2	Linifanib	0.068146	0.146604	219790
RTKN2	Lisitinib	-0.03054	0.666344	219790
RTKN2	MG-132	0.161584	0.022708	219790
RTKN2	MK-2206	-0.03338	0.54349	219790
RTKN2	MLN4924	0.123189	0.013869	219790
RTKN2	MP470	-0.06227	0.145026	219790
RTKN2	MPS-1-IN-1	-0.04868	0.210428	219790
RTKN2	MS-275	-0.03066	0.749469	219790
RTKN2	Masitinib	0.05923	0.120553	219790
RTKN2	Methotrexate	-0.00641	0.879824	219790
RTKN2	Midostaurin	0.061444	0.160116	219790
RTKN2	Mitomycin C	-0.00024	0.99718	219790
RTKN2	NG-25	0.018545	0.641198	219790
RTKN2	NPK76-II-72-1	-0.08508	0.016217	219790
RTKN2	NSC-207895	-0.08474	0.04118	219790
RTKN2	NSC-87877	0.02598	0.674704	219790
RTKN2	NU-7441	0.03536	0.643902	219790
RTKN2	Navitoclax	-0.13439	0.00023	219790
RTKN2	Nilotinib	0.085108	0.063614	219790
RTKN2	Nutlin-3a (-)	0.137948	0.000801	219790
RTKN2	OSI-027	-0.04986	0.1815	219790
RTKN2	OSI-930	0.039242	0.334385	219790
RTKN2	OSU-03012	-0.00782	0.904584	219790
RTKN2	Obatoclax Mesylate	0.053363	0.256573	219790
RTKN2	Olaparib	0.029415	0.528483	219790
RTKN2	PAC-1	-0.04219	0.33589	219790
RTKN2	PD-0325901	0.174931	2.7E-06	219790
RTKN2	PD-0332991	0.015688	0.820455	219790
RTKN2	PD-173074	0.02491	0.892985	219790
RTKN2	PF-4708671	0.044096	0.646626	219790
RTKN2	PF-562271	0.011923	0.863507	219790
RTKN2	PFI-1	0.093845	0.029631	219790
RTKN2	PHA-665752	0.074406	0.591928	219790
RTKN2	PHA-793887	-0.04256	0.246274	219790
RTKN2	PI-103	-0.04184	0.267957	219790
RTKN2	PIK-93	-0.04898	0.181278	219790
RTKN2	PLX4720	0.125489	0.000806	219790
RTKN2	Paclitaxel	0.039013	0.75204	219790

RTKN2	Parthenolide	0.030722	0.799665	219790
RTKN2	Pazopanib	0.061015	0.241848	219790
RTKN2	Phenformin	0.031928	0.419876	219790
RTKN2	Pyrimethamine	0.017285	0.904473	219790
RTKN2	QL-VIII-58	0.059477	0.436489	219790
RTKN2	QL-X-138	-0.06981	0.059265	219790
RTKN2	QL-XI-92	-0.00033	0.993801	219790
RTKN2	QL-XII-47	-0.00296	0.952247	219790
RTKN2	QL-XII-61	-0.01477	0.861759	219790
RTKN2	QS11	-0.01272	0.876079	219790
RTKN2	RDEA119	0.197763	8.14E-09	219790
RTKN2	RO-3306	0.092725	0.023105	219790
RTKN2	Rapamycin	-0.00423	0.983009	219790
RTKN2	Roscovitine	0.105662	0.442409	219790
RTKN2	Ruxolitinib	0.049947	0.316544	219790
RTKN2	S-Trityl-L-cysteine	0.050635	0.560564	219790
RTKN2	SB 216763	0.086134	0.068333	219790
RTKN2	SB 505124	-0.00451	0.969615	219790
RTKN2	SB52334	-0.09862	0.016214	219790
RTKN2	SB590885	0.134023	0.001424	219790
RTKN2	SGC0946	-0.01434	0.829934	219790
RTKN2	SL 0101-1	0.052826	0.481058	219790
RTKN2	SN-38	0.050743	0.250371	219790
RTKN2	SNX-2112	0.021487	0.591703	219790
RTKN2	STF-62247	0.019097	0.679018	219790
RTKN2	Salubrinal	0.03192	0.73936	219790
RTKN2	Saracatinib	0.018384	0.85312	219790
RTKN2	Shikonin	-0.00991	0.880631	219790
RTKN2	Sorafenib	0.142676	0.074278	219790
RTKN2	Sunitinib	0.086309	0.228042	219790
RTKN2	T0901317	-0.03646	0.39717	219790
RTKN2	TAE684	0.011171	0.945212	219790
RTKN2	TAK-715	-0.03213	0.407828	219790
RTKN2	TG101348	-0.00711	0.862588	219790
RTKN2	TGX221	0.083587	0.196052	219790
RTKN2	THZ-2-102-1	-0.01096	0.789384	219790
RTKN2	THZ-2-49	0.021225	0.599193	219790
RTKN2	TL-1-85	0.049471	0.187725	219790
RTKN2	TL-2-105	-0.00687	0.87301	219790
RTKN2	TPCA-1	-0.0344	0.360153	219790
RTKN2	TW 37	0.060287	0.17629	219790
RTKN2	Talazoparib	0.0166	0.750962	219790
RTKN2	Tamoxifen	0.029838	0.815286	219790
RTKN2	Temozolomide	0.0971	0.058264	219790
RTKN2	Temsirolimus	0.097602	0.023635	219790
RTKN2	Thapsigargin	-0.00673	0.942082	219790
RTKN2	Tipifarnib	0.039515	0.559779	219790
RTKN2	Tivozanib	0.054405	0.319104	219790
RTKN2	Trametinib	0.21027	1.73E-09	219790
RTKN2	Tubastatin A	-0.04776	0.196602	219790

RTKN2	UNC0638	0.000552	0.989546	219790
RTKN2	UNC1215	0.026023	0.644524	219790
RTKN2	VNLG/124	0.068475	0.090076	219790
RTKN2	VX-11e	0.056914	0.217743	219790
RTKN2	VX-680	-0.04892	0.623808	219790
RTKN2	VX-702	0.084214	0.490743	219790
RTKN2	Veliparib	0.081459	0.187487	219790
RTKN2	Vinblastine	0.113117	0.005143	219790
RTKN2	Vinorelbine	-0.00906	0.898911	219790
RTKN2	Vorinostat	-0.05419	0.150172	219790
RTKN2	WH-4-023	0.080753	0.286699	219790
RTKN2	WZ-1-84	0.009189	0.924791	219790
RTKN2	WZ3105	-0.10288	0.003644	219790
RTKN2	XAV939	0.007211	0.875894	219790
RTKN2	XL-184	0.017127	0.753328	219790
RTKN2	XMD11-85h	-0.00979	0.967877	219790
RTKN2	XMD13-2	-0.04155	0.270199	219790
RTKN2	XMD14-99	0.004649	0.918421	219790
RTKN2	XMD15-27	-0.01597	0.774608	219790
RTKN2	XMD8-85	0.126176	0.079776	219790
RTKN2	XMD8-92	0.020447	0.88257	219790
RTKN2	Y-39983	-0.00548	0.902056	219790
RTKN2	YK 4-279	0.080291	0.09785	219790
RTKN2	YM155	-0.05331	0.287525	219790
RTKN2	YM201636	-0.06337	0.089017	219790
RTKN2	Z-LLNle-CHO	0.173769	0.005505	219790
RTKN2	ZG-10	-0.06521	0.32236	219790
RTKN2	ZM-447439	-0.02754	0.593736	219790
RTKN2	ZSTK474	-0.06361	0.084695	219790
RTKN2	Zibotentan	0.005666	0.990687	219790
RTKN2	piperlongumine	0.097014	0.015352	219790
RTKN2	rTRAIL	0.027932	0.731938	219790
RTKN2	selumetinib	0.183813	1.04E-07	219790
SKA3	(5Z)-7-Oxozeaenol	0.142258	0.000122	221150
SKA3	17-AAG	0.193613	6.71E-08	221150
SKA3	5-Fluorouracil	-0.0973	0.007132	221150
SKA3	681640	0.014305	0.841517	221150
SKA3	A-443654	-0.00446	0.987925	221150
SKA3	A-770041	0.003415	0.976152	221150
SKA3	AC220	-0.06842	0.12327	221150
SKA3	AG-014699	0.017345	0.720076	221150
SKA3	AICAR	-0.10968	0.004338	221150
SKA3	AKT inhibitor VIII	0.078488	0.068622	221150
SKA3	AMG-706	0.026358	0.705945	221150
SKA3	AP-24534	-0.10318	0.008138	221150
SKA3	AR-42	-0.16671	1.71E-06	221150
SKA3	AS601245	0.07502	0.126769	221150
SKA3	AS605240	-0.05701	0.179156	221150
SKA3	AT-7519	-0.1576	5.81E-06	221150
SKA3	ATRA	-0.0549	0.255324	221150

SKA3	AUY922	0.015677	0.808003	221150
SKA3	AZ628	0.070325	0.348809	221150
SKA3	AZD6482	-0.0061	0.923029	221150
SKA3	AZD7762	-0.15199	5.68E-05	221150
SKA3	AZD8055	-0.11119	0.003763	221150
SKA3	Afatinib	0.117324	0.001044	221150
SKA3	Axitinib	-0.06789	0.164045	221150
SKA3	BAY 61-3606	-0.06149	0.133733	221150
SKA3	BEZ235	0.019853	0.724319	221150
SKA3	BHG712	-0.14877	2.21E-05	221150
SKA3	BI-2536	-0.15236	0.078709	221150
SKA3	BIRB 0796	0.047013	0.363733	221150
SKA3	BIX02189	-0.19126	3.78E-08	221150
SKA3	BMS-509744	-0.02012	0.85522	221150
SKA3	BMS-536924	-0.01876	0.824342	221150
SKA3	BMS-708163	0.089672	0.021878	221150
SKA3	BMS-754807	-0.00245	0.972274	221150
SKA3	BMS345541	-0.20246	4.04E-09	221150
SKA3	BX-795	-0.10319	0.012673	221150
SKA3	BX-912	-0.21036	6.85E-10	221150
SKA3	Belinostat	-0.13189	0.000261	221150
SKA3	Bexarotene	0.016159	0.869021	221150
SKA3	Bicalutamide	0.006994	0.90223	221150
SKA3	Bleomycin	0.046539	0.400795	221150
SKA3	Bleomycin (50 uM)	0.154596	1.1E-05	221150
SKA3	Bortezomib	0.095582	0.191651	221150
SKA3	Bosutinib	-0.04746	0.371649	221150
SKA3	Bryostatin 1	0.052019	0.309933	221150
SKA3	CAL-101	-0.12404	0.00061	221150
SKA3	CAY10603	-0.14631	2.89E-05	221150
SKA3	CCT007093	0.087751	0.032751	221150
SKA3	CCT018159	0.046102	0.37055	221150
SKA3	CEP-701	-0.10347	0.008013	221150
SKA3	CGP-082996	-0.0285	0.808834	221150
SKA3	CGP-60474	0.021195	0.84281	221150
SKA3	CH5424802	-0.05372	0.329059	221150
SKA3	CHIR-99021	0.020658	0.628743	221150
SKA3	CI-1040	0.064988	0.131268	221150
SKA3	CMK	0.037018	0.780703	221150
SKA3	CP466722	-0.18296	1.13E-07	221150
SKA3	CP724714	0.076881	0.104481	221150
SKA3	CUDC-101	-0.12605	0.000447	221150
SKA3	CX-5461	-0.14289	5.54E-05	221150
SKA3	Camptothecin	-0.09979	0.014071	221150
SKA3	Cetuximab	0.122419	0.00142	221150
SKA3	Cisplatin	-0.00385	0.954238	221150
SKA3	Crizotinib	-0.09059	0.430642	221150
SKA3	Cyclopamine	-0.03198	0.800202	221150
SKA3	Cytarabine	-0.05618	0.25308	221150
SKA3	DMOG	-0.07436	0.08319	221150

SKA3	Dabrafenib	0.091389	0.023711	221150
SKA3	Dasatinib	0.06597	0.3349	221150
SKA3	Docetaxel	0.116795	0.001596	221150
SKA3	Doxorubicin	0.01016	0.903794	221150
SKA3	EHT 1864	0.0132	0.882253	221150
SKA3	EKB-569	-0.11524	0.002271	221150
SKA3	EX-527	-0.00925	0.947844	221150
SKA3	Elesclomol	0.006282	0.907889	221150
SKA3	Embelin	0.010706	0.893669	221150
SKA3	Epothilone B	0.032021	0.557502	221150
SKA3	Erlotinib	0.145117	0.031472	221150
SKA3	Etoposide	-0.08063	0.065016	221150
SKA3	FH535	0.130245	0.001646	221150
SKA3	FK866	-0.12422	0.000477	221150
SKA3	FMK	-0.07827	0.179339	221150
SKA3	FR-180204	-0.07736	0.098167	221150
SKA3	FTI-277	0.118988	0.002508	221150
SKA3	Foretinib	-0.11584	0.002148	221150
SKA3	GDC0449	-0.01273	0.925056	221150
SKA3	GDC0941	-0.04782	0.38437	221150
SKA3	GNF-2	-0.06773	0.759599	221150
SKA3	GSK-650394	-0.0158	0.867426	221150
SKA3	GSK1070916	-0.2436	1.64E-12	221150
SKA3	GSK1904529A	0.049618	0.280527	221150
SKA3	GSK2126458	-0.13141	0.000267	221150
SKA3	GSK269962A	0.024529	0.681063	221150
SKA3	GSK429286A	-0.14058	0.000163	221150
SKA3	GSK690693	-0.16671	2.11E-06	221150
SKA3	GW 441756	0.026927	0.905152	221150
SKA3	GW-2580	-0.0302	0.94536	221150
SKA3	GW843682X	-0.15741	0.032444	221150
SKA3	Gefitinib	0.125596	0.001291	221150
SKA3	Gemcitabine	-0.06449	0.170906	221150
SKA3	Genentech Cpd 10	-0.18245	1.64E-07	221150
SKA3	HG-5-113-01	-0.03781	0.631864	221150
SKA3	HG-5-88-01	-0.01294	0.952514	221150
SKA3	HG-6-64-1	-0.00684	0.913168	221150
SKA3	I-BET-762	-0.20749	9.83E-10	221150
SKA3	IOX2	0.033165	0.587655	221150
SKA3	IPA-3	-0.08355	0.031298	221150
SKA3	Imatinib	-0.10601	0.32014	221150
SKA3	Ispinesib Mesylate	-0.1219	0.000636	221150
SKA3	JNJ-26854165	0.001264	0.988425	221150
SKA3	JNK Inhibitor VIII	0.07114	0.08966	221150
SKA3	JNK-9L	-0.01096	0.881815	221150
SKA3	JQ1	-0.03208	0.479817	221150
SKA3	JQ12	-0.02665	0.657931	221150
SKA3	JW-7-24-1	-0.16387	2.32E-06	221150
SKA3	JW-7-52-1	0.002204	0.989896	221150
SKA3	KIN001-055	0.026913	0.710531	221150

SKA3	KIN001-102	-0.17406	4.47E-07	221150
SKA3	KIN001-135	0.077181	0.428196	221150
SKA3	KIN001-236	-0.12331	0.0006	221150
SKA3	KIN001-244	-0.14544	4.15E-05	221150
SKA3	KIN001-260	-0.1303	0.000236	221150
SKA3	KIN001-266	-0.07869	0.077251	221150
SKA3	KIN001-270	-0.09596	0.011461	221150
SKA3	KU-55933	-0.04597	0.446507	221150
SKA3	LAQ824	-0.13266	0.000406	221150
SKA3	LFM-A13	0.049877	0.322973	221150
SKA3	LY317615	-0.10732	0.007321	221150
SKA3	Lapatinib	0.191727	0.001052	221150
SKA3	Lenalidomide	-0.03629	0.645897	221150
SKA3	Linifanib	-0.0449	0.386084	221150
SKA3	Lisitinib	-0.00781	0.932215	221150
SKA3	MG-132	0.086941	0.288674	221150
SKA3	MK-2206	-0.09598	0.039695	221150
SKA3	MLN4924	0.102567	0.04447	221150
SKA3	MP470	-0.03673	0.416292	221150
SKA3	MPS-1-IN-1	-0.10934	0.002816	221150
SKA3	MS-275	-0.15188	0.030158	221150
SKA3	Masitinib	-0.1454	5E-05	221150
SKA3	Methotrexate	-0.18606	1.95E-07	221150
SKA3	Midostaurin	0.028446	0.558762	221150
SKA3	Mitomycin C	-0.06388	0.186777	221150
SKA3	NG-25	-0.14792	2.29E-05	221150
SKA3	NPK76-II-72-1	-0.2649	2.52E-15	221150
SKA3	NSC-207895	-0.1129	0.004816	221150
SKA3	NSC-87877	0.068385	0.19447	221150
SKA3	NU-7441	-0.02279	0.793044	221150
SKA3	Navitoclax	-0.25309	6.88E-13	221150
SKA3	Nilotinib	-0.10063	0.023978	221150
SKA3	Nutlin-3a (-)	0.03799	0.451234	221150
SKA3	OSI-027	-0.14127	6.17E-05	221150
SKA3	OSI-930	-0.08969	0.017691	221150
SKA3	OSU-03012	-0.05388	0.266699	221150
SKA3	Obatoclax Mesylate	0.001574	0.981335	221150
SKA3	Olaparib	0.016308	0.744477	221150
SKA3	PAC-1	-0.16056	2.63E-05	221150
SKA3	PD-0325901	0.152371	5.15E-05	221150
SKA3	PD-0332991	-0.04113	0.470001	221150
SKA3	PD-173074	0.008645	0.974013	221150
SKA3	PF-4708671	0.046579	0.618355	221150
SKA3	PF-562271	0.009021	0.900504	221150
SKA3	PFI-1	0.028457	0.590093	221150
SKA3	PHA-665752	0.017972	0.918175	221150
SKA3	PHA-793887	-0.16445	1.72E-06	221150
SKA3	PI-103	-0.12966	0.00025	221150
SKA3	PIK-93	-0.19981	4.87E-09	221150
SKA3	PLX4720	0.096573	0.012822	221150

SKA3	Paclitaxel	0.00574	0.973869	221150
SKA3	Parthenolide	-0.02448	0.845281	221150
SKA3	Pazopanib	0.021442	0.73425	221150
SKA3	Phenformin	-0.09915	0.006733	221150
SKA3	Pyrimethamine	-0.09429	0.372382	221150
SKA3	QL-VIII-58	0.042364	0.607675	221150
SKA3	QL-X-138	-0.15666	9.1E-06	221150
SKA3	QL-XI-92	-0.17313	6.66E-07	221150
SKA3	QL-XII-47	-0.07264	0.063949	221150
SKA3	QL-XII-61	-0.1086	0.071837	221150
SKA3	QS11	-0.08457	0.120655	221150
SKA3	RDEA119	0.165068	1.93E-06	221150
SKA3	RO-3306	0.103763	0.010423	221150
SKA3	Rapamycin	0.00989	0.955754	221150
SKA3	Roscovitine	0.080253	0.602306	221150
SKA3	Ruxolitinib	-0.0429	0.403468	221150
SKA3	S-Trityl-L-cysteine	-0.05354	0.532807	221150
SKA3	SB 216763	0.065556	0.177368	221150
SKA3	SB 505124	0.029497	0.718937	221150
SKA3	SB52334	-0.03726	0.454786	221150
SKA3	SB590885	0.124288	0.003519	221150
SKA3	SGC0946	0.04909	0.346224	221150
SKA3	SL 0101-1	0.014408	0.878841	221150
SKA3	SN-38	-0.02045	0.686328	221150
SKA3	SNX-2112	-0.13986	7.57E-05	221150
SKA3	STF-62247	-0.10435	0.006911	221150
SKA3	Salubrinal	-0.05949	0.490235	221150
SKA3	Saracatinib	-0.0182	0.854712	221150
SKA3	Shikonin	-0.02259	0.709216	221150
SKA3	Sorafenib	-0.02756	0.831812	221150
SKA3	Sunitinib	-0.12137	0.070755	221150
SKA3	T0901317	-0.09479	0.015005	221150
SKA3	TAE684	-0.03192	0.813485	221150
SKA3	TAK-715	-0.13815	9.04E-05	221150
SKA3	TG101348	-0.20034	5.18E-09	221150
SKA3	TGX221	0.11266	0.071664	221150
SKA3	THZ-2-102-1	-0.17173	9.17E-07	221150
SKA3	THZ-2-49	-0.10462	0.003945	221150
SKA3	TL-1-85	-0.16366	2.62E-06	221150
SKA3	TL-2-105	-0.15023	2.47E-05	221150
SKA3	TPCA-1	-0.1961	9.64E-09	221150
SKA3	TW 37	0.013504	0.810497	221150
SKA3	Talazoparib	-0.01136	0.833281	221150
SKA3	Tamoxifen	0.026983	0.839806	221150
SKA3	Temozolomide	0.035894	0.630263	221150
SKA3	Temsirolimus	-0.01051	0.86395	221150
SKA3	Thapsigargin	0.005292	0.955679	221150
SKA3	Tipifarnib	0.01748	0.831656	221150
SKA3	Tivozanib	-0.0876	0.067565	221150
SKA3	Trametinib	0.223793	1.29E-10	221150

SKA3	Tubastatin A	-0.14926	1.87E-05	221150
SKA3	UNC0638	-0.1498	1.15E-05	221150
SKA3	UNC1215	0.071373	0.127795	221150
SKA3	VNLG/124	-0.08742	0.026365	221150
SKA3	VX-11e	0.02232	0.68298	221150
SKA3	VX-680	-0.12236	0.133422	221150
SKA3	VX-702	-0.02713	0.912545	221150
SKA3	Veliparib	-0.01469	0.859417	221150
SKA3	Vinblastine	-0.04661	0.301866	221150
SKA3	Vinorelbine	-0.05104	0.34268	221150
SKA3	Vorinostat	-0.17465	1.09E-06	221150
SKA3	WH-4-023	0.037211	0.666297	221150
SKA3	WZ-1-84	0.016808	0.858344	221150
SKA3	WZ3105	-0.17611	3.3E-07	221150
SKA3	XAV939	0.05971	0.128956	221150
SKA3	XL-184	-0.07064	0.107604	221150
SKA3	XMD11-85h	0.013745	0.953656	221150
SKA3	XMD13-2	-0.16793	1.32E-06	221150
SKA3	XMD14-99	-0.08556	0.021718	221150
SKA3	XMD15-27	-0.06342	0.158951	221150
SKA3	XMD8-85	-0.05368	0.531633	221150
SKA3	XMD8-92	-0.03263	0.787803	221150
SKA3	Y-39983	-0.17185	1.17E-06	221150
SKA3	YK 4-279	0.058669	0.253198	221150
SKA3	YM155	-0.03881	0.469946	221150
SKA3	YM201636	-0.12829	0.000317	221150
SKA3	Z-LLNle-CHO	0.050745	0.522122	221150
SKA3	ZG-10	-0.09882	0.107721	221150
SKA3	ZM-447439	-0.14213	0.000461	221150
SKA3	ZSTK474	-0.1444	4.2E-05	221150
SKA3	Zibotentan	-0.01784	0.958851	221150
SKA3	piperlongumine	0.075375	0.068585	221150
SKA3	rTRAIL	0.024106	0.773622	221150
SKA3	selumetinib	0.166092	1.79E-06	221150
TTK	(5Z)-7-Oxozeaenol	0.169082	3.48E-06	7272
TTK	17-AAG	0.138364	0.000146	7272
TTK	5-Fluorouracil	-0.00953	0.816637	7272
TTK	681640	-0.00551	0.940837	7272
TTK	A-443654	-0.02592	0.911146	7272
TTK	A-770041	-0.01844	0.856398	7272
TTK	AC220	0.000133	0.998189	7272
TTK	AG-014699	0.039723	0.368808	7272
TTK	AICAR	-0.05913	0.143354	7272
TTK	AKT inhibitor VIII	0.049369	0.278975	7272
TTK	AMG-706	0.040668	0.511837	7272
TTK	AP-24534	-0.04176	0.33144	7272
TTK	AR-42	-0.09996	0.005077	7272
TTK	AS601245	0.083463	0.081365	7272
TTK	AS605240	0.027713	0.556695	7272
TTK	AT-7519	-0.13378	0.000135	7272

TTK	ATRA	-0.02198	0.695722	7272
TTK	AUY922	0.034994	0.531292	7272
TTK	AZ628	0.183967	0.003474	7272
TTK	AZD6482	0.046054	0.322983	7272
TTK	AZD7762	-0.09628	0.013768	7272
TTK	AZD8055	-0.0436	0.288783	7272
TTK	Afatinib	0.072302	0.050523	7272
TTK	Axitinib	-0.06643	0.175237	7272
TTK	BAY 61-3606	0.039836	0.353746	7272
TTK	BEZ235	0.021961	0.6924	7272
TTK	BHG712	-0.04477	0.235296	7272
TTK	BI-2536	-0.11429	0.204518	7272
TTK	BIRB 0796	0.040431	0.445694	7272
TTK	BIX02189	-0.05182	0.169111	7272
TTK	BMS-509744	0.079689	0.369846	7272
TTK	BMS-536924	0.064963	0.263096	7272
TTK	BMS-708163	0.077646	0.048557	7272
TTK	BMS-754807	0.083521	0.089318	7272
TTK	BMS345541	-0.11172	0.001613	7272
TTK	BX-795	-0.06616	0.133805	7272
TTK	BX-912	-0.12552	0.000334	7272
TTK	Belinostat	-0.08691	0.018726	7272
TTK	Bexarotene	0.032571	0.68393	7272
TTK	Bicalutamide	0.029989	0.532171	7272
TTK	Bleomycin	0.054833	0.305926	7272
TTK	Bleomycin (50 uM)	0.071039	0.05165	7272
TTK	Bortezomib	0.058879	0.471259	7272
TTK	Bosutinib	-0.03572	0.529098	7272
TTK	Bryostatin 1	0.071528	0.139064	7272
TTK	CAL-101	-0.06205	0.103389	7272
TTK	CAY10603	-0.10962	0.002006	7272
TTK	CCT007093	0.065593	0.122166	7272
TTK	CCT018159	-0.01186	0.85735	7272
TTK	CEP-701	-0.02276	0.614409	7272
TTK	CGP-082996	0.007515	0.956265	7272
TTK	CGP-60474	0.025279	0.80702	7272
TTK	CH5424802	-0.00047	0.996271	7272
TTK	CHIR-99021	0.060456	0.115769	7272
TTK	CI-1040	0.158272	4.54E-05	7272
TTK	CMK	0.041546	0.745058	7272
TTK	CP466722	-0.0944	0.008183	7272
TTK	CP724714	0.028641	0.6308	7272
TTK	CUDC-101	-0.10498	0.003784	7272
TTK	CX-5461	-0.09258	0.010826	7272
TTK	Camptothecin	-0.07706	0.067317	7272
TTK	Cetuximab	0.04028	0.341541	7272
TTK	Cisplatin	-0.01378	0.819376	7272
TTK	Crizotinib	-0.03784	0.816487	7272
TTK	Cyclopamine	0.004609	0.97485	7272
TTK	Cytarabine	-0.01599	0.790377	7272

TTK	DMOG	-0.03446	0.476418	7272
TTK	Dabrafenib	0.182041	1.06E-06	7272
TTK	Dasatinib	0.036603	0.616957	7272
TTK	Docetaxel	-0.0013	0.97615	7272
TTK	Doxorubicin	0.014601	0.855491	7272
TTK	EHT 1864	0.052133	0.402449	7272
TTK	EKB-569	-0.05266	0.195473	7272
TTK	EX-527	0.011708	0.933014	7272
TTK	Elesclomol	-0.03558	0.449312	7272
TTK	Embelin	0.026301	0.696501	7272
TTK	Epothilone B	0.00313	0.96103	7272
TTK	Erlotinib	0.149327	0.026172	7272
TTK	Etoposide	-0.09163	0.032509	7272
TTK	FH535	0.084985	0.051921	7272
TTK	FK866	-0.10661	0.002947	7272
TTK	FMK	0.032009	0.661564	7272
TTK	FR-180204	0.021866	0.717961	7272
TTK	FTI-277	0.138353	0.000384	7272
TTK	Foretinib	-0.05601	0.175301	7272
TTK	GDC0449	0.027389	0.810183	7272
TTK	GDC0941	-0.02547	0.691522	7272
TTK	GNF-2	-0.04358	0.865101	7272
TTK	GSK-650394	-0.00605	0.952702	7272
TTK	GSK1070916	-0.12627	0.000428	7272
TTK	GSK1904529A	0.049363	0.283406	7272
TTK	GSK2126458	-0.07004	0.062483	7272
TTK	GSK269962A	0.057244	0.240219	7272
TTK	GSK429286A	-0.07741	0.052107	7272
TTK	GSK690693	-0.12589	0.000428	7272
TTK	GW 441756	0.013507	0.970373	7272
TTK	GW-2580	0.022261	0.967411	7272
TTK	GW843682X	-0.13207	0.079952	7272
TTK	Gefitinib	0.07379	0.071413	7272
TTK	Gemcitabine	-0.0573	0.232823	7272
TTK	Genentech Cpd 10	-0.11672	0.001127	7272
TTK	HG-5-113-01	-0.00873	0.926182	7272
TTK	HG-5-88-01	0.052736	0.718896	7272
TTK	HG-6-64-1	0.045766	0.347587	7272
TTK	I-BET-762	-0.08631	0.014185	7272
TTK	IOX2	-0.0087	0.910579	7272
TTK	IPA-3	-0.04742	0.243884	7272
TTK	Imatinib	-0.05793	0.709339	7272
TTK	Ispinesib Mesylate	-0.07888	0.031304	7272
TTK	JNJ-26854165	0.036415	0.494792	7272
TTK	JNK Inhibitor VIII	0.032092	0.479321	7272
TTK	JNK-9L	0.043428	0.447253	7272
TTK	JQ1	0.032697	0.470481	7272
TTK	JQ12	0.022265	0.718275	7272
TTK	JW-7-24-1	-0.08568	0.016757	7272
TTK	JW-7-52-1	0.024021	0.845787	7272

TTK	KIN001-055	0.02626	0.719057	7272
TTK	KIN001-102	-0.10836	0.002086	7272
TTK	KIN001-135	0.077549	0.425168	7272
TTK	KIN001-236	-0.03382	0.389366	7272
TTK	KIN001-244	-0.07945	0.030622	7272
TTK	KIN001-260	-0.05652	0.127945	7272
TTK	KIN001-266	-0.01019	0.858811	7272
TTK	KIN001-270	-0.08928	0.019359	7272
TTK	KU-55933	-0.05139	0.378518	7272
TTK	LAQ824	-0.05876	0.142948	7272
TTK	LFM-A13	0.07713	0.102259	7272
TTK	LY317615	-0.00963	0.85886	7272
TTK	Lapatinib	0.155255	0.009615	7272
TTK	Lenalidomide	-0.00431	0.971792	7272
TTK	Linifanib	0.022476	0.710602	7272
TTK	Lisitinib	0.069747	0.213702	7272
TTK	MG-132	0.060921	0.499525	7272
TTK	MK-2206	-0.09208	0.049664	7272
TTK	MLN4924	0.04474	0.438855	7272
TTK	MP470	-0.04509	0.305879	7272
TTK	MPS-1-IN-1	-0.01579	0.710364	7272
TTK	MS-275	-0.09802	0.195278	7272
TTK	Masitinib	-0.05284	0.170275	7272
TTK	Methotrexate	-0.05327	0.163707	7272
TTK	Midostaurin	0.05399	0.224099	7272
TTK	Mitomycin C	0.002597	0.969984	7272
TTK	NG-25	-0.09045	0.011664	7272
TTK	NPK76-II-72-1	-0.18523	5.61E-08	7272
TTK	NSC-207895	-0.1586	5.22E-05	7272
TTK	NSC-87877	0.084819	0.099104	7272
TTK	NU-7441	-0.01277	0.900815	7272
TTK	Navitoclax	-0.17565	1.03E-06	7272
TTK	Nilotinib	-0.08847	0.052486	7272
TTK	Nutlin-3a (-)	0.210104	9.83E-08	7272
TTK	OSI-027	-0.09237	0.010243	7272
TTK	OSI-930	-0.02386	0.575719	7272
TTK	OSU-03012	-0.00542	0.936758	7272
TTK	Obatoclax Mesylate	0.027384	0.597076	7272
TTK	Olaparib	0.00319	0.952783	7272
TTK	PAC-1	-0.02831	0.541813	7272
TTK	PD-0325901	0.1918	2.33E-07	7272
TTK	PD-0332991	0.064894	0.207352	7272
TTK	PD-173074	0.000783	0.996072	7272
TTK	PF-4708671	0.086719	0.285271	7272
TTK	PF-562271	0.03292	0.585408	7272
TTK	PFI-1	0.067921	0.134236	7272
TTK	PHA-665752	0.091231	0.494896	7272
TTK	PHA-793887	-0.09344	0.007977	7272
TTK	PI-103	-0.09616	0.007417	7272
TTK	PIK-93	-0.11723	0.00081	7272

TTK	PLX4720	0.178907	5.88E-07	7272
TTK	Paclitaxel	-0.01513	0.922295	7272
TTK	Parthenolide	-0.01954	0.87983	7272
TTK	Pazopanib	0.049059	0.368079	7272
TTK	Phenformin	-0.02659	0.507694	7272
TTK	Pyrimethamine	-0.03546	0.78008	7272
TTK	QL-VIII-58	-0.00275	0.981539	7272
TTK	QL-X-138	-0.11826	0.000978	7272
TTK	QL-XI-92	-0.08977	0.012655	7272
TTK	QL-XII-47	-0.06438	0.104674	7272
TTK	QL-XII-61	-0.04661	0.510489	7272
TTK	QS11	-0.04367	0.486864	7272
TTK	RDEA119	0.226839	2.68E-11	7272
TTK	RO-3306	0.020167	0.65987	7272
TTK	Rapamycin	-0.02691	0.85913	7272
TTK	Roscovitine	0.082216	0.592831	7272
TTK	Ruxolitinib	-0.04154	0.423039	7272
TTK	S-Trityl-L-cysteine	-0.03672	0.691991	7272
TTK	SB 216763	0.048789	0.330723	7272
TTK	SB 505124	0.058851	0.391247	7272
TTK	SB52334	0.04762	0.315576	7272
TTK	SB590885	0.188014	1.66E-06	7272
TTK	SGC0946	0.064679	0.185542	7272
TTK	SL 0101-1	0.080436	0.271668	7272
TTK	SN-38	0.001589	0.978926	7272
TTK	SNX-2112	-0.07537	0.039895	7272
TTK	STF-62247	-0.01976	0.667043	7272
TTK	Salubrinal	0.032385	0.735897	7272
TTK	Saracatinib	-0.03759	0.678611	7272
TTK	Shikonin	-0.02908	0.61683	7272
TTK	Sorafenib	0.067454	0.519867	7272
TTK	Sunitinib	0.012154	0.902263	7272
TTK	T0901317	-0.04731	0.258657	7272
TTK	TAE684	0.035403	0.791265	7272
TTK	TAK-715	-0.08107	0.025536	7272
TTK	TG101348	-0.08121	0.023873	7272
TTK	TGX221	0.161307	0.007302	7272
TTK	THZ-2-102-1	-0.12266	0.000557	7272
TTK	THZ-2-49	-0.02776	0.482994	7272
TTK	TL-1-85	-0.09167	0.010954	7272
TTK	TL-2-105	-0.0986	0.006979	7272
TTK	TPCA-1	-0.09386	0.008027	7272
TTK	TW 37	-0.0716	0.096247	7272
TTK	Talazoparib	0.024673	0.620682	7272
TTK	Tamoxifen	0.022898	0.868068	7272
TTK	Temozolomide	0.012177	0.898805	7272
TTK	Temsirolimus	-0.00685	0.913993	7272
TTK	Thapsigargin	0.011203	0.900962	7272
TTK	Tipifarnib	0.036302	0.600687	7272
TTK	Tivozanib	-0.03613	0.551817	7272

TTK	Trametinib	0.257337	9.55E-14	7272
TTK	Tubastatin A	-0.07083	0.050098	7272
TTK	UNC0638	-0.05118	0.158031	7272
TTK	UNC1215	0.101221	0.023617	7272
TTK	VNLG/124	-0.00868	0.863178	7272
TTK	VX-11e	0.1183	0.003998	7272
TTK	VX-680	-0.06125	0.517218	7272
TTK	VX-702	0.017467	0.945864	7272
TTK	Veliparib	0.028824	0.692956	7272
TTK	Vinblastine	-0.07424	0.080356	7272
TTK	Vinorelbine	-0.06397	0.21314	7272
TTK	Vorinostat	-0.14792	4.11E-05	7272
TTK	WH-4-023	0.038259	0.656009	7272
TTK	WZ-1-84	0.072294	0.353153	7272
TTK	WZ3105	-0.11869	0.000733	7272
TTK	XAV939	0.015486	0.724462	7272
TTK	XL-184	0.000858	0.989499	7272
TTK	XMD11-85h	0.085165	0.546089	7272
TTK	XMD13-2	-0.07388	0.041454	7272
TTK	XMD14-99	0.040301	0.312249	7272
TTK	XMD15-27	0.016033	0.773673	7272
TTK	XMD8-85	0.088221	0.255366	7272
TTK	XMD8-92	0.007222	0.963538	7272
TTK	Y-39983	-0.08004	0.031379	7272
TTK	YK 4-279	0.023145	0.709015	7272
TTK	YM155	-0.09683	0.030261	7272
TTK	YM201636	-0.06611	0.075171	7272
TTK	Z-LLNle-CHO	0.053811	0.492274	7272
TTK	ZG-10	-0.06314	0.341994	7272
TTK	ZM-447439	-0.06899	0.121597	7272
TTK	ZSTK474	-0.09049	0.012259	7272
TTK	Zibotentan	-0.01527	0.968639	7272
TTK	piperlongumine	0.027071	0.572839	7272
TTK	rTRAIL	0.073602	0.246262	7272
TTK	selumetinib	0.242762	8.82E-13	7272
UBE2T	(5Z)-7-Oxozeaenol	0.184954	3.07E-07	29089
UBE2T	17-AAG	0.190666	1.08E-07	29089
UBE2T	5-Fluorouracil	-0.03924	0.30572	29089
UBE2T	681640	0.069463	0.223191	29089
UBE2T	A-443654	0.027911	0.9026	29089
UBE2T	A-770041	0.158449	0.019396	29089
UBE2T	AC220	-0.01765	0.760436	29089
UBE2T	AG-014699	0.070102	0.092287	29089
UBE2T	AICAR	-0.06626	0.098228	29089
UBE2T	AKT inhibitor VIII	0.1318	0.001284	29089
UBE2T	AMG-706	0.079336	0.127423	29089
UBE2T	AP-24534	0.058942	0.154751	29089
UBE2T	AR-42	-0.11868	0.000801	29089
UBE2T	AS601245	0.155091	0.000311	29089
UBE2T	AS605240	-0.01069	0.837134	29089

UBE2T	AT-7519	-0.06188	0.089438	29089
UBE2T	ATRA	-0.00773	0.90165	29089
UBE2T	AUY922	0.144518	0.000597	29089
UBE2T	AZ628	0.171043	0.007143	29089
UBE2T	AZD6482	0.159759	3.04E-05	29089
UBE2T	AZD7762	-0.02638	0.549987	29089
UBE2T	AZD8055	0.021443	0.622075	29089
UBE2T	Afatinib	0.100099	0.005658	29089
UBE2T	Axitinib	-0.03424	0.542823	29089
UBE2T	BAY 61-3606	0.041336	0.333492	29089
UBE2T	BEZ235	0.160556	7.71E-05	29089
UBE2T	BHG712	0.012106	0.765604	29089
UBE2T	BI-2536	-0.01125	0.938442	29089
UBE2T	BIRB 0796	0.091448	0.052314	29089
UBE2T	BIX02189	0.008288	0.845515	29089
UBE2T	BMS-509744	0.115139	0.174182	29089
UBE2T	BMS-536924	0.027979	0.719366	29089
UBE2T	BMS-708163	0.126843	0.000936	29089
UBE2T	BMS-754807	0.052186	0.331266	29089
UBE2T	BMS345541	-0.09142	0.010698	29089
UBE2T	BX-795	0.006309	0.917726	29089
UBE2T	BX-912	-0.09161	0.009918	29089
UBE2T	Belinostat	-0.09246	0.012105	29089
UBE2T	Bexarotene	0.068085	0.264993	29089
UBE2T	Bicalutamide	0.058671	0.180787	29089
UBE2T	Bleomycin	0.129179	0.004013	29089
UBE2T	Bleomycin (50 uM)	0.188099	6.77E-08	29089
UBE2T	Bortezomib	0.155123	0.018548	29089
UBE2T	Bosutinib	0.083928	0.070831	29089
UBE2T	Bryostatin 1	0.102971	0.024636	29089
UBE2T	CAL-101	-3.8E-05	0.999408	29089
UBE2T	CAY10603	-0.09637	0.006924	29089
UBE2T	CCT007093	0.112524	0.005087	29089
UBE2T	CCT018159	0.062781	0.196388	29089
UBE2T	CEP-701	0.022022	0.627328	29089
UBE2T	CGP-082996	0.061776	0.519468	29089
UBE2T	CGP-60474	0.145151	0.039154	29089
UBE2T	CH5424802	-0.03889	0.519953	29089
UBE2T	CHIR-99021	0.158624	8.21E-06	29089
UBE2T	CI-1040	0.154364	7.17E-05	29089
UBE2T	CMK	0.074936	0.493018	29089
UBE2T	CP466722	-0.0804	0.025505	29089
UBE2T	CP724714	0.049962	0.341803	29089
UBE2T	CUDC-101	-0.09709	0.007659	29089
UBE2T	CX-5461	-0.10619	0.003222	29089
UBE2T	Camptothecin	-0.03304	0.486939	29089
UBE2T	Cetuximab	0.102512	0.008534	29089
UBE2T	Cisplatin	0.029244	0.584478	29089
UBE2T	Crizotinib	0.003945	0.988655	29089
UBE2T	Cyclopamine	0.111186	0.212135	29089

UBE2T	Cytarabine	0.026922	0.633728	29089
UBE2T	DMOG	0.025168	0.619544	29089
UBE2T	Dabrafenib	0.152158	6.46E-05	29089
UBE2T	Dasatinib	0.209952	0.000417	29089
UBE2T	Docetaxel	0.170059	3.08E-06	29089
UBE2T	Doxorubicin	0.071412	0.201053	29089
UBE2T	EHT 1864	0.037675	0.582995	29089
UBE2T	EKB-569	0.00402	0.934703	29089
UBE2T	EX-527	0.022556	0.856192	29089
UBE2T	Elesclomol	0.050668	0.259899	29089
UBE2T	Embelin	0.062895	0.242423	29089
UBE2T	Epothilone B	0.099524	0.029347	29089
UBE2T	Erlotinib	0.103929	0.143568	29089
UBE2T	Etoposide	-0.05067	0.283044	29089
UBE2T	FH535	0.080857	0.066738	29089
UBE2T	FK866	-0.1618	4.05E-06	29089
UBE2T	FMK	0.054055	0.403169	29089
UBE2T	FR-180204	0.039661	0.459748	29089
UBE2T	FTI-277	0.177561	3.66E-06	29089
UBE2T	Foretinib	-0.01448	0.767535	29089
UBE2T	GDC0449	0.075026	0.34838	29089
UBE2T	GDC0941	0.064504	0.205071	29089
UBE2T	GNF-2	-0.00259	0.995323	29089
UBE2T	GSK-650394	0.034857	0.649914	29089
UBE2T	GSK1070916	-0.15203	1.8E-05	29089
UBE2T	GSK1904529A	0.078426	0.072903	29089
UBE2T	GSK2126458	-0.04056	0.301928	29089
UBE2T	GSK269962A	0.084478	0.056025	29089
UBE2T	GSK429286A	-0.03263	0.463965	29089
UBE2T	GSK690693	-0.10029	0.005691	29089
UBE2T	GW 441756	-0.00618	0.990735	29089
UBE2T	GW-2580	0.032369	0.938845	29089
UBE2T	GW843682X	-0.03565	0.723572	29089
UBE2T	Gefitinib	0.107752	0.006444	29089
UBE2T	Gemcitabine	-0.00097	0.98903	29089
UBE2T	Genentech Cpd 10	-0.0995	0.006024	29089
UBE2T	HG-5-113-01	0.001033	0.992354	29089
UBE2T	HG-5-88-01	0.078127	0.513772	29089
UBE2T	HG-6-64-1	0.18243	2.7E-06	29089
UBE2T	I-BET-762	-0.08685	0.01356	29089
UBE2T	IOX2	0.051752	0.358021	29089
UBE2T	IPA-3	-0.00805	0.86412	29089
UBE2T	Imatinib	-0.01827	0.923845	29089
UBE2T	Ispinesib Mesylate	-0.04229	0.269898	29089
UBE2T	JNJ-26854165	0.121972	0.004609	29089
UBE2T	JNK Inhibitor VIII	0.065035	0.123975	29089
UBE2T	JNK-9L	0.095201	0.044988	29089
UBE2T	JQ1	0.064659	0.120649	29089
UBE2T	JQ12	0.020004	0.749947	29089
UBE2T	JW-7-24-1	-0.062	0.089175	29089

UBE2T	JW-7-52-1	0.149043	0.052057	29089
UBE2T	KIN001-055	0.039769	0.534504	29089
UBE2T	KIN001-102	-0.10111	0.004201	29089
UBE2T	KIN001-135	0.056893	0.591865	29089
UBE2T	KIN001-236	-0.01768	0.668322	29089
UBE2T	KIN001-244	-0.03588	0.356013	29089
UBE2T	KIN001-260	-0.01494	0.715186	29089
UBE2T	KIN001-266	-0.01747	0.749117	29089
UBE2T	KIN001-270	-0.05034	0.210504	29089
UBE2T	KU-55933	0.022518	0.757193	29089
UBE2T	LAQ824	-0.05039	0.215261	29089
UBE2T	LFM-A13	0.096036	0.036251	29089
UBE2T	LY317615	-0.01678	0.739873	29089
UBE2T	Lapatinib	0.138475	0.022405	29089
UBE2T	Lenalidomide	-0.02596	0.770724	29089
UBE2T	Linifanib	0.032449	0.55905	29089
UBE2T	Lisitinib	0.030149	0.671054	29089
UBE2T	MG-132	0.120241	0.109912	29089
UBE2T	MK-2206	0.003581	0.960673	29089
UBE2T	MLN4924	0.12044	0.016522	29089
UBE2T	MP470	-0.11469	0.005757	29089
UBE2T	MPS-1-IN-1	-0.07049	0.0618	29089
UBE2T	MS-275	-0.12363	0.087923	29089
UBE2T	Masitinib	-0.02452	0.552197	29089
UBE2T	Methotrexate	-0.07828	0.035968	29089
UBE2T	Midostaurin	0.130738	0.001124	29089
UBE2T	Mitomycin C	-0.03543	0.51489	29089
UBE2T	NG-25	-0.02509	0.521199	29089
UBE2T	NPK76-II-72-1	-0.16765	1.01E-06	29089
UBE2T	NSC-207895	-0.10951	0.006449	29089
UBE2T	NSC-87877	0.087996	0.085426	29089
UBE2T	NU-7441	0.078088	0.195891	29089
UBE2T	Navitoclax	-0.20975	3.96E-09	29089
UBE2T	Nilotinib	-0.02247	0.704667	29089
UBE2T	Nutlin-3a (-)	0.108918	0.010113	29089
UBE2T	OSI-027	-0.0621	0.0916	29089
UBE2T	OSI-930	0.001992	0.967544	29089
UBE2T	OSU-03012	0.064969	0.167228	29089
UBE2T	Obatoclax Mesylate	0.060245	0.190199	29089
UBE2T	Olaparib	0.064511	0.131941	29089
UBE2T	PAC-1	-0.02925	0.526488	29089
UBE2T	PD-0325901	0.211559	9.65E-09	29089
UBE2T	PD-0332991	0.110357	0.016059	29089
UBE2T	PD-173074	0.011712	0.957247	29089
UBE2T	PF-4708671	0.084778	0.298798	29089
UBE2T	PF-562271	0.107929	0.025474	29089
UBE2T	PFI-1	0.116108	0.005854	29089
UBE2T	PHA-665752	0.08294	0.547454	29089
UBE2T	PHA-793887	-0.08215	0.020401	29089
UBE2T	PI-103	-0.08855	0.014144	29089

UBE2T	PIK-93	-0.07469	0.036928	29089
UBE2T	PLX4720	0.106731	0.005282	29089
UBE2T	Paclitaxel	0.097309	0.298357	29089
UBE2T	Parthenolide	0.012144	0.928485	29089
UBE2T	Pazopanib	0.095648	0.044414	29089
UBE2T	Phenformin	-0.05381	0.15702	29089
UBE2T	Pyrimethamine	0.01505	0.91801	29089
UBE2T	QL-VIII-58	0.154679	0.010552	29089
UBE2T	QL-X-138	-0.08915	0.014484	29089
UBE2T	QL-XI-92	-0.0749	0.039545	29089
UBE2T	QL-XII-47	-0.04573	0.265808	29089
UBE2T	QL-XII-61	-0.03222	0.672069	29089
UBE2T	QS11	-0.02833	0.680005	29089
UBE2T	RDEA119	0.24305	7.51E-13	29089
UBE2T	RO-3306	0.094027	0.021094	29089
UBE2T	Rapamycin	0.06797	0.554192	29089
UBE2T	Roscovitine	0.151732	0.209241	29089
UBE2T	Ruxolitinib	-0.02205	0.710039	29089
UBE2T	S-Trityl-L-cysteine	0.01525	0.883214	29089
UBE2T	SB 216763	0.081764	0.084784	29089
UBE2T	SB 505124	0.068884	0.292188	29089
UBE2T	SB52334	-0.06364	0.151282	29089
UBE2T	SB590885	0.135171	0.001258	29089
UBE2T	SGC0946	0.050452	0.330255	29089
UBE2T	SL 0101-1	0.083593	0.254436	29089
UBE2T	SN-38	0.050784	0.249895	29089
UBE2T	SNX-2112	-0.03024	0.43982	29089
UBE2T	STF-62247	0.004066	0.936269	29089
UBE2T	Salubrinal	0.05135	0.563842	29089
UBE2T	Saracatinib	0.094458	0.234423	29089
UBE2T	Shikonin	0.035758	0.523915	29089
UBE2T	Sorafenib	0.056213	0.616225	29089
UBE2T	Sunitinib	0.06106	0.42962	29089
UBE2T	T0901317	-0.01922	0.676524	29089
UBE2T	TAE684	0.031859	0.814106	29089
UBE2T	TAK-715	-0.05612	0.130718	29089
UBE2T	TG101348	-0.05995	0.101585	29089
UBE2T	TGX221	0.265082	2.9E-06	29089
UBE2T	THZ-2-102-1	-0.09932	0.005689	29089
UBE2T	THZ-2-49	-0.01401	0.736002	29089
UBE2T	TL-1-85	-0.01387	0.734308	29089
UBE2T	TL-2-105	-0.08297	0.024738	29089
UBE2T	TPCA-1	-0.07437	0.037851	29089
UBE2T	TW 37	-0.00519	0.93225	29089
UBE2T	Talazoparib	0.006941	0.900492	29089
UBE2T	Tamoxifen	0.089019	0.285036	29089
UBE2T	Temozolomide	0.033806	0.652801	29089
UBE2T	Temsirolimus	0.100333	0.019435	29089
UBE2T	Thapsigargin	0.036423	0.591594	29089
UBE2T	Tipifarnib	0.086512	0.106405	29089

UBE2T	Tivozanib	0.007995	0.917713	29089
UBE2T	Trametinib	0.285657	0	29089
UBE2T	Tubastatin A	-0.06964	0.054199	29089
UBE2T	UNC0638	-0.05815	0.106318	29089
UBE2T	UNC1215	0.092811	0.040334	29089
UBE2T	VNLG/124	0.017306	0.719141	29089
UBE2T	VX-11e	0.104756	0.012328	29089
UBE2T	VX-680	-0.02103	0.859654	29089
UBE2T	VX-702	0.023455	0.926443	29089
UBE2T	Veliparib	0.04449	0.516011	29089
UBE2T	Vinblastine	0.0615	0.157398	29089
UBE2T	Vinorelbine	0.038	0.50577	29089
UBE2T	Vorinostat	-0.1725	1.49E-06	29089
UBE2T	WH-4-023	0.215685	0.000815	29089
UBE2T	WZ-1-84	0.169103	0.011247	29089
UBE2T	WZ3105	-0.11028	0.001766	29089
UBE2T	XAV939	0.090688	0.017608	29089
UBE2T	XL-184	0.054888	0.231433	29089
UBE2T	XMD11-85h	0.088335	0.522152	29089
UBE2T	XMD13-2	-0.05215	0.159123	29089
UBE2T	XMD14-99	-0.02341	0.575831	29089
UBE2T	XMD15-27	-0.01106	0.848861	29089
UBE2T	XMD8-85	0.148731	0.0317	29089
UBE2T	XMD8-92	0.114053	0.177839	29089
UBE2T	Y-39983	-0.0378	0.340141	29089
UBE2T	YK 4-279	0.123664	0.006237	29089
UBE2T	YM155	-0.03679	0.498203	29089
UBE2T	YM201636	-0.0783	0.033041	29089
UBE2T	Z-LLNle-CHO	0.168045	0.007663	29089
UBE2T	ZG-10	0.005039	0.956248	29089
UBE2T	ZM-447439	-0.04351	0.363768	29089
UBE2T	ZSTK474	-0.05463	0.142552	29089
UBE2T	Zibotentan	0.060052	0.725079	29089
UBE2T	piperlongumine	0.126455	0.001117	29089
UBE2T	rTRAIL	0.051828	0.463516	29089
UBE2T	selumetinib	0.247458	2.93E-13	29089

Symbol:Name of gene
Drug:Name of drug
Cor:Correlation coefficient
Fdr:False Discovery Rate
Entrez:Gene ID in NCBI database
