# Supplementary material for: Identification of the molecular subtypes and construction of risk models in neuroblastoma
Source: Sci Rep. 2023 Jul 21;13:11790. doi: 10.1038/s41598-023-35401-3 (PMC10362029; doi:10.1038/s41598-023-35401-3)
Supplement: Supplementary file 2 — Supplementary Information 2. [file 41598_2023_35401_MOESM2_ESM.pdf]

Scientific reports

Supplementary Materials for
Identification of the molecular subtypes and Construction of risk models in neuroblastoma

Enyang He, Bowen Shi, Ziyu Liu, Kaili Chang, Hailan Zhao, Wei Zhao, and Hualei Cui
Correspondence should be addressed to Hualei Cui; chlyfjp@163.com

The PDF file includes:
Supplementary Figures: S1 to S8.
Supplementary Tables: T1 to T2.

Additional notes: The module gene results obtained from the WGCNA analysis and the results from the GDSC database analysis are uploaded as separate PDF files as supplementary material due to the size of the files. The file names of the PDFs are GDSC_IC50 and WGCNAmodulegene respectively.

Supplementary Figure S1

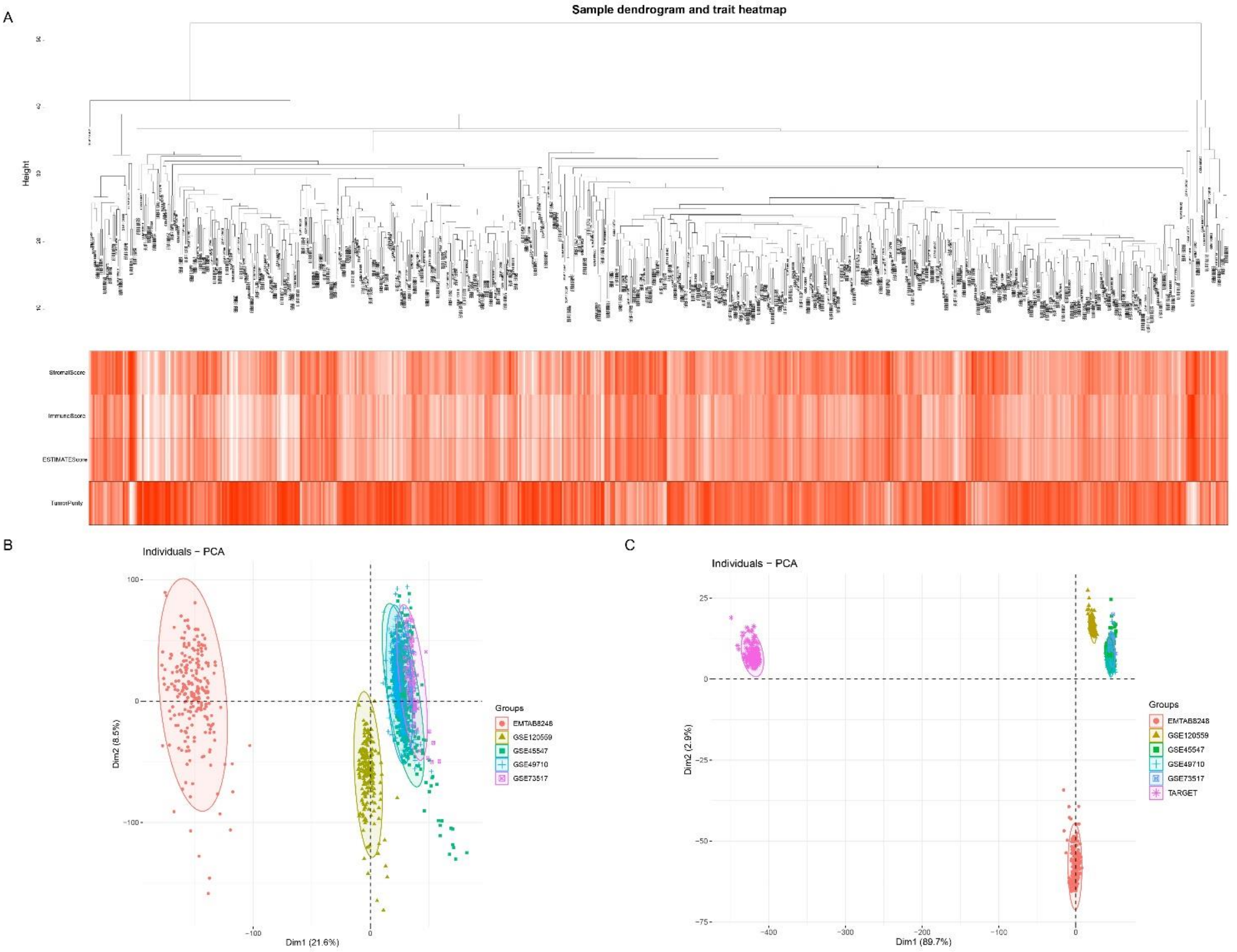

Supplementary Figure S1: Processing of data.

(S1A) From all the sample clusters, there are no outliers or abnormal values. Clustering dendrogram of samples based on their Euclidean distance.

(S1B, C) Principal component analysis (PCA) of the gene expression in datasets. The visualization of patients by scatter plots were based on the top two Dims of gene expression profiles without the removal of batch effect.

Supplementary Figure S2

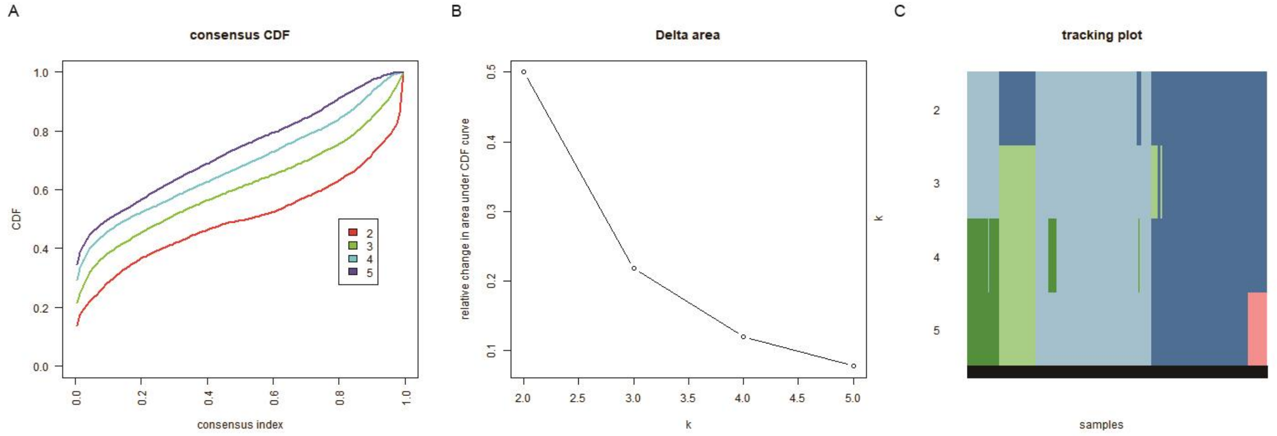

Supplementary Figure S2: The evidence for determining K values by cluster analysis.
(S2A) Consensus CDF with cluster count (k) =2–5.
(S2B) Relative alterations in the area under CDF curve with cluster count (k) =2–5.
(S2C) Tracking plot showing the sample classification with cluster count (k) =2–5.

Supplementary Figure S3

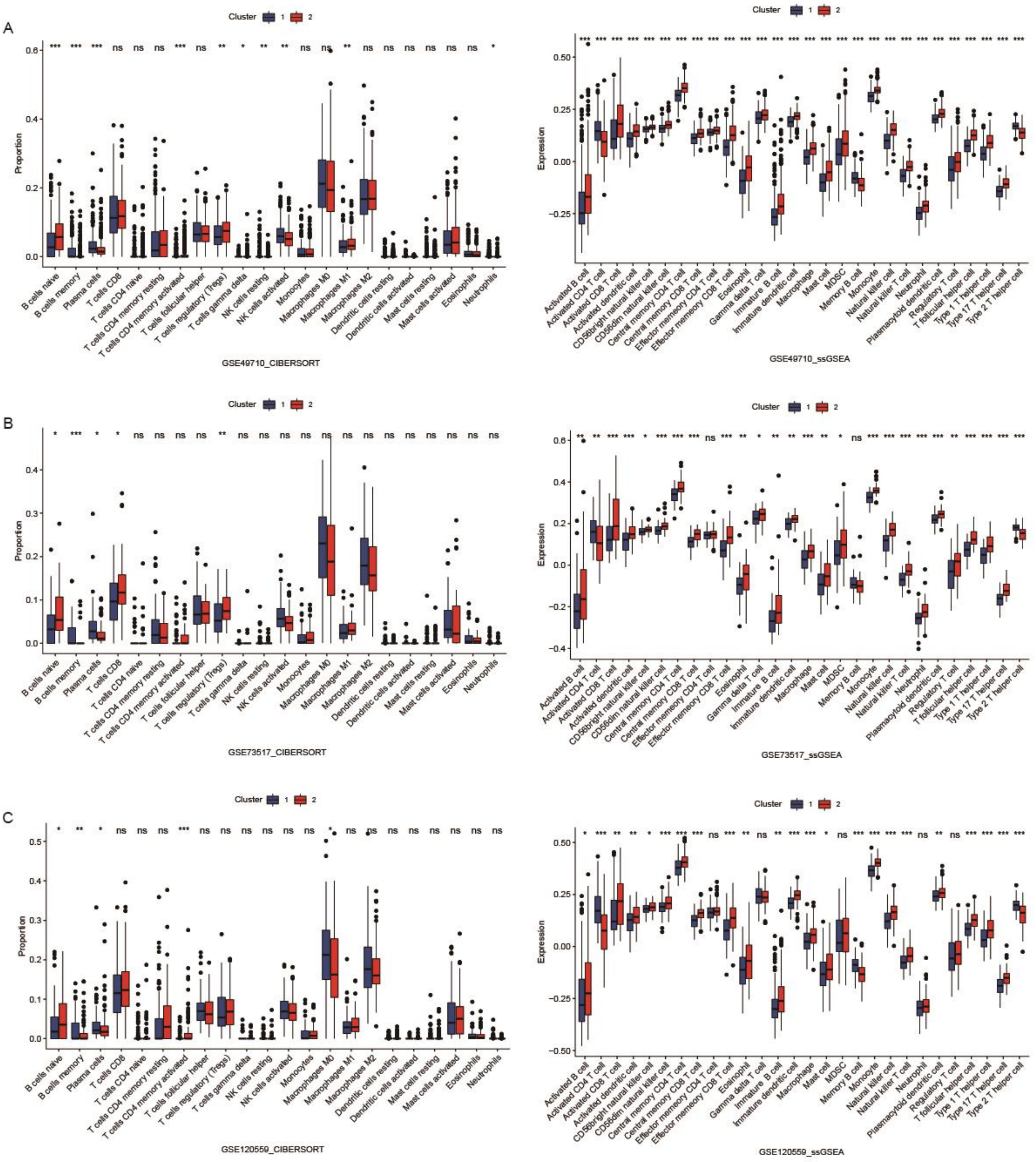

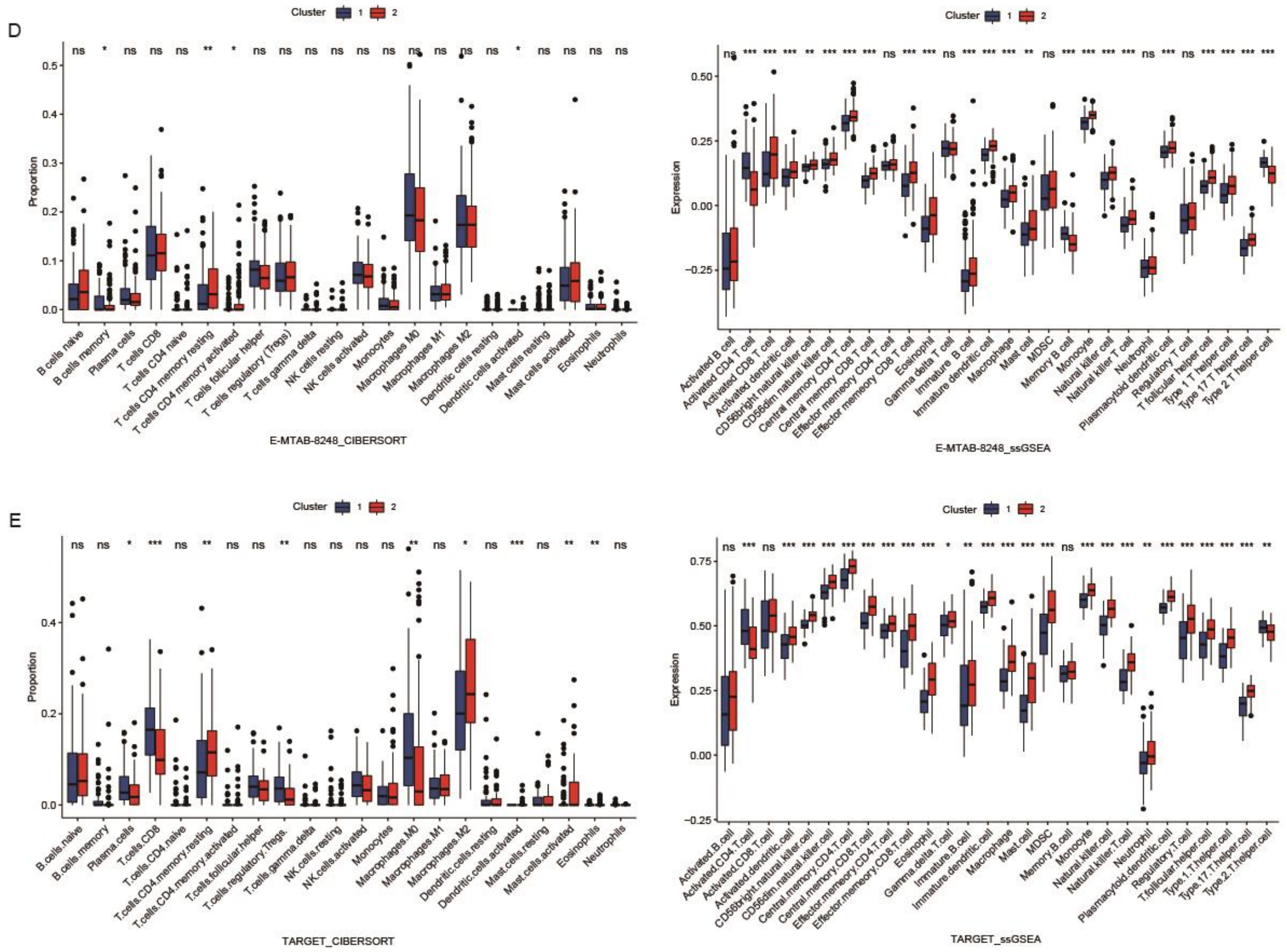

Supplementary Figure S3: Results of the immunity analysis for different clusters using different algorithms.
(S3A) Box plot of the distribution of immune cell expression between the two clusters as calculated by the the CIBERSORT algorithm and ssGSEA algorithm in GSE49710 dataset. (*: *P*<0.05; **: *P*<0.01; ***: *P*<0.001).
(S3B) Box plot of the distribution of immune cell expression between the two clusters as calculated by the the CIBERSORT algorithm and ssGSEA algorithm in GSE73517 dataset. (*: *P*<0.05; **: *P*<0.01; ***: *P*<0.001).
(S3C) Box plot of the distribution of immune cell expression between the two clusters as calculated by the the CIBERSORT algorithm and ssGSEA algorithm in GSE120559 dataset. (*: *P*<0.05; **: *P*<0.01; ***: *P*<0.001).
(S3D) Box plot of the distribution of immune cell expression between the two clusters as calculated by the the CIBERSORT algorithm and ssGSEA algorithm in E-MTAB-8248 dataset. (*: *P*<0.05; **: *P*<0.01; ***: *P*<0.001).
(S3E) Box plot of the distribution of immune cell expression between the two clusters as calculated by the the CIBERSORT algorithm and ssGSEA algorithm in TARGET dataset. (*: *P*<0.05; **: *P*<0.01; ***: *P*<0.001).

Supplementary Figure S4

A

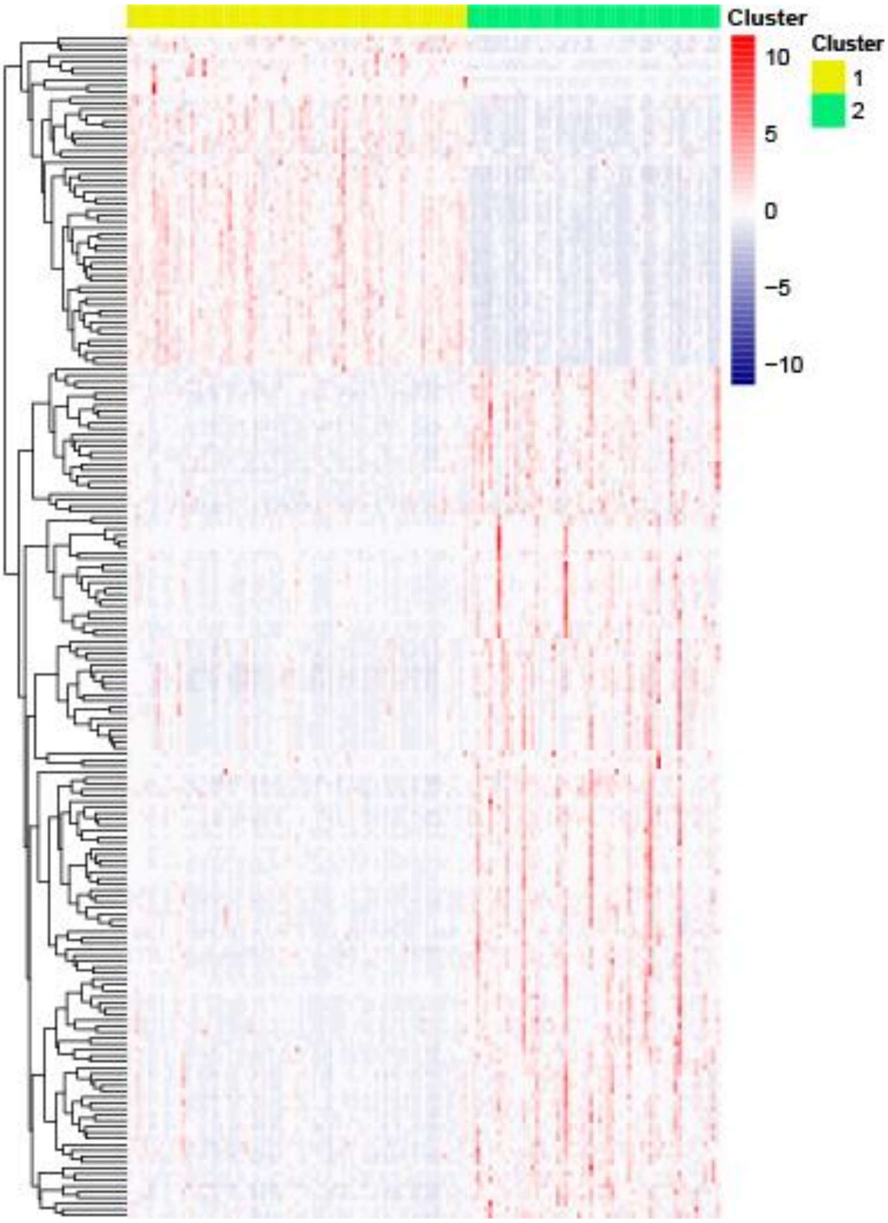

B

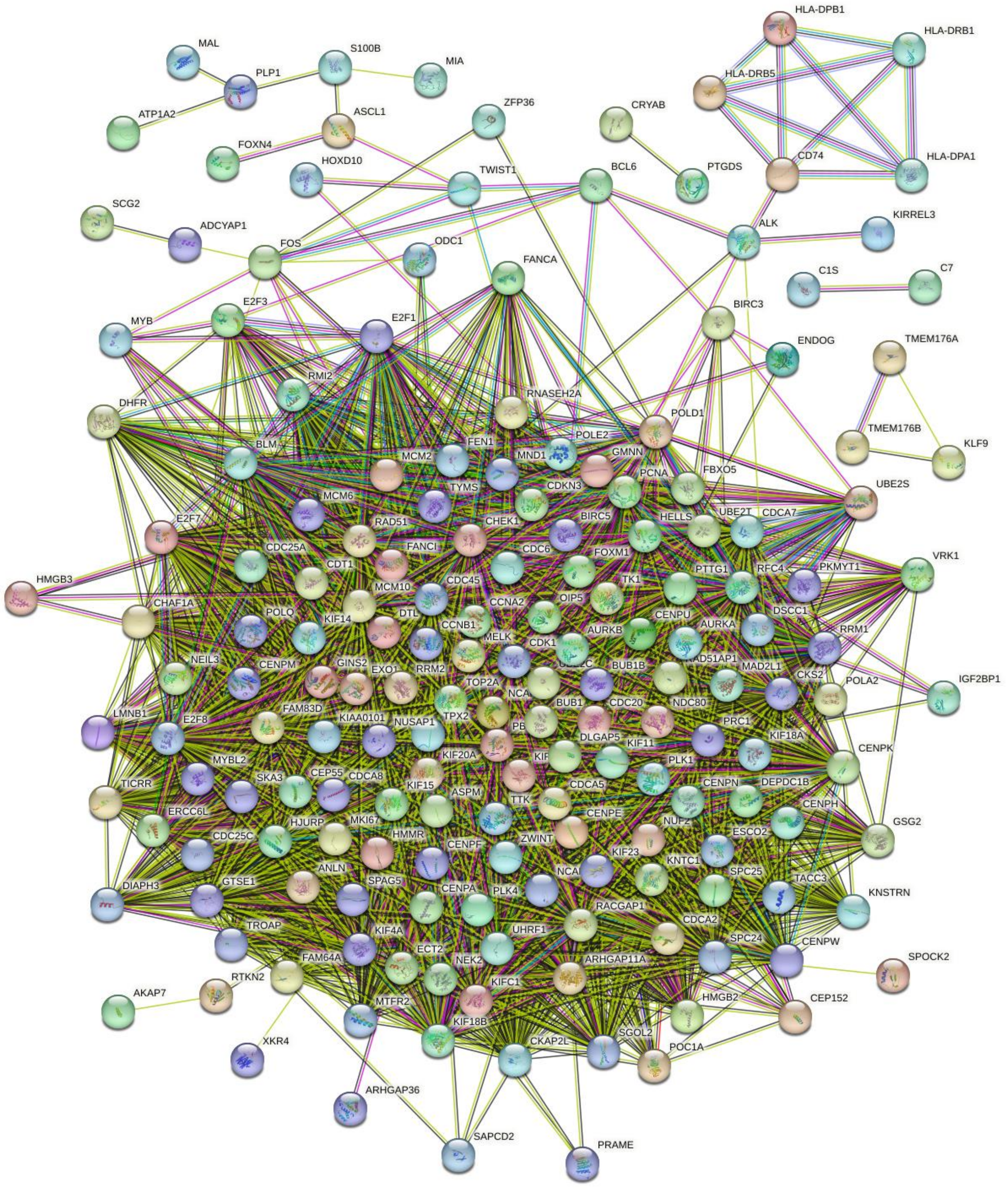

Supplementary Figure S4: Identification of differentially expressed genes.

(S4A) Heatmap showed differential gene expression in TARGET dataset. The genes shown in the heat map were the top 200 genes with the largest Median absolute deviation of gene expression.

(S4B) PPI network (206 DEGs) based on STRING database.

Supplementary Figure S5

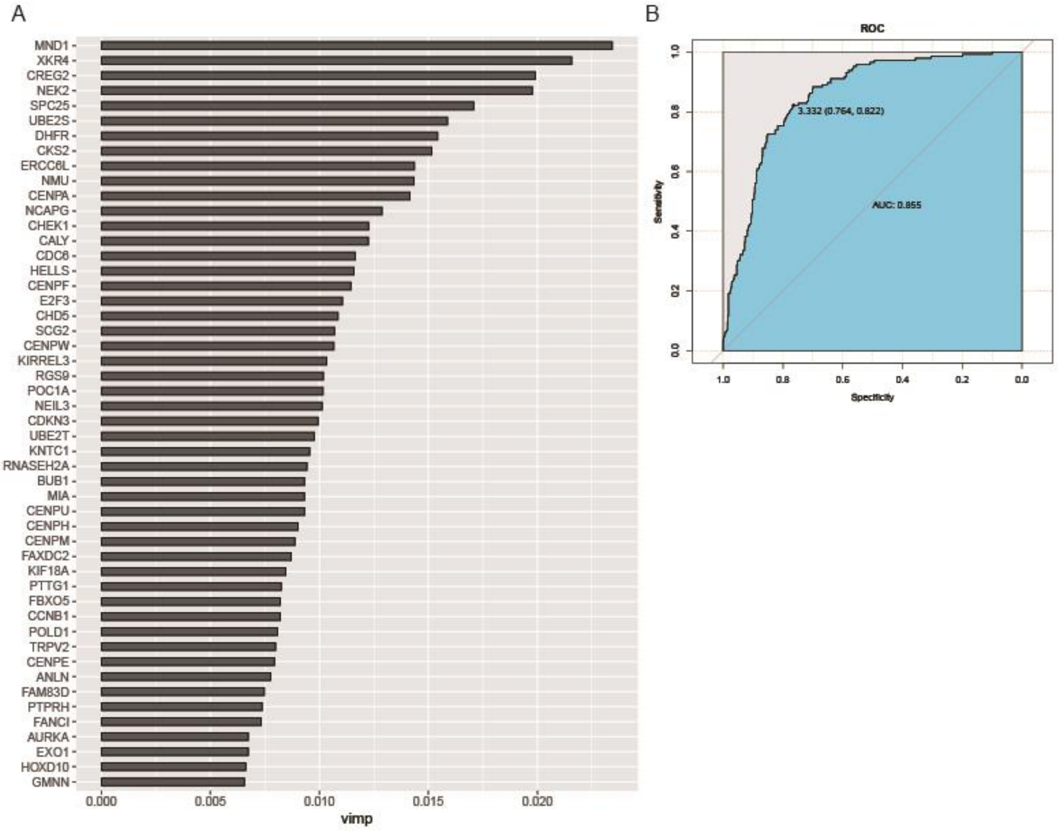

Supplementary Figure S5: Filtering for key genes and determining cut-off values.

(S5A) The bar chart showed the TOP 50 genes ranked according to the VIMP algorithm.

(S5B) Based on the ROC curves, we determined the optimal cutoff values for high and low risk groups.

Supplementary Figure S6

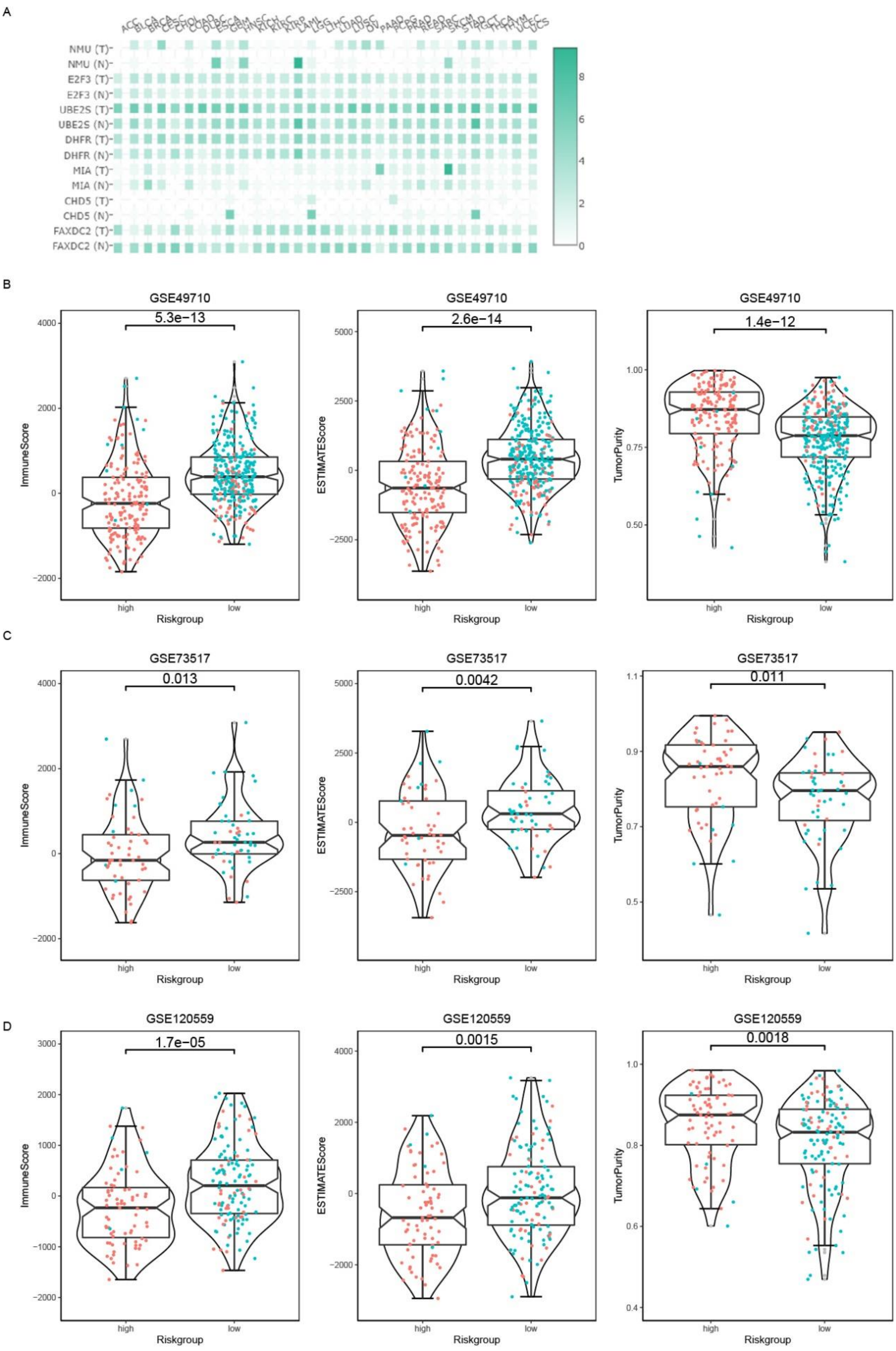

E

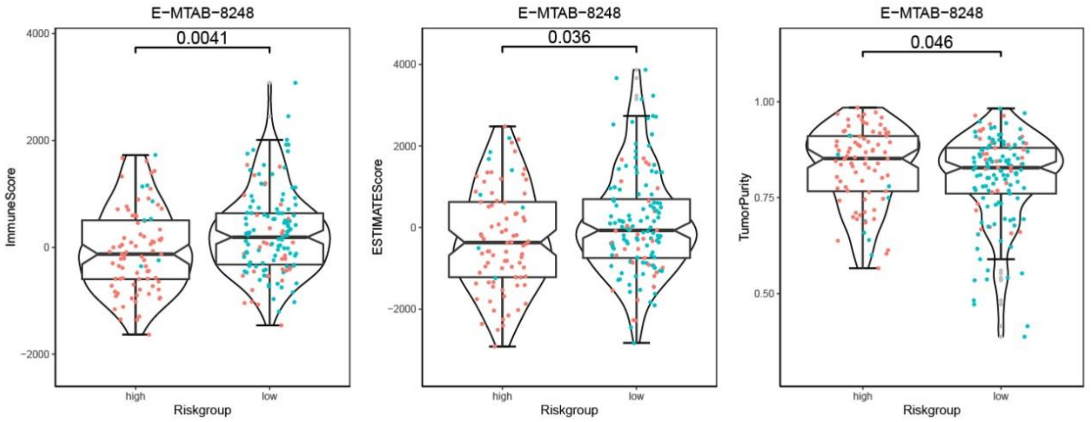

Supplementary Figure S6: The result of ESTIMATE algorithms in high and low risk groupings across different datasets.

(S6A) Expression of 7 risk score model genes in multiple tumors.

(S6B-E) Distribution of Immune Score, ESTIMATE Score, and Tumor Purity in high and low risk groups in the GSE49710 dataset (B), GSE73517 dataset (C), GSE120559 dataset (D) and E-MTAB-8248 dataset (E). Red dots represented patients belonging to Cluster 1, green dots represented patients belonging to Cluster 2.

Supplementary Figure S7

A

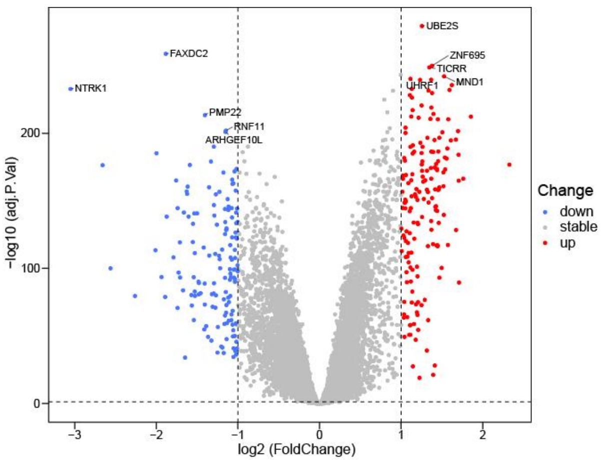

B

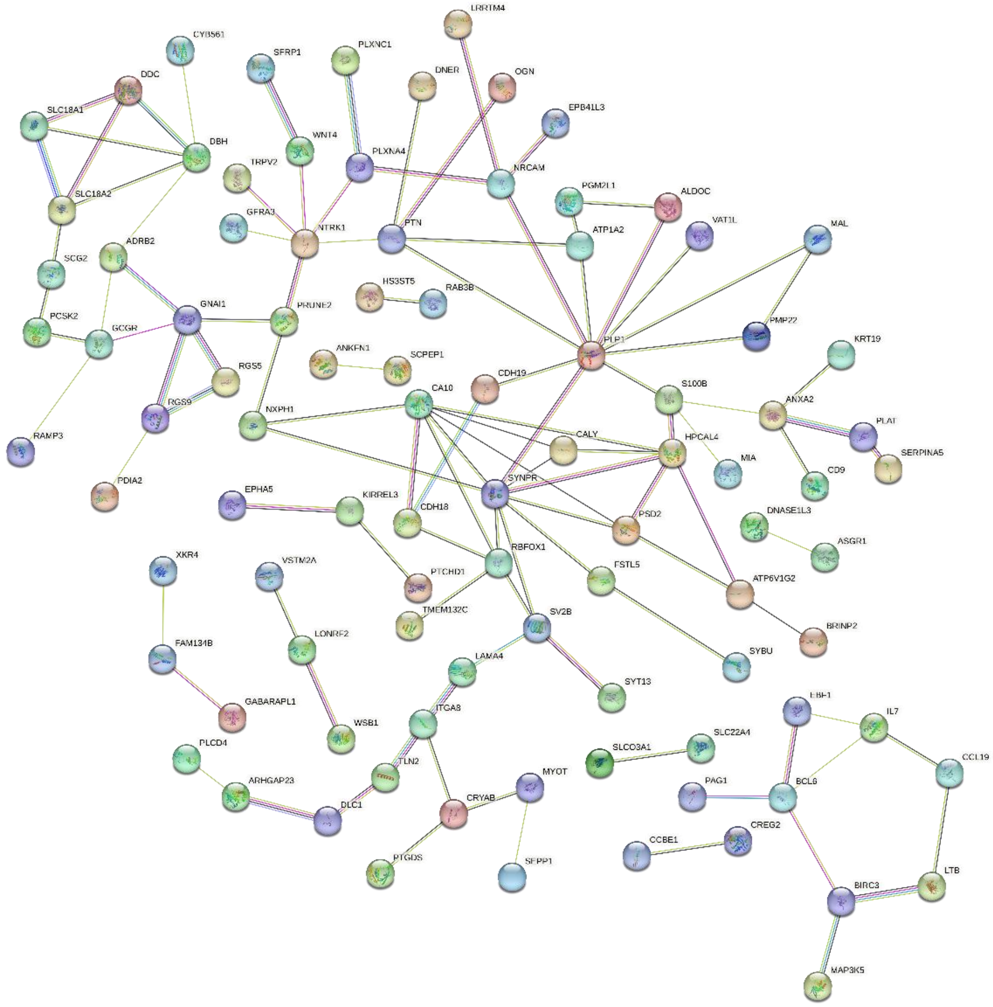

C

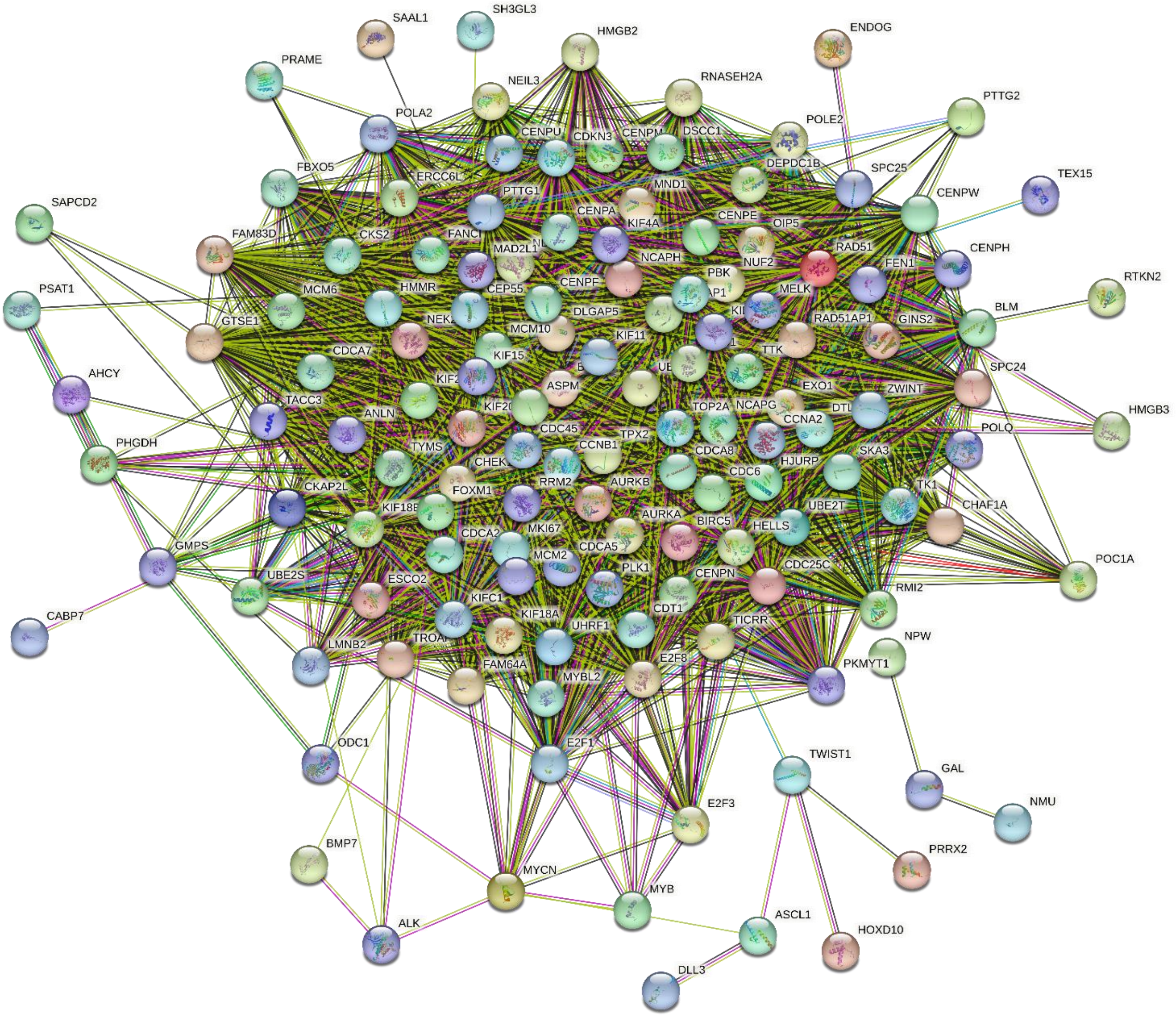

Supplementary Figure S7: Identification of differential expressed genes between risk subgroups.

(S7A) Volcano plot depicted the distribution of DEGs in 5 microarray datasets between the two risk groups. and labeled the top 5 genes with the smallest ranking according to adjusted p-value.

(S7B) PPI network (146 down DEGs) based on STRING database.

(S7C) PPI network (168 up DEGs) based on STRING database.

Supplementary Figure S8

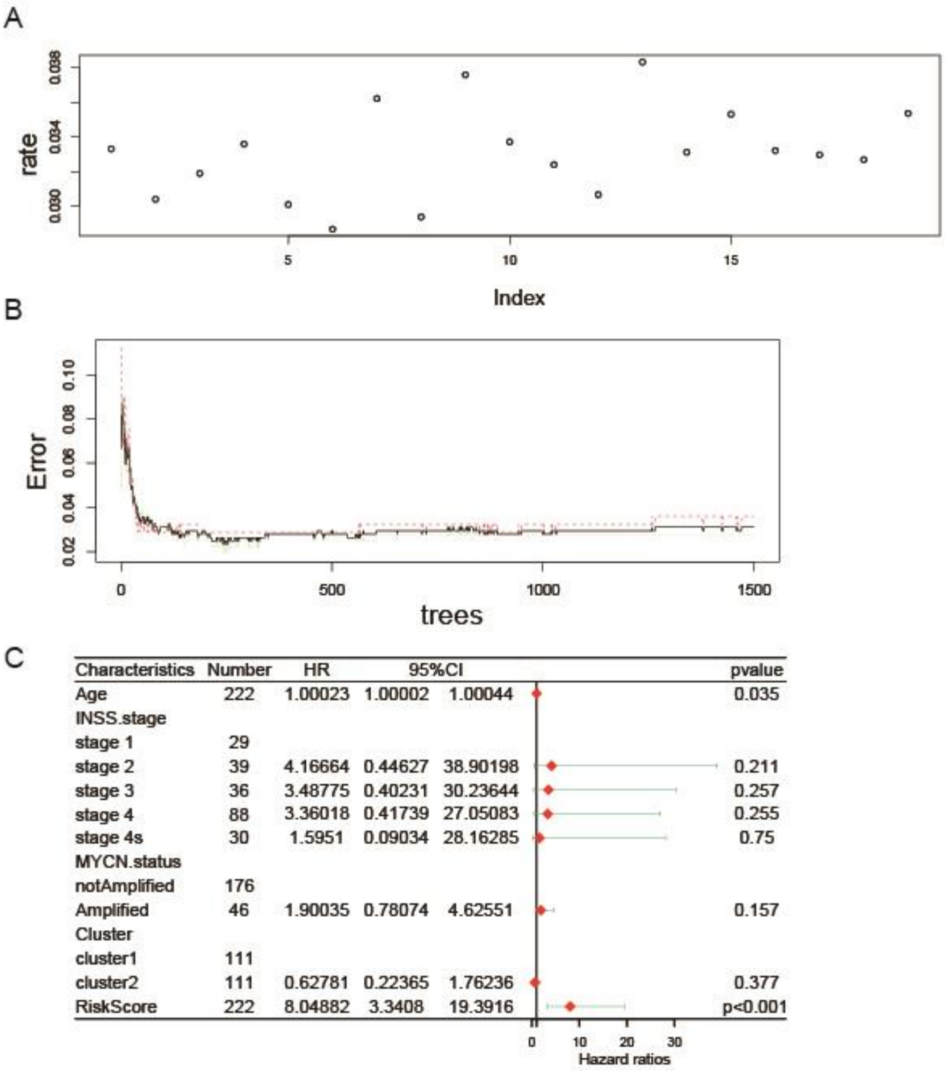

Supplementary Figure S8: Filtering of model parameters and forest plots for different characteristics.

(S8A) The scatter plot showed the relationship between the model error rate and mtry (1-20). The rate has the minimum value when mtry=6.

(S8B) The line graph showed the relationship between the model error and the number of trees.

(S8C) The forest plot showed the results of the multifactorial COX regression analysis.

Supplementary Table1

Supplementary Table1: 177 OS-related genes with a filtering threshold of P value less than 0.01 by univariate Cox regression analysis

Gene	HR	z	p value	Confidence intervals	
				lower	upper
MCM6	2.02167	3.793301	0.000149	1.405247	2.908492
KNSTRN	2.575466	4.100828	4.12E-05	1.638664	4.047825
S100B	0.743348	-3.82619	0.00013	0.638573	0.865315
DHFR	3.678661	5.929789	3.03E-09	2.391736	5.658044
RFC4	2.700155	4.374802	1.22E-05	1.7303	4.213626
HMGB2	2.31609	4.223919	2.40E-05	1.568567	3.419853
CCNA2	2.20543	4.629157	3.67E-06	1.577828	3.082668
OIP5	2.008274	4.087976	4.35E-05	1.437592	2.8055
SPC25	2.258719	5.217977	1.81E-07	1.663203	3.067462
CCNB1	2.289225	4.888488	1.02E-06	1.642393	3.190804
BLM	2.133814	4.767652	1.86E-06	1.562577	2.91388
ANGPTL7	0.802243	-3.66677	0.000246	0.713108	0.902519
KIF4A	2.124181	4.505143	6.63E-06	1.530547	2.94806
PLK4	2.149392	4.301442	1.70E-05	1.516684	3.046044
GMNN	2.538967	4.173264	3.00E-05	1.63912	3.932814
RMI2	1.828571	3.820673	0.000133	1.341687	2.492139
ERCC6L	2.298243	5.21433	1.84E-07	1.680948	3.142227
PCNA	2.268688	3.896448	9.76E-05	1.502503	3.42558
RACGAP1	2.100491	3.392136	0.000694	1.367996	3.225199
UBE2T	3.038879	5.175727	2.27E-07	1.994884	4.629234
FANCI	2.199865	4.813392	1.48E-06	1.595791	3.032608
DSCC1	2.04736	4.106247	4.02E-05	1.454311	2.882248
ECT2	1.970036	3.887674	0.000101	1.399637	2.772892
NEIL3	1.995585	4.551475	5.33E-06	1.482018	2.687119
PLP1	0.817332	-2.95665	0.00311	0.715036	0.934264
CDC6	2.256116	4.731184	2.23E-06	1.610557	3.160434
AURKA	2.525019	4.997669	5.80E-07	1.755926	3.630972
VRK1	2.339656	4.509836	6.49E-06	1.617035	3.385203
SGO2	2.080234	3.754077	0.000174	1.419153	3.049265
DEPDC1B	1.755407	3.876688	0.000106	1.320767	2.333081
CENPA	2.117897	4.992028	5.97E-07	1.577424	2.843553
CEP152	1.9733	4.813503	1.48E-06	1.496223	2.602496
CENPF	1.893485	4.641963	3.45E-06	1.446087	2.479302
POLE2	1.969096	4.119224	3.80E-05	1.426435	2.718203
MELK	1.956134	3.834338	0.000126	1.388184	2.75645
MAD2L1	1.689853	3.876306	0.000106	1.296112	2.203207
TYMS	1.984546	4.032728	5.51E-05	1.422308	2.769038

FBXO5	1.943087	3.464588	0.000531	1.334407	2.829411
RRM1	2.229627	3.607913	0.000309	1.442309	3.446721
CENPE	2.302164	4.710185	2.47E-06	1.627223	3.257058
ARHGAP11A	1.818816	3.724449	0.000196	1.327629	2.491729
KIF15	1.705447	3.820852	0.000133	1.29692	2.242658
CENPU	1.948694	4.340957	1.42E-05	1.441859	2.633688
KIF11	2.08023	4.071201	4.68E-05	1.462062	2.959763
CDK1	1.65246	3.987233	6.68E-05	1.290941	2.115219
RAD51	1.870139	3.795194	0.000148	1.353534	2.583917
FEN1	2.342345	5.044377	4.55E-07	1.682771	3.260443
RTKN2	1.71383	3.585837	0.000336	1.276687	2.300651
RNASEH2A	2.56279	4.862349	1.16E-06	1.753739	3.74508
NCAPH	1.866506	3.974539	7.05E-05	1.372071	2.539114
CENPH	2.007096	4.09248	4.27E-05	1.437681	2.802035
KIF18A	2.098483	4.47714	7.56E-06	1.516996	2.902864
GINS2	1.744308	3.615957	0.000299	1.290196	2.358255
POLA2	1.971709	3.872943	0.000108	1.398405	2.780052
NUF2	1.927533	4.200865	2.66E-05	1.419158	2.61802
CDC25A	1.673885	3.200258	0.001373	1.220976	2.294797
CHAF1A	2.002975	3.942633	8.06E-05	1.418098	2.829075
CDKN3	2.531475	5.065255	4.08E-07	1.767222	3.626236
NUSAP1	1.909215	3.799831	0.000145	1.367695	2.665144
NCAPG	2.212967	5.273263	1.34E-07	1.647232	2.973002
NDC80	1.995975	3.975136	7.03E-05	1.419587	2.806393
EXO1	1.91702	4.737728	2.16E-06	1.464556	2.509269
KLF9	0.664198	-2.8385	0.004533	0.500721	0.881048
PTTG1	2.16725	4.516955	6.27E-06	1.549365	3.031546
TICRR	1.942603	4.636978	3.54E-06	1.467201	2.572046
MCM2	1.823857	3.735594	0.000187	1.330626	2.499916
TACC3	2.164676	4.769228	1.85E-06	1.576013	2.973214
MCM10	2.021374	4.364983	1.27E-05	1.473687	2.772605
ASPM	1.863471	4.45892	8.24E-06	1.417423	2.449885
BUB1B	1.926768	4.059641	4.91E-05	1.403835	2.644496
ANLN	1.832232	3.451479	0.000558	1.299104	2.584145
KNTC1	2.073662	4.299852	1.71E-05	1.487175	2.891437
CENPW	2.655338	4.986382	6.15E-07	1.808897	3.897856
BUB1	2.286531	4.607167	4.08E-06	1.608338	3.2507
CEP55	2.023669	4.32724	1.51E-05	1.470543	2.784846
AURKB	1.583747	3.302491	0.000958	1.205525	2.080633
TPX2	2.059223	4.208209	2.57E-05	1.470946	2.88277
CDC25C	2.025894	4.269258	1.96E-05	1.465051	2.801436
MKI67	1.491107	2.970881	0.002969	1.145621	1.940783
TTK	1.682941	3.338137	0.000843	1.23975	2.284566

CENPK	1.75677	3.62144	0.000293	1.295008	2.383183
SKA3	1.902108	4.095139	4.22E-05	1.39827	2.587494
KIF2C	1.716761	3.356809	0.000788	1.252184	2.353702
ESCO2	1.576933	3.163607	0.001558	1.189217	2.091054
POLD1	3.269511	5.05995	4.19E-07	2.066325	5.17329
CDCA7	1.776951	3.999736	6.34E-05	1.340691	2.355169
RRM2	1.514068	2.825426	0.004722	1.135483	2.018879
KIF20A	1.91461	4.10775	4.00E-05	1.404394	2.610186
GTSE1	1.803093	3.492819	0.000478	1.295257	2.510039
E2F1	1.957875	4.35023	1.36E-05	1.446517	2.650005
PBK	1.547269	3.06743	0.002159	1.170687	2.044988
CDCA2	1.998605	4.292356	1.77E-05	1.456833	2.741852
KIF23	2.191568	4.198558	2.69E-05	1.519443	3.161008
PIMREG	1.607558	3.293279	0.00099	1.211905	2.132381
ZWINT	1.907133	3.764198	0.000167	1.36267	2.66914
POC1A	2.339188	4.53645	5.72E-06	1.620359	3.376905
CHEK1	1.904677	4.910626	9.08E-07	1.472777	2.463233
HMMR	1.823786	3.791949	0.000149	1.336851	2.488082
POLQ	1.763025	3.832167	0.000127	1.319196	2.356175
HMGB3	1.769509	3.477775	0.000506	1.282835	2.440814
ENDOG	1.766782	3.154596	0.001607	1.240529	2.516278
CENPN	2.142787	4.457943	8.27E-06	1.532717	2.995684
CDC45	1.976784	4.374156	1.22E-05	1.45662	2.6827
MND1	2.308266	5.692445	1.25E-08	1.730625	3.078709
PLK1	2.169357	4.189266	2.80E-05	1.510001	3.116626
CENPM	2.032455	4.092251	4.27E-05	1.447091	2.854606
CDCA8	1.732925	3.526363	0.000421	1.27663	2.352309
KIFC1	1.761941	3.596577	0.000322	1.29401	2.399081
CDC20	2.005704	3.933399	8.38E-05	1.417918	2.837152
SAPCD2	1.733565	4.222865	2.41E-05	1.34289	2.237895
DIAPH3	1.952516	3.982223	6.83E-05	1.404657	2.714057
SPC24	1.652999	3.872976	0.000108	1.281779	2.131728
E2F3	2.744449	5.925846	3.11E-09	1.965336	3.832424
DTL	1.83897	3.787014	0.000152	1.341666	2.520605
DLGAP5	2.000159	4.21092	2.54E-05	1.448554	2.761813
E2F8	1.774091	3.909915	9.23E-05	1.330973	2.364735
UBE2C	1.737346	3.890618	1.00E-04	1.315345	2.294738
KIF14	2.071223	4.444879	8.79E-06	1.502405	2.855399
HJURP	1.83554	4.030253	5.57E-05	1.36613	2.466241
UHRF1	2.079891	4.42322	9.72E-06	1.503538	2.87718
PCLAF	1.815204	3.018614	0.002539	1.232557	2.673277
SPAG5	1.723294	2.807017	0.005	1.178486	2.519963
KIF18B	1.754706	3.976815	6.98E-05	1.329991	2.315048

TK1	1.802341	3.771902	0.000162	1.327084	2.447797
BIRC5	1.79583	3.862028	0.000112	1.334217	2.417151
FAM83D	1.874003	4.401827	1.07E-05	1.416825	2.478703
PKMYT1	1.91357	3.536436	0.000406	1.33549	2.741877
BCL6	0.6086	-3.81739	0.000135	0.47163	0.785349
MIA	0.499478	-5.64212	1.68E-08	0.392452	0.635691
PHF21B	2.112416	4.224017	2.40E-05	1.49307	2.988675
HELLS	1.579788	5.023259	5.08E-07	1.32163	1.888372
CDCA5	1.881744	4.137923	3.50E-05	1.394802	2.538684
MTFR2	2.597345	4.845595	1.26E-06	1.765465	3.821203
FOXM1	1.836418	3.723759	0.000196	1.333622	2.528776
UBE2S	3.628362	6.023329	1.71E-09	2.385521	5.518715
HASPIN	1.782153	3.62604	0.000288	1.304076	2.435493
E2F7	1.881033	4.361975	1.29E-05	1.416126	2.498566
DIRAS3	0.739489	-3.96532	7.33E-05	0.637012	0.858451
MYBL2	1.969594	4.35787	1.31E-05	1.452045	2.671612
ZNF695	1.584512	4.134434	3.56E-05	1.273894	1.970869
TROAP	1.577749	3.020015	0.002528	1.173581	2.121107
CALY	0.597026	-4.97325	6.58E-07	0.487205	0.731601
NEK2	1.634094	4.668152	3.04E-06	1.32963	2.008275
HPDL	1.520504	3.707751	0.000209	1.218391	1.897528
CKS2	2.642814	5.339713	9.31E-08	1.849887	3.775617
TMEM97	1.795123	4.330518	1.49E-05	1.377504	2.339352
CDT1	1.637941	3.850369	0.000118	1.274129	2.105636
HLA-DRB1	0.722681	-2.57649	0.009981	0.564476	0.925225
FANCA	1.648934	3.442344	0.000577	1.240322	2.192159
SPOCK2	0.552269	-4.0579	4.95E-05	0.414581	0.735685
HOXD10	1.200684	3.286858	0.001013	1.076627	1.339036
SCG2	0.652166	-4.58963	4.44E-06	0.543352	0.782772
MAL	0.643809	-3.9807	6.87E-05	0.518316	0.799685
NMU	1.432406	5.999362	1.98E-09	1.273738	1.61084
ODC1	1.652767	4.382626	1.17E-05	1.320154	2.069181
CD74	0.73553	-2.64991	0.008051	0.586048	0.923139
ALK	1.605958	3.380298	0.000724	1.22024	2.1136
FAXDC2	0.478755	-5.76401	8.21E-09	0.372684	0.615015
TRPV2	0.584399	-4.59215	4.39E-06	0.464664	0.734987
XKR4	0.669755	-5.16636	2.39E-07	0.575272	0.779755
RGS9	0.765217	-4.16869	3.06E-05	0.674753	0.86781
ARHGAP36	0.881985	-2.73195	0.006296	0.805998	0.965136
EPB41L3	0.760049	-3.32663	0.000879	0.646602	0.893402
CGNL1	0.6198	-4.03777	5.40E-05	0.491371	0.781798
CHD5	0.687134	-5.60533	2.08E-08	0.602644	0.783468
IGSF9	1.611114	3.426861	0.000611	1.226485	2.116364

AKAP7	0.649615	-4.26259	2.02E-05	0.532739	0.792133
SV2B	0.770691	-3.47122	0.000518	0.665288	0.892795
KIRREL3	0.61406	-4.65395	3.26E-06	0.500055	0.754056
CREG2	0.626538	-5.2883	1.23E-07	0.526856	0.745081
AMIGO2	0.707917	-4.33301	1.47E-05	0.605515	0.827638
PTPRH	0.691221	-5.26059	1.44E-07	0.602369	0.79318
BIRC3	0.706718	-3.5249	0.000424	0.582669	0.857177
ASCL1	1.290406	3.479953	0.000502	1.117799	1.489667
PRAME	1.291478	3.281255	0.001033	1.108491	1.504671
TWIST1	1.383676	4.543715	5.53E-06	1.202813	1.591734
FSTL5	0.833022	-3.221	0.001277	0.745377	0.930972

Supplementary Table2

Supplementary Table2: Correspondence coefficients of risk model genes

Gene	Coefficient
UBE2S	0.241572950619027
DHFR	0.150059604933231
NMU	0.0984131905094628
E2F3	0.011081949073031
CHD5	-0.0563058944686111
MIA	-0.117424610155511
FAXDC2	-0.0767325162975187
