# Supplementary material for: Identification of the molecular subtypes and construction of risk models in neuroblastoma
Source: Sci Rep. 2023 Jul 21;13:11790. doi: 10.1038/s41598-023-35401-3 (PMC10362029; doi:10.1038/s41598-023-35401-3)
Supplement: Supplementary file 3 — Supplementary Information 3. [file 41598_2023_35401_MOESM3_ESM.pdf]

Result of WGCNA

Gene	ModuleColor
AAAS	grey
AATF	grey
ABCB1	grey
ABL1	grey
ABRAXAS1	turquoise
ABRAXAS2	turquoise
ACTB	grey
ACTL6A	turquoise
ACTL6B	grey
ACTR2	grey
ACTR3	turquoise
ACTR5	grey
ACTR8	turquoise
ACVR1	grey
ACVR1B	grey
ADAM17	grey
ADAMTS1	grey
ADARB1	turquoise
ADCYAP1	grey
AFAP1L2	turquoise
AGO4	grey
AHCTF1	turquoise
AHR	turquoise
AICDA	grey
AIF1	turquoise
AJUBA	turquoise
AKAP8	grey
AKAP8L	grey
AKT1	grey
AKT2	grey
ALKBH4	turquoise
ALMS1	turquoise
ALOX15B	grey
AMBRA1	grey
ANAPC1	turquoise
ANAPC10	turquoise
ANAPC11	grey
ANAPC13	turquoise
ANAPC15	grey
ANAPC16	grey
ANAPC2	grey
ANAPC4	turquoise
ANAPC5	turquoise
ANAPC7	turquoise
ANGEL2	turquoise
ANK3	grey
ANKFN1	grey
ANKK1	grey
ANKLE1	grey
ANKLE2	turquoise
ANKRD17	turquoise
ANKRD31	grey
ANKRD53	turquoise
ANLN	turquoise
ANXA1	grey
ANXA11	turquoise

APBB1	grey
APBB2	grey
APC	turquoise
APEX1	turquoise
APEX2	turquoise
APP	grey
APPL1	turquoise
APPL2	turquoise
ARAP1	turquoise
ARF1	turquoise
ARF6	grey
ARHGEF10	grey
ARHGEF2	grey
ARID1A	grey
ARID1B	grey
ARID2	turquoise
ARL2	turquoise
ARL3	grey
ARL8A	turquoise
ARL8B	grey
ARNTL	grey
ARPP19	turquoise
ASAH2	grey
ASCL1	turquoise
ASNS	turquoise
ASPM	turquoise
ASZ1	grey
ATAD5	turquoise
ATF2	turquoise
ATF5	grey
ATM	grey
ATP2B4	grey
ATR	turquoise
ATRIP	grey
ATRX	turquoise
AUNIP	turquoise
AURKA	turquoise
AURKAIP1	turquoise
AURKB	turquoise
AURKC	grey
AVEN	grey
AVPI1	turquoise
AXIN2	grey
AZI2	turquoise
BABAM1	grey
BABAM2	grey
BACH1	turquoise
BAG6	turquoise
BAK1	turquoise
BANF1	grey
BANP	grey
BAP1	grey
BARD1	turquoise
BAX	grey
BAZ1B	turquoise
BBS4	grey
BCAT1	turquoise
BCCIP	turquoise

BCL2	grey
BCL2L1	turquoise
BCL2L11	grey
BCL6	turquoise
BCL7A	grey
BCL7B	grey
BCL7C	turquoise
BCR	turquoise
BECN1	grey
BEX2	grey
BID	grey
BIN1	turquoise
BIN3	turquoise
BIRC2	turquoise
BIRC3	grey
BIRC5	turquoise
BIRC6	turquoise
BIRC7	turquoise
BIRC8	grey
BLCAP	turquoise
BLM	turquoise
BMP2	grey
BMP4	grey
BMP7	grey
BOD1	turquoise
BOD1L2	turquoise
BOLL	grey
BOP1	grey
BORA	turquoise
BRCA1	turquoise
BRCA2	turquoise
BRCC3	turquoise
BRD4	grey
BRD7	turquoise
BRD8	turquoise
BRDT	grey
BRINP1	grey
BRINP2	grey
BRINP3	grey
BRIP1	turquoise
BRME1	grey
BRSK1	grey
BRSK2	grey
BTBD18	grey
BTC	turquoise
BTG1	grey
BTG2	turquoise
BTG3	turquoise
BTG4	grey
BTN2A2	turquoise
BTRC	grey
BUB1	turquoise
BUB1B	turquoise
BUB3	turquoise
C2CD3	turquoise
CABLES1	grey
CABLES2	grey
CACNB4	turquoise

CACUL1	turquoise
CALM1	grey
CALM2	grey
CALM3	turquoise
CALR	turquoise
CAMK1	grey
CAMK2A	turquoise
CAPN3	turquoise
CASP2	grey
CASP3	turquoise
CASP8AP2	turquoise
CATSPERZ	grey
CAV2	grey
CCAR1	turquoise
CCAR2	grey
CCDC102B	grey
CCDC124	grey
CCDC57	turquoise
CCDC61	turquoise
CCDC69	turquoise
CCDC8	turquoise
CCL2	turquoise
CCN2	grey
CCNA1	grey
CCNA2	turquoise
CCNB1	turquoise
CCNB1IP1	turquoise
CCNB2	turquoise
CCNB3	grey
CCNC	turquoise
CCND1	turquoise
CCND2	turquoise
CCND3	turquoise
CCNDBP1	grey
CCNE1	grey
CCNE2	turquoise
CCNF	grey
CCNG1	turquoise
CCNG2	turquoise
CCNH	turquoise
CCNI	turquoise
CCNJ	turquoise
CCNJL	turquoise
CCNK	grey
CCNL1	turquoise
CCNL2	grey
CCNO	turquoise
CCNP	grey
CCNQ	turquoise
CCNT2	turquoise
CCNY	grey
CCNYL1	grey
CCP110	turquoise
CCPG1	grey
CCSAP	grey
CD28	grey
CD2AP	turquoise
CDC123	turquoise

CDC14A	grey
CDC14B	grey
CDC14C	grey
CDC16	turquoise
CDC20	turquoise
CDC23	turquoise
CDC25A	turquoise
CDC25B	grey
CDC25C	turquoise
CDC26	grey
CDC27	turquoise
CDC34	grey
CDC37	grey
CDC42	grey
CDC45	turquoise
CDC5L	turquoise
CDC6	turquoise
CDC7	turquoise
CDC73	turquoise
CDCA2	turquoise
CDCA3	grey
CDCA5	turquoise
CDCA8	turquoise
CDK1	turquoise
CDK10	grey
CDK11B	grey
CDK12	turquoise
CDK13	turquoise
CDK14	grey
CDK15	grey
CDK16	grey
CDK17	turquoise
CDK18	turquoise
CDK19	turquoise
CDK2	turquoise
CDK20	grey
CDK2AP1	turquoise
CDK2AP2	turquoise
CDK3	grey
CDK4	grey
CDK5	grey
CDK5R1	grey
CDK5R2	turquoise
CDK5RAP1	grey
CDK5RAP2	turquoise
CDK5RAP3	grey
CDK6	turquoise
CDK7	turquoise
CDK8	turquoise
CDK9	turquoise
CDKL1	grey
CDKL2	grey
CDKL3	turquoise
CDKL5	grey
CDKN1A	turquoise
CDKN1B	turquoise
CDKN1C	grey
CDKN2A	grey

CDKN2B	grey
CDKN2C	grey
CDKN2D	grey
CDKN3	turquoise
CDT1	turquoise
CEBPA	turquoise
CECR2	grey
CENATAC	grey
CENPA	turquoise
CENPC	turquoise
CENPE	turquoise
CENPF	turquoise
CENPH	turquoise
CENPJ	turquoise
CENPK	turquoise
CENPQ	turquoise
CENPS	grey
CENPT	grey
CENPV	turquoise
CENPW	turquoise
CENPX	grey
CEP120	turquoise
CEP126	grey
CEP131	grey
CEP135	turquoise
CEP152	turquoise
CEP164	turquoise
CEP192	turquoise
CEP250	turquoise
CEP295	turquoise
CEP44	turquoise
CEP55	turquoise
CEP63	turquoise
CEP68	turquoise
CEP72	turquoise
CEP76	turquoise
CEP85	grey
CEP97	turquoise
CETN1	grey
CETN2	grey
CETN3	turquoise
CFL1	grey
CGREF1	grey
CGRRF1	turquoise
CHAF1A	turquoise
CHAF1B	turquoise
CHAMP1	turquoise
CHD3	grey
CHEK1	turquoise
CHEK2	turquoise
CHFR	grey
CHMP1A	turquoise
CHMP1B	grey
CHMP2A	grey
CHMP2B	grey
CHMP3	grey
CHMP4A	grey
CHMP4B	grey

CHMP4C	grey
CHMP5	turquoise
CHMP6	turquoise
CHMP7	grey
CHORDC1	turquoise
CHTF18	grey
CHTF8	grey
CIB1	turquoise
CINP	grey
CIT	turquoise
CITED2	grey
CKAP2	turquoise
CKAP5	turquoise
CKS1B	turquoise
CKS2	turquoise
CLASP1	grey
CLASP2	turquoise
CLIC1	turquoise
CLIP1	grey
CLOCK	turquoise
CLSPN	turquoise
CLTA	grey
CLTC	turquoise
CLTCL1	grey
CNPPD1	turquoise
CNTD1	grey
CNTLN	turquoise
CNTRL	turquoise
CNTROB	grey
COPS5	turquoise
CORT	grey
CPSF3	turquoise
CRADD	grey
CREBL2	grey
CRLF3	grey
CRNN	grey
CROCC	grey
CRY1	turquoise
CSNK1A1	turquoise
CSNK1D	grey
CSNK2A1	turquoise
CSNK2A2	turquoise
CSPP1	turquoise
CTBP1	grey
CTC1	turquoise
CTCF	turquoise
CTCFL	grey
CTDNEP1	grey
CTDP1	turquoise
CTDSP1	turquoise
CTDSP2	grey
CTDSPL	grey
CTNNB1	grey
CUL1	turquoise
CUL2	turquoise
CUL3	turquoise
CUL4A	turquoise
CUL4B	turquoise

CUL5	grey
CUL7	grey
CUL9	grey
CUZD1	turquoise
CXCR5	turquoise
CYLD	grey
CYP1A1	grey
CYP26B1	grey
CYP27B1	grey
DAB2IP	turquoise
DACH1	grey
DACT1	grey
DAPK3	turquoise
DBF4	turquoise
DBF4B	grey
DCLRE1A	turquoise
DCTN1	grey
DCTN2	grey
DCTN3	grey
DCTN6	turquoise
DCUN1D3	grey
DDB1	turquoise
DDIAS	turquoise
DDIT3	grey
DDR2	grey
DDRGK1	turquoise
DDX11	turquoise
DDX39B	grey
DDX3X	turquoise
DDX4	grey
DEUP1	grey
DHCR24	grey
DIRAS3	grey
DIS3L2	turquoise
DLG1	turquoise
DLGAP5	turquoise
DMAP1	turquoise
DMC1	turquoise
DMRT1	turquoise
DMRTC2	grey
DMTF1	turquoise
DNA2	turquoise
DNM2	turquoise
DNMT3A	grey
DNMT3L	grey
DONSON	turquoise
DOT1L	grey
DPF1	grey
DPF2	grey
DPF3	grey
DR1	turquoise
DRD2	turquoise
DRD3	grey
DRG1	turquoise
DSCC1	turquoise
DSN1	turquoise
DTL	turquoise
DUSP1	turquoise

DUSP13	grey
DUSP3	turquoise
DYNC1H1	grey
DYNC1LI1	turquoise
DYNLT1	turquoise
DYNLT3	grey
DYRK3	grey
E2F1	turquoise
E2F2	turquoise
E2F3	turquoise
E2F4	grey
E2F5	turquoise
E2F6	turquoise
E2F7	turquoise
E2F8	turquoise
E4F1	turquoise
ECD	grey
ECRG4	turquoise
ECT2	turquoise
EDN1	turquoise
EDN3	grey
EDNRA	grey
EFHC1	grey
EFHC2	grey
EGF	grey
EGFR	turquoise
EHMT2	grey
EID1	turquoise
EIF2AK4	turquoise
EIF4E	turquoise
EIF4EBP1	grey
EIF4G1	grey
EIF4G2	turquoise
EME1	turquoise
EME2	grey
EML1	turquoise
EML3	turquoise
EML4	grey
ENSA	grey
ENTR1	grey
EP300	turquoise
EP400	grey
EPB41	turquoise
EPB41L2	grey
EPC1	turquoise
EPC2	turquoise
EPGN	grey
EPS8	grey
ERCC1	grey
ERCC2	turquoise
ERCC3	turquoise
ERCC4	grey
ERCC6	turquoise
ERCC6L	turquoise
EREG	grey
ERH	turquoise
ESCO1	turquoise
ESCO2	turquoise

ESPL1	turquoise
ESRRB	grey
ESX1	grey
ETAA1	turquoise
EVI2B	turquoise
EVI5	grey
EXD1	grey
EXO1	turquoise
EXOC1	turquoise
EXOC2	grey
EXOC3	grey
EXOC4	grey
EXOC5	turquoise
EXOC6	turquoise
EXOC6B	grey
EXOC7	grey
EXOC8	turquoise
EZH2	turquoise
EZR	grey
FAM107A	turquoise
FAM110A	grey
FAM32A	grey
FAM83D	turquoise
FAM9A	grey
FAM9B	grey
FAM9C	grey
FANCA	turquoise
FANCD2	turquoise
FANCI	turquoise
FANCM	turquoise
FAP	grey
FBXL12	grey
FBXL15	turquoise
FBXL17	grey
FBXL21P	grey
FBXL22	grey
FBXL3	grey
FBXL6	grey
FBXL7	grey
FBXL8	turquoise
FBXO31	turquoise
FBXO4	grey
FBXO43	turquoise
FBXO5	turquoise
FBXO6	turquoise
FBXO7	grey
FBXW11	turquoise
FBXW5	turquoise
FBXW7	turquoise
FEM1B	grey
FEN1	turquoise
FES	turquoise
FGF10	grey
FGF2	grey
FGF8	turquoise
FGFR2	grey
FHL1	grey
FIGN	turquoise

FIGNL1	turquoise
FKBP6	grey
FLCN	turquoise
FLNA	turquoise
FMN2	grey
FOSL1	grey
FOXA1	grey
FOXC1	grey
FOXE3	grey
FOXG1	grey
FOXJ2	turquoise
FOXJ3	grey
FOXM1	turquoise
FOXN3	turquoise
FOXO4	turquoise
FSD1	grey
FZD3	turquoise
FZD9	turquoise
FZR1	turquoise
GADD45A	grey
GADD45B	turquoise
GADD45G	grey
GADD45GIP1	grey
GAK	turquoise
GAS1	grey
GAS2	grey
GATA3	grey
GATA6	grey
GBF1	grey
GEM	grey
GEN1	turquoise
GFI1	turquoise
GFI1B	grey
GIGYF2	turquoise
GINS1	turquoise
GINS3	turquoise
GIPC1	turquoise
GIT1	turquoise
GJA1	grey
GJC2	turquoise
GLI1	turquoise
GML	turquoise
GMNN	turquoise
GNAI1	grey
GNAI2	turquoise
GNAI3	turquoise
GOLGA2	turquoise
GOLGA2P5	grey
GOLGA6B	grey
GOLGA8A	grey
GOLGA8B	grey
GOLGA8O	grey
GPER1	turquoise
GPNMB	grey
GPR132	turquoise
GPR3	grey
GPSM1	grey
GPSM2	turquoise

GRK5	grey
GSPT1	turquoise
GSPT2	grey
GTF2H1	turquoise
GTPBP4	turquoise
GTSE1	turquoise
H1-8	grey
H2AX	grey
HACE1	turquoise
HASPIN	turquoise
HAUS1	turquoise
HAUS2	turquoise
HAUS3	turquoise
HAUS4	grey
HAUS5	turquoise
HAUS6	turquoise
HAUS7	grey
HAUS8	turquoise
HBP1	grey
HCFC1	grey
HDAC3	grey
HDAC8	turquoise
HECA	grey
HECW2	grey
HELLS	turquoise
HEPACAM	turquoise
HEPACAM2	grey
HERC5	grey
HES1	turquoise
HEXIM1	turquoise
HEXIM2	grey
HFM1	turquoise
HGF	grey
HHEX	grey
HINFP	grey
HIPK2	grey
HJURP	turquoise
HLA-G	turquoise
HMCN1	grey
HMG20B	turquoise
HMGA2	grey
HNRNPU	turquoise
HORMAD1	grey
HORMAD2	grey
HOXA13	grey
HOXC9	grey
HOXD10	grey
HPGD	grey
HRAS	grey
HSF1	grey
HSF2BP	grey
HSP90AB1	turquoise
HSPA1A	grey
HSPA2	grey
HSPA8	turquoise
HTRA2	grey
HTT	grey
HUS1	grey

HUS1B	turquoise
HYAL1	grey
ID2	grey
ID3	turquoise
ID4	grey
IFFO1	turquoise
IFNW1	grey
IGF1	turquoise
IGF1R	grey
IGF2	grey
IHO1	grey
IK	turquoise
IKZF1	grey
IL10	grey
IL1A	grey
IL1B	turquoise
INCENP	grey
ING1	grey
ING2	turquoise
ING3	turquoise
ING4	grey
ING5	grey
INHA	grey
INHBA	grey
INIP	turquoise
INO80	turquoise
INO80B	grey
INO80C	grey
INO80D	grey
INO80E	grey
INS	grey
INSM1	grey
INSM2	grey
INSR	grey
INTS13	turquoise
INTS3	turquoise
INTS7	turquoise
IPO5	turquoise
IPO7	turquoise
IQGAP1	grey
IQGAP3	grey
IRF1	turquoise
IST1	grey
ITGB1	grey
ITGB3BP	turquoise
JADE1	turquoise
JADE2	turquoise
JADE3	grey
JTB	turquoise
JUN	grey
JUNB	turquoise
JUND	turquoise
KANK2	turquoise
KASH5	grey
KAT14	turquoise
KAT2A	grey
KAT2B	grey
KAT7	grey

KATNA1	turquoise
KATNB1	grey
KCNA5	turquoise
KCNH5	grey
KCTD11	turquoise
KDM8	grey
KHDRBS1	turquoise
KIAA0753	turquoise
KIAA1614	turquoise
KIF11	turquoise
KIF13A	grey
KIF14	turquoise
KIF15	turquoise
KIF18A	turquoise
KIF18B	turquoise
KIF20A	turquoise
KIF20B	turquoise
KIF22	turquoise
KIF23	turquoise
KIF25	grey
KIF2A	turquoise
KIF2B	grey
KIF2C	turquoise
KIF3A	grey
KIF3B	grey
KIF4A	turquoise
KIFC1	turquoise
KIFC2	grey
KIZ	turquoise
KLF11	grey
KLF4	turquoise
KLHDC3	grey
KLHDC8B	turquoise
KLHL13	turquoise
KLHL18	grey
KLHL21	turquoise
KLHL22	grey
KLHL42	turquoise
KLHL9	turquoise
KLK10	turquoise
KMT2E	grey
KMT5A	turquoise
KNL1	turquoise
KNSTRN	turquoise
KNTC1	turquoise
KPNB1	turquoise
KRT18	grey
L3MBTL1	grey
LATS1	grey
LATS2	turquoise
LCMT1	grey
LEP	grey
LFNG	turquoise
LGMN	turquoise
LIF	turquoise
LIG1	turquoise
LIG3	grey
LIG4	turquoise

LILRB1	turquoise
LIMK2	grey
LIN54	turquoise
LIN9	turquoise
LLGL1	grey
LLGL2	turquoise
LMLN	grey
LMNA	turquoise
LPIN1	grey
LRP5	grey
LRP6	turquoise
LRRCC1	grey
LSM10	turquoise
LSM11	grey
LSM14A	turquoise
LZTS1	grey
LZTS2	turquoise
M1AP	grey
MACROH2A1	turquoise
MAD1L1	turquoise
MAD2L1	turquoise
MAD2L1BP	grey
MAD2L2	turquoise
MADD	grey
MAEA	grey
MAEL	grey
MAGEA4	grey
MAJIN	grey
MAP10	turquoise
MAP1S	turquoise
MAP2K6	turquoise
MAP3K11	turquoise
MAP3K20	grey
MAP3K8	grey
MAP4	turquoise
MAP9	grey
MAPK1	grey
MAPK12	grey
MAPK13	grey
MAPK14	turquoise
MAPK15	turquoise
MAPK3	turquoise
MAPK4	grey
MAPK6	turquoise
MAPK7	grey
MAPRE1	turquoise
MAPRE2	grey
MAPRE3	turquoise
MARF1	grey
MARK4	turquoise
MASTL	turquoise
MAU2	grey
MBIP	turquoise
MBLAC1	turquoise
MBTD1	turquoise
MCM2	turquoise
MCM3	turquoise
MCM4	turquoise

MCM5	grey
MCM6	turquoise
MCM7	turquoise
MCM8	turquoise
MCMBP	turquoise
MCMDC2	grey
MCPH1	turquoise
MCRS1	grey
MCTS1	turquoise
MDC1	grey
MDM1	turquoise
MDM2	grey
MDM4	turquoise
MEAF6	grey
MECOM	grey
MECP2	grey
MED1	turquoise
MEI1	turquoise
MEIOB	grey
MEIOC	grey
MEIS2	grey
MELK	turquoise
MEN1	grey
MEPCE	turquoise
METTL13	grey
METTL3	turquoise
MICAL3	grey
MIIP	turquoise
MIR137HG	grey
MIS12	turquoise
MIS18A	turquoise
MIS18BP1	turquoise
MISP	grey
MITD1	turquoise
MKI67	turquoise
MLF1	grey
MLH1	turquoise
MLH3	turquoise
MN1	turquoise
MNAT1	turquoise
MND1	turquoise
MNS1	turquoise
MNT	turquoise
MOK	grey
MORF4L1	turquoise
MORF4L2	turquoise
MOS	grey
MOV10L1	turquoise
MPLKIP	turquoise
MRE11	turquoise
MRGBP	grey
MRGPRX2	grey
MRNIP	grey
MRPL41	turquoise
MS4A3	grey
MSH2	turquoise
MSH4	grey
MSH6	turquoise

MSX1	turquoise
MSX2	grey
MTA3	turquoise
MTBP	turquoise
MUC1	turquoise
MUS81	grey
MX2	grey
MYB	turquoise
MYBBP1A	turquoise
MYBL1	turquoise
MYBL2	turquoise
MYC	turquoise
MYH10	turquoise
MYH9	turquoise
MYO16	grey
MYO19	turquoise
MYOCD	grey
MYOG	turquoise
MZT1	turquoise
NAA10	grey
NAA50	turquoise
NABP1	grey
NABP2	grey
NACC2	turquoise
NAE1	turquoise
NANOS3	grey
NASP	turquoise
NAT10	grey
NBN	turquoise
NCAPD2	turquoise
NCAPD3	turquoise
NCAPG	turquoise
NCAPG2	turquoise
NCAPH	turquoise
NCAPH2	grey
NCOR1	turquoise
NDC1	turquoise
NDC80	turquoise
NDE1	grey
NDEL1	turquoise
NEDD1	turquoise
NEDD9	grey
NEK1	turquoise
NEK10	grey
NEK11	grey
NEK2	turquoise
NEK3	turquoise
NEK4	turquoise
NEK6	turquoise
NEK7	grey
NEK9	turquoise
NES	grey
NEUROG1	grey
NF2	grey
NFRKB	grey
NIN	grey
NIPBL	turquoise
NKX3-1	turquoise

NLE1	grey
NLRP5	grey
NME6	grey
NOLC1	turquoise
NOP53	grey
NOX5	grey
NPAT	turquoise
NPM1	turquoise
NPM2	grey
NPPC	grey
NPR2	grey
NR2E1	grey
NR2F2	turquoise
NR3C1	grey
NR4A1	turquoise
NRDE2	turquoise
NSFL1C	grey
NSL1	turquoise
NSMCE2	turquoise
NSUN2	turquoise
NTMT1	grey
NUBP1	grey
NUDC	turquoise
NUDT15	turquoise
NUDT16	grey
NUF2	turquoise
NUMA1	grey
NUP214	turquoise
NUP37	turquoise
NUP43	turquoise
NUP62	turquoise
NUP88	turquoise
NUPR1	turquoise
NUSAP1	turquoise
OBSL1	grey
ODF2	grey
OFD1	turquoise
OIP5	turquoise
OOEP	grey
OPN1LW	grey
OPN1MW	grey
OR1A2	grey
ORC1	turquoise
OSGIN2	grey
OVOL1	grey
P3H4	grey
PABIR1	grey
PAF1	grey
PAFAH1B1	grey
PAGR1	grey
PAK4	grey
PARD3	turquoise
PARD3B	turquoise
PARD6A	grey
PARD6B	turquoise
PARD6G	grey
PARP3	turquoise
PAX6	turquoise

PAXIP1	turquoise
PBK	turquoise
PBRM1	turquoise
PBX1	grey
PCID2	turquoise
PCLAF	turquoise
PCM1	turquoise
PCNA	turquoise
PCNP	turquoise
PCNT	turquoise
PDCD2L	grey
PDCD6IP	turquoise
PDE3A	grey
PDE4DIP	grey
PDGFB	turquoise
PDGFRB	turquoise
PDIK1L	turquoise
PDS5A	turquoise
PDS5B	turquoise
PDXP	grey
PELO	grey
PER2	grey
PES1	grey
PHACTR4	grey
PHB2	turquoise
PHF13	turquoise
PHF8	grey
PHGDH	grey
PHIP	turquoise
PHOX2B	grey
PIBF1	turquoise
PIDD1	grey
PIK3C3	turquoise
PIK3R4	turquoise
PIM1	grey
PIM2	turquoise
PIM3	turquoise
PIMREG	turquoise
PIN1	grey
PINX1	turquoise
PIWIL1	grey
PIWIL2	grey
PIWIL3	grey
PIWIL4	grey
PKD1	grey
PKD2	turquoise
PKHD1	grey
PKIA	grey
PKMYT1	turquoise
PKN2	grey
PKP4	grey
PLAGL1	grey
PLCB1	grey
PLCG2	turquoise
PLD6	grey
PLEC	turquoise
PLK1	turquoise
PLK2	grey

PLK3	turquoise
PLK4	turquoise
PLRG1	turquoise
PLSCR1	grey
PMF1	grey
PML	turquoise
PNPT1	turquoise
POC1B	turquoise
POC5	turquoise
POGZ	turquoise
POLA1	turquoise
POLDIP2	grey
POLE	grey
POU4F1	grey
PPM1A	turquoise
PPM1D	turquoise
PPM1G	grey
PPME1	grey
PPP1CA	grey
PPP1CB	turquoise
PPP1CC	turquoise
PPP1R10	grey
PPP1R12A	turquoise
PPP1R13B	grey
PPP1R15A	turquoise
PPP1R1C	grey
PPP1R35	grey
PPP1R9B	turquoise
PPP2CA	turquoise
PPP2CB	grey
PPP2R1A	turquoise
PPP2R2D	grey
PPP2R3B	grey
PPP2R5B	turquoise
PPP3CA	turquoise
PPP5C	grey
PPP6C	turquoise
PRAP1	grey
PRC1	turquoise
PRCC	grey
PRDM11	turquoise
PRDM5	grey
PRDM7	grey
PRDM9	grey
PRKACA	turquoise
PRKCA	grey
PRKCB	grey
PRKCD	turquoise
PRKCE	grey
PRKDC	turquoise
PRMT2	grey
PRMT5	turquoise
PRNP	grey
PROX1	grey
PRPF19	grey
PRPF40A	turquoise
PRR11	turquoise
PRR5	turquoise

PSMA8	grey
PSMC3IP	turquoise
PSMD10	turquoise
PSMD13	grey
PSME1	turquoise
PSME2	grey
PSME3	grey
PSMG2	turquoise
PSRC1	turquoise
PTCH1	grey
PTEN	turquoise
PTGS2	grey
PTK6	grey
PTP4A1	turquoise
PTPA	turquoise
PTPN11	turquoise
PTPN3	grey
PTPN6	turquoise
PTPRC	grey
PTPRK	grey
PTTG1	turquoise
PTTG2	turquoise
PTTG3P	turquoise
PUM1	grey
PYHIN1	grey
RAB11A	turquoise
RAB11FIP3	grey
RAB11FIP4	grey
RAB35	turquoise
RAB6C	grey
RABGAP1	grey
RACGAP1	turquoise
RACK1	grey
RAD1	turquoise
RAD17	turquoise
RAD21	turquoise
RAD23A	grey
RAD50	turquoise
RAD51	turquoise
RAD51AP1	turquoise
RAD51B	grey
RAD51C	turquoise
RAD51D	grey
RAD54B	turquoise
RAD54L	turquoise
RAD9A	grey
RAD9B	turquoise
RAE1	turquoise
RALA	grey
RALB	grey
RAN	turquoise
RANBP1	turquoise
RARA	turquoise
RASA1	turquoise
RASSF1	grey
RASSF2	grey
RASSF4	turquoise
RB1	turquoise

RB1CC1	turquoise
RBBP4	turquoise
RBBP8	turquoise
RBL1	turquoise
RBL2	turquoise
RBM14	grey
RBM38	turquoise
RBM7	turquoise
RCBTB1	turquoise
RCC1	turquoise
RCC2	grey
RDX	turquoise
REC114	turquoise
REC8	grey
RECQL5	grey
REEP3	grey
REEP4	grey
RFPL1	grey
RFWD3	turquoise
RGCC	turquoise
RGS14	turquoise
RGS2	grey
RHEB	grey
RHNO1	turquoise
RHOA	grey
RHOB	turquoise
RHOC	turquoise
RHOU	grey
RIDA	turquoise
RIF1	turquoise
RINT1	turquoise
RIOK2	turquoise
RMDN1	grey
RMI1	turquoise
RMI2	turquoise
RNASEH2B	turquoise
RNF112	grey
RNF167	grey
RNF2	turquoise
RNF20	turquoise
RNF212	grey
RNF4	grey
RNF40	grey
RNF8	turquoise
ROCK1	grey
ROCK2	turquoise
ROPN1B	grey
RPA1	turquoise
RPA2	grey
RPA3	turquoise
RPA4	turquoise
RPL10L	grey
RPL23	turquoise
RPL24	grey
RPL26	turquoise
RPRD1B	turquoise
RPRM	grey
RPS15A	turquoise

RPS27L	grey
RPS3	grey
RPS6	grey
RPS6KA1	turquoise
RPS6KA2	grey
RPS6KA3	grey
RPS6KB1	turquoise
RPTOR	grey
RRM1	turquoise
RRM2	turquoise
RRM2B	grey
RRP8	grey
RRS1	grey
RSPH1	grey
RTEL1	grey
RTF2	grey
RTKN	turquoise
RTTN	turquoise
RUNX3	turquoise
RUVBL1	turquoise
RUVBL2	grey
RXFP3	turquoise
SAC3D1	grey
SAPCD2	turquoise
SASS6	turquoise
SBDS	grey
SCRIB	grey
SDCBP	grey
SDCCAG8	grey
SDE2	grey
SEH1L	turquoise
SENP5	turquoise
SENP6	turquoise
SEPTIN1	turquoise
SEPTIN10	grey
SEPTIN11	turquoise
SEPTIN12	grey
SEPTIN14	grey
SEPTIN2	turquoise
SEPTIN3	grey
SEPTIN4	turquoise
SEPTIN5	grey
SEPTIN6	grey
SEPTIN7	turquoise
SEPTIN8	grey
SEPTIN9	grey
SERTAD1	turquoise
SETD2	turquoise
SETDB2	turquoise
SETMAR	turquoise
SFN	turquoise
SFPQ	turquoise
SFRP1	grey
SGF29	grey
SGO1	turquoise
SGO2	turquoise
SGSM3	turquoise
SH2B1	turquoise

SH3GLB1	grey
SHB	turquoise
SHCBP1L	grey
SHOC1	grey
SIAH1	turquoise
SIAH2	grey
SIK1	turquoise
SIN3A	grey
SIPA1	turquoise
SIRT1	turquoise
SIRT2	turquoise
SIRT7	grey
SIX3	grey
SKA1	turquoise
SKA2	turquoise
SKA3	turquoise
SKIL	turquoise
SKP2	turquoise
SLC16A1	turquoise
SLC25A31	grey
SLC26A8	grey
SLC2A8	turquoise
SLC39A5	grey
SLC6A4	grey
SLC9A3R1	turquoise
SLF1	turquoise
SLF2	turquoise
SLFN11	grey
SLX4	grey
SMARCA2	grey
SMARCA4	turquoise
SMARCA5	turquoise
SMARCAD1	turquoise
SMARCB1	grey
SMARCC1	turquoise
SMARCC2	grey
SMARCD1	grey
SMARCD2	grey
SMARCD3	turquoise
SMARCE1	turquoise
SMC1A	turquoise
SMC1B	grey
SMC2	turquoise
SMC3	turquoise
SMC4	turquoise
SMC5	turquoise
SMIM22	grey
SMOC2	turquoise
SMPD3	grey
SND1	grey
SNX18	turquoise
SNX33	turquoise
SNX9	grey
SON	turquoise
SOX15	grey
SOX2	turquoise
SOX9	grey
SPAG5	turquoise

SPAG8	grey
SPAST	turquoise
SPATA22	grey
SPC24	turquoise
SPC25	turquoise
SPDL1	turquoise
SPDYA	grey
SPDYE1	grey
SPDYE3	grey
SPDYE4	grey
SPDYE5	grey
SPDYE6	grey
SPDYE7P	grey
SPECC1L	grey
SPHK1	turquoise
SPICE1	turquoise
SPIN1	turquoise
SPIN2B	grey
SPIRE1	turquoise
SPIRE2	grey
SPO11	grey
SPOUT1	turquoise
SPRY1	turquoise
SPRY2	grey
SPTBN1	grey
SRA1	grey
SRC	turquoise
SRPK2	grey
SRSF5	grey
SSTR5	grey
SSX2IP	turquoise
STAG1	turquoise
STAG2	turquoise
STAG3	grey
STAG3L1	grey
STAG3L3	grey
STAG3L4	turquoise
STAMBP	turquoise
STARD9	grey
STAT3	turquoise
STAT5B	grey
STEAP3	turquoise
STIL	turquoise
STK10	turquoise
STK11	turquoise
STK33	grey
STK35	grey
STMN1	turquoise
STOX1	grey
STRA8	grey
STRADA	grey
STRADB	turquoise
STXBP4	turquoise
SUGT1	turquoise
SUN1	grey
SUN2	turquoise
SUSD2	turquoise
SUV39H1	grey

SUV39H2	turquoise
SVIL	grey
SYCE1	grey
SYCE3	grey
SYCP1	grey
SYCP2	grey
SYCP2L	grey
SYCP3	grey
SYF2	grey
TACC1	turquoise
TACC2	grey
TACC3	turquoise
TADA2A	turquoise
TADA3	grey
TAF1	turquoise
TAF10	grey
TAF1L	turquoise
TAF2	turquoise
TAF6	grey
TAL1	turquoise
TAOK1	grey
TAOK2	turquoise
TAOK3	turquoise
TARDBP	turquoise
TAS1R2	grey
TAS2R13	grey
TASOR	turquoise
TBCD	grey
TBCE	turquoise
TBRG1	grey
TBRG4	grey
TBX2	grey
TBX3	grey
TCIM	turquoise
TDRD1	grey
TDRD12	grey
TDRD9	grey
TDRKH	turquoise
TELO2	turquoise
TENT4A	turquoise
TENT4B	turquoise
TENT5B	turquoise
TERB1	grey
TERB2	grey
TERF1	turquoise
TERF2	grey
TERT	grey
TESMIN	grey
TET2	grey
TEX11	grey
TEX12	turquoise
TEX14	grey
TEX15	turquoise
TEX19	grey
TFAP4	grey
TFDP1	turquoise
TFDP2	turquoise
TFDP3	turquoise

TFPT	turquoise
TGFA	turquoise
TGFB1	turquoise
TGFB2	grey
TGFBR1	turquoise
TGM1	grey
THAP1	turquoise
THAP5	turquoise
THOC1	turquoise
THOC5	grey
TICRR	turquoise
TIMELESS	turquoise
TIPIN	turquoise
TIPRL	turquoise
TLE6	grey
TLK1	turquoise
TLK2	turquoise
TM4SF5	grey
TMEM14B	turquoise
TMEM250	turquoise
TMEM67	grey
TMEM8B	turquoise
TMOD3	grey
TMPRSS11A	grey
TNF	grey
TNFAIP3	turquoise
TNKS	turquoise
TOGARAM1	turquoise
TOGARAM2	grey
TOM1L1	grey
TOM1L2	turquoise
TOP2A	turquoise
TOP2B	turquoise
TOP3A	grey
TOPBP1	turquoise
TP53	grey
TP53BP1	turquoise
TP53BP2	turquoise
TP53I13	turquoise
TP53INP1	grey
TP73	turquoise
TPD52L1	grey
TPPP	grey
TPR	turquoise
TPRA1	turquoise
TPX2	turquoise
TRAPPC12	grey
TREX1	turquoise
TRIAP1	turquoise
TRIM21	turquoise
TRIM32	grey
TRIM35	turquoise
TRIM36	grey
TRIM37	turquoise
TRIM39	grey
TRIOBP	turquoise
TRIP13	turquoise
TRNP1	turquoise

TRRAP	grey
TSC1	grey
TSC2	grey
TSG101	turquoise
TSPYL2	turquoise
TTBK1	turquoise
TTC19	grey
TTC28	turquoise
TTI1	turquoise
TTI2	grey
TTK	turquoise
TTL	grey
TTLL12	grey
TTN	grey
TTYH1	turquoise
TUBA1A	grey
TUBA1B	grey
TUBA1C	grey
TUBA3C	grey
TUBA3D	grey
TUBA4A	turquoise
TUBA8	turquoise
TUBAL3	grey
TUBB	grey
TUBB1	grey
TUBB2A	turquoise
TUBB2B	grey
TUBB3	grey
TUBB4A	turquoise
TUBB4B	grey
TUBB6	turquoise
TUBB8	grey
TUBD1	turquoise
TUBE1	turquoise
TUBG1	grey
TUBG2	grey
TUBGCP2	grey
TUBGCP3	turquoise
TUBGCP4	turquoise
TUBGCP5	turquoise
TUBGCP6	turquoise
TUSC2	turquoise
TXLNG	turquoise
TXNIP	grey
TXNL4A	grey
TXNL4B	grey
UBA3	turquoise
UBB	grey
UBD	grey
UBE2B	grey
UBE2C	turquoise
UBE2DNL	grey
UBE2E2	grey
UBE2I	grey
UBE2L3	grey
UBE2S	grey
UBR2	grey
UBXN2B	turquoise

UCHL5	turquoise
UHMK1	grey
UHRF1	turquoise
UHRF2	turquoise
UIMC1	turquoise
UNC119	turquoise
UPF1	grey
URGCP	turquoise
USH1C	grey
USP16	turquoise
USP17L2	turquoise
USP19	grey
USP2	grey
USP22	grey
USP26	grey
USP28	turquoise
USP29	grey
USP3	turquoise
USP33	turquoise
USP37	turquoise
USP39	turquoise
USP44	grey
USP47	turquoise
USP51	grey
USP8	turquoise
USP9X	turquoise
UTP14C	turquoise
UVRAG	grey
UXT	grey
VASH1	grey
VCP	grey
VCPIP1	grey
VPS4A	grey
VPS4B	grey
VPS72	grey
VRK1	turquoise
WAC	turquoise
WAPL	turquoise
WASHC5	turquoise
WASL	grey
WBP2NL	grey
WDHD1	turquoise
WDR12	turquoise
WDR5	grey
WDR6	grey
WDR62	grey
WDR76	turquoise
WEE1	turquoise
WEE2-AS1	grey
WIZ	grey
WNT10B	turquoise
WNT4	turquoise
WNT5A	grey
WRAP73	grey
WRN	turquoise
WTAP	turquoise
XIAP	grey
XPC	grey

XPO1	turquoise
XRCC2	turquoise
XRCC3	grey
YEATS2	turquoise
YEATS4	turquoise
YTHDC2	turquoise
YTHDF2	turquoise
YWHAE	turquoise
YY1	turquoise
YY1AP1	grey
ZBED3	grey
ZBED9	turquoise
ZBTB17	turquoise
ZBTB49	turquoise
ZC3HC1	turquoise
ZCWPW1	grey
ZFHX3	grey
ZFP36L1	turquoise
ZFP36L2	turquoise
ZFP42	grey
ZFYVE19	turquoise
ZFYVE26	grey
ZMPSTE24	grey
ZMYND11	turquoise
ZNF16	turquoise
ZNF207	turquoise
ZNF268	turquoise
ZNF318	grey
ZNF324	grey
ZNF503	grey
ZNF655	grey
ZNF703	turquoise
ZNF830	turquoise
ZNRD2	grey
ZPR1	grey
ZW10	turquoise
ZWILCH	turquoise
ZWINT	turquoise
ZZZ3	turquoise
CREBBP	grey
GSK3B	turquoise
HDAC1	grey
HDAC2	turquoise
ORC2	turquoise
ORC3	turquoise
ORC4	turquoise
ORC5	turquoise
ORC6	turquoise
RBX1	turquoise
SKP1	grey
SMAD2	turquoise
SMAD3	grey
SMAD4	turquoise
TGFB3	grey
YWHAB	grey
YWHAG	grey
YWHAH	grey
YWHAQ	turquoise

YWHAZ	grey

Gene:Name of Gene
ModuleColor:The module in which genes were divided
